# High energy density dihydroazaborinine dyads and triad for molecular solar thermal energy storage†

**DOI:** 10.1039/d5sc03159a

**Published:** 2025-07-25

**Authors:** Sonja M. Biebl, Robert C. Richter, Markus Ströbele, Ivana Fleischer, Holger F. Bettinger

**Affiliations:** a Institut für Organische Chemie, Eberhard Karls Universität Tübingen Auf der Morgenstelle 18 72076 Tübingen Germany holger.bettinger@uni-tuebingen.de; b Institut für Anorganische Chemie, Eberhard Karls Universität Tübingen Auf der Morgenstelle 18 72076 Tübingen Germany

## Abstract

The reversible photoisomerization of 1,2-dihydro-1,2-azaborinines (BN benzenes) to their Dewar isomers (2-aza-3-borabicyclo[2.2.0]hex-5-enes) provides a promising platform for molecular solar thermal (MOST) energy conversion, storage, and release. We examine how energy density can be optimized by bundling multiple dihydroazaborinine units into a single molecule and explore how properties change depending on the connectivity of these units. Remarkably high molar energy densities of up to 644 kJ mol^−1^ were obtained, as well as a significant decrease in the half-life of the storage state in the order of *ortho* > *meta* > *para*. Moreover, the absorption is shifted from the UV-C of the parent 1,2-dihydro-1,2-azaborinine into the UV-A region. The investigated dyads and triades meet several criteria for an ideal molecular solar thermal storage material.

## Abstract 2

The reversible photoisomerization of 1,2-dihydro-1,2-azaborinines (BN benzenes) to their Dewar isomers (2-aza-3-borabicyclo[2.2.0]hex-5-enes) provides a promising platform for molecular solar thermal (MOST) energy conversion, storage, and release.

## Graphical abstract

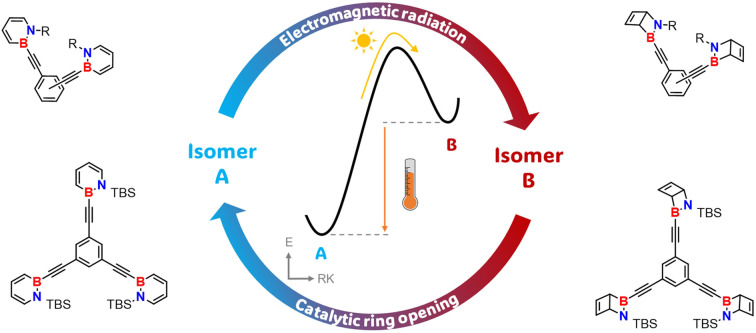

## Introduction

**Fig. 1:**
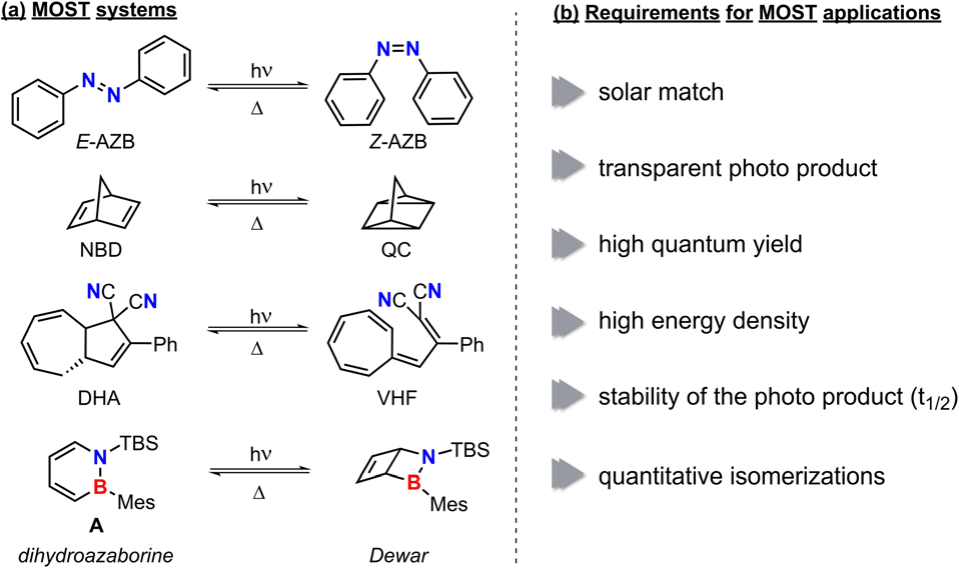
Examples of different MOST systems (a) and criteria for a potential application as MOST system (b). AZB: azobenzene, NBD: norbornadiene, QC: quadricyclane, DHA: dihydroazulene, VHF: vinylheptafulvene.

In an effort to combat climate change in accordance with a stable energy supply, developments of environmentally acceptable energy storage systems are indispensable. Switchable molecules that enable the storage of solar power by photoisomerization, as well as the release of the stored energy on demand for molecular solar thermal (MOST) energy storage are a vibrant research field.^1,2^ MOST compounds isomerize to a higher-energy metastable species upon energy input, usually in the form of electromagnetic radiation (Fig. 1a). Great promise for these applications is shown by 1,2-dihydro-1,2-azaborinines as a new member in the field.^3–5^ These six-membered aromatic heterocycles are isoelectronic to benzene.^6–8^ In contrast to the multichannel photoisomerization of their all-carbon analogue, the electrocyclic ring closure of 1,2-disubstituted 1,2-dihydro-1,2-azaborinine A occurs selectively to the corresponding Dewar isomer (2-aza-3-borabicyclo[2.2.0]hex-5-ene) upon irradiation with UV light (Fig. 1). By the introduction of aryl substituents *para* to the boron heteroatom Ozaki *et al.* recently reported the formation of BN-benzvalene (3-aza-4-boratricyclo[3.1.0.0]hexane) isomers as follow-up products.^9^ With regards to the remarkably high storage energy (47 kcal mol^−1^) of the Dewar isomer as well as the quantitative photoisomerization and catalytic ring opening (Wilkinson's catalyst and coinage metal Lewis acids) this compound class is promising for MOST energy storage.^3,5,10^

To comprise a MOST system, the considered compound must meet a series of requirements (Fig. 1b). In the first place (a) the ground state storage molecule should absorb in the visible spectrum (solar match),^11^ while (b) the resulting photoisomer should be transparent and therefore cause no competing absorption or photochemical reactions. After excitation the desired isomerization should proceed (c) selectively with high quantum yield.^12^ Once formed, high (d) gravimetric energy density and (e) stability of the metastable isomer are required.^13^ The latter must allow for thermal,^14^ catalytic^15^ or electrochemical^16^ reversion of the isomerization. Ultimately, (f) all considered reactions should proceed quantitatively to ensure high durability over many charge and discharge cycles (cyclability).^17^ Among the extensively studied norbornadienes and azobenzenes, many other substance classes like dihydroazulenes^18^ (Fig. 1a), certain fulvalene ruthenium complexes,^19^ anthracenes,^14^ or fluorinated acetophenones^20^ fulfill many of the criteria and thus are promising candidates for energy storage application.

**Fig. 2:**
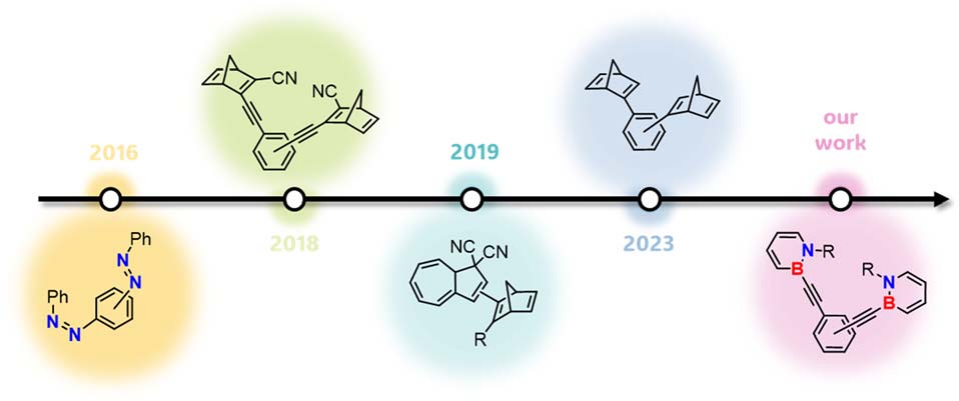
Representative oligomeric MOST systems from the last ten years incorporating azobenzenes,^27^ norbornadienes^28^ and dihydroazulenes.^31^

With sterically demanding substituents on the reactive boron and nitrogen atom, the properties of dihydroazaborinines already fulfill many requirements for MOST applications.^3^ However, a major challenge remains red shifting the absorption wavelength. Since photon energy decreases with increasing wavelength, bathochromic shifts toward the visible spectrum inherently counteract the optimization of energy density and may impose limitations due to the reduced excitation energy.^11,21^ Considering established MOST systems such as norbornadienes, diazo compounds and dihydroazulene, the incorporation of a donor–acceptor system could be a promising approach.^22,23^ However, due to the typically large acceptor and donor substituents, these compounds often exhibit increased molecular weights, which in turn reduces their energy density.^24^ Given that the molar energy density in established MOST systems shows only minor variation depending on the substitution pattern, the molar mass emerges as the key parameter in this regard.^23^ A well-established strategy to address this limitation is the integration of multiple photoswitches within a single molecule (Fig. 2).^25^ For example, norbornadiene with various linkers^26^ and connected azobenzenes^27,28^ have demonstrated excellent suitability as MOST systems. It has been demonstrated that both the connectivity pattern^28–30^ and the linking unit^26^ play a crucial role, influencing properties ranging from UV-vis absorption to the half-life of metastable isomers and the efficiency of photoconversion. In the case of oligo-azobenzenes, Wegner, Wachtveitl, Dreuw and coworkers demonstrated that phenylene-*meta*-connected azobenzene units are maximally decoupled and therefore retain the individual photochemistry of the subsystems.^28,31^ In hybrid MOST systems stepwise photoisomerizations, increased energy densities and a broader overlap with the solar spectrum have been achieved.^32^

Since dihydroazaborinines exhibit a certain degree of aromaticity,^33,34^ a second dihydroazaborinine unit can be used to extend the π-system, even if the two molecular switches are connected over one of the heteroatoms. Here, we report the synthesis and characterization of a series of 1,2-dihydro-1,2-azaborinines with extended π-systems. We chose ethynylene linkers between the boron heteroatom of the individual dihydroazaborinines and a central phenylene ring. The alkynylene linker, known for the high bond strength and planar geometry, improved the molecular planarity and aggregation in films thereby enabling precise control over morphology and phase separation in photoactive layers of organic solar cells.^35^ Containing two or more dihydroazaborinine units, our target molecules absorb in the UV-A range and in addition provide high storage densities. Studies addressing the versatile and complex interactions between the dihydroazaborinine building blocks of such molecules are not available. Our investigations provide an insight into the photoisomerization behavior of multichromophoric dihydroazaborinine systems.

## Results and discussion

### Synthesis

**Scheme 1:**
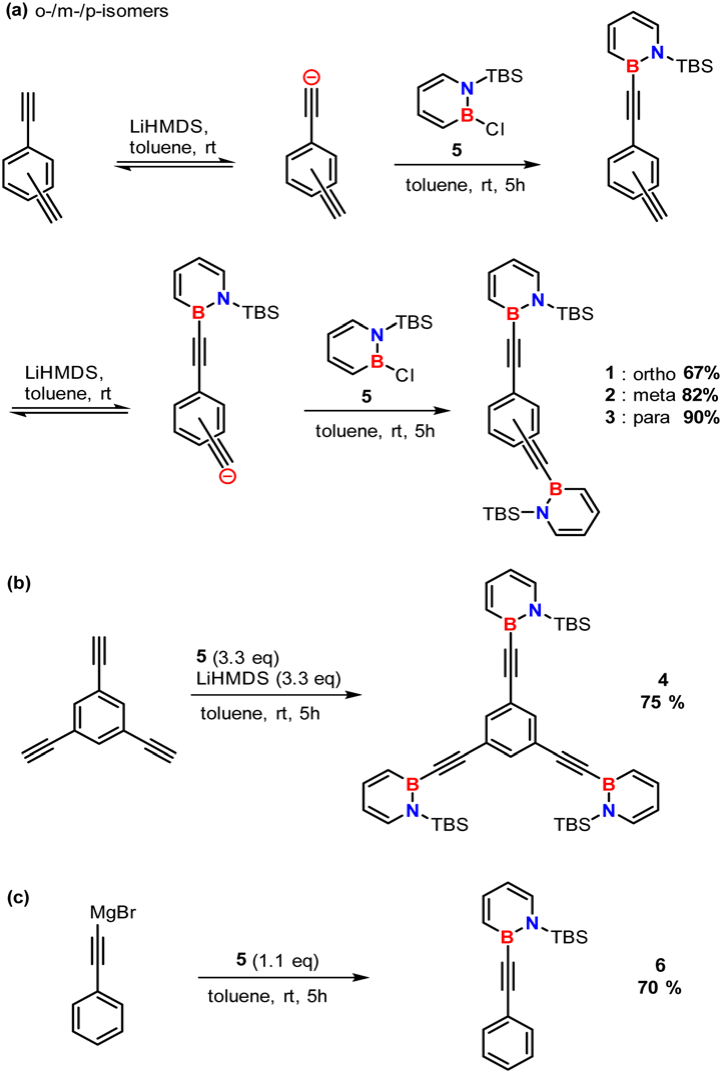
Synthesis of (a) the high energy density dihydroazaborinines 1–3, (b) the trifold substituted species 4, and (c) the reference compound 6 with one dihydroazaborinine unit.

The high-energy density dihydroazaborinines 1–4 can be prepared in a two-step one-pot procedure from the literature known 1-(*tert*-butyldimethylsilyl)-2-chloro-1,2-dihydro-1,2-azaborinine 5.^6,36^ Treatment of the *bis*-ethynylbenzene with lithiumhexamethyldisilazide furnishes the corresponding deprotonated species, which are highly nucleophilic and react with the electrophilic boron atom of dihydroazaborinine 5 (Scheme 1a). The triad 4 was synthesized using the same strategy but an adjusted quantity of base and compound 5. In case of 1-(*tert*-butyldimethylsilyl)-2-phenylethynyl-1,2-dihydro-1,2-azaborinine 6 instead of an organolithium reagent the commercially available *Grignard* reagent was used.

At room temperature, the neat compounds 1–2 are colorless oils, while the dihydroazaborinines 3–4 are colorless solids. All four species are stable at room temperature under dry atmospheric conditions.

### Solid state structure

**Fig. 3:**
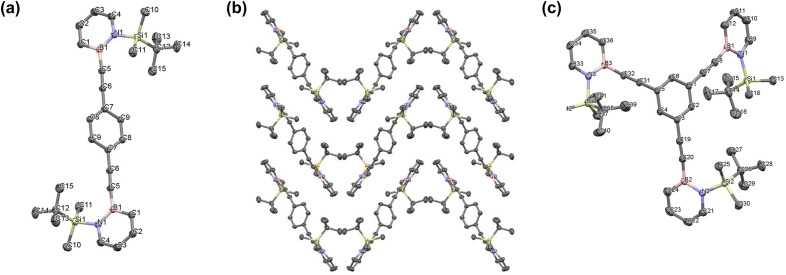
(a) Molecular structure of dihydroazaborinine 3 in the solid state (2253439), (b) packing of 3 in the solid state, (c) molecular structure of 4 in the solid state (2240655). Hydrogen atoms omitted for clarity. Thermal ellipsoids are drawn at the 50% probability level.

Single crystals of 3 and 4 suitable for X-ray crystallography were obtained by diffusion of dichloromethane vapor into *n*-hexane (Fig. 3). The dihydroazaborinine units of compound 3 are coplanar with a N1–B1–N1–B1 dihedral angle of exactly 180°. They are twisted with respect to the central benzene ring, by 48.1° (N1–B1–C7–C8) and −42.8° (N1–B1–C7–C8). A herringbone-like packing of the molecules in the crystal is observed, which points to intermolecular van der Waals interactions (Fig. 3). The dihydroazaborinine units of 4 are rotated with respect to each other, with dihedral angles of 55.1° (N1–B1–B2–N2), 37.0° (N1–B1–B3–N3) and −104.9° (N2–B2–B3–N3). In addition, the dihydroazaborinine units are non-coplanar with respect to the bridging benzene ring with dihedral angles of 5.8° (N3–B3–C5–C4), 15.4° (N1–B1–C1–C2) and 53.5° (N2–B2–C3–C2).

### UV-vis spectra

**Fig. 4:**
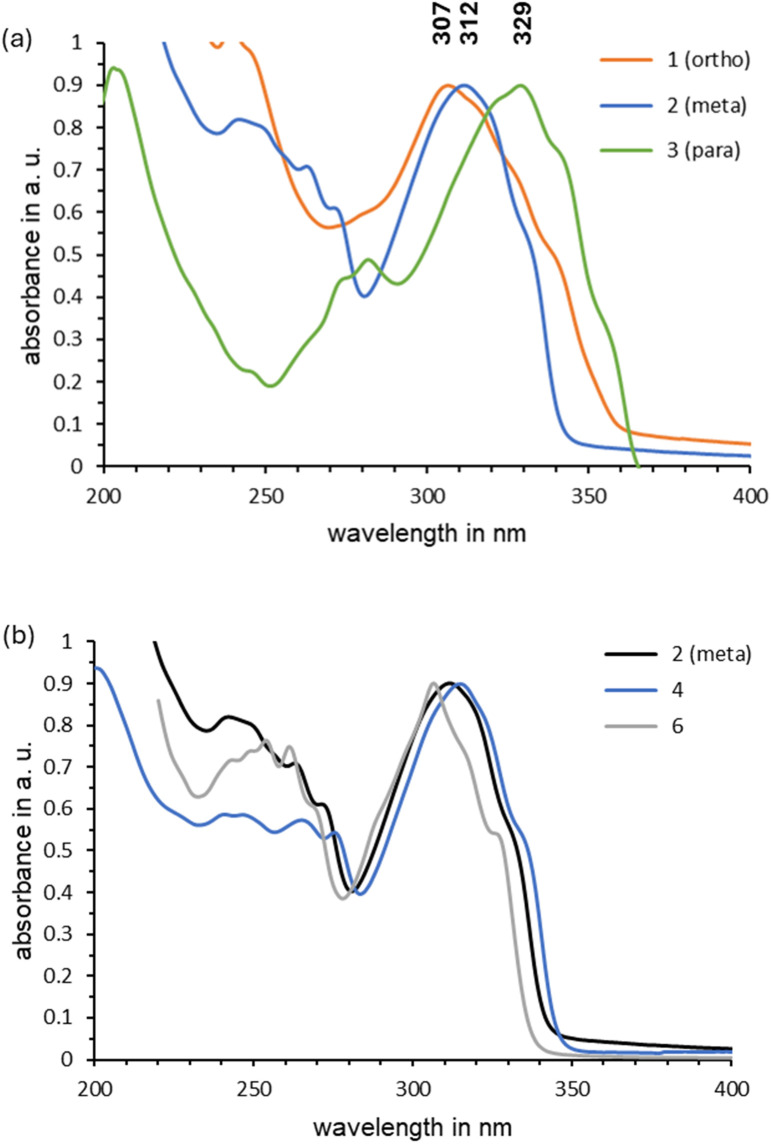
Comparison of the UV-vis absorption spectra in cyclohexane (0.005–0.01 M, RT) of (a) the high energy density isomers 1–3 and (b) the *meta*-aligned high energy density dihydroazaborinines 2 and 4.

All UV-vis spectra were measured in cyclohexane (Fig. 4), which provides a larger spectral window compared to other suitable solvents, *e.g.* benzene. Compared to reference compound 6, which contains only one dihydroazaborinine unit, a red-shift of the absorption maxima is observed for all compounds 1–4. This can be regarded to result from the enlarged π-system of the oligomers. An increasing bathochromic shift of the absorption onset can be found in the series *meta* (2) < *ortho* (1) < *para* (3). The longer wavelength onset of absorbance in the *para* system 3 can be rationalized by the linear conjugation pathway between the two photoswitching units (Fig. 4). In contrast, the absorption maximum of the *ortho* isomer 1 at 307 nm is almost identical to that of reference compound 6 (306 nm), which contains only one dihydroazaborinine unit, but shares the phenylethynyl substitution at boron with the oligomers. This can be explained by a poor orbital overlap between the dihydroazaborinine units of 1. With respect to the connectivity, also the UV-vis absorption band shape changes significantly for the three isomers 1–3.

The two *meta*-substituted species 2 and 4 have absorption spectra similar to that of 6. This indicates that there is no enhanced π extension over the second (or third) photo switching unit for the *meta* connected isomers 2 and 4. The para connected compound 3, on the other hand, has its absorption maximum bathochromically shifted to 329 nm and an onset of absorption at about 370 nm. With a bathochromic shift of 23 nm compared to species 6, the absorption of *para* isomer 3 lies in the UV-A range.

A comparable trend was reported earlier for absorption maxima of *ortho*, *meta* and *para* connected bis-azobenzenes.^28^ Moreover, detailed studies of the *cis*–*trans*-interconversion of azobenzene units depending on their connectivity revealed that the *meta*-bisazenes have largely decoupled photo switching units, while the *para* isomer showed the highest degree of π conjugation.^28^ Similarly, norbornadiene (NBD) oligomers reported by Moth-Poulsen and coworkers have larger differences of the absorption maxima between the *meta* and the *para* isomer.^29^

### Photoisomerization

**Fig. 5:**
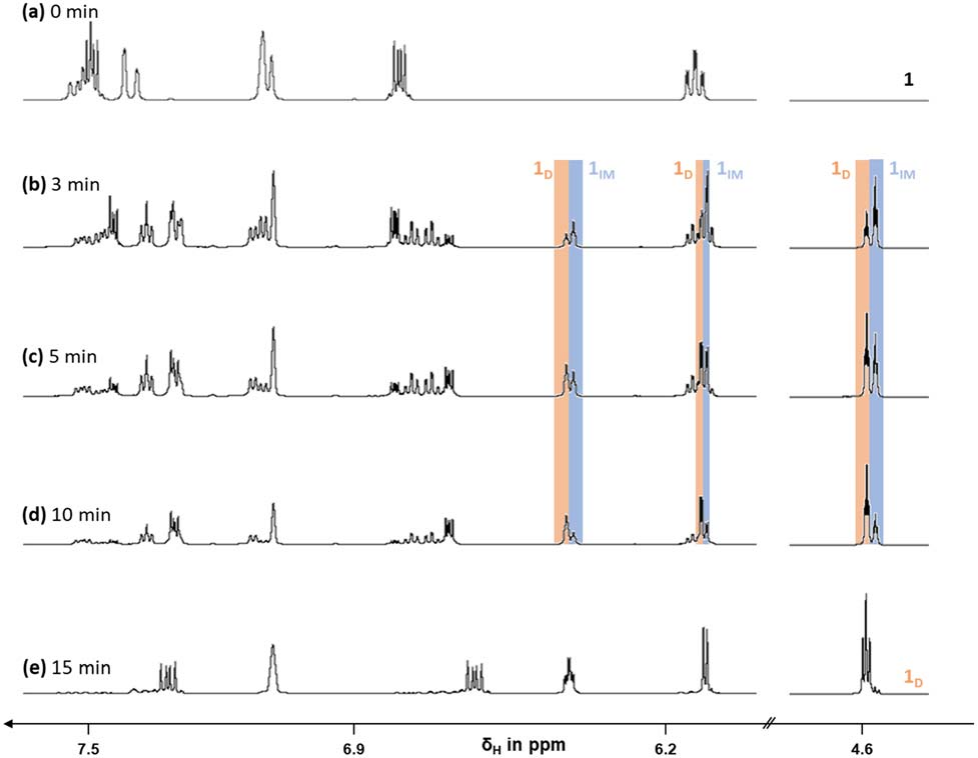
^1^H NMR spectra of dihydroazaborinine 1 upon irradiation (280–400 nm) after (a) 0 min, (b) 3 min, (c) 5 min, (d) 10 min and (e) 15 min reveal stepwise photoisomerization process. Only the aromatic region as well as one additional Dewar signal (4.6 ppm) are shown for clarity. Full spectra can be found in the ESI.†

While the photochemistry of the parent 1,2-dihydro-1,2-azaborinine was investigated under matrix isolation conditions (4 K, Ne matrix),^5^ the irradiation of 1,2-*bis*-substituted dihydroazaborinines like 1–4 can be conducted in 50 mM or 25 mM deuterated cyclohexane solutions at room temperature with 280–400 nm light using J.-Young quartz tubes.^3^ The reaction progress was monitored *via* proton NMR spectroscopy, and complete photoisomerization of all dihydroazaborinine units was observed. For the proximal *ortho* isomer 1 an exceptional insight into the mechanism of the isomerization was gained. Upon irradiation two NMR signals for each Dewar bridgehead proton arose and the signals for the dihydroazaborinine protons doubled as well (Fig. 5).

**Scheme 2:**
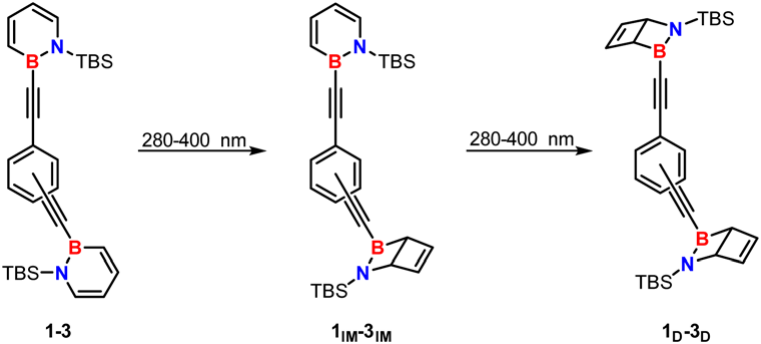
Stepwise photoisomerization process proposed based on the ^1^H NMR spectra of 1 upon irradiation (see Fig. 5).

After an extended irradiation time all dihydroazaborinine signals vanish due to complete isomerization. This is in accordance with disappearance of one set of Dewar proton signals (Fig. 5e), yielding the expected proton NMR spectrum for Dewar isomer 1_D_. These additional signals are in good agreement with an intermediate photoproduct 1_IM_, that combines two isomers of the dihydroazaborinine building block in one molecule (Scheme 2). A second photoisomerization process converts this species readily further to product 1_D_. Therefore, only in the beginning of the irradiation an excess of intermediate 1_IM_ can be noted (Fig. 5b).

The signals of the intermediate (IM) and the all-Dewar species (D) are less separated for the *meta* and *para*-substituted compounds 2 and 3. Bearing in mind the spatial distance of the dihydroazaborinine units of the isomers 2–3 (as well as in compound 4), very similar chemical shifts of the single Dewar fragments in 2_IM_–4_IM_ compared to the corresponding products 2_D_–4_D_ seem plausible. The same assertion goes for the dihydroazaborinine units of the intermediates 2_IM_–4_IM_ and the precursors 2–4. Nevertheless, the formation of similar intermediate species upon irradiation of 2–4 can be demonstrated *via*^1^H NMR spectroscopy considering the signals of the silyl substituents. The two methyl groups on silicon show a distinct change in the chemical shift in going from 2_IM_–4_IM_ to 2_D_–4_D_. For all compounds under investigation a conversion above 90% can be ascertained *via* NMR spectroscopy after 30 minutes of irradiation.

All Dewar isomers show partial decomposition upon removal of the solvent at room temperature, even under very mild conditions without any vacuum. The Dewar isomers 1_D_ and 2_D_ were obtained as orange–brown viscous oils, while 3_D_ and 4_D_ were isolated as amorphous brown solids. Further examination of the species formed upon drying the Dewar isomers 1_D_–4_D_ and 6_D_ was impossible due to insolubility. Attempts to purify or isolate the Dewar isomer from this mixture of unknown composition failed due to the high reactivity of this isomer, which e. g. does not allow for column chromatography. The NMR spectra after irradiation match excellently with the spectra of the Dewar isomer of dihydroazborinine A. The Dewar isomer of A was crystallized by Richter *et al.* as a complex with silver(i) hexafluoroantimonate, providing clear evidence of the Dewar isomer as the photoproduct.^10^ Based on the comparison of these NMR spectra, the formation of the Dewar isomer upon irradiation in solution can be clearly confirmed for the compounds 1–4 and 6.

### Thermal ring opening

The thermal ring opening of all four compounds was investigated using solutions. The solvent was never removed in between irradiation and heating. Due to the insufficient solubility of the dihydroazaborinines 2 and 3 in deuterated cyclohexane, all kinetic experiments were conducted in deuterated benzene. In order to obtain the activation barriers of the thermal cycloreversion, a 50 mM benzene solution of 1_D_–4_D_ was heated within the NMR spectrometer and the reaction progress was tracked using ^1^H NMR spectra. The cycloreversion of all dihydroazaborinines involves intermediates (IM) as was proven by means of ^1^H NMR spectroscopy.

For the isomers 1–3 the measurements proved a depletion of the all-Dewar species in agreement with first order kinetics. Accordingly, the time dependence of the decay can be described according to eqn (1).

1 [Dewar] = *A*_0_·e^−k_1_·*t*^

**Fig. 6:**
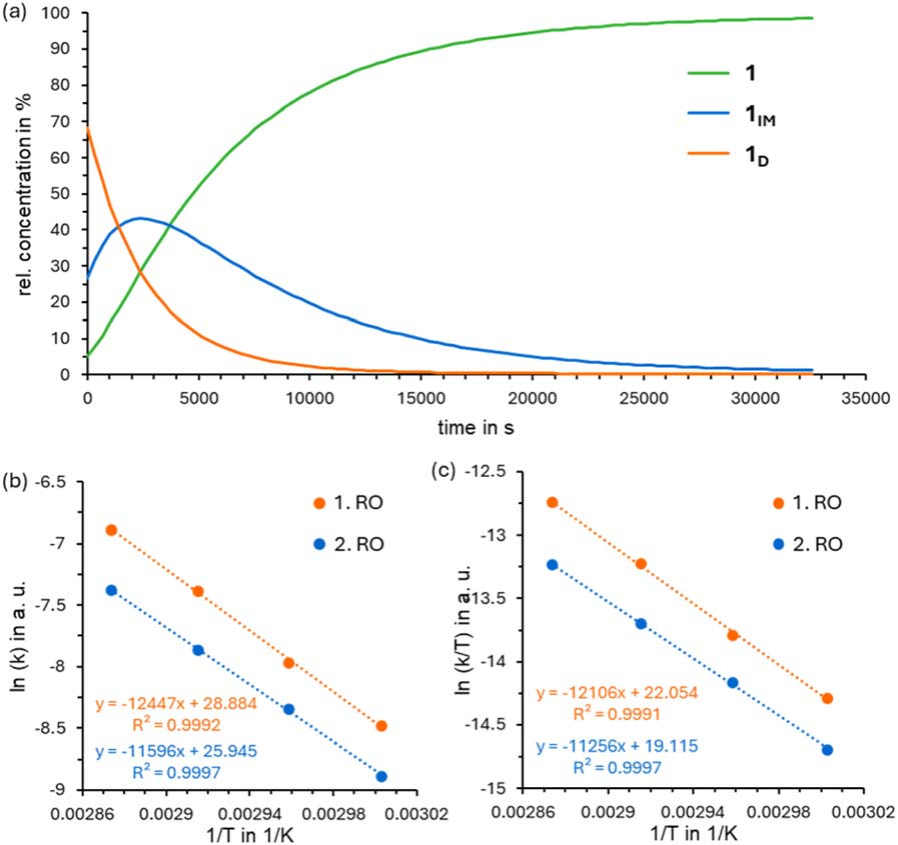
(a) Concentration change of dihydroazaborinine 1, its corresponding intermediate 1_IM_ and Dewar isomer 1_D_ upon constant heating at 338 K for 10 h*,* (b) Arrhenius plot resulting from separate measurements at 333 K, 338 K, 343 K and 348 K. (c) Eyring plot resulting from separate measurements at 333 K, 338 K, 343 K and 348 K. RO: ring opening.

After about 2.8 h 1_D_–3_D_ was completely converted into 1_IM_–3_IM_ (Fig. 6) and the decrease of the intermediate species can be observed nearly exclusively. This subsequent reaction could be identified to follow first order kinetics as well. Therefore, the reaction rate could be determined by employing eqn (2) of a follow-up reaction.2
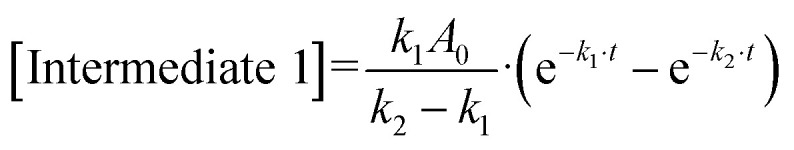


**Fig. 7:**
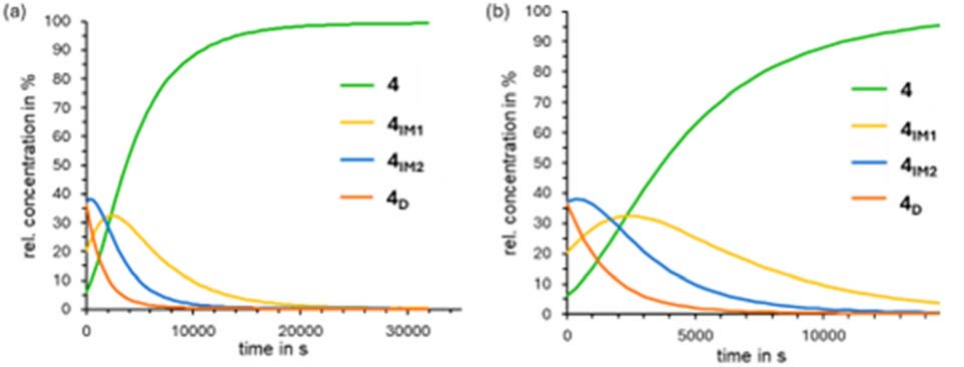
Concentration changes of dihydroazaborinine 4, its corresponding intermediates 4_IM1_ and 4_IM2_ and Dewar isomer 4_D_ upon constant heating to 338 K for 9 h (a) and concentration change in the first 4 h (b).

In case of the triply substituted dihydroazaborinine 4, two intermediate species appear, that provide separate signals in the ^1^H NMR spectrum. To our delight, a determination of all three activation barriers was possible. Again, the thermal ring opening of all-Dewar compound 4_D_ to 4_IM2_ follows first order kinetics. Both subsequent ring opening reactions were assumed to be first order kinetics, based on their behavior once the previous species or intermediate has mainly disappeared (after 1.3 h or 2.6 h respectively, Fig. 7). A derivation of the resulting time dependence (eqn (3)) can be found in the ESI† (section 5).3
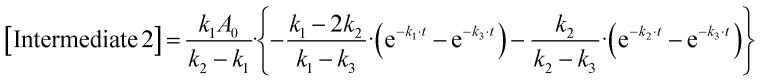


The results of the kinetic experiments and density functional computations (Table 1) for the thermal ring opening are in good agreement, which supports the above-mentioned conclusion of first order kinetics for the individual ring opening reactions.

**Table 1:** Comparison of calculated (B3LYP/6-311+G(d,p)) values and the experimentally determined activation barrier (Arrhenius) or Δ*G*^‡^ (Eyring) for the thermal ring opening (in kcal mol^−1^) as well as the half lifes (*t*_1/2_ in h) of the all-Dewar isomers at 298 K

		6	*ortho* (1)	*meta* (2)	*para* (3)	4
Arrhenius	*E*_a1,exp_	21.2	24.6	23.8	23.3	23.1
	*E*_a2,exp_	—	23.0	26.1	26.1	25.3
	*E*_a3,exp_	—	—	—	—	25.7
	*t*_1/2,1_	165	53	55	41	30
Eyring	Δ*G*^‡^_1.ro_	25.5	25.1	24.9	24.7	24.5
	Δ*G*^‡^_2.ro_	—	25.1	25.4	25.3	24.9
	Δ*G*^‡^_3.ro_	—	—	—	—	25.3
Theory	Δ*G*^‡^_1.ro_	—	23.9	—	25.1	—
	Δ*G*^‡^_2.ro_	—	23.8	23.5	25.5	—

The activation energies (*E*_a_) were determined from the parameters of Arrhenius plots using the Arrhenius equation. Activation enthalpies (Δ*H*^‡^), activation entropies (Δ*S*^‡^), and Gibbs free activation energies (Δ*G*^‡^) were calculated using the Eyring equation (for further details, see the ESI†). According to these activation barriers, the compounds allow for the storage of energy over several days at room temperature.

Since the same sample was used for the kinetic experiments described above, without any interim processing, the experiments provide insight into the stability over multiple isomerization cycles. To estimate the completeness of the conversion as well as potential decomposition, the normalized integrals of the kinetic measurements were utilized. During or after the NMR experiment no precipitation or cloudiness was observed, however, repeated irradiation and heating led to yellow to orange coloration of the solution. This could indicate traces of decomposition or a byproduct, which cannot be identified using NMR spectroscopy due to its low concentration. Qualitatively it is apparent that only minor decomposition is observed in four photoisomerization cycles (see ESI,† section 8).

The cycloreversion of 6 at room temperature can be triggered using silver(i) salts with weakly coordinating anions as Lewis acid catalyst, as reported earlier for 1-(*tert*-butyldimethylsilyl)-2-mesityl-1,2-dihydro-1,2-azaborinine A (see Fig. 1a) by Richter *et al.*^10^ Especially Ag[Al(OC(CF_3_)_3_)_4_]^37^ performed well (for additional information see ESI†). The catalysis can successfully be conducted in benzene, despite the silver ion suffering a reduction of its activity due to coordination by this solvent. Solvents like dichloromethane, used by Richter *et al.*,^10^ are unsuitable because they lead to decomposition of the Dewar isomers. The catalytic ring-opening of the isomers containing two Dewar units was studied using the *para*-isomer 3. The silver(i) salt also exhibits catalytic activity toward this isomer, however, the back reaction is accompanied by significant decomposition. In case of the triad, only decomposition is observed. The Wilkinson catalyst used by Edel *et al.* exclusively leads to the decomposition of the species 3, 4 and 6.^3^

### DSC measurement

To assess the energy stored in each isomer, differential scanning calorimetry (DSC) measurements were performed. To avoid decomposition of the Dewar isomers, mesitylene solutions were used for the DSC measurements. The compounds were irradiated in mesitylene solutions, and the solvent was never removed (for details see ESI†). Due to its poor solubility in the high boiling solvents under consideration, no experimental data could be recorded for isomer 3.

**Fig. 8:**
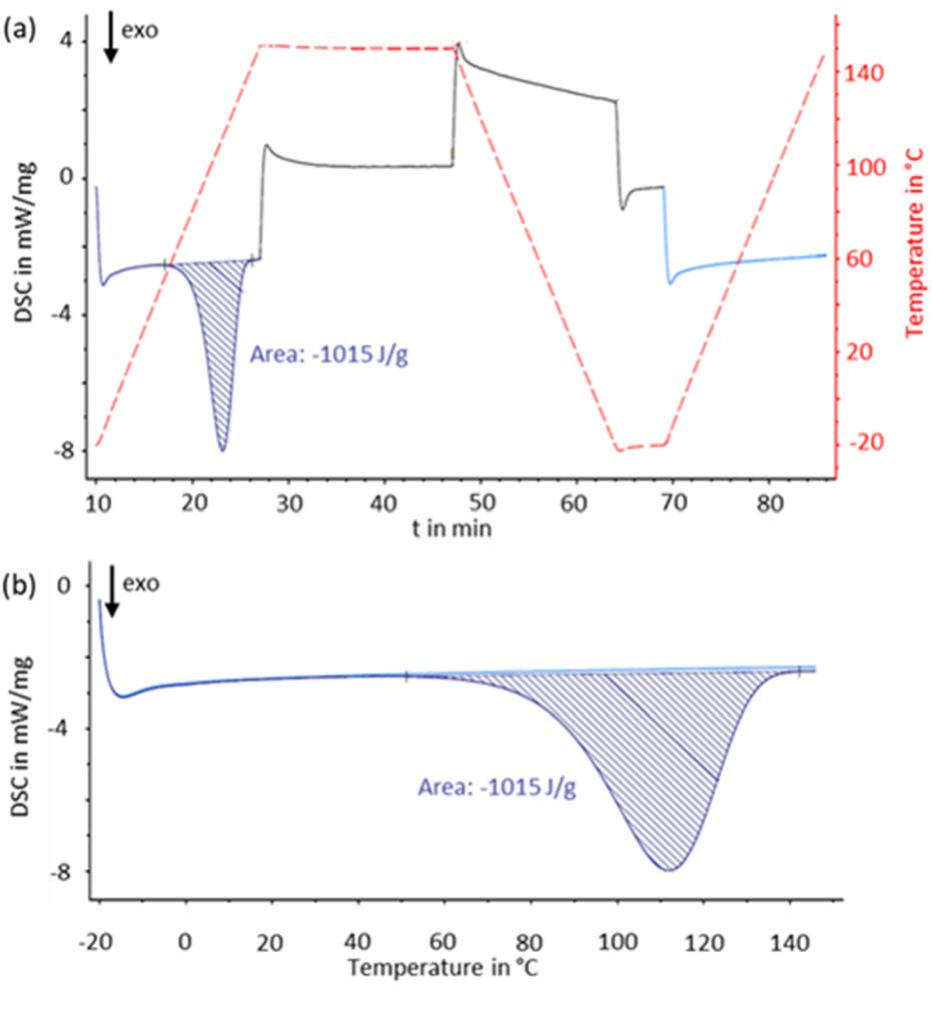
DSC measurement of compound 4 including both heating gradients as well as the intermediate cooling in the device (b) and direct comparison of the first (dark blue) and second (light blue) heating plotted against the temperature.

All photoproducts exhibited the characteristic DSC signal pattern of an exothermic process, which was assigned to the thermal back-conversion of the Dewar isomer (Fig. 8). During the first heating cycle, heat release is observed at 80 °C (1 and 4), 105 °C (2) or 65 °C (6). The completeness of energy release was verified by cooling the sample to −20 °C in the device and subsequently subject it to the same heating cycle again without any intermediate treatment (Fig. 8). No further exothermic signal was observed (see ESI† for further DSC data, section 8).

The resulting measurements show the expected additive behavior of the energy stored (Table 2). Oligomers containing two switching units store approximately twice the energy of a monomeric analog 6 (and A), and this added up to 157.1 kcal mol^−1^ for compound 4 with three units. Due to the additional dihydroazaborinine unit(s), the gravimetric energy density of the isomers and compound 4 remains largely constant compared to reference compound 6, despite the increased molar mass. For all five dihydroazaborinines the stored energy was also assessed by DFT computations. Large storage energies of about 50 kcal mol^−1^ per dihydroazaborinine unit were computed, which is in good agreement with previous calculations and reaction calorimetry experiments for monomeric dihydroazaborinine A.^3^

**Table 2:** Comparison of the experimental and computed (B3LYP/6-311+G(d,p)) values of the stored energy for the thermal ring opening of 1–5

	6	*ortho* (1)	*meta* (2)	*para* (3)	4
Δ*E*_exp_ (kJ kg^−1^)	741.2	794.4	873.5	—	994.4
Δ*E*_exp_ (kcal mol^−1^)	51.9	96.5	106.2	—	150.9
Δ*E*_theor_ (kcal mol^−1^)	48.2	109.3	109.4	109.8	157.1

### Comparative assessment

When directly compared to norbornadiene- and diazo-triad systems, compound 4 displays a significantly higher energy storage capacity (Table 3).^30,38^ As previously outlined, this advantage is accompanied by a trade-off in terms of absorption wavelength. Given that the excitation energy does not directly correlate with the stored energy but merely sets an upper limit, a bathochromic shift of 30 nm compared to the norbornadiene-based compound is still achieved.^30^ From an application-oriented perspective, the high energy density is compromised by the limited solubility of the investigated compounds 1–4.

**Table 3:** Comparison of triad 4 with comparable norbornadiene or diazo systems in terms of absorption wavelength, lifetime of the storage isomers and stored energy

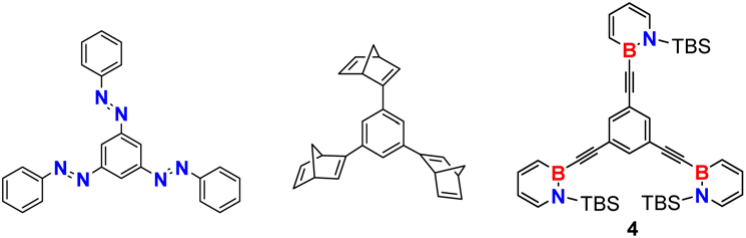			
*λ*_max_ (nm)	338/450	283	315
*t*_1/2_ (d)	5.6	8.0	1.3
Δ*E*_exp_ (kJ kg^−1^)	45.3	307.0	994.4
Δ*E*_exp_ (kcal mol^−1^)	10.8	61.1	150.9

Only in terms of half-life the triad 4 is outperformed by both the diazo and the norbornadiene systems. Yet, all three exhibit a comparable order of magnitude. For each of the compound classes represented by these three examples, systems with significantly longer half-lives have been reported.^3,39,40^

## Conclusion

In summary, we developed a synthesis of ethynylphenylene bridged high energy density dihydroazaborinines and investigated their photoisomerization behavior with respect to connectivity. The present systems provide a multimode photoswitch that is capable of sequentially switching between up to three states. The compounds can store high energies of up to 151 kcal mol^−1^ or almost 1 MJ kg^−1^ and provide storage times of multiple days at room temperature. If the dihydoazaborinine units are separated in a linearly conjugated path, as in case of *para* isomer 3, the chromophore is strongly altered, and the absorption maximum shows a distinct bathochromic shift. Even though none of the considered compounds absorb in the visible range, the overlap with the solar spectrum has been improved compared to previously published azaborinines^3^ and about 10% of the sunlight reaching the earth become usable. Due to the sufficiently high barrier for thermal ring opening and the considerable energy density, similar structural motifs could also be evaluated for the development of new MOST systems in the future. Potential improvements include the attachment of donor substituents to the central benzene ring to generate a further red shift. Given the high half-lives provided by sterically demanding groups at the boron atom,^3^ substituting the central ring with sterically demanding groups and the direct linkage of the dihydroazaborinine to the phenylene unit are also conceivable. Recent theoretical investigations further demonstrated that substitution at the carbon positions *para* to the heteroatoms exerts a significant impact on the absorption wavelength.^41^ Current efforts in our laboratory focus on implementing such strategies for red-shifting the absorption spectrum, scaling up synthetic methodologies, and enhancing the stability of the Dewar isomers. We anticipate that these ongoing studies will enable an evidence-based assessment of the technological implementation of dihydroazaborinines in the MOST context.

## Author contributions

H. F. Bettinger and S. M. Biebl conceptualized the study. S. M. Biebl performed the syntheses, crystallizations, spectroscopy, kinetic analysis, DSC measurements and DFT computations and wrote the manuscript together with H. F. Bettinger. M. Ströbele measured and solved the single crystal structures. R. C. Richter performed the synthesis of the catalysts and investigated the catalytic back reaction. I. Fleischer acquired funding. H. F. Bettinger acquired funding and performed initial DFT calculations on the thermal ring opening of the *para* isomer.

## Conflicts of interest

The authors declare no conflict of interest.

## Supplementary Material

SC-016-D5SC03159A-s001

SC-016-D5SC03159A-s002

## Data Availability

The data underlying this study are available in the published article and its ESI.†

## References

[cit1] Moth-Poulsen K. , in Organic Synthesis and Molecular Engineering, Wiley Inc., 2013, pp. 179–196

[cit2] Sun C.-L., Wang C., Boulatov R. (2019). ChemPhotoChem.

[cit3] Edel K., Yang X., Ishibashi J. S. A., Lamm A. N., Maichle-Mössmer C., Giustra Z. X., Liu S.-Y., Bettinger H. F. (2018). Angew. Chem., Int. Ed..

[cit4] Bettinger H. F., Hauler O. (2013). Beilstein J. Org. Chem..

[cit5] Brough S. A., Lamm A. N., Liu S.-Y., Bettinger H. F. (2012). Angew. Chem., Int. Ed..

[cit6] Marwitz A. J. V., Matus M. H., Zakharov L. N., Dixon D. A., Liu S.-Y. (2009). Angew. Chem., Int. Ed..

[cit7] Campbell P. G., Marwitz A. J. V., Liu S.-Y. (2012). Angew. Chem., Int. Ed..

[cit8] Giustra Z. X., Liu S.-Y. (2018). J. Am. Chem. Soc..

[cit9] Ozaki T., Bentley S. K., Rybansky N., Li B., Liu S.-Y. (2024). J. Am. Chem. Soc..

[cit10] Richter R. C., Biebl S. M., Einholz R., Walz J., Maichle-Mössmer C., Ströbele M., Bettinger H. F., Fleischer I. (2024). Angew. Chem., Int. Ed..

[cit11] Börjesson K., Lennartson A., Moth-Poulsen K. (2013). ACS Sustain. Chem. Eng..

[cit12] Gimenez-Gomez A., Magson L., Peñin B., Sanosa N., Soilán J., Losantos R., Sampedro D. (2022). Photochem.

[cit13] Bren V. A., Alexander D. D., Vladimir I. M., Chernoivanov V. A. (1991). Russ. Chem. Rev..

[cit14] Chakraborty S., Nguyen H. P. Q., Usuba J., Choi J. Y., Sun Z., Raju C., Sigelmann G., Qiu Q., Cho S., Tenney S. M., Shulenberger K. E., Schmidt-Rohr K., Park J., Han G. G. D. (2024). Chem.

[cit15] Wang Z., Roffey A., Losantos R., Lennartson A., Jevric M., Petersen A. U., Quant M., Dreos A., Wen X., Sampedro D., Börjesson K., Moth-Poulsen K. (2019). Energy Environ. Sci..

[cit16] Franz E., Kunz A., Oberhof N., Heindl A. H., Bertram M., Fusek L., Taccardi N., Wasserscheid P., Dreuw A., Wegner H. A., Brummel O., Libuda J. (2022). ChemSusChem.

[cit17] Wang Z., Erhart P., Li T., Zhang Z.-Y., Sampedro D., Hu Z., Wegner H. A., Brummel O., Libuda J., Nielsen M. B., Moth-Poulsen K. (2021). Joule.

[cit18] Broman S. L., Jevric M., Bond A. D., Nielsen M. B. (2014). J. Org. Chem..

[cit19] Kanai Y., Srinivasan V., Meier S. K., Vollhardt K. P. C., Grossman J. C. (2010). Angew. Chem., Int. Ed..

[cit20] Maag H., Schmitz M., Sandvoß A., Mundil D., Pedada A., Glaser F., Kerzig C., Wahl J. M. (2024). J. Am. Chem. Soc..

[cit21] Wang Z., Hölzel H., Moth-Poulsen K. (2022). Chem. Soc. Rev..

[cit22] Jevric M., Petersen A. U., Mansø M., Kumar Singh S., Wang Z., Dreos A., Sumby C., Nielsen M. B., Börjesson K., Erhart P., Moth-Poulsen K. (2018). Chem.–Eur. J..

[cit23] Kuisma M. J., Lundin A. M., Moth-Poulsen K., Hyldgaard P., Erhart P. (2016). J. Phys. Chem. C.

[cit24] Lennartson A., Roffey A., Moth-Poulsen K. (2015). Tetrahedron Lett..

[cit25] Salthouse R. J., Moth-Poulsen K. (2024). J. Mater. Chem. A.

[cit26] Mansø M., Tebikachew B. E., Moth-Poulsen K., Nielsen M. B. (2018). Org. Biomol. Chem..

[cit27] Sun S., Liang S., Xu W.-C., Wang M., Gao J., Zhang Q., Wu S. (2022). Soft Matter.

[cit28] Slavov C., Yang C., Schweighauser L., Boumrifak C., Dreuw A., Wegner H. A., Wachtveitl J. (2016). Phys. Chem. Chem. Phys..

[cit29] Mansø M., Petersen A. U., Wang Z., Erhart P., Nielsen M. B., Moth-Poulsen K. (2018). Nat. Commun..

[cit30] Schulte R., Afflerbach S., Paululat T., Ihmels H. (2023). Angew. Chem., Int. Ed..

[cit31] Yang C., Slavov C., Wegner H. A., Wachtveitl J., Dreuw A. (2018). Chem. Sci..

[cit32] Kilde M. D., Mansø M., Ree N., Petersen A. U., Moth-Poulsen K., Mikkelsen K. V., Nielsen M. B. (2019). Org. Biomol. Chem..

[cit33] Campbell P. G., Abbey E. R., Neiner D., Grant D. J., Dixon D. A., Liu S.-Y. (2010). J. Am. Chem. Soc..

[cit34] Abbey E. R., Zakharov L. N., Liu S.-Y. (2008). J. Am. Chem. Soc..

[cit35] Yang F., Fang H., Guo E., Xiao C., Lu Z., Wang Y., Fan H., Zhang A., Lai W., Li W. (2025). Angew. Chem., Int. Ed..

[cit36] Marwitz A. J. V., Abbey E. R., Jenkins J. T., Zakharov L. N., Liu S.-Y. (2007). Org. Lett..

[cit37] Krossing I. (2001). Chem.–Eur. J..

[cit38] Dong L., Chen Y., Zhai F., Tang L., Gao W., Tang J., Feng Y., Feng W. (2020). J. Mater. Chem. A.

[cit39] Orrego-Hernández J., Dreos A., Moth-Poulsen K. (2020). Acc. Chem. Res..

[cit40] Adrion D. M., Lopez S. A. (2023). Org. Biomol. Chem..

[cit41] Müller A. J., Markhart J., Bettinger H. F., Dreuw A. (2025). Chem. Commun..

