# Supplementary material for: High energy density dihydroazaborinine dyads and triad for molecular solar thermal energy storage
Source: Chem Sci. 2025 Jul 25;16(33):15231–8. doi: 10.1039/d5sc03159a (PMC12291973; doi:10.1039/d5sc03159a)
Supplement: SC-016-D5SC03159A-s001 [file SC-016-D5SC03159A-s001.pdf]

Supplementary Information (SI) for Chemical Science.
This journal is © The Royal Society of Chemistry 2025

Electronic Supporting Information

Achieving high energy density in MOST systems with dihydroazaborinine oligomers

Sonja M. Biebl, Robert C. Richter, Markus Ströbele, Ivana Fleischer,* Holger F. Bettinger*

Institut für Organische Chemie, Auf der Morgenstelle 18, 72076 Tübingen, Germany

* ivana.fleischer@uni-tuebingen.de

* holger.bettinger@uni-tuebingen.de

Table of Contents

1. Methods 3

2. Analytics 3

3. Synthesis 5

Bis-(2-Ethinyl-1-tert-butyldimethylsilyl)-1,2-dihydro-1,2-azaborinin)benzene isomers 1-3 5

1,2-bis-(2-Ethinyl-1-tert-butyldimethylsilyl)-1,2-dihydro-1,2azaborinin)benzene 1 6

1,3-bis-(2-Ethinyl-1-tert-butyldimethylsilyl)-1,2-dihydro-1,2-azaborinin)benzene 2 8

1,4-bis-(2-Ethinyl-1-tert-butyldimethylsilyl)-1,2-dihydro-1,2-azaborinin)benzene 3 10

1,3,5-Tris-(2-Ethinyl-1-tert-butyldimethylsilyl)-1,2-dihydro-1,2-azaborinin)benzene 4 12

1-(Tert-butyldimethylsilyl)-2-(phenylethynyl)-1,2-dihydro-1,2-azaborinine 5 15

4. Irradiation experiments 16

NMR data of 1_{Dewar} 17

NMR data of 2_{Dewar} 19

NMR data of 3_{Dewar} 21

NMR data of 4_{Dewar} 23

NMR data of 6_{Dewar} 25

5. Kinetic experiments 27

Arrhenius treatment of the measured data 28

Eyring treatment of the data 30

Derivation of the time dependence of the concentrations 32

Concentrations profiles 35

6. Catalytic ring opening 36

Catalytic back conversion of 6 36

Catalytic back conversion of 3 37

7. Cyclability 38

8. DSC measurements 40

9. UV-Vis spectra 51

10. Crystal structures 52

11. Computations 58

Rotamers 122

References 123

1. Methods

Unless otherwise noted, all experiments were carried out under inert conditions using Schlenk technique with argon or nitrogen as the protective gas or in a glove box (UNIlab Pro, MBraun). Glassware was dried before use by heating. Commercial triethylamine (water content ~ 0.2%) was refluxed over KOH for two hours and then distilled before use. The remaining chemicals used were employed as received from the manufacturer without further purification. Anhydrous solvents were obtained from Thermo Fisher Scientific Inc., Acros Organics B.V.B.A., or Sigma-Aldrich, or, in the case of dichloromethane, diethyl ether, n-hexane, tetrahydrofuran, and toluene, from an SPS-800 solvent drying system by the manufacturer MBraun.

2. Analytics

NMR Spectroscopy: The acquisition of ^{1}H, ^{13}C-{^{1}H}, and ^{11}B-{^{1}H} NMR spectra was performed on a Bruker Advance III HD 400 MHz instrument (^{1}H spectra) at 101 MHz (^{13}C-{^{1}H} spectra), and 128 MHz (^{11}B-{1H} spectra). Complementarily, spectra of these nuclei were recorded on a Bruker Advance III HD 300 MHz NanoBay at 300 MHz (^{1}H spectra), 76 MHz (^{13}C-{^{1}H} spectra), and 96 MHz (^{11}B-{^{1}H} spectra). The measurement of ^{119}Sn spectra of the presented stannanes was exclusively carried out using the Bruker Advance III HD 300 MHz NanoBay at 112 MHz (Bruker Corporation). For high-temperature NMR measurements (kinetic experiments), a Bruker Avance III HDX 600 spectrometer was employed with a measuring frequency of 600 MHz (^{1}H spectra) or 192 MHz (^{11}B-{^{1}H} spectra). All obtained NMR spectra were referenced to the solvent peak. Deuterated dichloromethane (5.32 ppm), benzene (7.16 ppm), chloroform (7.26 ppm), cyclohexane (1.38 ppm), or tetrachloroethane (5.91 ppm) from Sigma-Aldrich or Deutero were used for referencing.

MPLC Chromatography: Column chromatographic purifications were performed using the puriFlash 430 in combination with pre-packed aluminium oxide gel columns (particle size: 20 µm) from Interchim. Detection was carried out using a puriFlash One Series UV detector (DAD 200-600 nm) from the same manufacturer.

All solvents used were of HPLC-grade purity. Solvent mixtures are indicated as V/V ratio.

GPC Chromatography: The size exclusion chromatography was conducted on a Recycling Preparative HPLC and GPC (LaboAC LC-7080 Plus II) device and a JAIGEL-2.5 HR Plus column both from Japan Analytical Industries Co.

UV/VIS Spectroscopy: For determining the absorption maxima of the presented 1,2-substituted 1,2-dihydro-1,2-azaborinines, a Lambda 1050 UV/VIS/NIR spectrometer (Perkin Elmer) with a 3D WB detection

module was used, operated with the accompanying UV Win Lab software (Version 2.0.2). The wavelength range covered was from 250 nm to 850 nm. All solvents used had a cut-off wavelength below 250 nm and were water-free.

Irradiation experiments: As light source an Osram HBO-500-W/2 high pressure mercury lamp in an Oriel housing with quartz optics and a dichroic mirror (280 – 400 nm) was applied. No additional filters were used.

The samples for the kinetic studies were prepared in quartz glass J.-Young-NMR tubes as the reaction vessel.

X-Ray Crystallography: Crystals suiTable S for X-Ray diffraction were grown by vapour diffusion with *n*-hexane and dichloromethane at room temperature. Single crystals were selected, coated with Parabar 10312 and fixed on a microloop.

Data were collected on a XtaLAB Synergy, Dualflex, HyPix diffractometer using *w* scans with Cu K_a radiation. The crystal was kept at a steady temperature during data collection. The diffraction pattern was indexed and the total number of runs and images was based on the strategy calculation from the program CrysAlisPro 1.171.42.49, which was also used for refining the unit cell. The structure was solved with the **ShelXT** 2018/2 solution program[1-3] using dual methods and by using **Olex2** 1.5-ac5-024 as the graphical interface.[4] The model was refined with **olex2.refine** 1.5-ac5-024 using full matrix least squares minimisation on $\boldsymbol{F}^2$.[5]

Mass spectrometry: High resolution mass spectra were recorded on a HR-ESI/APCI-TOF device (maXis 4G, Bruker). The sample was dissolved in dichloromethane and injection was carried out using a syringe pump.

3. Synthesis

Precursor **5** was synthesized according to Richter *et al.*[6] The chemical shift assignments of all new compounds were performed based on 2D NMR spectroscopy measurements.

Bis-(2-Ethinyl-1-tert-butyldimethylsilyl)-1,2-dihydro-1,2-azaborinin)benzene isomers 1-3

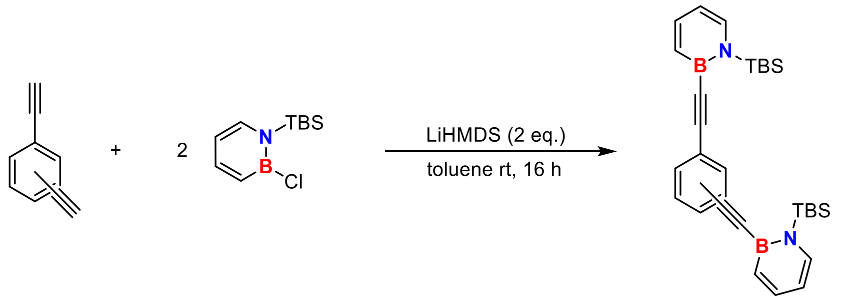

In a glovebox a solution of precursor dihydoazaborin **5** (0.2 g, 0.88 mmol, 2.5 eq.) was dissolved in 1 mL toluene. In a separate vessel the corresponding *bis*-ethynylbenzene derivative (0.04 g, 0.35 mmol, 1 eq.) was dissolved in 2 mL toluene and a solution of LiHMDS (1 M in hexane, 0.88 mL, 0.88 mmol, 2.5 eq.) was slowly added. The resulting suspension was treated with the previously prepared dihydroazaborinine solution and stirred for 16 h at room temperature. The reaction mixture was transferred into 5 mL of distilled water, and the organic phase is expanded by 2 mL of *n*-hexane. After the separation of the organic phase the aqueous Phase was extracted three times with 5 mL of *n*-hexene. The collected organic layers are washed with 25 mL of brine, dried over magnesium sulfate, filtered and the solvent was removed *in vacuo*. The crude product was purified by column chromatography (neutral aluminium oxide, *n*-hexane/dichloromethane gradient from 100/0 to 85/15) followed by GPC size exclusion chromatography (*n*-hexane/dichloromethane 75/25). The products were obtained as colorless oils in case of compound **1** (83%) and **2** (97%) or as a colorless solid in case of dihydroazaborinine **3** (80%).

For the assignment of the NMR signals of the subsequent azaborinines and their Dewar isomers, the numbering provided below was employed.

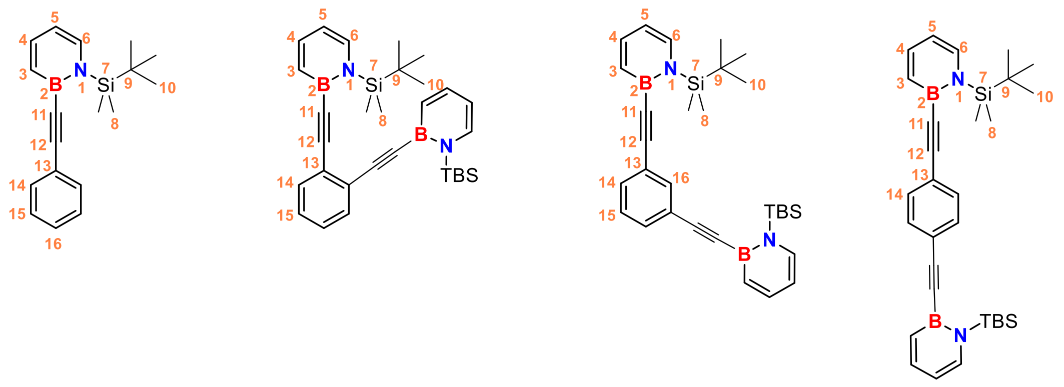

1,2-bis-(2-Ethinyl-1-tert-butyldimethylsilyl)-1,2-dihydro-1,2azaborinin)benzene 1

^{1}H-NMR (600 MHz, **C_6D_{12}**): δ = 7.55 (dd, $^3J_{HH}$ = 6.39 Hz, 2H, H-4), 7.53 (m, 2H, H-14), 7.45 (dm, $^3J_{HH}$ = 10.94 Hz, 2H, H-6), 7.14 (d, $^3J_{HH}$ = 6.72 Hz, 2H, H-3), 6.86 (m, 2H, H-15), 6.23 (dt, $^3J_{HH}$ = 6.39 Hz, $^4J_{HH}$ = 1.45 Hz, 2H, H-5), 0.92 (s, 18H, H-10), 0.49 (s, 12H, H-8) ppm.

^{13}C-{^{1}H}-NMR (600 MHz, **C_6D_{12}**): δ = 143.9 (C4), 138.8 (C6), 134.9 (br., C3), 132.6 (C14), 128.7 (C13), 127.4 (C15), 113.1 (C5), 107.4 (C12), 103.6 (br., C11) ppm.

^{11}B-{^{1}H}-NMR (600 MHz, **C_6D_{12}**): δ = 29.9 ppm.

HR-MS (ESI): m/z calc. for $[M+Na]^+$ 531.29648, found 531.29762.

$C_{30}H_{42}B_2N_2NaSi_2$

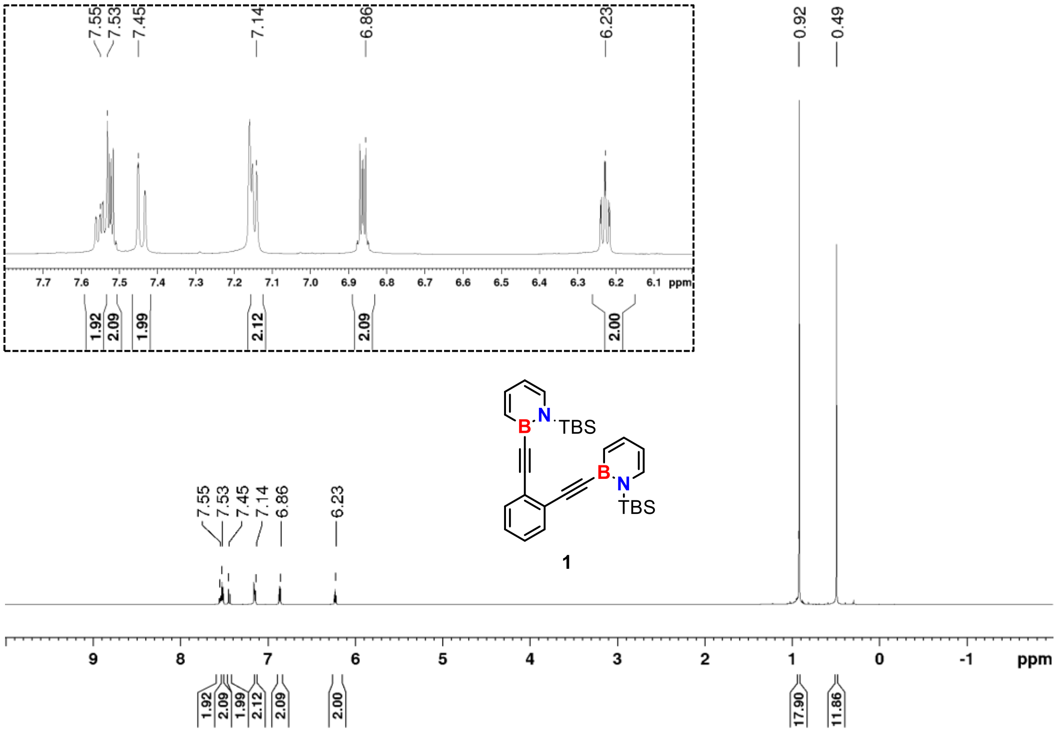

Figure S1. ^{1}H-NMR spectrum of **1** measured in benzene-d_6. The enlarged section shows the region between 6 and 7.8 ppm for a better visibility of the aromatic signals.

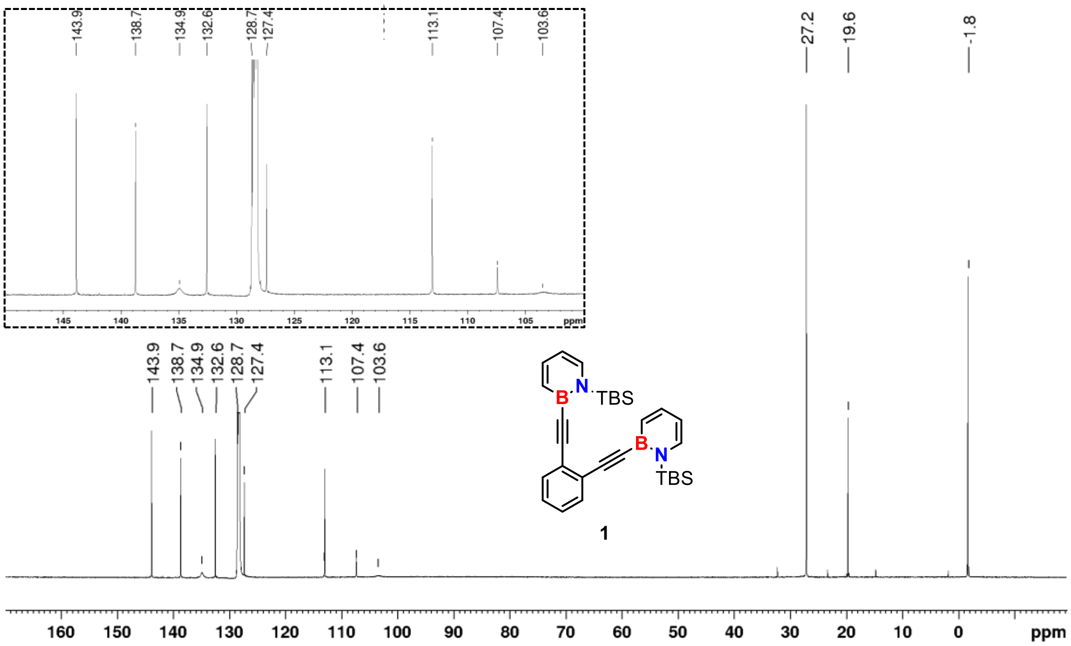

Figure S2. ^{1}H-{^{13}C}—NMR spectrum of **1** measured in benzene-d$_6$. The enlarged section shows the region between 150 and 100 ppm for a better visibility of the aromatic signals.

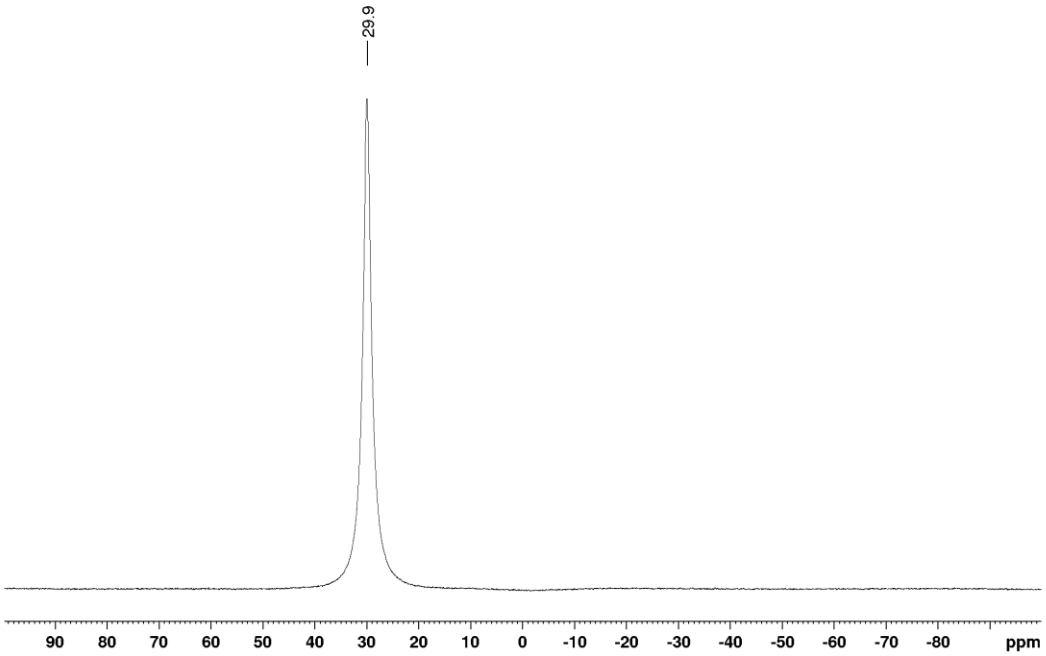

Figure S3. ^{11}B-NMR spectrum of **1** measured in benzene-d6.

1,3-bis-(2-Ethinyl-1-tert-butyldimethylsilyl)-1,2-dihydro-1,2-azaborinin)benzene 2

^{1}H-NMR (600 MHz, **C_6D_{12}**): δ = 7.99 (m, 1H, H-16), 7.56 (dd, 2H, H-4), 7.41 (dd, 2H, H-14), 7.36 (d, 2H, H-3), 7.15 (d, 2H, H-3), 6.86 (t, 1H, H-15), 6.25 (dt, 2H, H-5), 0.92 (s, 18H, H-10), 0.45 (s, 12H, H-8) ppm.

^{13}C-{^{1}H}-NMR (600 MHz, **C_6D_{12}**): δ = 144.2 (C4), 138.9 (C6), 134.7 (br., C3), 134.5 (C16), 131.7 (C15), 129.3 (C14), 125.7 (C13), 113.2 (C5), 107.4 (C12), 100.0 (br., C11), 27.1 (C10), 19.6 (C9), -1.9 (C8) ppm.

^{11}B-{^{1}H}-NMR (600 MHz, **C_6D_{12}**): δ = 29.7 ppm.

HR-MS (APCI): m/z calc. for $[M+H]^+$ 509.31454, found 509.31568.

$C_{30}H_{43}B_2N_2Si_2$

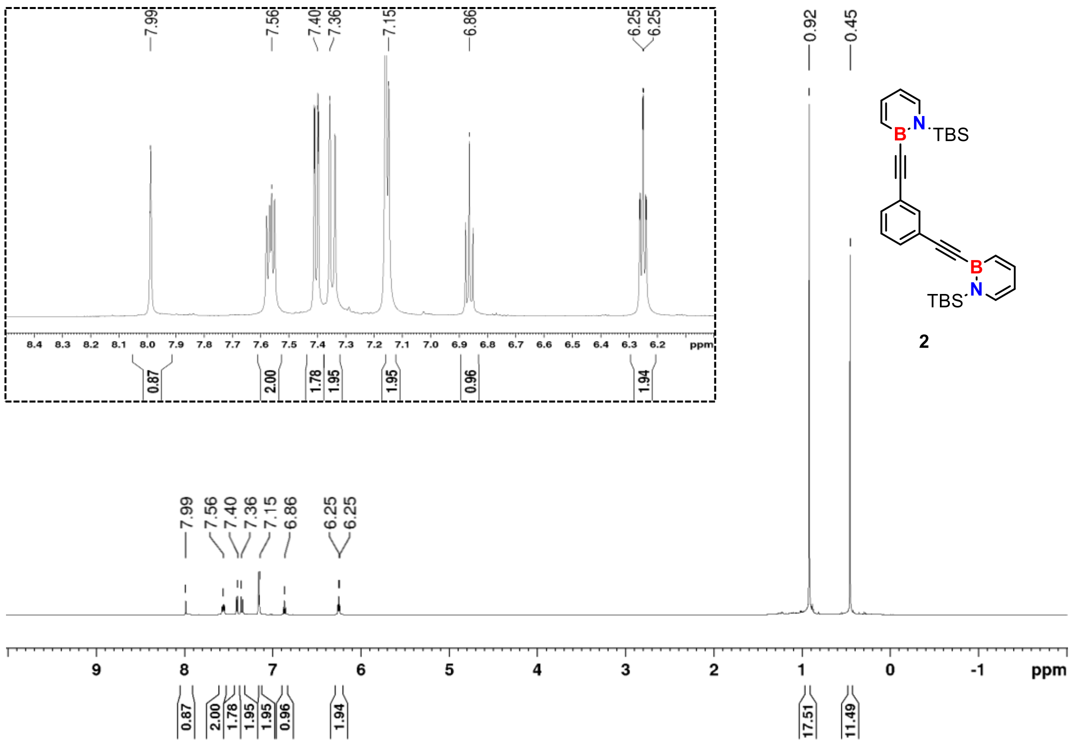

Figure S4. ^{1}H-NMR spectrum of **2** measured in benzene-d_6. The enlarged section shows the region between 6 and 8.5 ppm for a better visibility of the aromatic signals.

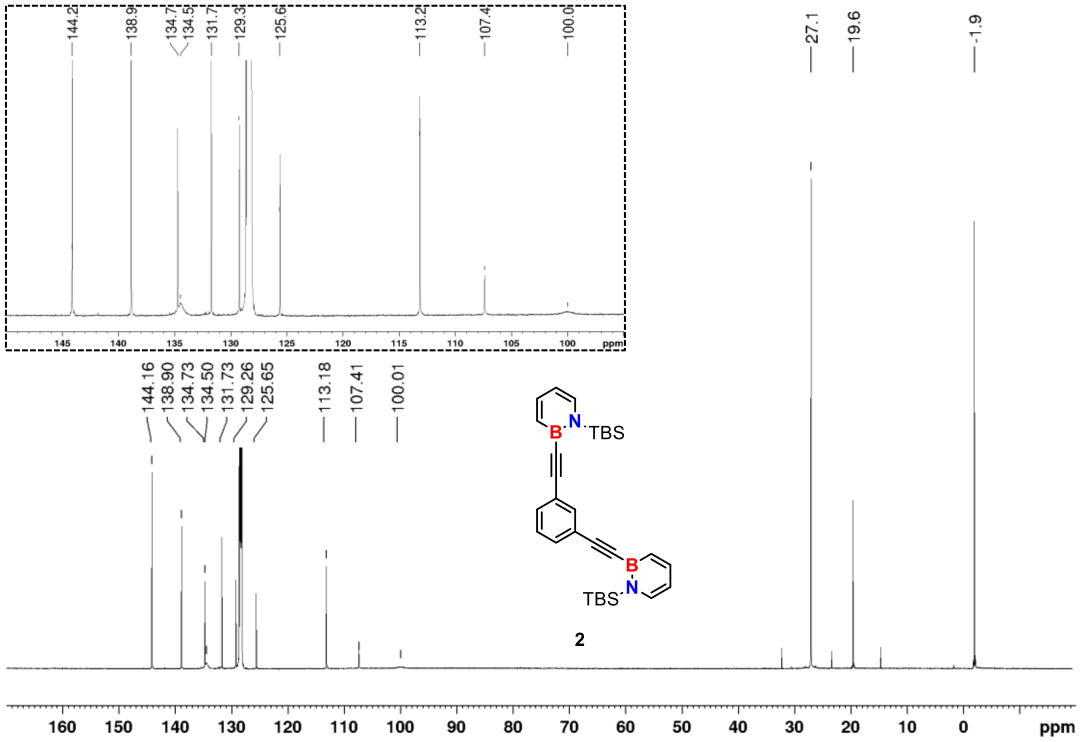

Figure S5. ^{1}H-{^{13}C}—NMR spectrum of **2** measured in benzene-d$_6$. The enlarged section shows the region between 150 and 100 ppm for a better visibility of the aromatic signals.

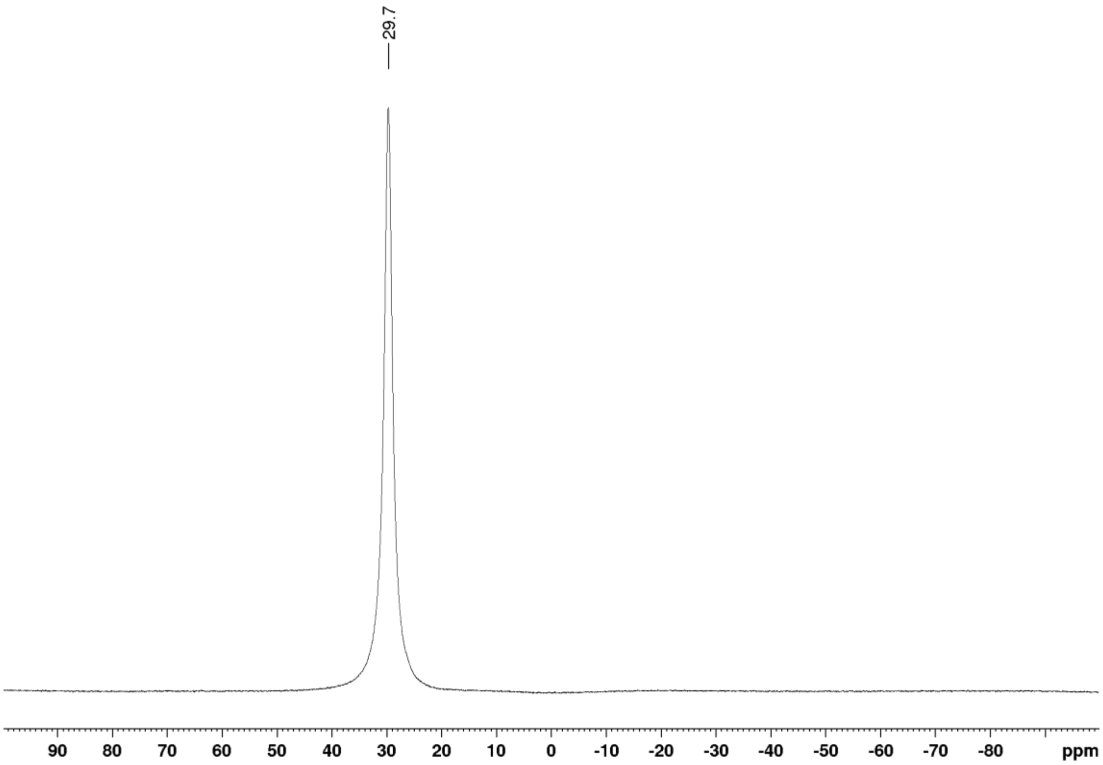

Figure S6. ^{11}B-NMR spectrum of **2** measured in benzene-d6.

1,4-bis-(2-Ethinyl-1-tert-butyldimethylsilyl)-1,2-dihydro-1,2-azaborinin)benzene 3

^{1}H-NMR (600 MHz, **C_6D_{12}**): δ = 7.57 (dd, 2H, H-4), 7.42 (s, 4H, H-14), 7-37 (d, 2H, H-3), 7.17 (d, 2H, H-3), 6.26 (dt, 2H, H-5), 0.92 (s, 18H, H-10), 0.45 (s, 12H, H-8) ppm.

^{13}C-{^{1}H}-NMR (600 MHz, **C_6D_{12}**): δ = 144.2 (C4), 138.9 (C6), 134.4 (br., C3), 132.1 (C14), 124.9 (C13), 113.2 (C5), 108.0 (C12), 101.3 (br., C11), 27.1 (C10), 19.6 (C9), -1.9 (C8) ppm.

^{11}B-{^{1}H}-NMR (600 MHz, **C_6D_{12}**): δ = 29.7 ppm.

HR-MS (APCI): m/z calc. for $[M+H]^+$ 509.31454, found 509.31568.

$C_{30}H_{43}B_2N_2Si_2$

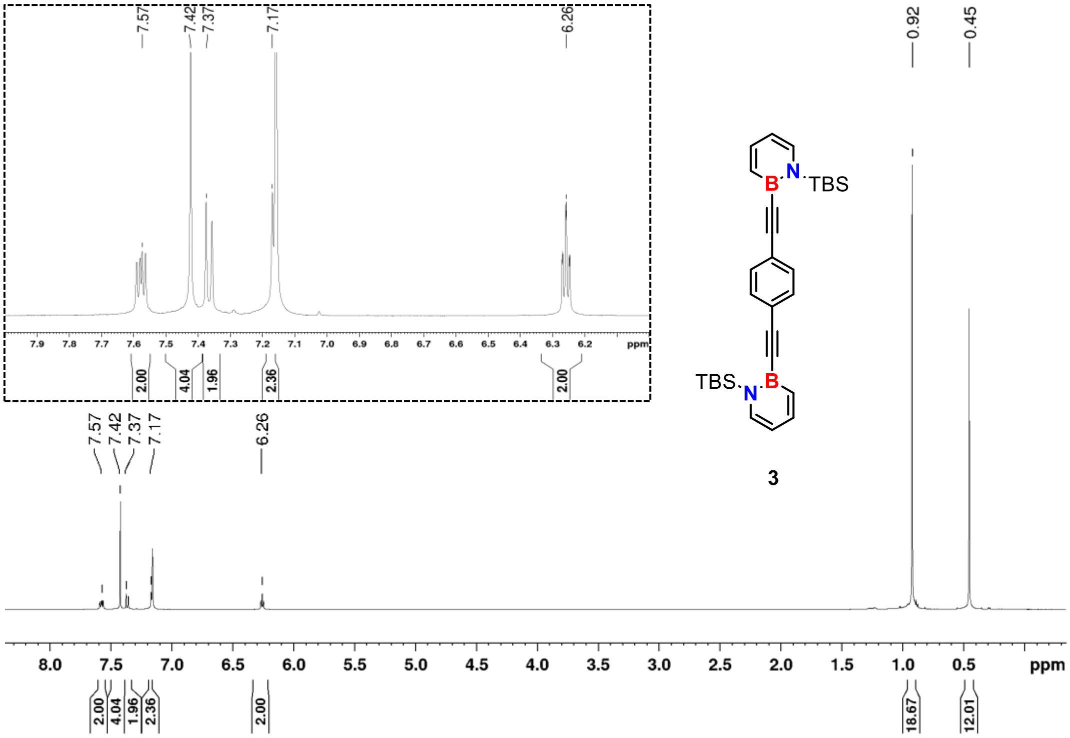

Figure S7. ^{1}H-NMR spectrum of **3** measured in benzene-d_6. The enlarged section shows the region between 6 and 8.5 ppm for a better visibility of the aromatic signals.

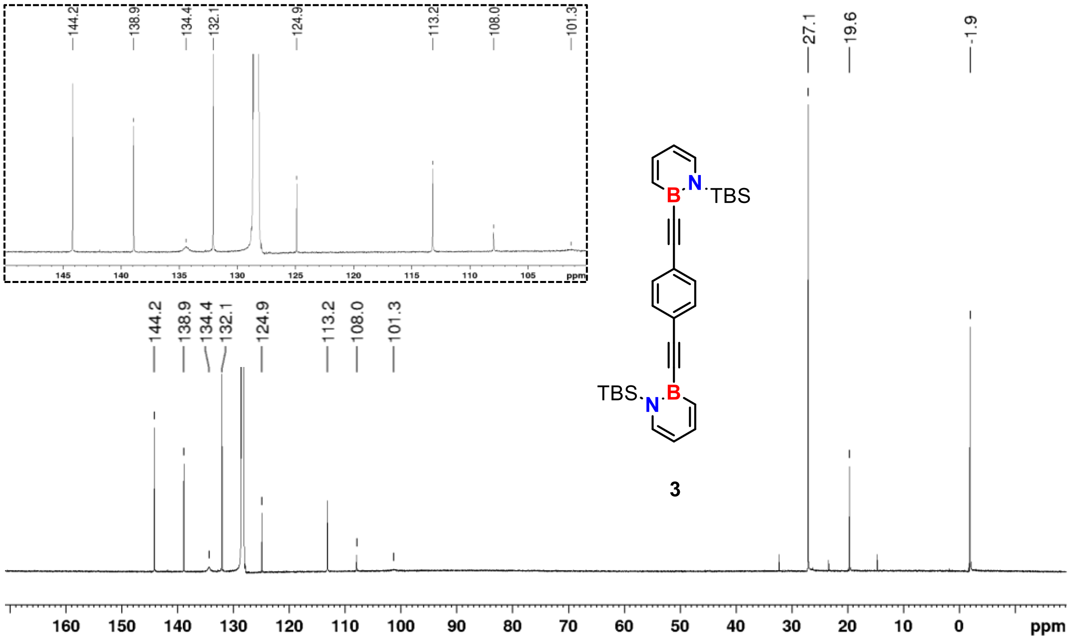

Figure S8. ^{13}C-{^{1}H} NMR spectrum of **3** measured in benzene-d6. The enlarged section shows the region between 150 and 100 ppm for a better visibility of the aromatic signals.

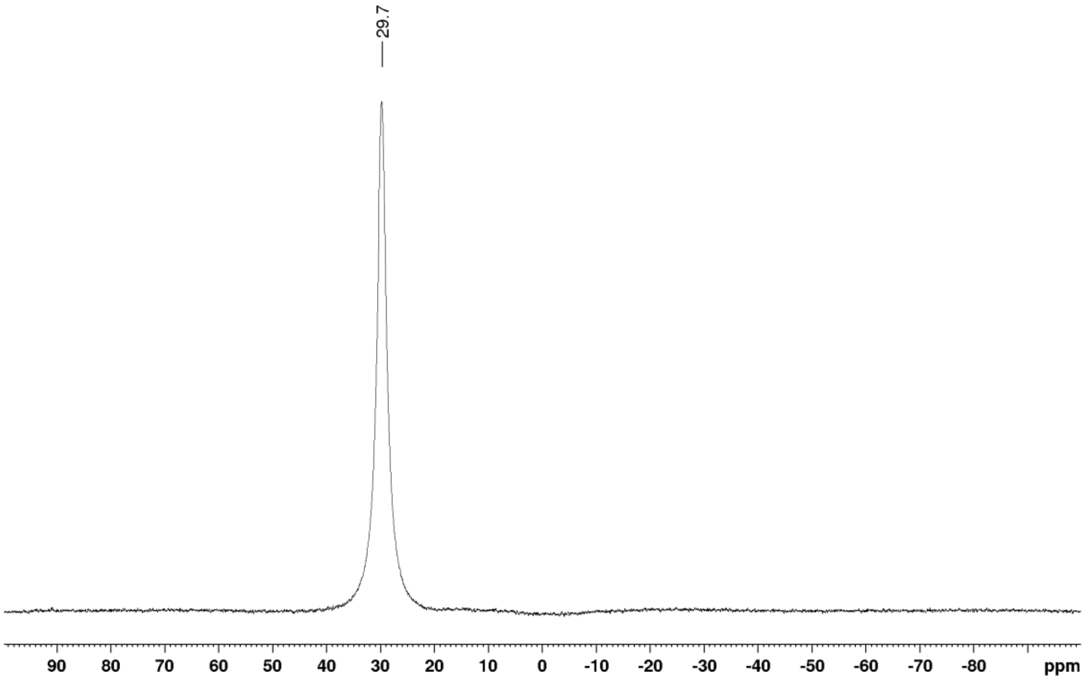

Figure S9. ^{11}B-NMR spectrum of **3** measured in benzene-d6.

1,3,5-Tris-(2-Ethinyl-1-tert-butyldimethylsilyl)-1,2-dihydro-1,2-azaborinin)benzene 4

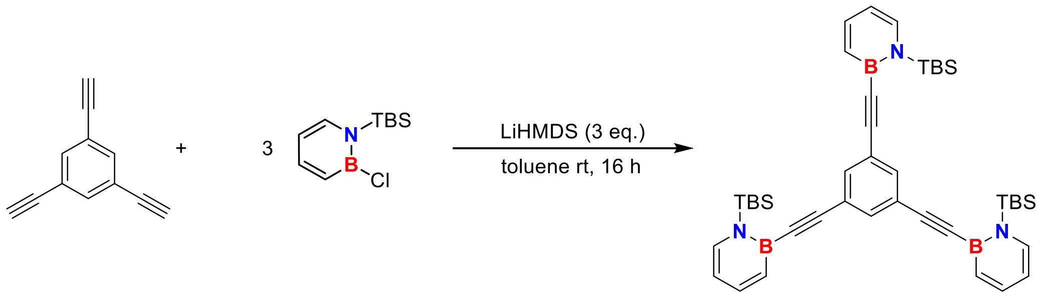

In a glovebox a solution of precursor dihydoazaborin **5** (0.88 g, 1.2 mmol, 3.5 eq.) was solved in 1 mL toluene. In a separate vessel the corresponding 1,3,5-*tris*-ethynylbenzene (0.04 g, 0.35 mmol, 1 eq.) was solved in 2 mL toluene and a solution of LiHMDS (1 M in hexane, 1.2 mL, 1.2 mmol, 3.5 eq.) was slowly added. The resulting suspension was treated with the previously prepared azaborinine solution and stirred for 16 h at room temperature. The reaction mixture is transferred into 5 mL of distilled water, and the organic phase is expanded by 2 mL of *n*-hexane. After the separation of the organic phase the aqueous phase was extracted three times with 5 mL of *n*-hexene. The collected organic layers were washed with 25 mL of brine, dried over magnesium sulfate, filtered and the solvent was removed in vacuo. The crude product was purified by column chromatography (neutral aluminium oxide, *n*-hexane/dichloromethane gradient from 100/0 to 85/15) followed by GPC size exclusion chromatography (*n*-hexane/dichloromethane 75/25). The product **4** was obtained as a colorless solid (75%).

^{1}H-NMR (600 MHz, **C_6D_6**): δ = 7.86 (s, 3H, H-14), 7.56 (dd, $^{3}J_{HH}$ = 11.18 Hz, $^{3}J_{HH}$ = 6.26 Hz, 3H, H-4), 7.32 (d, $^{3}J_{HH}$ = 11.18 Hz, 3H, H-6), 7.15 (d, $^{3}J_{HH}$ = 6.26 Hz, 3H, H-3), 6.25 (dt, $^{3}J_{HH}$ = 6.76 Hz, $^{4}J_{HH}$ = 1.50 Hz, 3H, H-5), 0.91 (s, 17H, H-10), 0.44 (s, 18H, H-8) ppm.

^{13}C-{^{1}H}-NMR (600 MHz, **C_6D_6**): δ = 144.2 (C4), 138.9 (C6), 134.5 (br., C3), 134.3 (C14), 128.7 (C13), 113.3 (C5), 106.6 (C12), 100.7 (br., C11), 27.1 (C10), 19.6 (C9), -2.0 (C8) ppm.

^{11}B-{^{1}H}-NMR (600 MHz, **C_6D_6**): δ = 29.6 ppm.

HR-MS (APCI): m/z calc. for $[M+H]^+$ 724.44470, found 724.44685.

$C_{42}H_{61}B_3N_3Si_3$

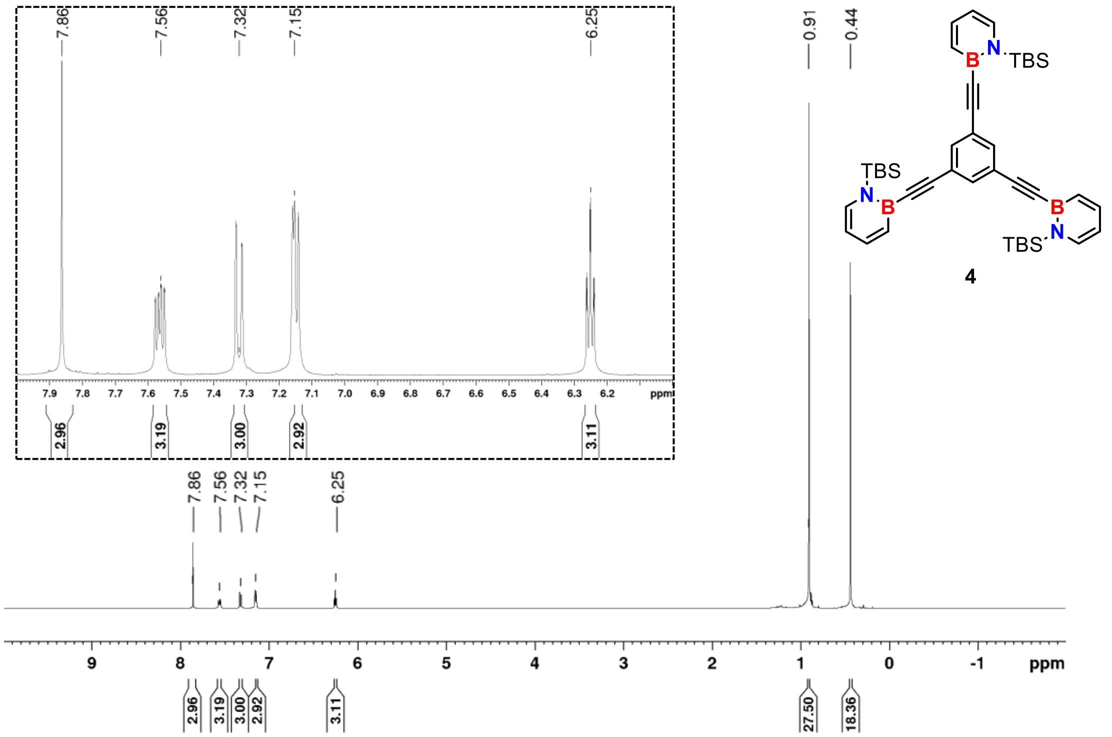

Figure S10. ^{1}H-NMR spectrum of **4** measured in benzene-d_6. The enlarged section shows the region between 6 and 8 ppm for a better visibility of the aromatic signals.

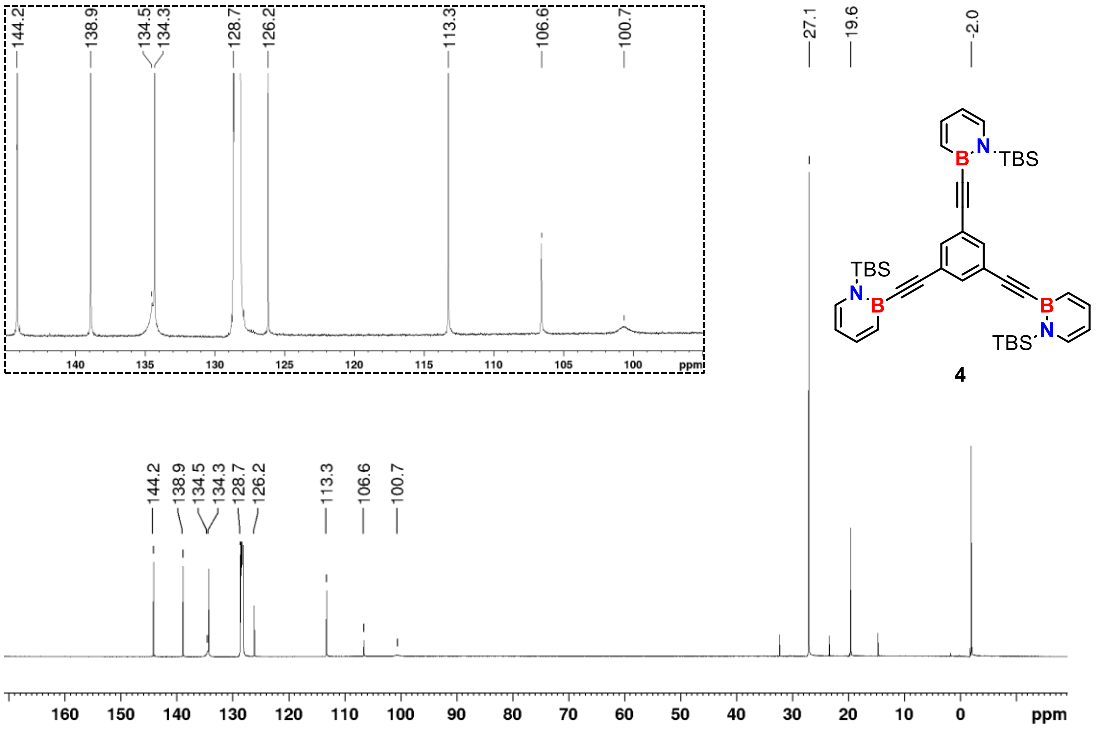

Figure S11. ^{13}C-{^{1}H}-NMR spectrum of **4** measured in benzene-d_6. The enlarged section shows the region between 95 and 150 ppm for a better visibility of the broad signals of the carbons next to the boron heteroatom.

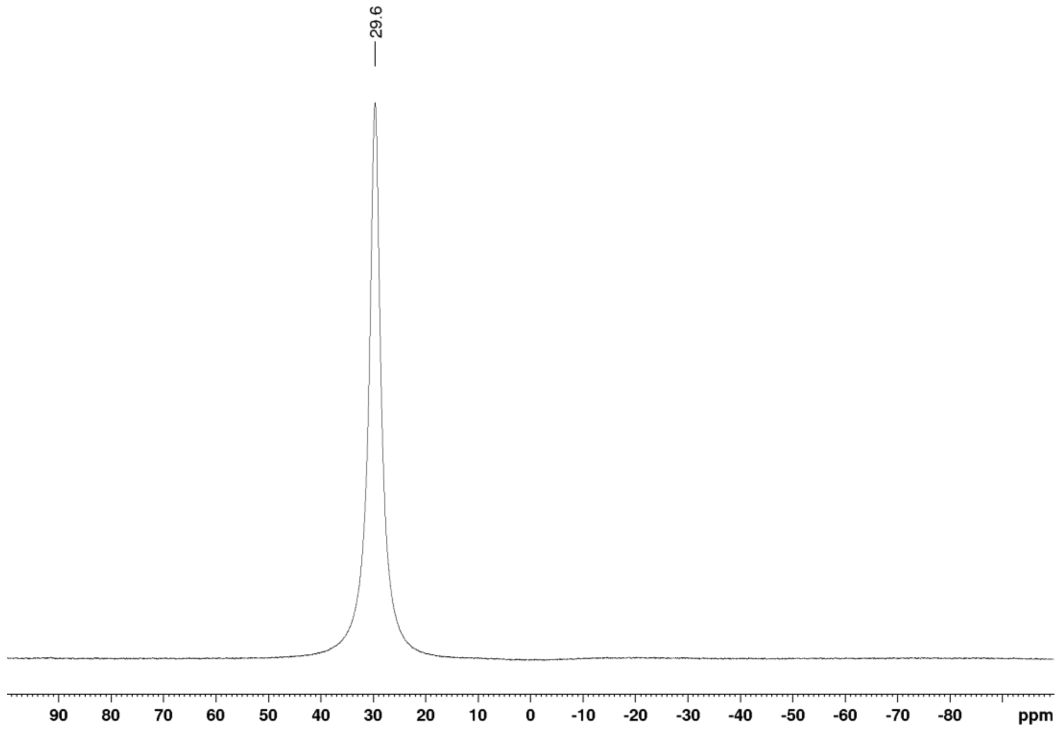

Figure S12. ^{11}B-NMR spectrum of **4** measured in benzene-d6.

1-(Tert-butyldimethylsilyl)-2-(phenylethynyl)-1,2-dihydro-1,2-azaborinine 5

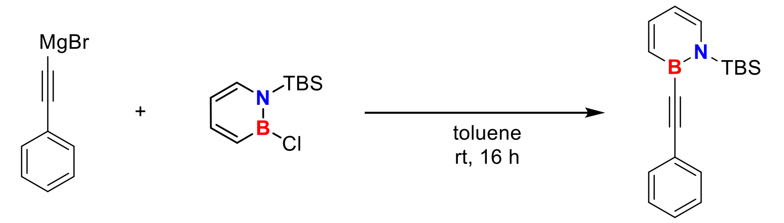

In a glovebox a solution of precursor dihydoazaborin **5** (0.2 g, 0.88 mmol, 1 eq.) was solved in 1 mL toluene. A solution of phenylethynyl *Grignard* reagent (1 M in THF, 1.2 mL, 1.2 eq.) was added slowly. The resulting solution was stirred for 16 h at room temperature. The reaction mixture was transferred into 5 mL of distilled water, and the organic phase is expanded by 2 mL of *n*-hexane. After the separation of the organic phase the aqueous phase was extracted three times with 5 mL of *n*-hexane. The collected organic layers were washed with 25 mL of brine, dried over magnesium sulfate, filtered and the solvent was removed in vacuo. The crude product was purified by column chromatography (neutral aluminium oxide, *n*-hexane/dichloromethane gradient from 100/0 to 80/15). The product **4** was obtained as a colorless oil (70%).

NMR spectra are consistent with data Liu ad coworkers published earlier.[7]

4. Irradiation experiments

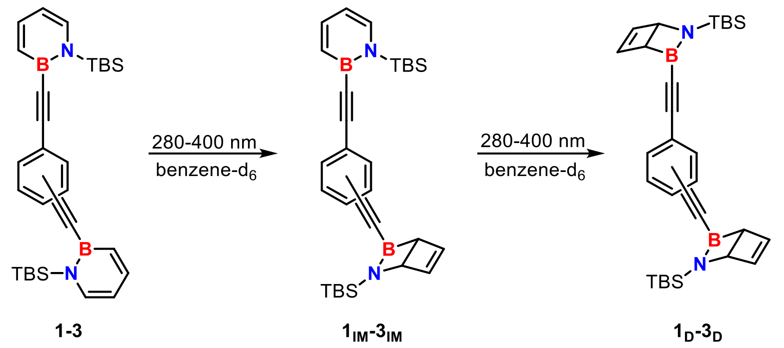

All irradiations were carried out using a dichroic mirror that selects the wavelength range 280-400 nm, without additional filters. The samples were placed in quartz J. Young NMR tubes and dissolved in deuterated benzene under argon before the irradiation. The exact irradiation time was dependent on the concentrations of the solution. The irradiation times to reach above 95% conversion to the *Dewar* isomer in 0.1 M solutions were 15 minutes for **6**, 30 minutes for **1-2** and 40 minutes for **4**. Due to solubility issues only 0.05 M solutions of **3** were prepared. The irradiations time for **3** was 20-25 minutes. The irradiation yielded the compounds $\mathbf{1}_{\mathbf{\mathit{Dewar}}}$-$\mathbf{6}_{\mathbf{Dewar}}$ in almost quantitative yield.

Comment on ^{11}B NMR data:

For all compounds the ^{1}H and the ^{13}C-{^{1}H}-spectra were recorded first. To capture the signals of the (quaternary) carbon atoms next to boron many scans are required, therefore these measurements took up to 9 hours. As the ^{11}B NMR spectra were recorded after the ^{13}C-{^{1}H}-spectra, some of the *Dewar* isomer already reacted back to the dihydroazaborinine during the measurement. For that reason, a small peak of the compound **1-4** or **6** is observable in the respective ^{11}B spectrum.

NMR data of 1*Dewar*

^{1}H-NMR (600 MHz, **C_6D_6**): δ =7.39 (m, 2H, H-14), 6.73 (m, 2H, H-15), 6.52 (br. m, 2H, H-4), 6.23 (d, $^{3}J_{HH}$ = 2.19 Hz, 2H, H-5), 4.61 (ps. t, $^{3}J_{HH}$ = 2.19 Hz, 2H, H-6), 3.04 (br. m, 2H, H-3), 1.01 (s, 18H, H-10), 0.29 (s, 6H, H-8), 0.23 (s, 6H, H-8) ppm.

^{13}C-{^{1}H}-NMR (600 MHz, **C_6D_6**): δ =144.0 (C4), 139.4 (C5), 132.9 (C14), 128.5 (C15), 126.0 (C13), 106.6 (C12) 97.1 (C11), 64.2 (C5), 43.2 (br., C3) 26.5 (C10), 18.4 (C9), -4.8 (C8), -5.4 (C8) ppm.

^{11}B-{^{1}H}-NMR (600 MHz, **C_6D_6**): δ = 41.5 ppm.

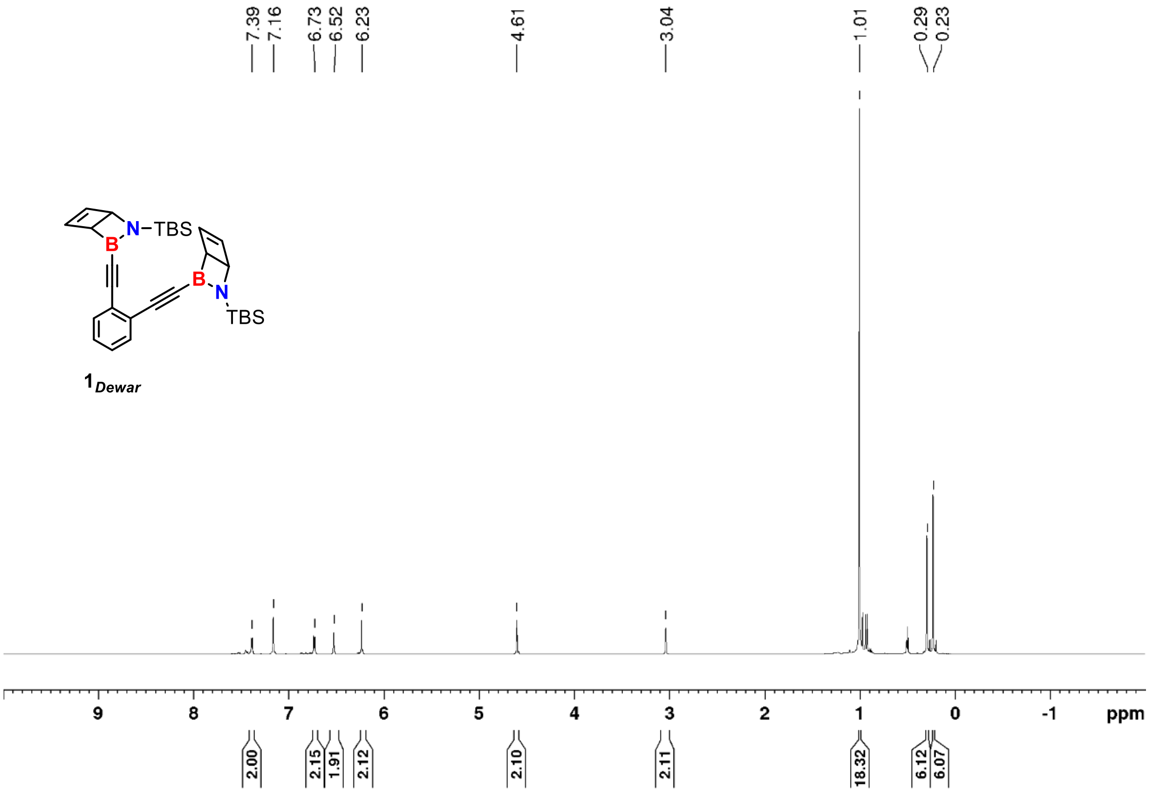

Figure S13. ^{1}H-NMR spectrum of **1***Dewar* measured in benzene-d$_6$.

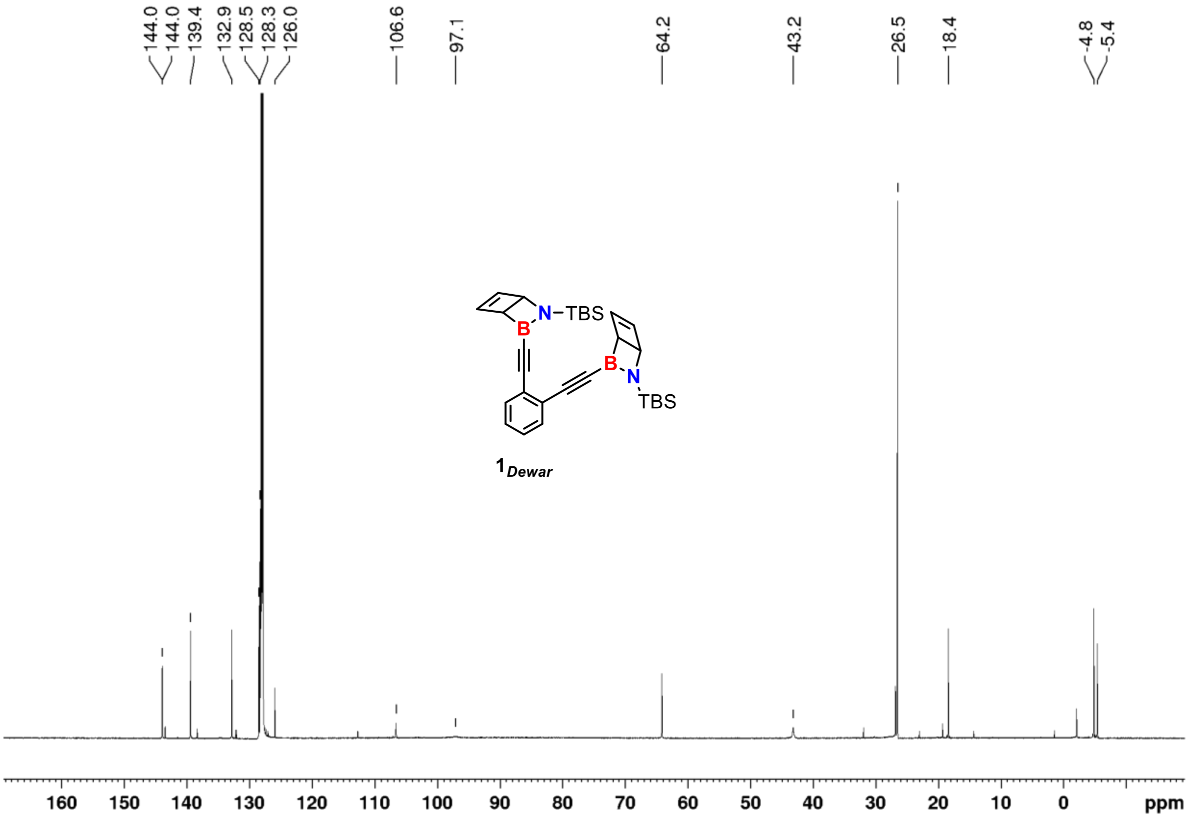

Figure S14. ^{13}C-{^{1}H}-NMR spectrum of **1***Dewar* measured in benzene-d$_6$.

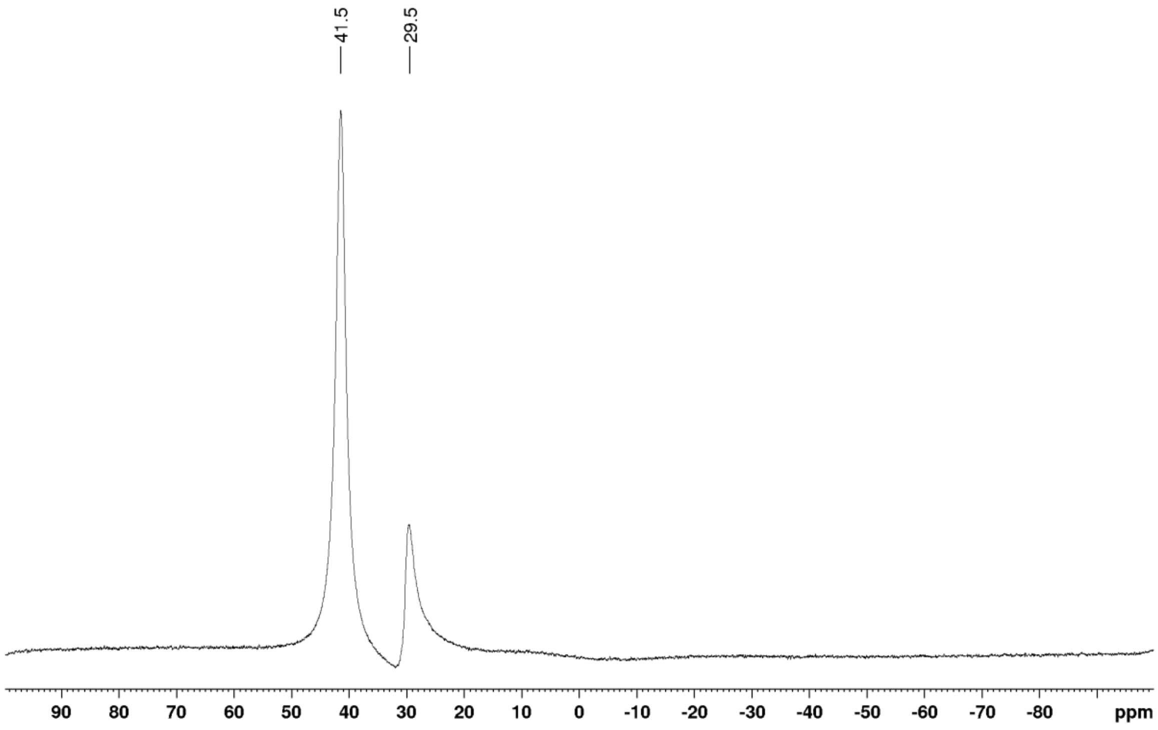

Figure S15. ^{11}B-NMR spectrum of **1***Dewar* measured in benzene-d$_6$.

NMR data of 2*Dewar*

^{1}H-NMR (600 MHz, **C_6D_6**): δ = 7.84 (ps. t, $^4J_{HH}$ = 1.53 Hz, 1H, H-15), 7.28 (dd, $^3J_{HH}$ = 7.87 Hz, $^4J_{HH}$ = 1.53 Hz, 3H H-), 6.72 (t, $^3J_{HH}$ = 7.87 Hz, 1H, H-16), 6.46 (m, 2H, H-4), 6.20 (d, $^3J_{HH}$ = 2.33 Hz, 2H, H-5), 4.56 (t, $^3J_{HH}$ = 2.24 Hz, 2H, H-6), 2.96 (m, 2H, H-3), 0.98 (s, 18H, H-10), 0.21 (s, 6H, H-8), 0.16 (s, 6H, H-8) ppm.

^{13}C-{^{1}H}-NMR (600 MHz, **C_6D_6**): δ = 143.8 (C4), 139.6 (C5), 135.6 (C15), 132.4 (C14), 128.9 (C16), 124.1 (C13), 107.0 (C12), 94.1 (br., C11), 64.2 (C5), 42.9 (br., C3), 26.4 (C10), 18.4 (C9), -5.1 (C8), -5.6 (C8) ppm.

^{11}B-{^{1}H}-NMR (600 MHz, **C_6D_6**): δ = 41.3 ppm.

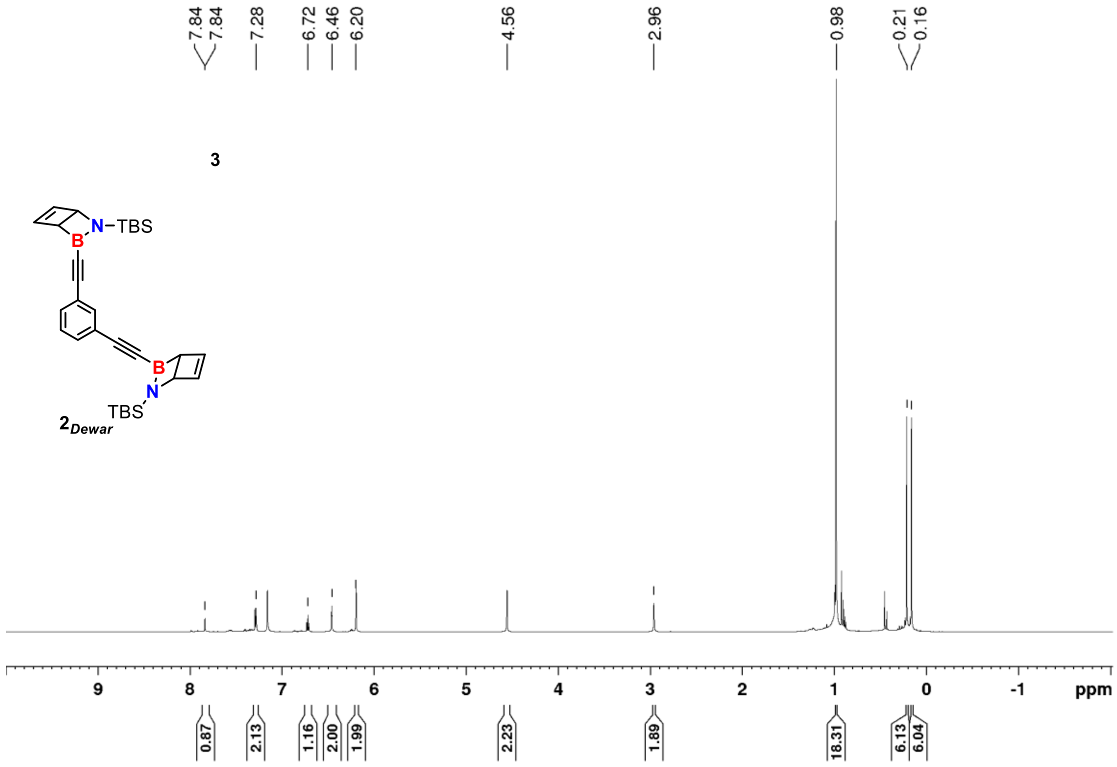

Figure S16. ^{1}H-NMR spectrum of **2*Dewar*** measured in benzene-d_6.

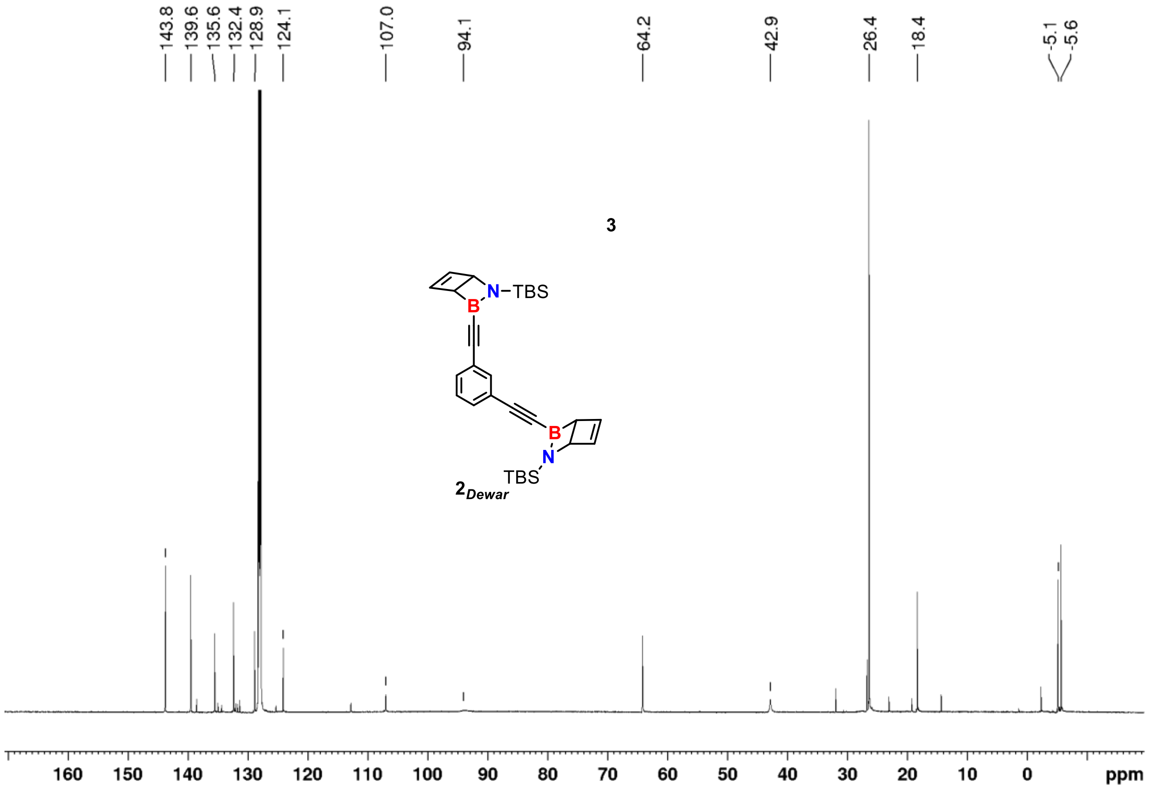

Figure S17. ^{13}C-{^{1}H}-NMR spectrum of **2*Dewar*** measured in benzene-d$_6$.

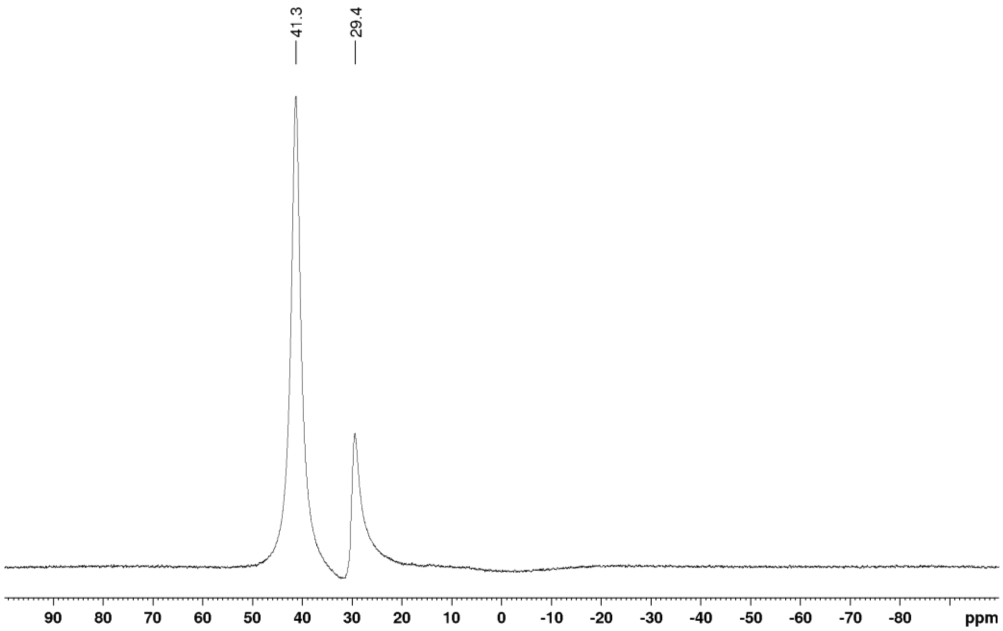

Figure S18. ^{11}B-NMR spectrum of **2*Dewar*** measured in benzene-d$_6$.

NMR data of 3*Dewar*

^{1}H-NMR (600 MHz, **C_6D_6**): δ = 7.27 (s, 4H, H-14), 6.48 (m, 2H, H-4), 6.21 (d, $^{3}J_{HH}$ = 2.19 Hz, 2H, H-5), 4.57 (t, $^{3}J_{HH}$ = 2.19 Hz, 2H, H-6), 2.98 (m, 2H, H-3), 0.99 (s, 18H, H-10), 0.23 (s, 6H, H-8), 0.18 (s, 6H, H-8) ppm.

^{13}C-{^{1}H}-NMR (600 MHz, **C_6D_6**): δ = 143.8 (C4), 139.6 (C5), 132.2 (C14), 124.1 (C13), 107.6 (C12), 95.1 (C11), 64.2 (C6), 42.9 (br., C3), 26.4 (C10), 18.4 (C9), -5.1 (C8), -5.6 (C8) ppm.

^{11}B-{^{1}H}-NMR (600 MHz, **C_6D_6**): δ = 41.5 ppm.

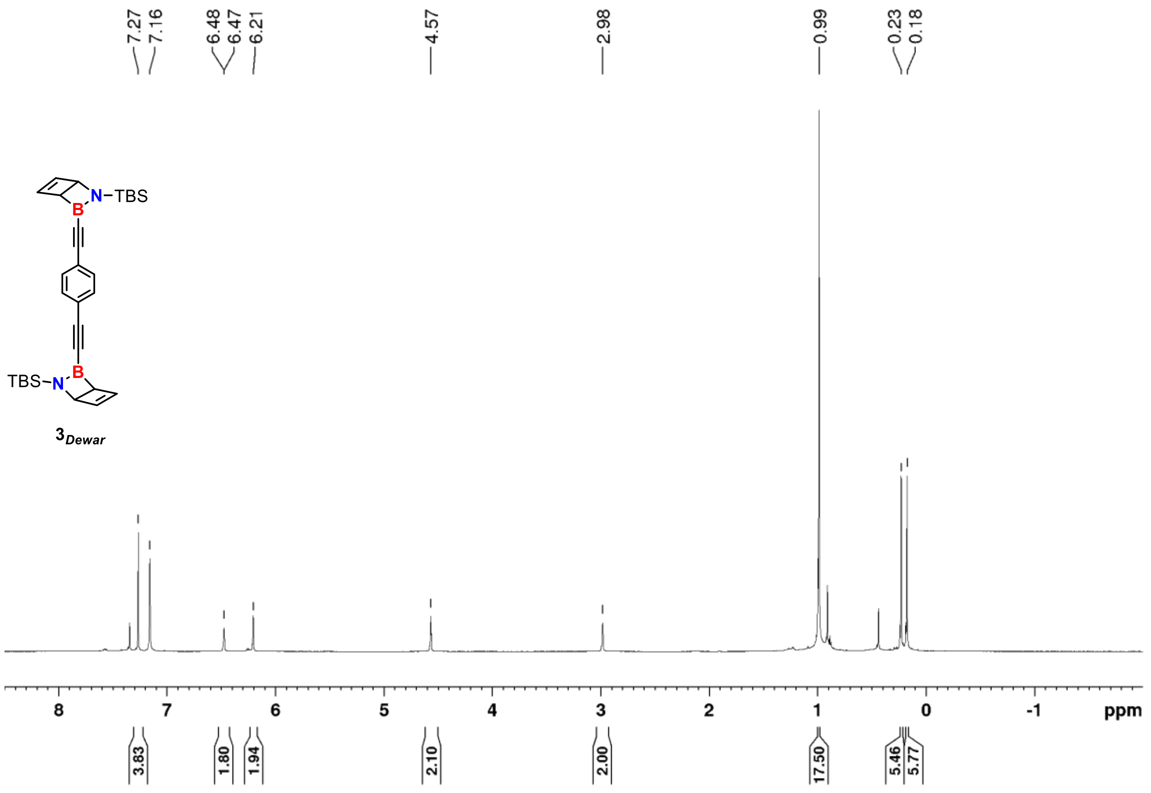

Figure S19. ^{1}H-NMR spectrum of **3*Dewar*** measured in benzene-d6.

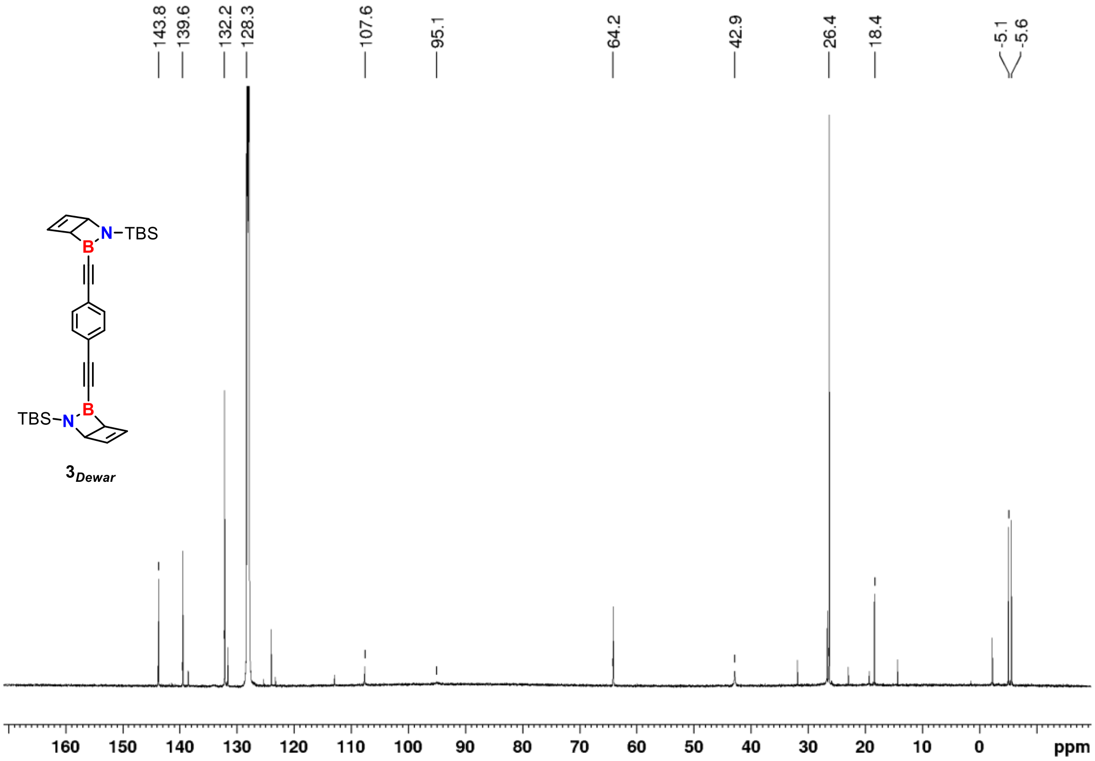

Figure S20. ^{13}C-{^{1}H}-NMR spectrum of **3***Dewar* measured in benzene-d$_6$.

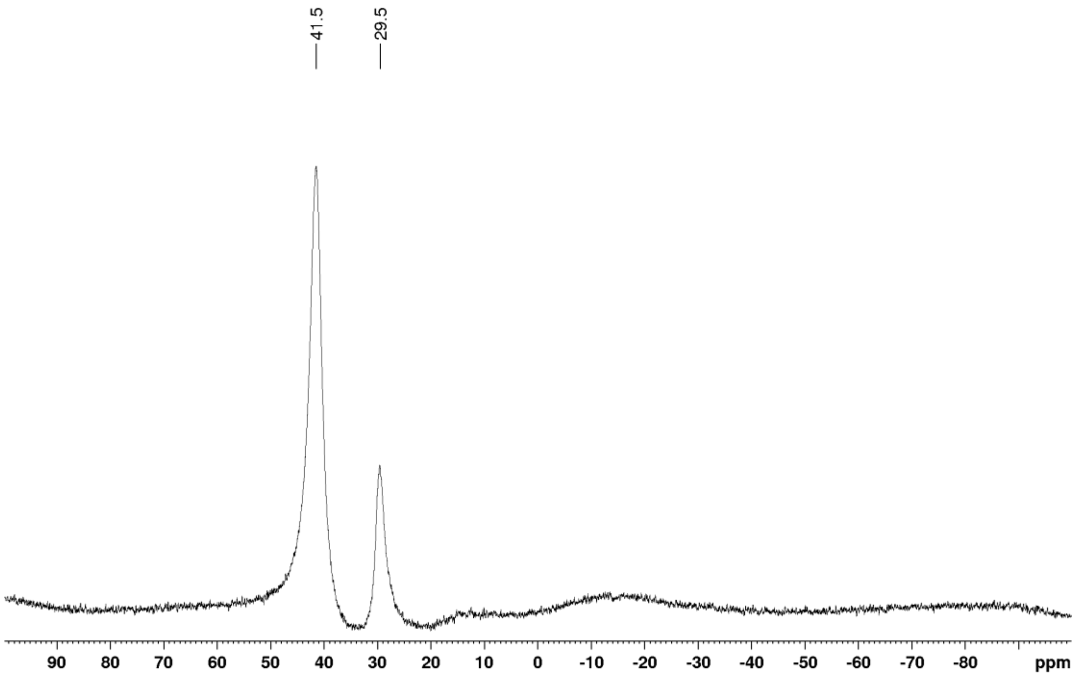

Figure S21. ^{11}B-NMR spectrum of **3***Dewar* measured in benzene-d$_6$.

NMR data of 4*Dewar*

^{1}H-NMR (600 MHz, **C_6D_6**): δ = 7.65 (s,3H, H-14), 6.45 (m, 2H, H-4), 6.19 (d, $^3J_{HH}$ = 2.22 Hz, 2H, H-5), 4.54 (t, $^3J_{HH}$ = 2.22 Hz, 2H, H-6), 2.93 (m, 2H, H-3), 0.96 (s, 18H, H-10), 0.19 (s, 6H, H-8), 0.14 (s, 6H, H-8) ppm.

^{13}C-{^{1}H}-NMR (600 MHz, **C_6D_6**): δ = 143.7 (C4), 139.5 (C5), 135.5 (C14), 124.7 (C13), 105.9 (C12), 94.4 (C11), 64.2 (C6), 42.8 (br., C3), 26.3 (C10), 18.4 (C9), -5.1 (C8), -5.7 (C8) ppm.

^{11}B-{^{1}H}-NMR (600 MHz, **C_6D_6**): δ = 41.3 ppm.

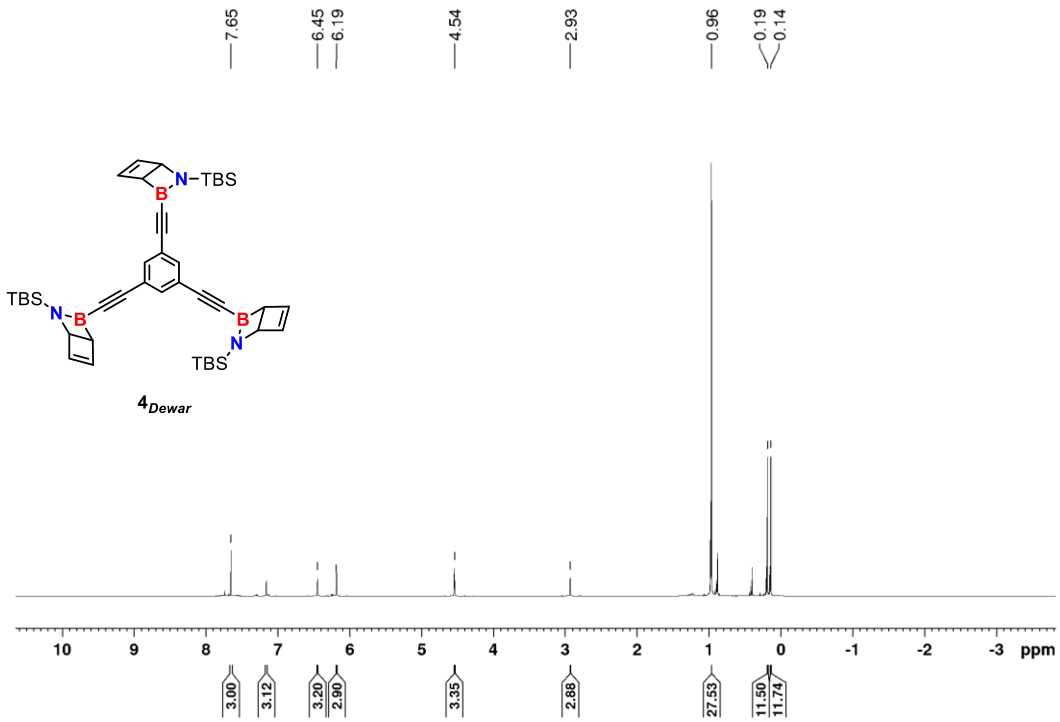

Figure S22. ^{1}H-NMR spectrum of **3*Dewar*** measured in benzene-d6.

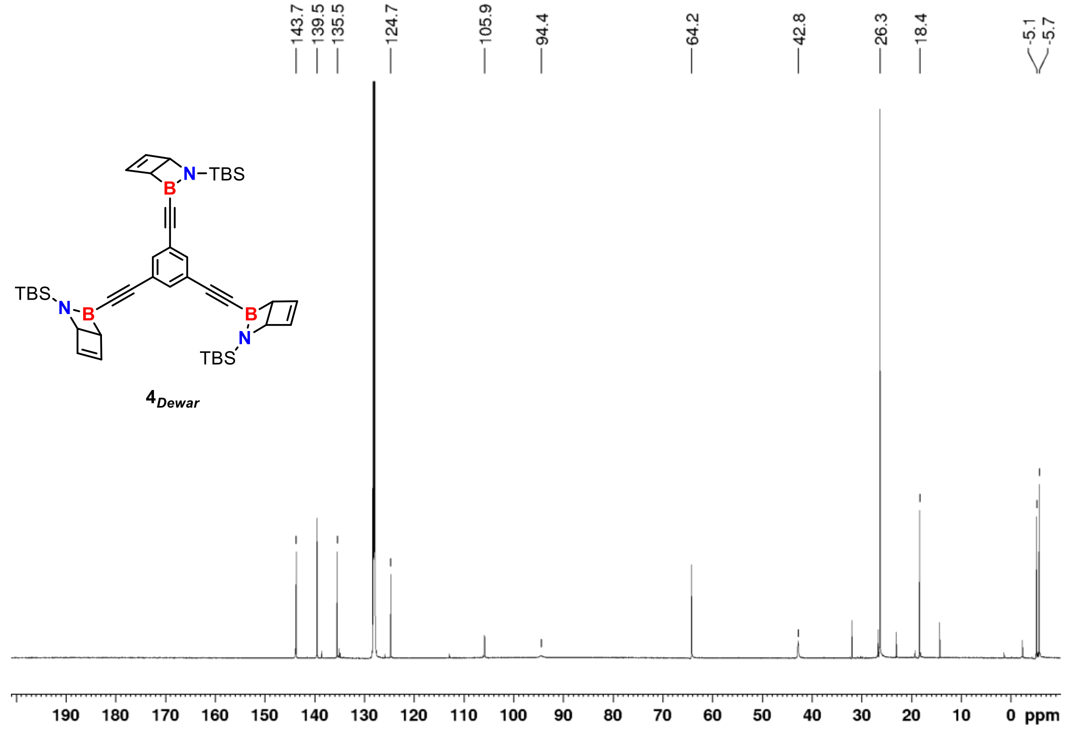

Figure S23. ^{13}C-{^{1}H}-NMR spectrum of **4*Dewar*** measured in benzene-d$_6$.

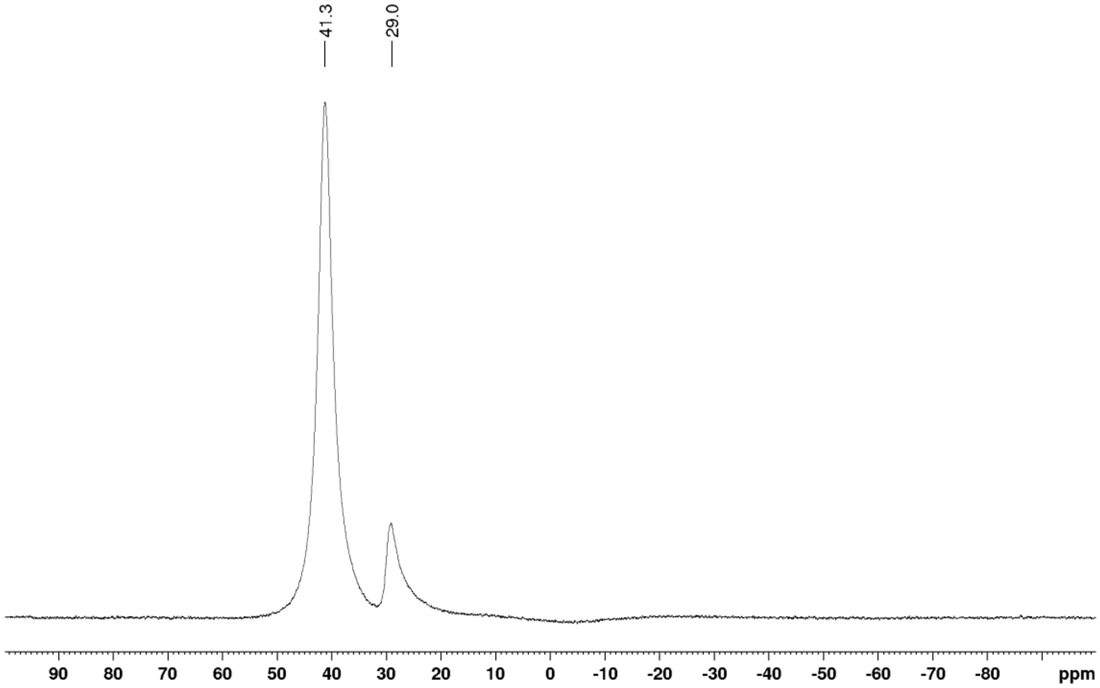

Figure S24. ^{11}B-NMR spectrum of **4*Dewar*** measured in benzene-d$_6$.

NMR data of 6*Dewar*

^{1}**H-NMR** (600 MHz, $\mathbf{C_6D_6}$): δ = 7.48 (m, 2H, H-14), 6.94 (m, 3H, H-15/16), 6.48 (m, 1H, H-4), 6.22 (d, $^{3}J_{HH}$ = 2.29 Hz, 1H, H-5), 4.58 (t, $^{3}J_{HH}$ = 2.29 Hz, 1H, H-6), 3.00 (m, 1H, H-3), 1.00 (s, 9H, H-10), 0.25 (s, 3H, H-8), 0.19 (s, 3H, H-8) ppm.

^{13}**C-{^{1}H}-NMR** (600 MHz, $\mathbf{C_6D_6}$): δ =143.8 (C4), 139.6 (C3), 132.3 (C14), 129.0 (C16), 128.6 (C15), 123.7 (C13), 107.9 (C12) 64.1 (C6), 43.0 (C3), 26.4 (C10), 18.4 (C9), -5.1 (C8), -5.6 (C8) ppm.

^{11}**B-{^{1}H}-NMR** (600 MHz, $\mathbf{C_6D_6}$): δ = 40.0 ppm.

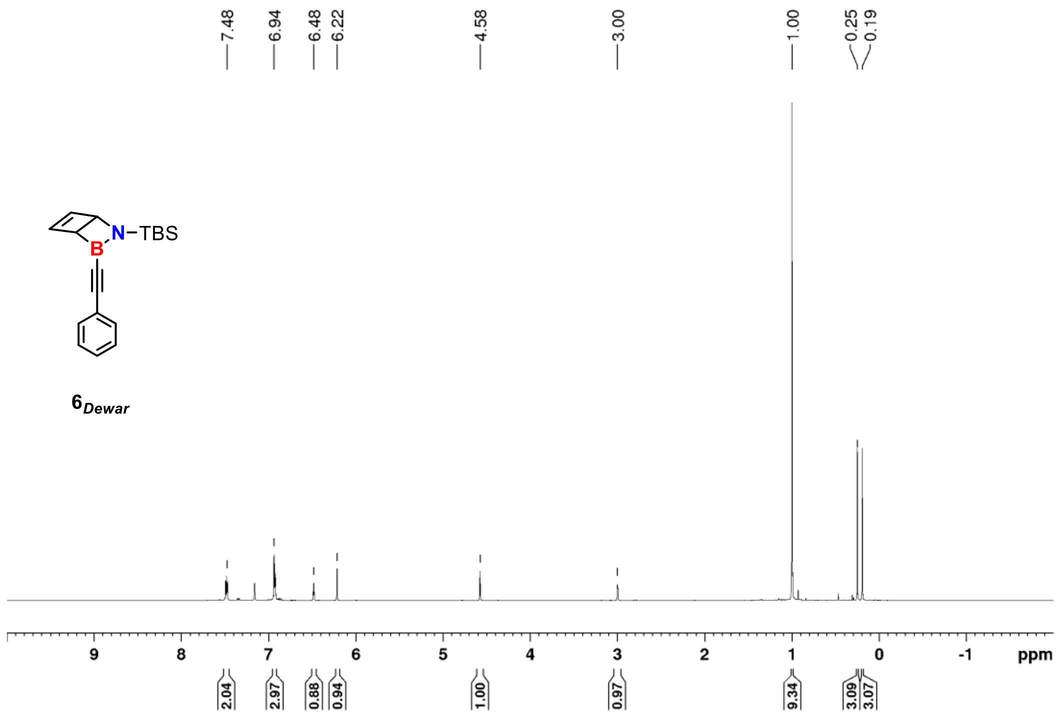

Figure S25. ^{1}H-NMR spectrum of **6***Dewar* measured in benzene-d$_6$.

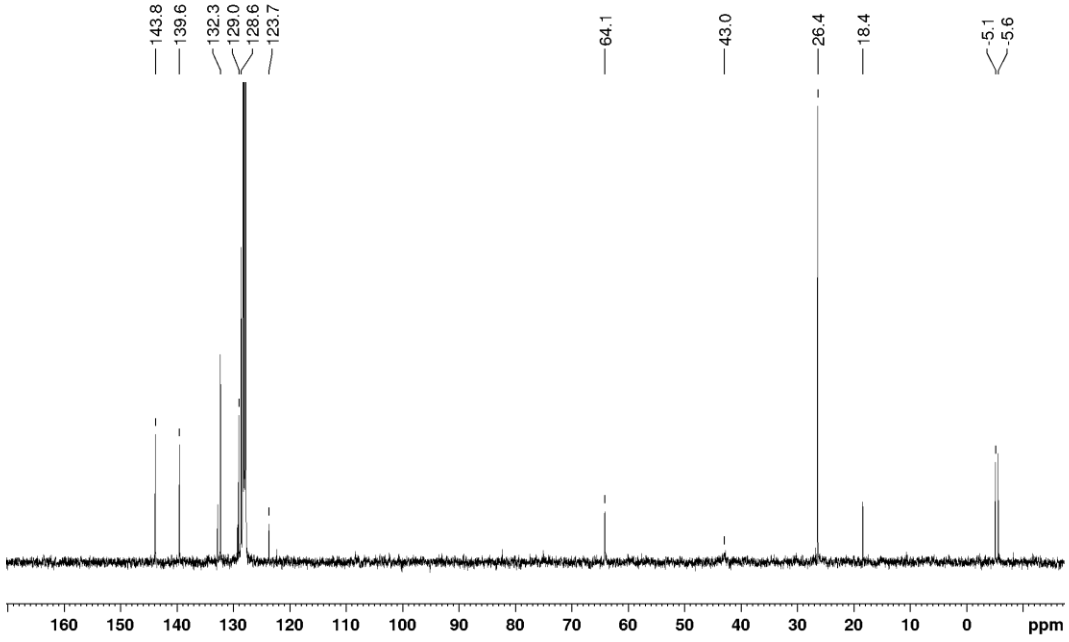

Figure S26. ^{13}C-{^{1}H}-NMR spectrum of **6***Dewar* measured in benzene-d_6.

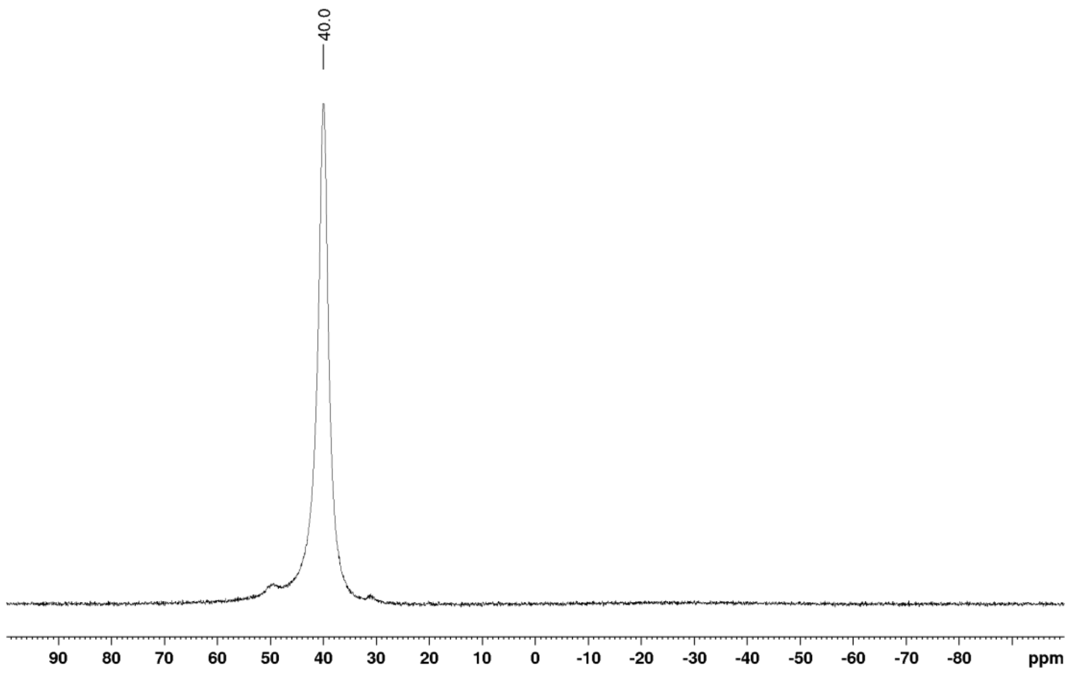

Figure S27. ^{11}B-NMR spectrum of **6***Dewar* measured in benzene-d_6.

5. Kinetic experiments

Kinetic studies: The kinetic constants were measured for T = 348, 343, 338 and 333 K using NMR spectroscopy (Bruker Avance III HDX 600) in case of **1**, **2**, **4** and **6**, while T = 330, 335, 340 and 345 K were analysed for **3**. For each temperature one experiment was performed. A 0.1 M solution of the given *Dewar* species in dried, degassed, deuterated benzene was placed in a J. Young quartz tube. The sample was allowed to reach the desired temperature, then every 5 min a ^{1}H NMR spectrum was recorded (16 scans; total experiment time = 126 s) for at least 3 half-life periods.

Table S1: Duration of the kinetic NMR measurements at the four different temperatures for the compounds **1-5**.

T in K	t (h) for **1**	t (h) for **2**	t (h) for **3**	t (h) for **4**	t (h) for **6**
333	16.5	10.5	16 [a]	10.5	12
338	9	5	8 [b]	9	8.5
343	3	3	4 [c]	2.5	3.5
348	2	1.5	2 [d]	1.7	1.5

a: measured at 330 K, b: measured at 335 K, c: measured at 340 K, d: measured at 345 K.

For determining the rate constants, the integrals of the signals of the TBS-methyl groups were used. The experiments yielded the following rate constants.

Table S2: Rate constants for the first, second and third ring opening (ro) at the four different temperatures for the compounds **1-5**.

T in K		k ($s^{-1}\cdot10^4$) for **1**	k ($s^{-1}\cdot10^4$) for **2**	k ($s^{-1}\cdot10^4$) for **3**	k ($s^{-1}\cdot10^4$) for **4**	k ($s^{-1}\cdot10^4$) for **6**
333	1. ro	2.068	2.416	1.763 [a]	4.207	0.537
	2. ro	1.382	1.302	1.194 [a]	2.844	-
	3. ro	-	-	-	1.430	-
338	1. ro	3.470	3.914	2.981 [b]	5.771	0.859
	2. ro	2.372	2.780	2.463 [b]	6.210	-
	3. ro	-	-	-	2.942	-
343	1. ro	6.216	7.050	5.048 [c]	11.10	1.323
	2. ro	3.838	5.110	4.228 [c]	9.766	-
	3. ro	-	-	-	4.537	-
348	1. ro	10.20	11.10	7.610 [d]	18.00	2.144
	2. ro	6.233	6.969	6.784 [d]	15.20	-
	3. ro	-	-	-	7.925	-

a: measured at 330 K, b: measured at 335 K, c: measured at 340 K, d: measured at 345 K.

Arrhenius treatment of the measured data

The Arrhenius equation is expressed as follows:

$$k = A \cdot e^{-\frac{E_a}{R \cdot T}}$$

A denotes the pre-exponential factor, R is the universal gas constant, k represents the rate constant, T is the absolute temperature, and E_a corresponds to the activation energy (activation barrier). By taking the natural logarithm, the equation can be rearranged into the following form:

$$\ln(k) = ln\,(A) - \frac{E_a}{R} \cdot \frac{1}{T}$$

If ln(k) is plotted against the reciprocal temperature (1/T), this yields a straight line of the general form

$$y = a + b \cdot x$$

with a being the y-intercept and b is the slope. Consequently, the y-intercept a and the slope b of the Arrhenius plot correlate with the activation barrier and the pre-exponential factor A, respectively, as described by the following equations.

$$A = e^{a}$$

$$E_a = -b \cdot R$$

The Arrhenius plot derived from the experimental data, along with the equations of the corresponding linear fits, are presented below.

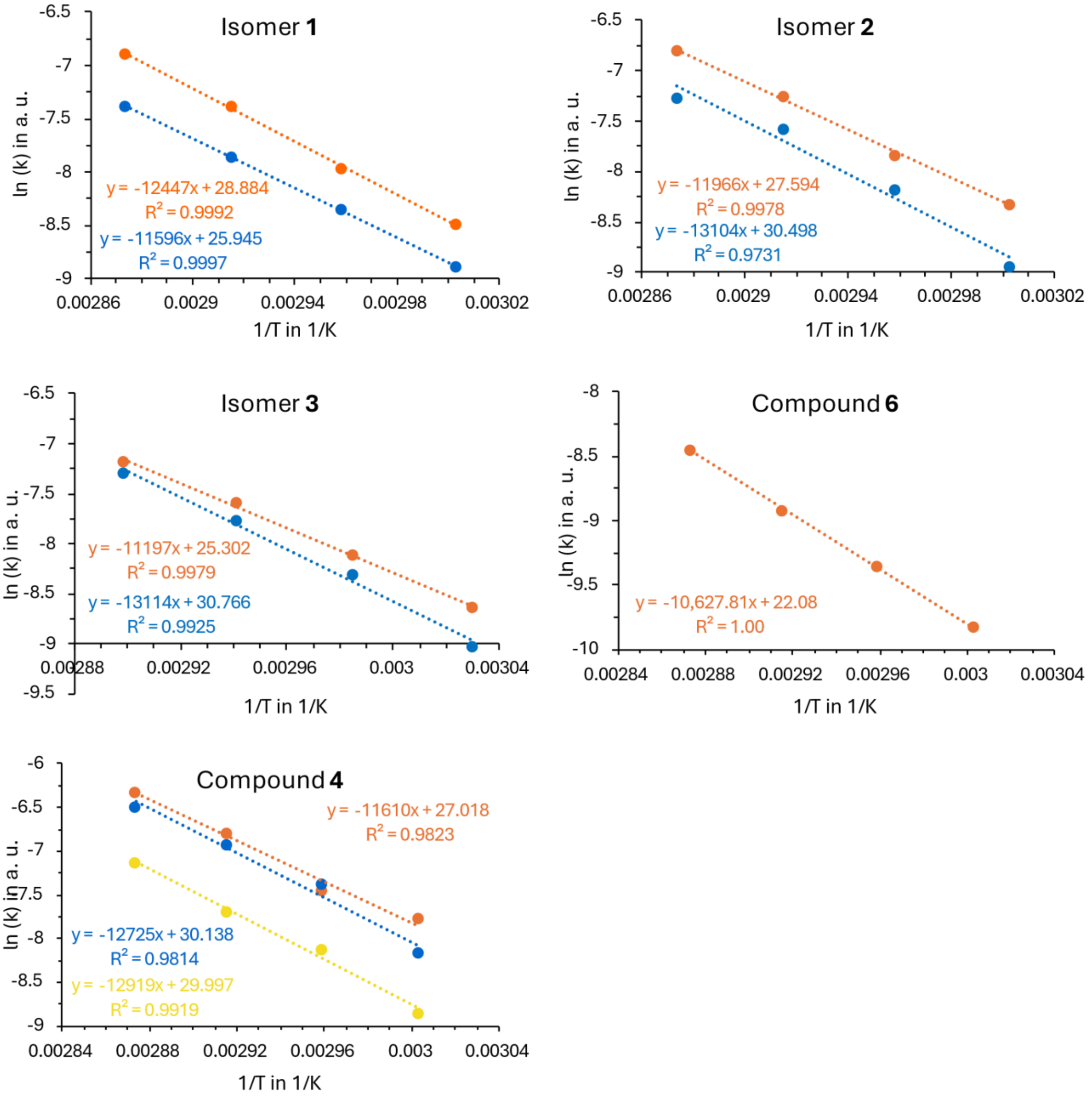

Figure S28: Arrhenius plots for the compounds **1-5**. The first ring opening is given in orange, the second ring opening is given in blue and the third ring opening is given in yellow.

Eyring treatment of the data

The Eyring equation is expressed as follows:

$$k = \frac{k_B T}{h} \cdot e^{\frac{\Delta S^{\ddagger}}{R}} \cdot e^{-\frac{\Delta H^{\ddagger}}{R \cdot T}}$$

κ denotes the transmission coefficient, R is the universal gas constant, k_B gives the Boltzmann constant, h is the Planck constant, k represents the rate constant, T is the absolute temperature, and $\Delta S^{\ddagger}$ and $\Delta H^{\ddagger}$ corresponds to the entropy or enthalpy of activation. One can put this equation into the following form:

$$\ln\left(\frac{k}{T}\right) = -\frac{\Delta H^{\ddagger}}{R} \cdot \frac{1}{T} + \ln\left(\frac{k_B}{h}\right) + \frac{\Delta S^{\ddagger}}{R}$$

If ln(k/T) is plotted against the reciprocal temperature (1/T), this yields a straight line of the general form

$$y = a + b \cdot x$$

with a being the y-intercept and b is the slope. Consequently, the y-intercept a and the slope b of the Eyring plot correlate with the entropy of activation and the enthalpy of activation, respectively, as described by the following equations.

$$\Delta S^{\ddagger} = \left(a - ln\left(\frac{k_B}{h}\right)\right) \cdot R$$

$$\Delta H^{\ddagger} = -b \cdot R$$

The Eyring plots derived from the experimental data, along with the equations of the corresponding linear fits, are presented below.

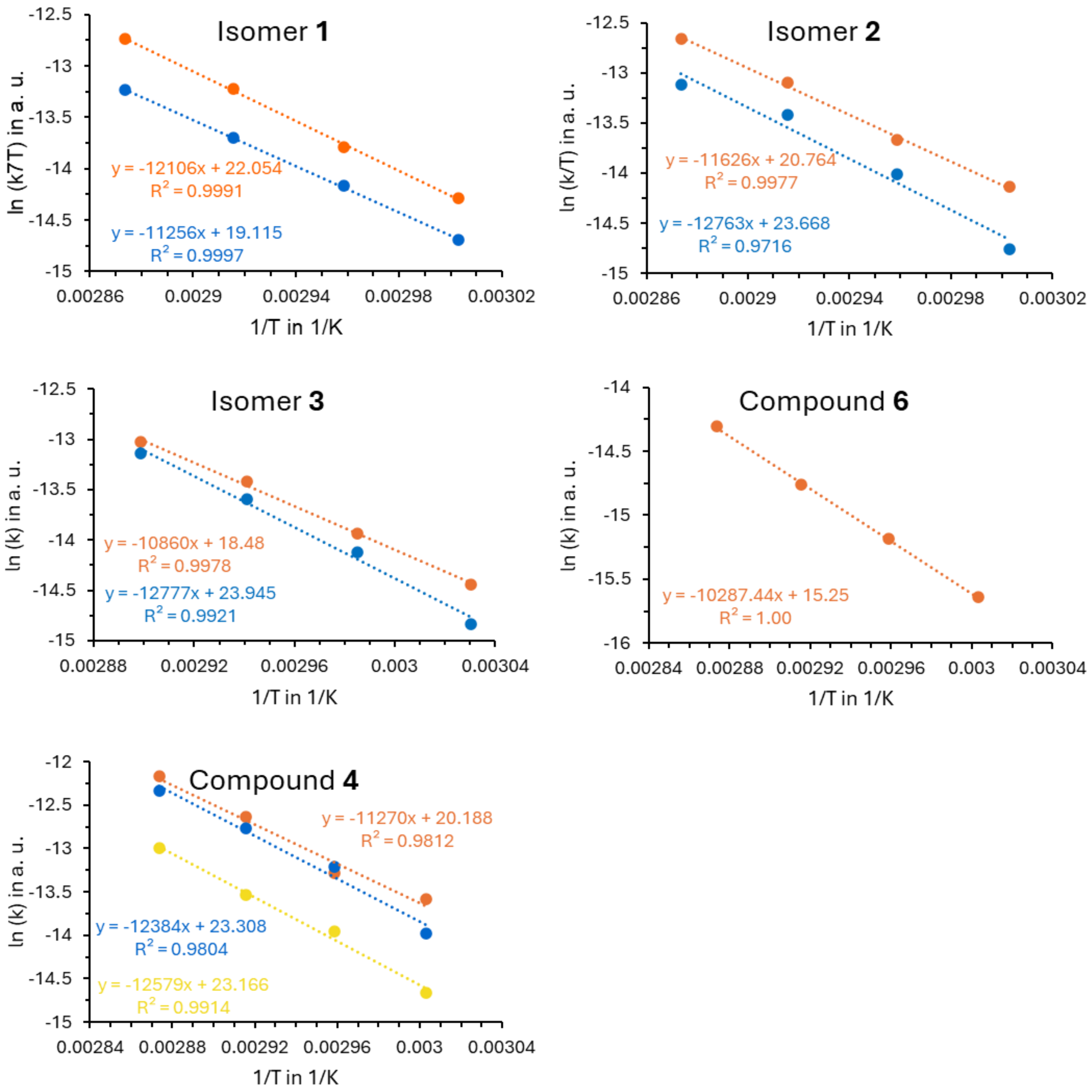

Figure S29. Eyring plots of the compounds **1-5**. The first ring opening is given in orange, the second ring opening is given in blue and the third ring opening is given in yellow.

Derivation of the time dependence of the concentrations

For deriving the concentration dependence, the following assumptions are made. The reaction under consideration is of the type:

$$A \xrightarrow{k_1} B \xrightarrow{k_2} C \xrightarrow{k_3} D$$

The three processes are assumed to follow irreversible first order kinetics and have the rate constants k_1, k_2 and k_3. This is in very good agreement with our experimental findings considering their behavior once the previous species or intermediate has mainly disappeared. The initial concentration of compound A is $A_0 = 1$. While the initial concentrations of B and C are $B_0 = C_0 = 0$.

Compound A follows a simple first order kinetic, therefore its concentration is given as:

$$\frac{d[A]}{dt} = -k_1 \cdot [A]$$

This equation can be transformed to

$$\frac{d[A]}{[A]} = -k_1 \cdot dt$$

An integration from A_0 to A (left side) or 0 to t (right side), respectively, results in

$$\ln[A] - \ln A_0 = -k_1 \cdot t$$

which finally rearranges to the well-known formula equation 1.

$$\boldsymbol{[A] = A_0 \cdot e^{-k_1 \cdot t}} \qquad \textit{(S1)}$$

For compound B the time dependence of the concentration can be described as:

$$\frac{d[B]}{dt} = k_1 \cdot [A] - k_2 \cdot [B]$$

If equations 1 is inserted in this term it can be rearranged to

$$\frac{d[B]}{dt} + k_2 \cdot [B] = k_1 \cdot A_0 \cdot e^{-k_1 \cdot t}$$

This is a differential equation of the general form

$$\frac{dy}{dx} + f(x) \cdot y = g(x)$$

which can be simplified using the integrating factor method. For this, both sides are multiplied by

$$e^{\int f(x)dx} = e^{\int k_2 dt} = e^{k_2 t}$$

to give:

$$e^{k_2 t} \cdot \frac{d[B]}{dt} + e^{k_2 t} \cdot k_2 \cdot [B] = k_1 \cdot A_0 \cdot e^{-k_1 \cdot t} \cdot e^{k_2 t}$$

This is equivalent to

$$\frac{d\left([B] \cdot e^{k_2 t}\right)}{dt} = k_1 \cdot A_0 \cdot e^{(k_2 - k_1) \cdot t}$$

Now an integration from B_0 to B (left side) or 0 to t (right side), respectively, results in

$$[B] \cdot e^{k_2 t} - B_0 \cdot e^0 = \frac{k_1 \cdot A_0}{k_2 - k_1} \cdot \left(e^{(k_2 - k_1) \cdot t} - 1\right)$$

As mentioned earlier, we assume $B_0 = 0$, which results in the following equation 2 for the time dependence of the concentration of compound B.

$$\boldsymbol{[B] = \frac{k_1 \cdot A_0}{k_2 - k_1} \cdot \left(e^{-k_1 \cdot t} - e^{-k_2 \cdot t}\right)} \qquad \textit{(S2)}$$

The concentration of the second intermediate C can finally be described as

$$\frac{d[C]}{dt} = k_1 \cdot [A] + k_2 \cdot [B] - k_3 \cdot [C]$$

If the equations 1 and 1 are inserted into this term it transfers to

$$\frac{d[C]}{dt} + k_3 \cdot [C] = k_1 \cdot A_0 \cdot e^{-k_1 \cdot t} + k_2 \cdot \frac{k_1 \cdot A_0}{k_2 - k_1} \cdot \left(e^{-k_1 \cdot t} - e^{-k_2 \cdot t}\right)$$

Here as described above the integrating factor method is used. It applies that

$$e^{\int f(x)dx} = e^{\int k_3 dt} = e^{k_3 t}$$

leading to the following equation.

$$e^{k_3 t} \cdot \frac{d[C]}{dt} + e^{k_3 t} \cdot k_3 \cdot [C] = k_1 \cdot A_0 \cdot e^{-k_1 \cdot t} \cdot e^{k_3 t} + \frac{k_1 \cdot k_2 \cdot A_0}{k_2 - k_1} \cdot \left(e^{(k_3 - k_1)\cdot t} - e^{(k_3 - k_2)\cdot t}\right)$$

This can be simplified to

$$\frac{d\left([C] \cdot e^{k_3 t}\right)}{dt} = k_1 \cdot A_0 \cdot e^{-k_1 \cdot t} \cdot e^{k_3 t} + \frac{k_1 \cdot k_2 \cdot A_0}{k_2 - k_1} \cdot \left(e^{(k_3 - k_1)\cdot t} - e^{(k_3 - k_2)\cdot t}\right)$$

An integration from C_0 to C (left side) or 0 to t (right side), respectively, while taking into account that $C_0 = 0$, results in

$$[C] \cdot e^{k_3 t} = \frac{k_1 \cdot A_0}{k_2 - k_1} \cdot \left\{ -\frac{k_1 \cdot 2k_2}{k_1 - k_3}\left(e^{(k_3 - k_1)\cdot t} - 1\right) - \frac{k_2}{k_2 - k_3}\left(e^{(k_3 - k_2)\cdot t} - 1\right) \right\}$$

Ultimately equation 3 is obtained.

$$[\boldsymbol{C}] = \frac{\boldsymbol{k_1 \cdot A_0}}{\boldsymbol{k_2 - k_1}} \cdot \left\{ -\frac{\boldsymbol{k_1 \cdot 2k_2}}{\boldsymbol{k_1 - k_3}}\left(\boldsymbol{e^{-k_1 \cdot t} - e^{-k_3 \cdot t}}\right) - \frac{\boldsymbol{k_2}}{\boldsymbol{k_2 - k_3}}\left(\boldsymbol{e^{-k_2 \cdot t} - e^{-k_3 \cdot t}}\right) \right\} \qquad \textit{(S3)}$$

Concentrations profiles

In the following selected concentrations profiles of compounds **1-4** are illustrated upon heating.

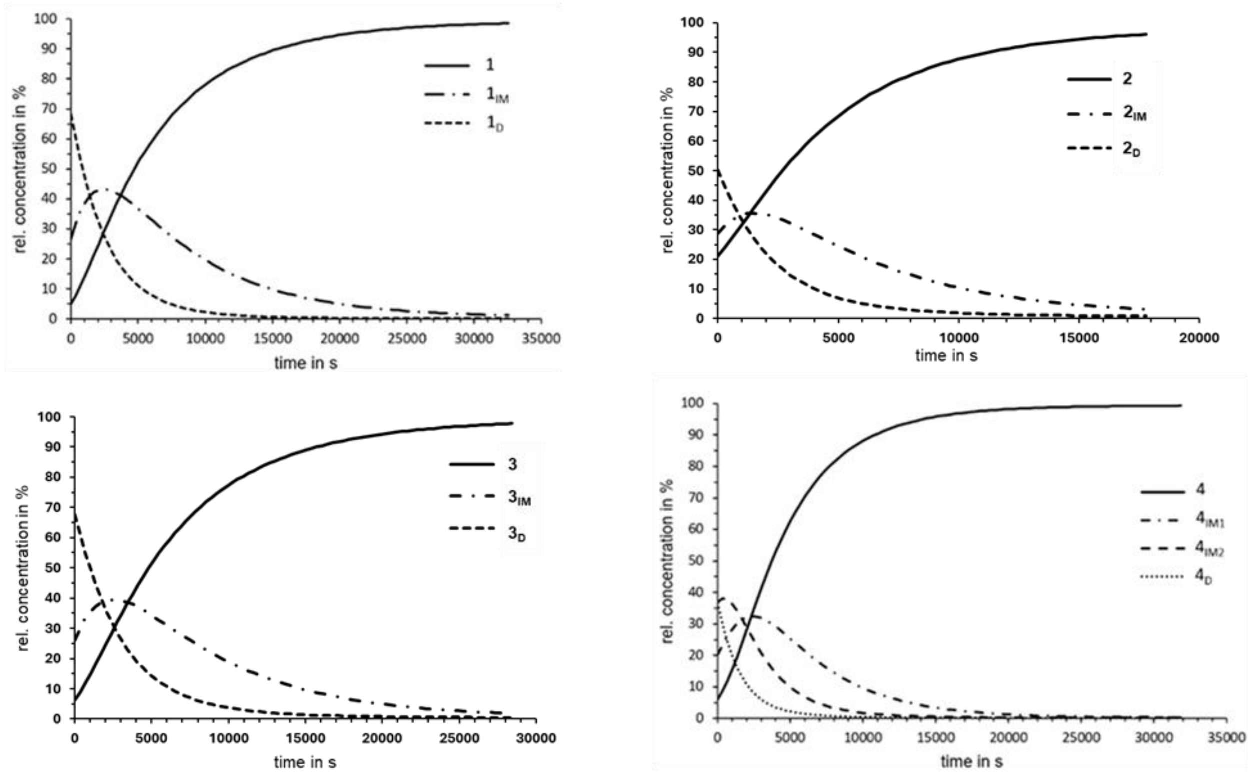

Figure S30. Concentration changes of dihydroazaborinine **1-4**, there corresponding intermediate **1-4**$_{\mathbf{IM}}$ and *Dewar* isomer **1-4**$_{\mathbf{D}}$ upon constant heating upon heating. **1** at 338 K for 10 h (left top), **2** at 338 K for 5 h (top right), **3** at 335 K for 8 h (bottom left) and **4** at 338 K for 9 h (bottom right).

6. Catalytic ring opening

Catalytic back conversion of 6

The catalytic cycloreversion of **6** was investigated utilising Wilkinson's catalyst and several Lewis acids known to be active for similar systems.[6, 8] Wilkinson's catalyst proved to be non-suiTable S, as mainly decomposition of the Dewar isomer was observed *via* ^{1}H NMR (**Fehler! Verweisquelle konnte nicht gefunden werden.**). In contrast, Lewis acidic catalysts proved to be a viable alternative. Especially Ag^{+} salts with weakly coordinating anions were active. Hereby, $Ag[Al(OC(CF_3)_3]_4$ enabled full conversion after 36 h at a catalyst loading of 10 mol% at room temperature without observable decomposition *via* ^{1}H NMR.

Table S3. Catalyst screening for the cycloreversion of 6 in C6D6 at room temperature.

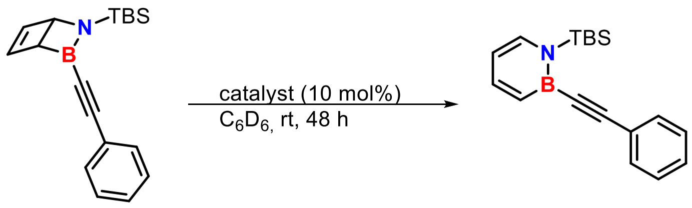

6Dewar

6

entry	catalyst	loading (mol%)	time (h)	conversion (%)
**1**	$Rh(PPh_3)_3Cl$	10	24	decomposition
**2**	$PdCl_2(MeCN)_2$	100	120	*quant.*, minor decomposition
**3**	$Cu(C_6H_5F)_{0.6}[Al(OC(CF_3)_3)_4]$	10	120	23
**4**	$AgSbF_6$	10	46	51
**5**	$Ag[Al(OC(CF_3)_3)_4]$	10	36	*quant.*
**6**	AuCl	50	120	78, slight decomposition

Reaction conditions: $\mathbf{6}_{\mathbf{Dewar}}$ (entry **1**: 27 µmol; entry **2**: 15 µmol; entry **3**: 17 µmol; entry **4**: 29 µmol; entry **5**: 17 µmol; entry **6**: 21 µmol;) in 0.4 mL C_6D_6.

Catalytic back conversion of 3

As $Ag[Al(OC(CF_3)_3]_4$ showed promising activity for the monomeric **3** it was also investigated for the dyad **3** (4.5 µmol in C_6D_6). Hereby, 10 mol% $Ag[Al(OC(CF_3)_3]_4$ at room temperature within 48 h reached full conversion of **3**. While this cycloreversion was accompanied by decomposition, it generally shows, that a Lewis acidic catalytic system could be optimized.

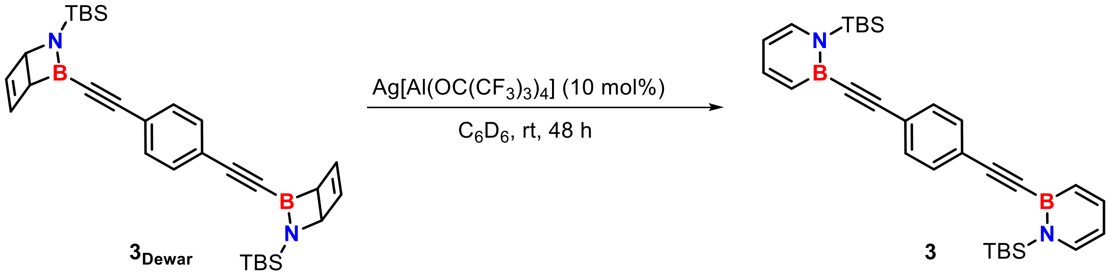

3_{Dewar} 3

full converson, 33% nmr yield

7. Cyclability

As the same sample was used for the kinetic experiments, without interim processing, the experimets provide insight into the stability over four isomerization cycles. No internal standard was added for the kinetic experiments. In case of the ortho dyad **1** neigther the signals of the dihydroazaborinin, the intermediate $\mathbf{1}_{\mathbf{IM}}$ nor the signals of $\mathbf{1}_{\mathbf{Dewar}}$ overlap with the residual solvent signal of benzene. Therefore this signal was utilized as a reference for the integration. As shown in Table S S2 and Figure S S31 with this approach the decomposition per full isomerization cycle to the Dewar and back to the dihydroazaborinin can be estimated to 0.75% for compound **1**.

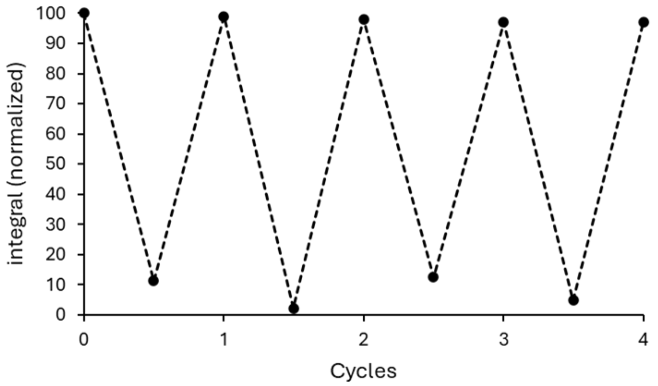

Figure S31. Normalized integrals of dihydroazaborinine **1** with reference to the residual solvent signal of C_6D_6. One cycle includes irradiation (photoisomerization) and thermal backreaction.

Table S4. Absolute values of the normalized integrals of dihydroazaborinin **1** with reference to the residual solvent signal of deuterated benzene as well as the temperature in the heating step

Cycle	norm. int. **1**	comment
0	100	starting material
1	11	irradiated for 30 min
	99	heated to 348 K
2	2	irradiated for 30 min
	98	heated to 338 K
3	12	irradiated for 30 min
	97	heated to 343 K
4	5	irradiated for 30 min
	97	heated to 333 K

For the compounds **2-4** and **6** it is qualitatively apparent that only minor decomposition is observed in four cycles. A comparison with the residual solvent signal is not possible in these cases because signals overlap with the solvents one. Therefore, no quantitative evaluation can be performed here. However, only very small new signals appear in silyl region of the NMR spectrum. Additionally, since no cloudiness or precipitation is observed in the solution, it can be assumed that potential impurities or decomposition products remain in solution and can be detected by NMR. If larger parts of the sample decompose, this should therefore lead to noticeable new signals.

8. DSC measurements

Sample preparation

In separate quartz J. Young the dihydroazaborinines **1-4** and **6** were weight in using a fine scale, with an accuracy of 0.01 mg. These NMR tubes were then transferred to a glovebox and non-deuterated mesitylene was added via a *Hamilton* syringe. The exact masses and volumes used can be found in **Table S5**. Each sample was irradiated with UV light of 280-400 nm and the conversion was qualitatively judged *via* ^{1}H and ^{11}B NMR spectroscopy. The tubes were again transferred to a glovebox. With an Eppendorf pipette 10 to 20 µL of this solution were transferred to pre-weighted aluminium crucibles. The crucibles were sealed with, also pre-weighted, concave lids in the glovebox. Usually, the curvature of the lid faces downward to ensure that the lid can be placed easily and perfectly centered on the crucible, preventing it from slipping before pressing. Since the crucibles had a relatively high fill level, capillary effects caused some solution to be drawn between the crucible and the lid or even completely out. As a consequence, such crucibles were often not airtight. Therefore, the lids were always placed with the curvature facing upward, keeping the contact surface between the crucible and the lid clean and ensuring that the capsules were properly sealed. The amount of solution was cross-checked by weighing on the fine scale. To about 0.1 mL of the mesitylene solution DCM-$d_2$was added, proton and ^{11}B NMR spectra were recorded and out of this data the conversion of the photoisomerization was determined.

Measurements

In each measurement a temperature of -20 °C was held for 10 minutes before heating. The isomers **1**, **2** and compound **4** were then heated up to 160 °C with a rate of 1 K/min. This temperature was held for 20 minutes, reduced back to -20 °C (rate: 1 K/min) and held for 10 minutes. To ensure that the energy release is complete each sample was then heated up a second time to 160 °C.

For reference compound **6** the maximum temperature was set to 140 °C due to boiling of the compound at higher temperatures. The results of these experiments are summarized in **Table S5**.

Table S5. Experimental data according to the sample preparation, results of the DSC measurement as well as corrected stored energy with respect to the conversion.

	M g/mol	m mg	V(solvent) mL	c(solution) g/mL	con %		m mg	m mg	ΔH_{exp} kJ/kg	ΔH_{corr} kcal/mol
**6** (*mono*)	293.23	10.63	0.419	0.025	98	1	50.13	0.39	788.1	56.36
						2	50.90	0.26	690.9	49.41
						3	50.50	0.39	676.2	48.36
						4	50.93	0.26	750.5	53.67
										**51.95**
**1** (*ortho*)	508.47	9.10	0.422	0.022	90	1	50.61	0.55	731.7	98.80
						2	50.84	0.61	666.9	90.05
						3	49.75	0.63	715.7	96.64
						4	50.09	0.55	745.6	100.68
										**96.54**
**2** (*meta*)	508.47	8.49	0.430	0.054	90	1	49.99	1.58	804.9	108.69
						2	51.22	1.58	750.8	101.38
						4	50.38	1.36	843.8	113.94
						5[1]	50.05	1.58	745.2	100.62
										**106.16**
**4**	723.65	8.42	0.525	0.081	85	1	50.86		1015.0	184.79
						2	51.47		631.9	115.05
						3	50.85		1008.0	183.52
						4	49.94		912.0	166.04
						5	50.10		659.1	119.99
										**153.88**

[1] Crucible opened during measurement, but after the heat release was completed

DSC no. 1 – compound **6**

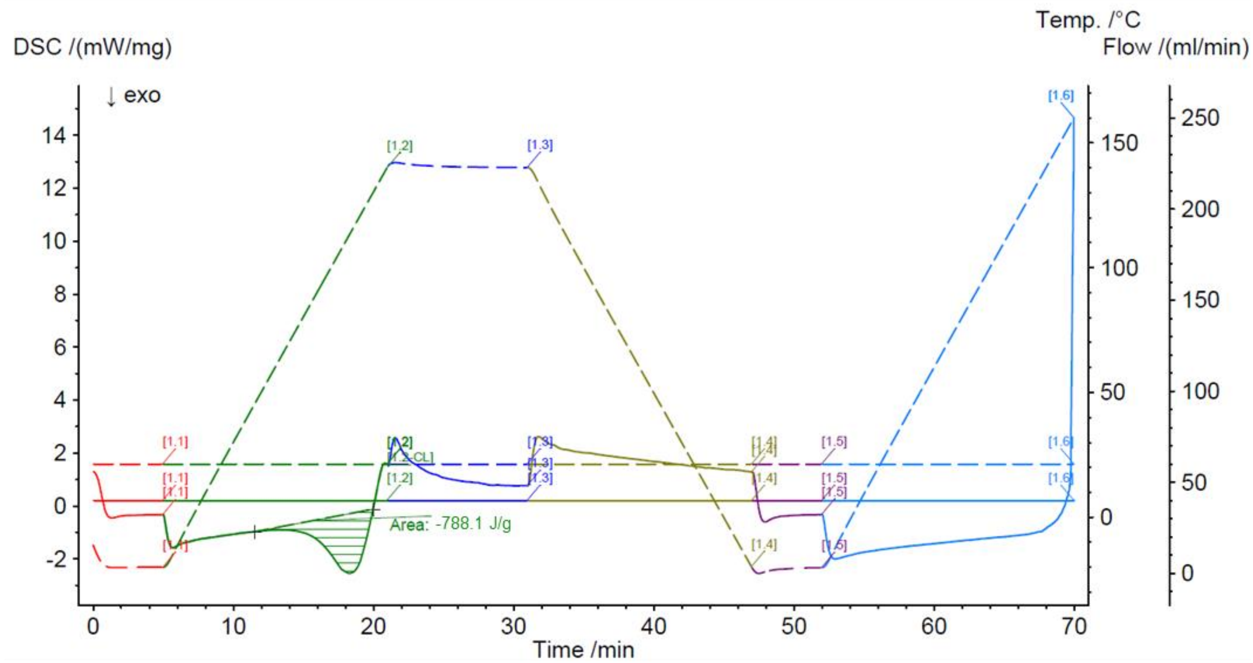

DSC no. 2 – compound **6**

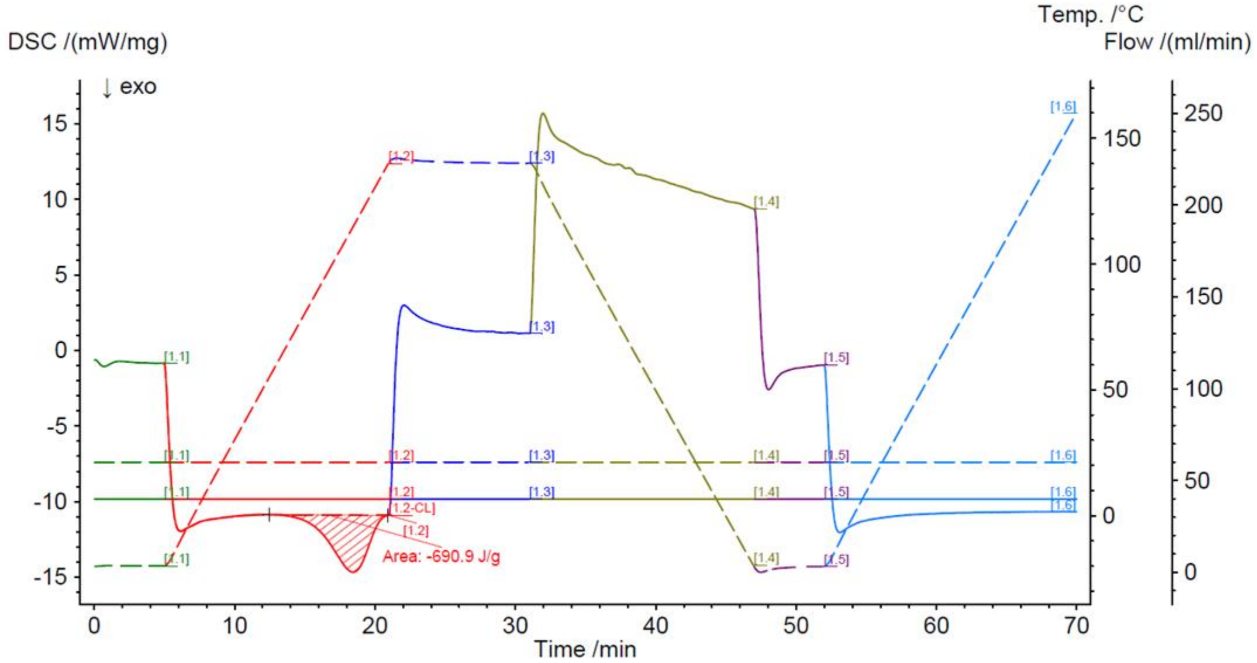

Figure S32. DSC measurements of compound **6**. The area under the exothermal reaction peak corresponds to the released energy.

Figure S32 (continued)

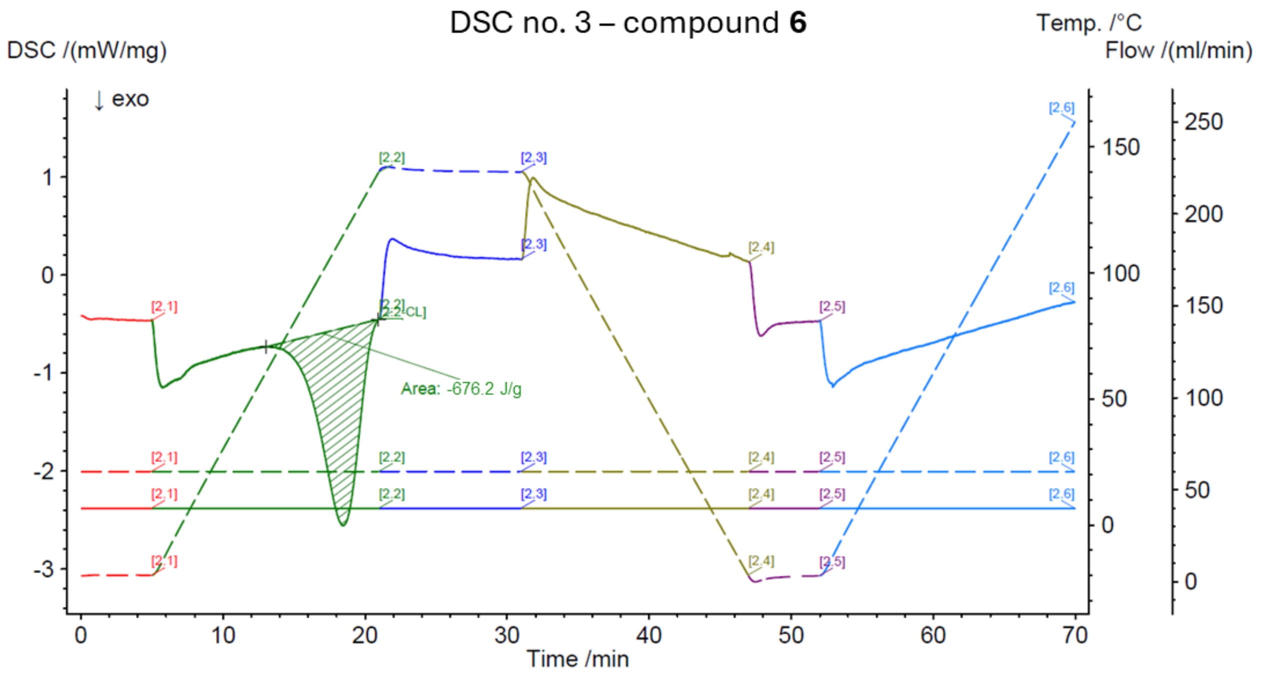

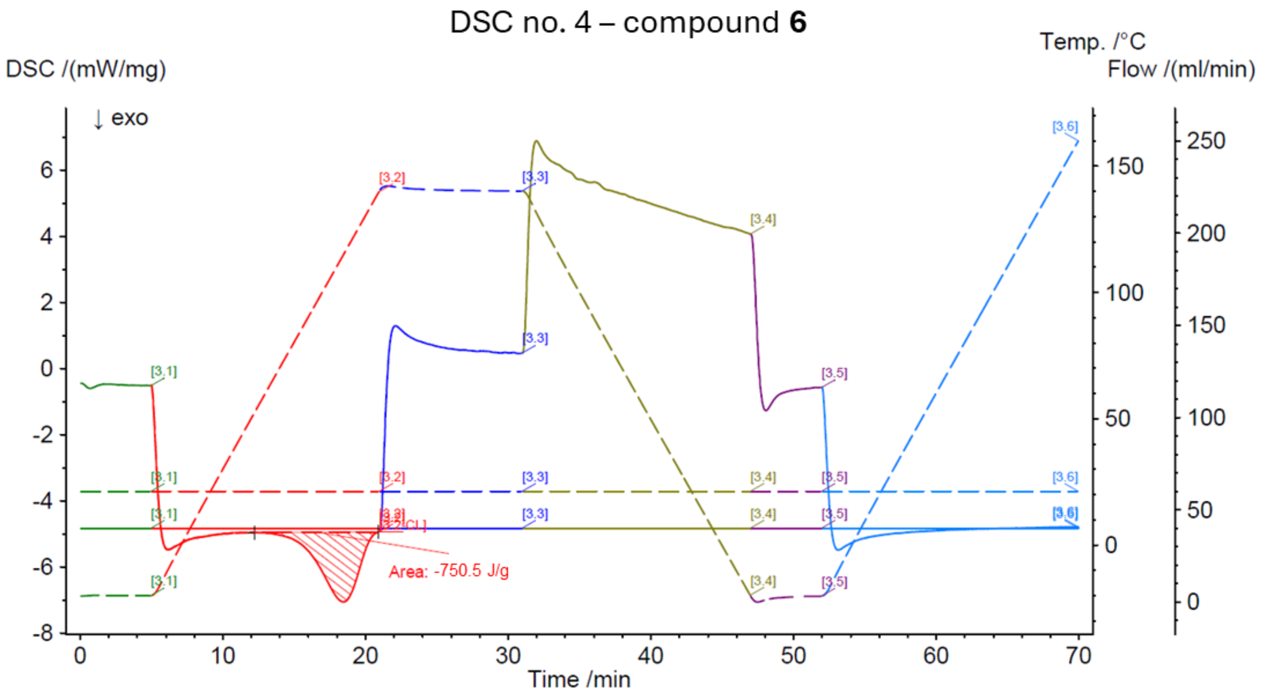

DSC no. 1 – compound **1**

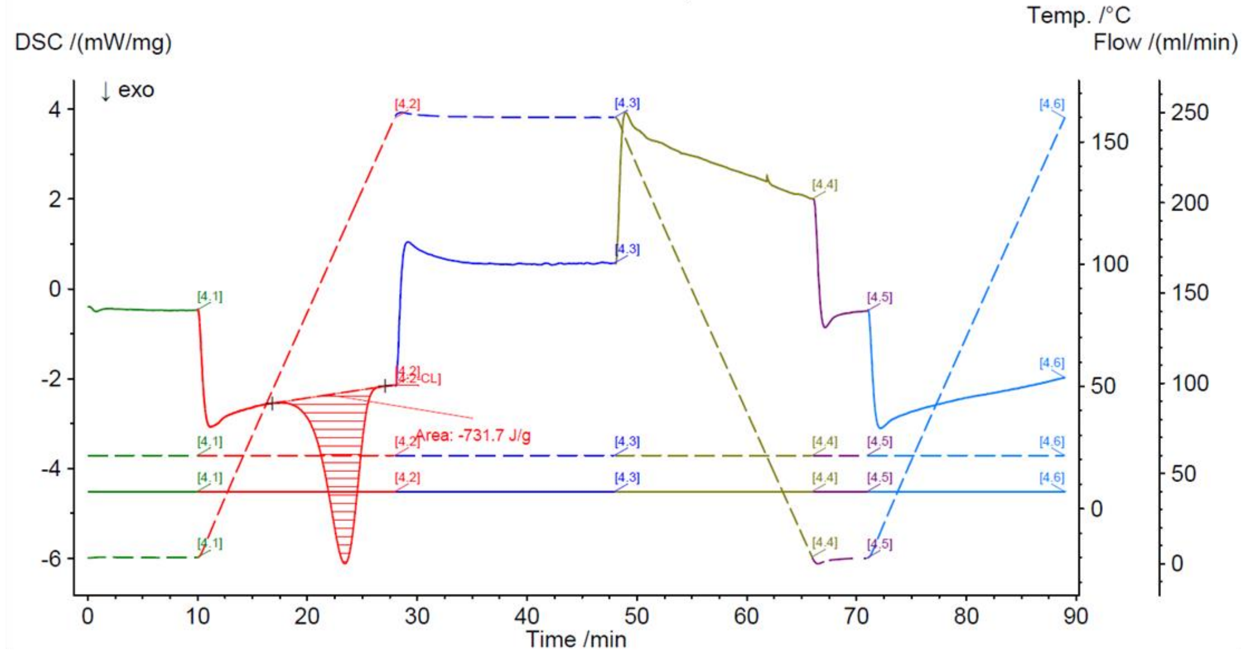

DSC no. 2 – compound **1**

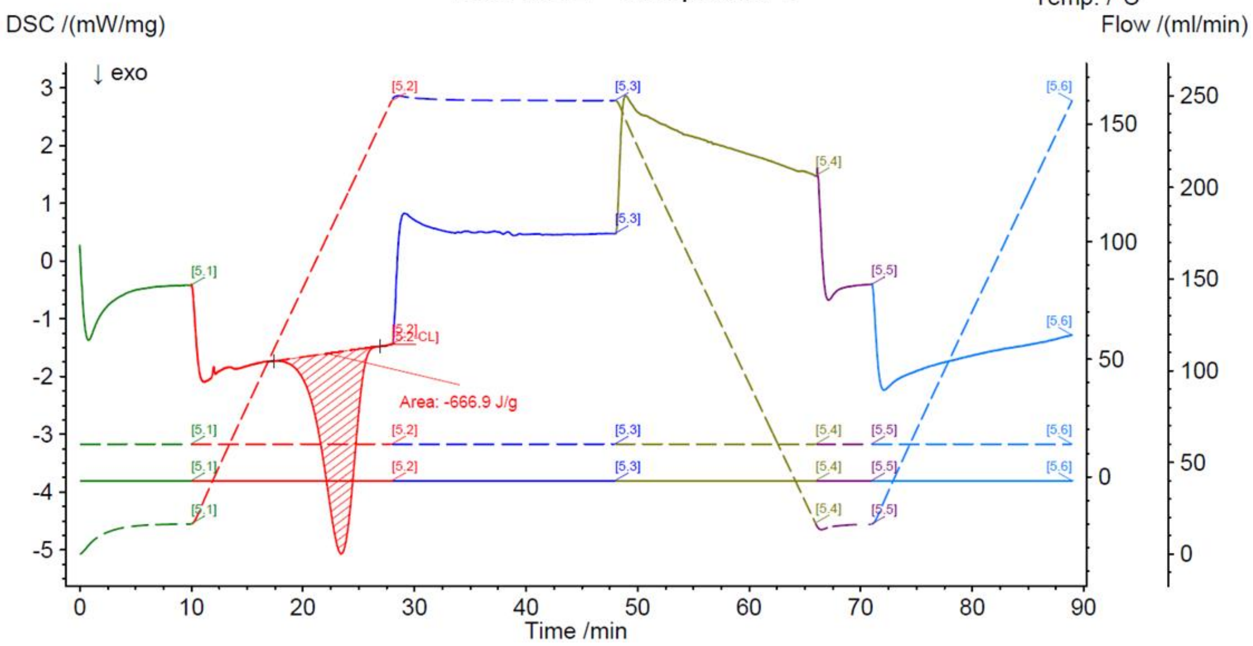

Figure S33. DSC measurements of compound **1**. The area under the exothermal reaction peak corresponds to the released energy.

Figure S33. (continued)

DSC no. 3 – compound **1**

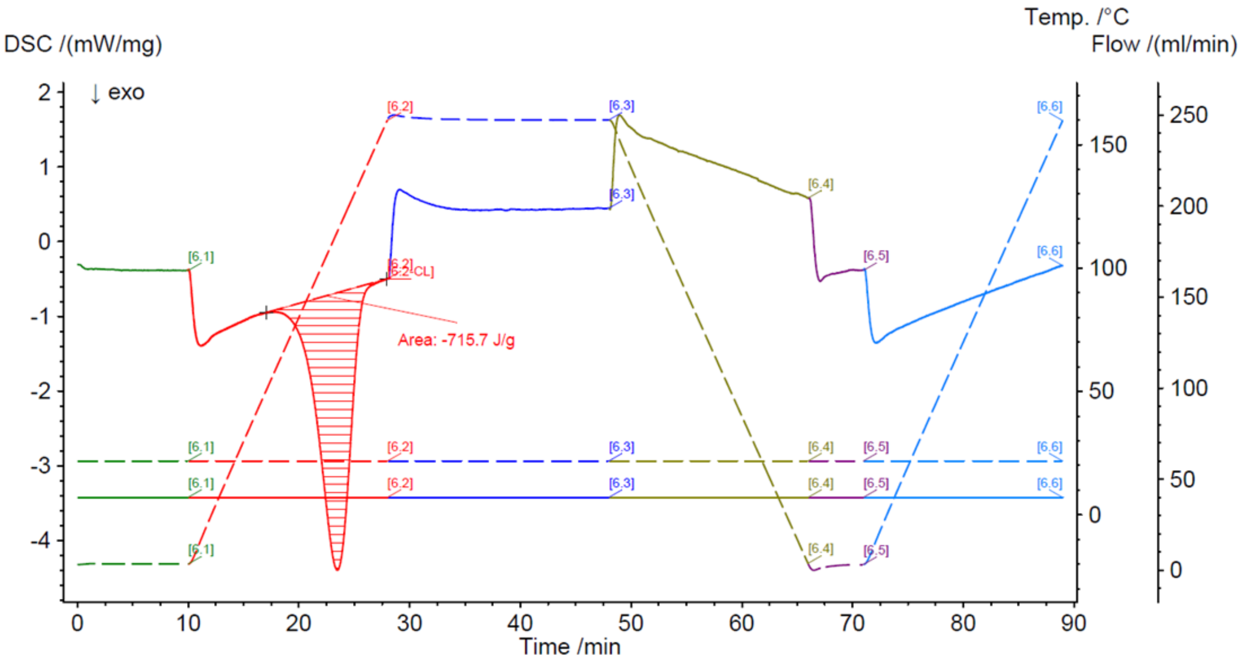

DSC no. 4 – compound **1**

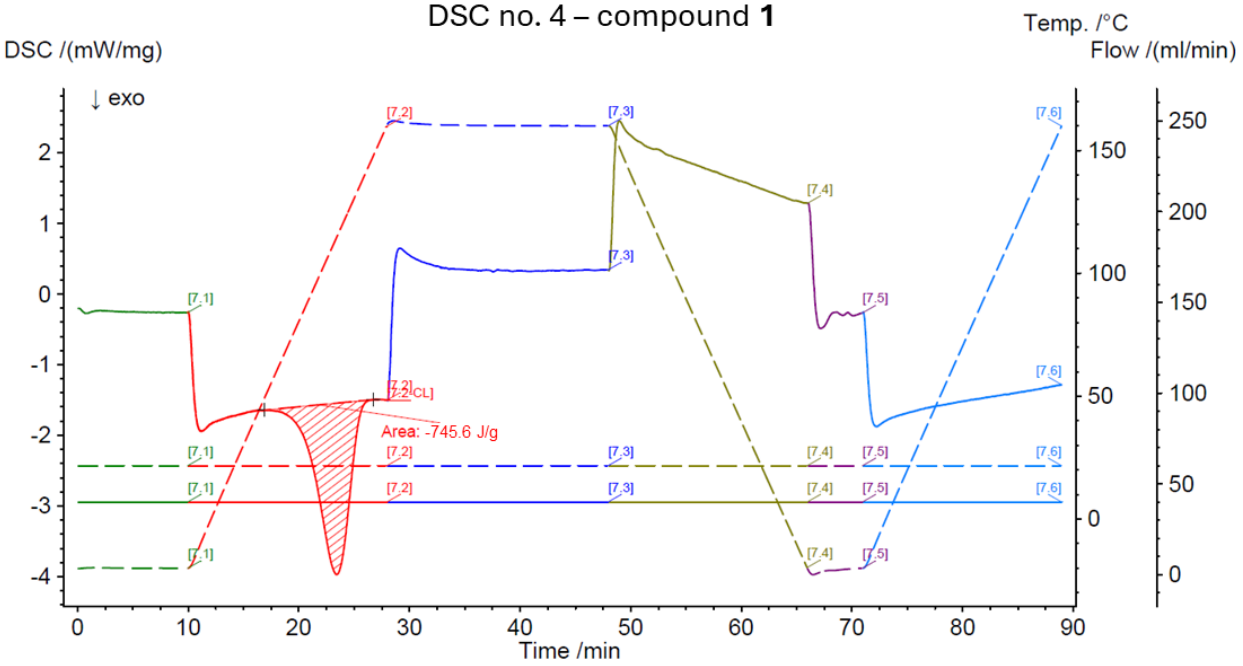

DSC no. 1 – compound **2**

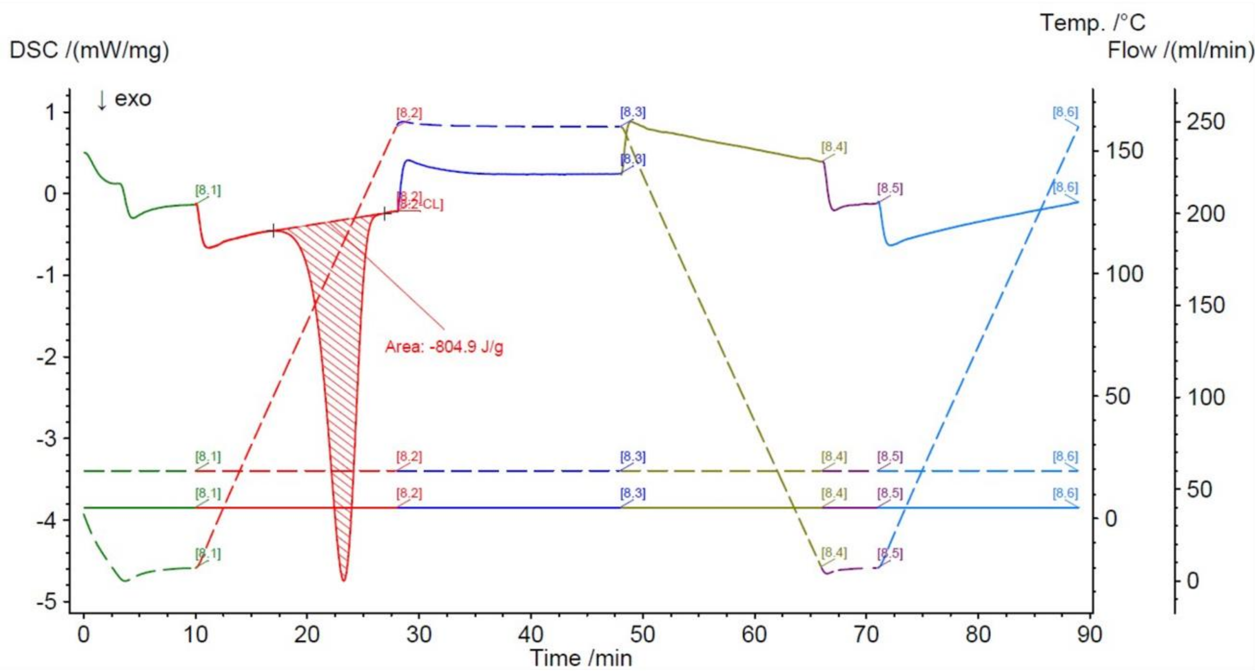

DSC no. 2 – compound **2**

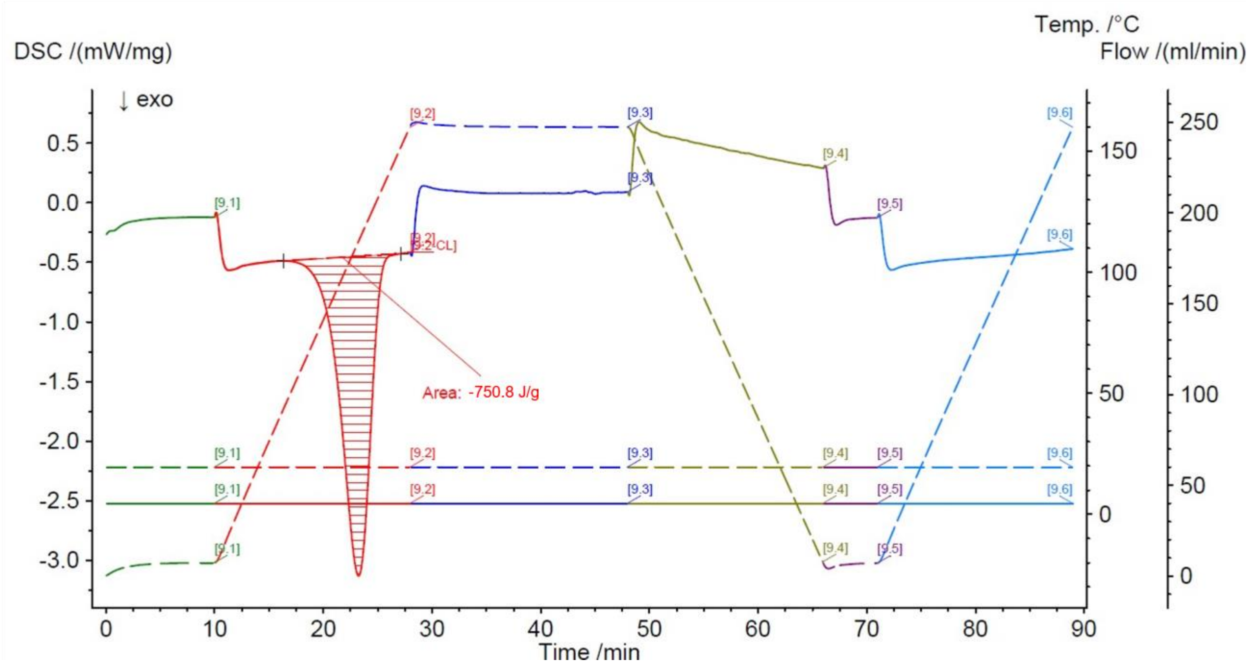

Figure S34. DSC measurements of compound **2**. The area under the exothermal reaction peak corresponds to the released energy.

Figure S34. (continued)

DSC no. 3 – compound **2**

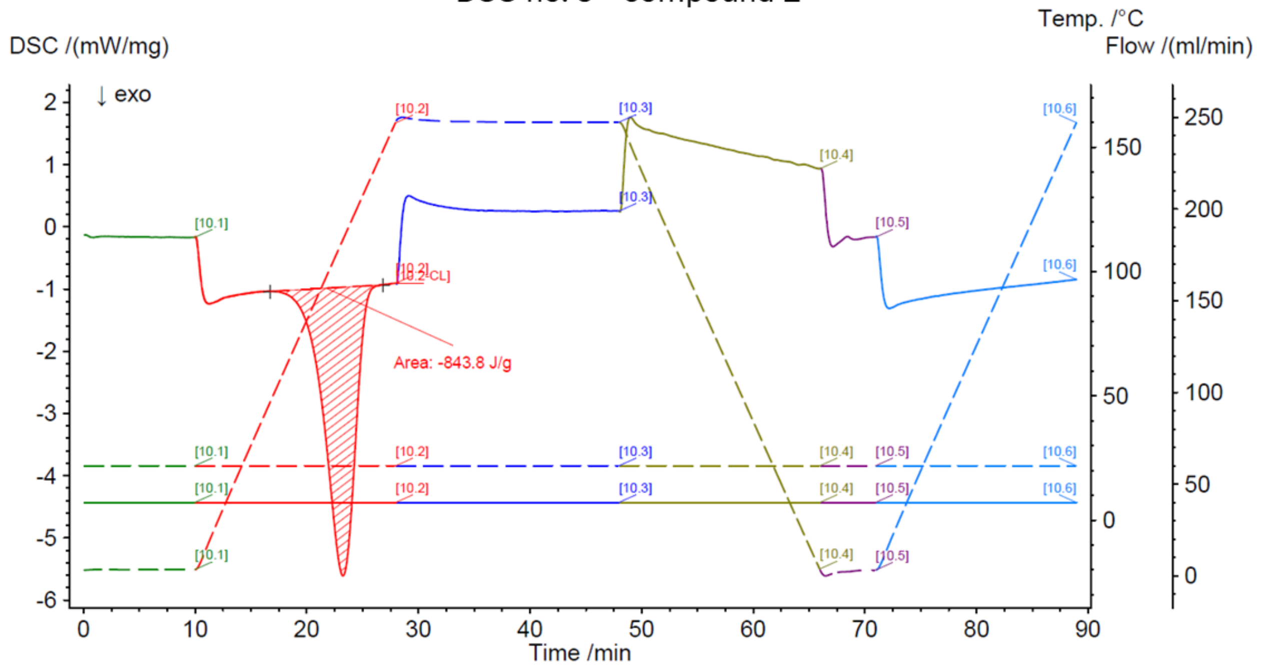

DSC no. 4 – compound **2**

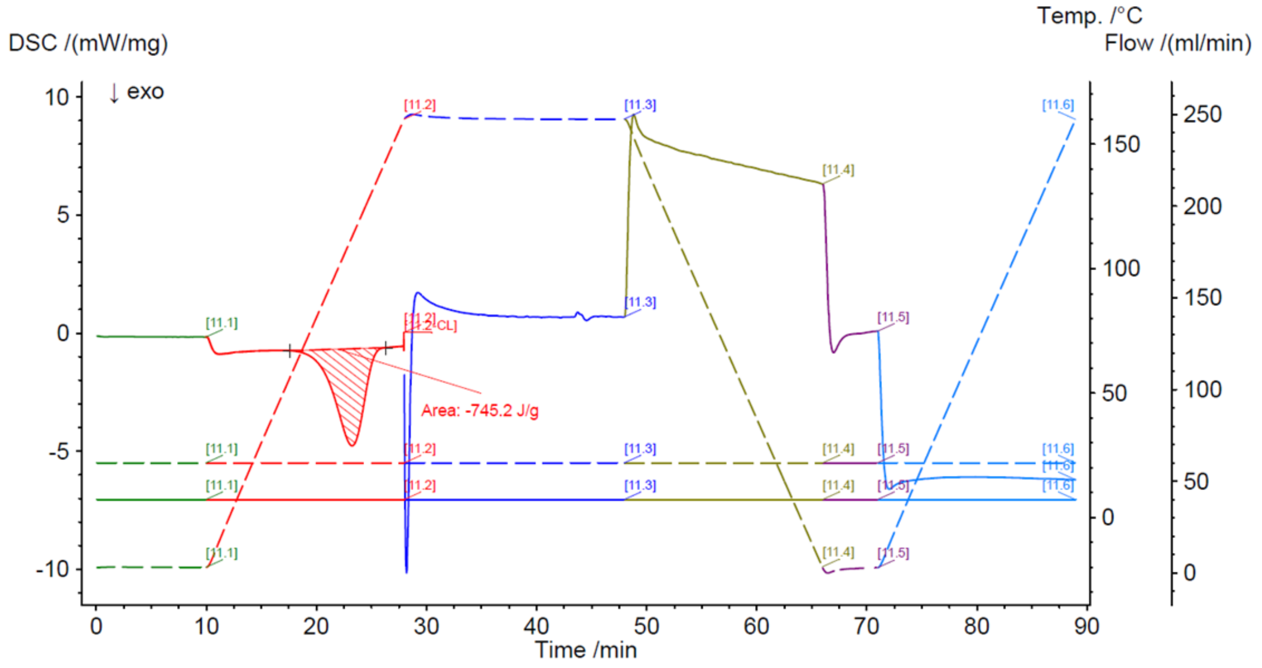

DSC no. 1 – compound **4**

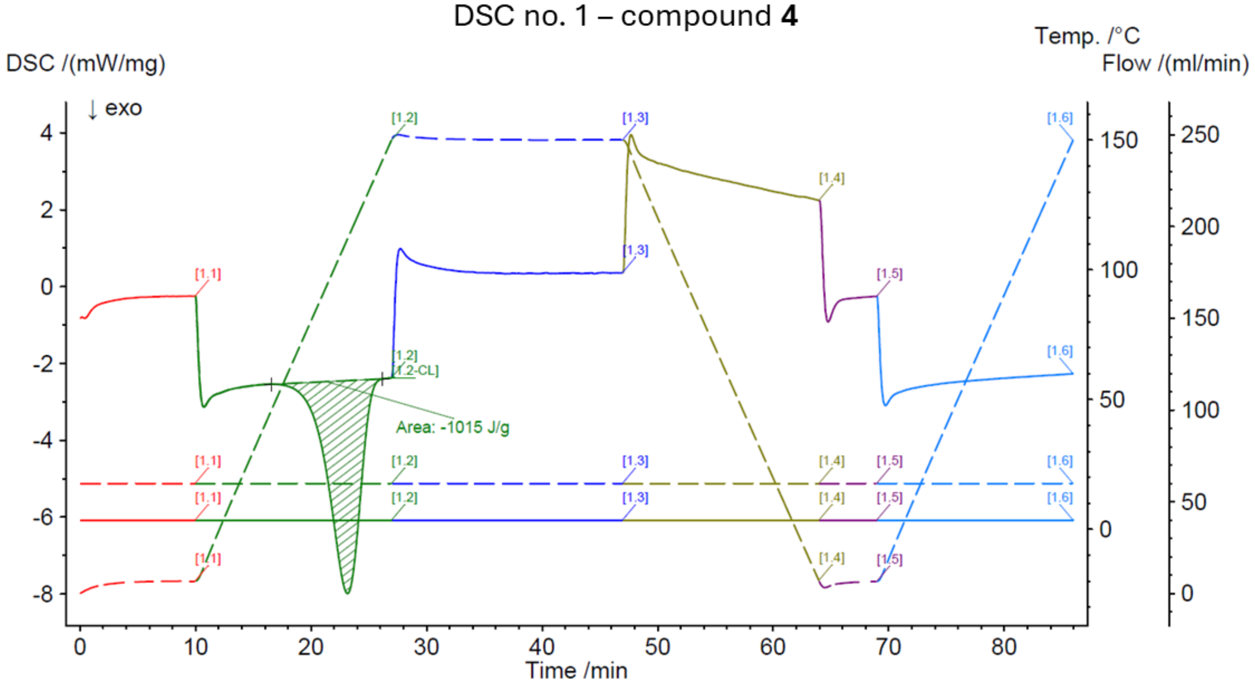

DSC no. 2 – compound **4**

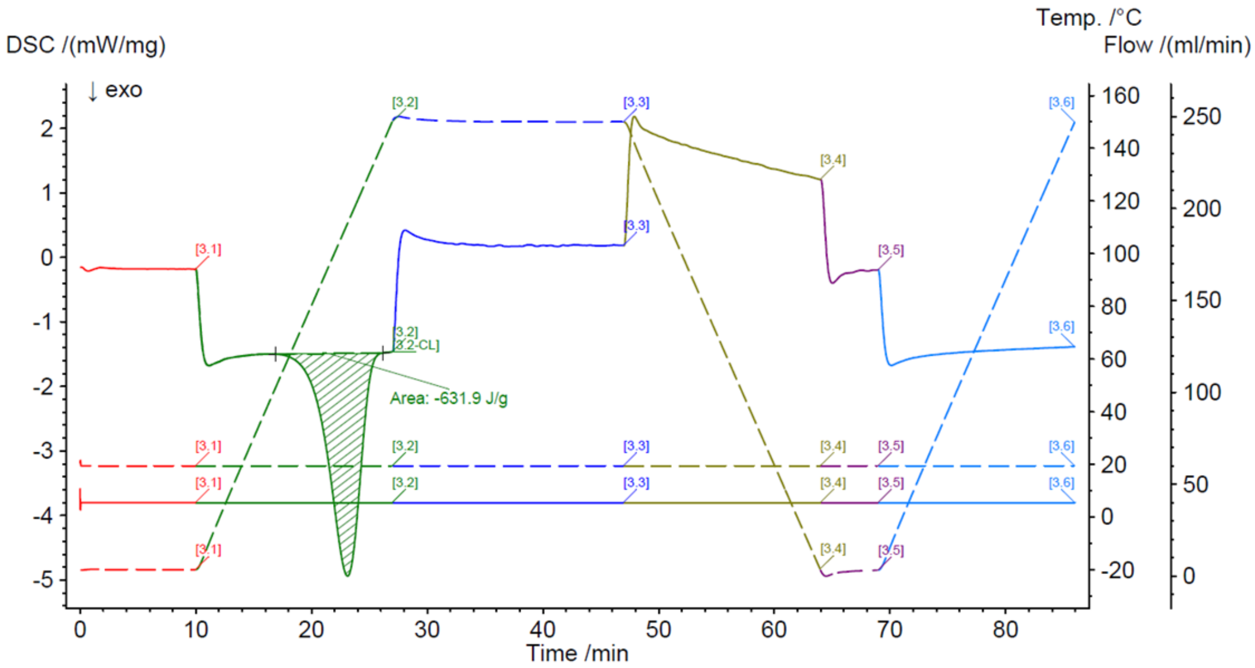

Figure S35. DSC measurements of compound **4**. The area under the exothermal reaction peak corresponds to the released energy.

Figure S35. (continued)

DSC no. 3 – compound **4**

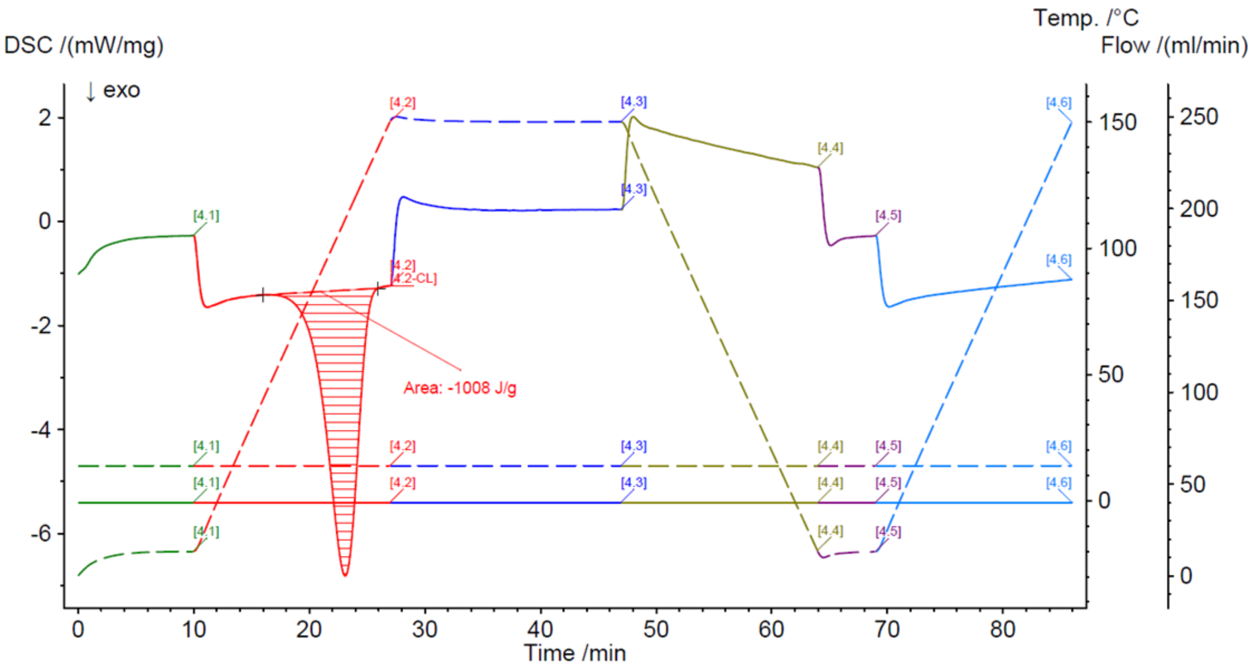

DSC no. 4 – compound **4**

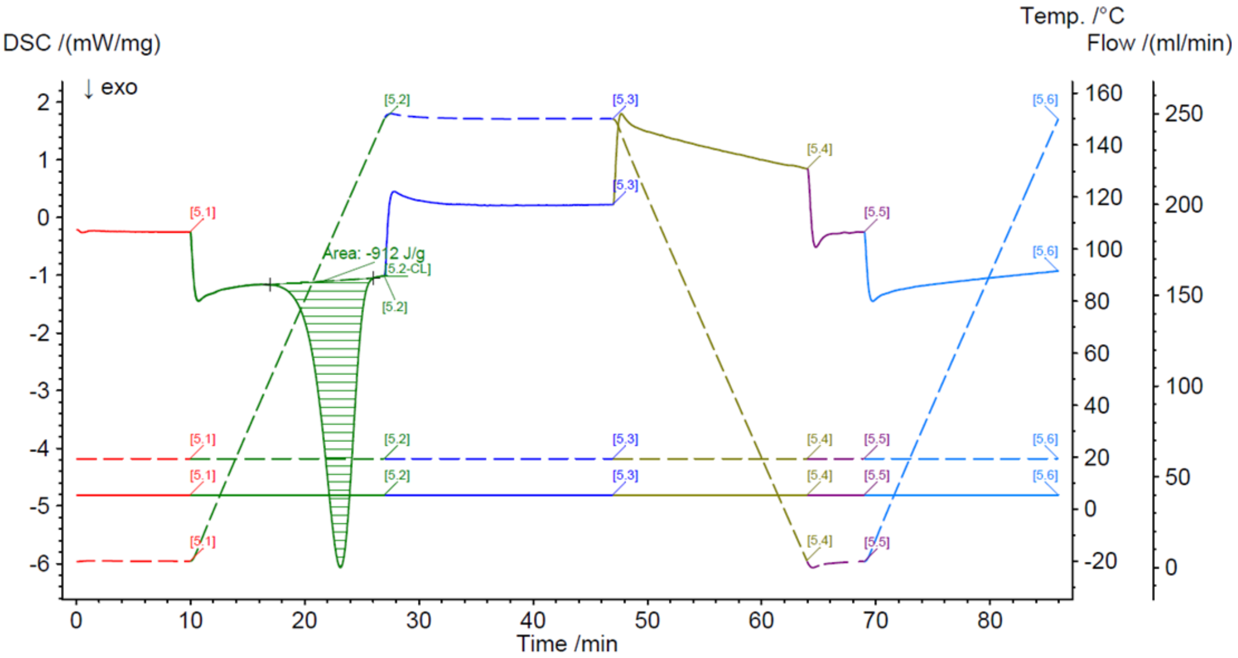

Figure S35. (continued)

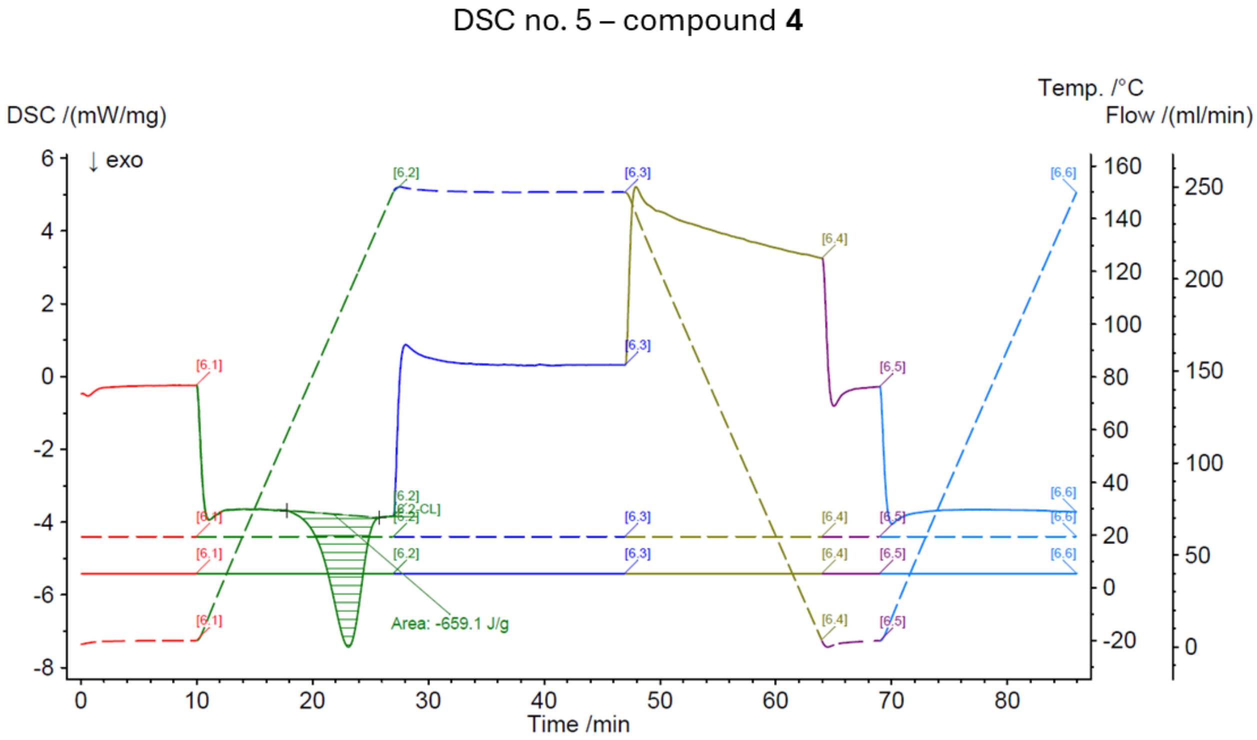

9. UV-Vis spectra

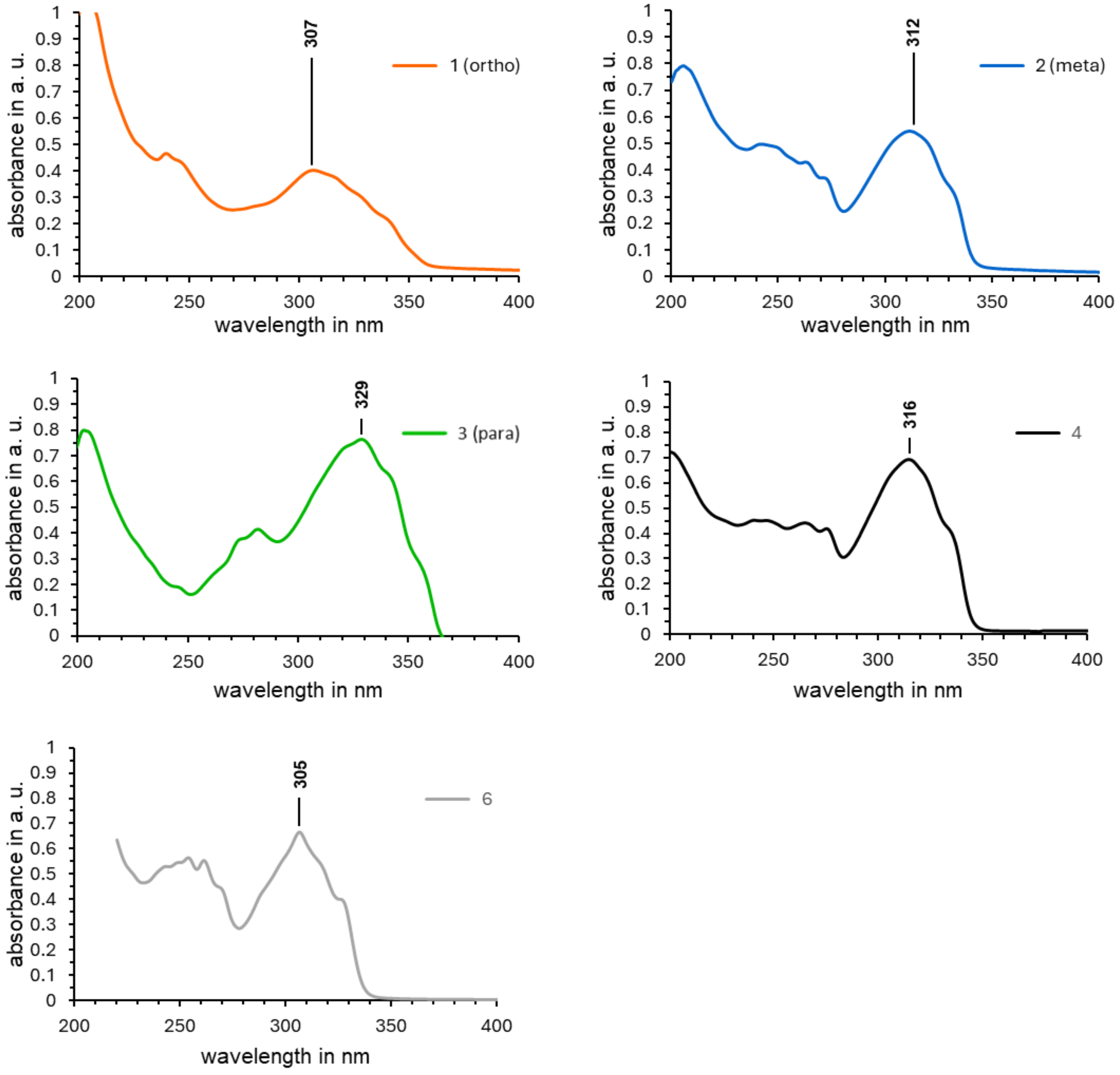

Figure S36. UV-Vis spectra of 1-4 and 6 measured in cyclohexane from 200-400 nm. The absorption maxima are labeled.

10. Crystal structures

Refinement details

The implementation NoSpherA2 for non-spherical atom form factors in Olex2 makes use of tailor made aspherical atomic form factors calculated from a Hirshfeld-partitioned electron density (ED), not from spherical atom form factors.[9] The electron density is calculated from a Gaussian basis set single determinant SCF wavefunction for a fragment of the crystal. This fragment can be embedded in an electrostatic crystal field by employing cluster charges or modelled using implicit solvation models, depending on the software used. This was computed using M062X/6-31G(d,p)[10-13], normal integration accuracy, a charge of 0 and a multiplicity of 1 with Orca 5.0.[14]

Table S6. Parameters and results of the X-ray diffraction measurement of compound 3 and 4.

Parameter	unit	3	4
Molecular formula		$C_{30}H_{42}B_2N_2Si_2$	$C_{42}H_{60}B_3N_3Si_3$
CCDC		2253439	2240655
$D_{calc.}$	g cm^{-3}	1.109	1.040
μ	mm^{-1}	1.194	1.157
Molar weight	g/mol	508.501	723.694
Colour		clear colourless	clear colourless
Shape		block-shaped	block-shaped
Size	mm^3	0.14×0.13×0.05	0.19×0.12×0.10
T/K		176(30)	150.00(10)
Crystal System		orthorhombic	triclinic
Space Group		*Pbca*	*P*-1
a/Å		9.0863(1)	11.9415(1)
b/Å		17.0451(2)	13.5799(1)
c/Å		19.6663(2)	17.4660(2)
α/°		90	67.299(1)
β/°		90	89.713(1)
γ/°		90	64.393(1)
V	$Å^3$	3045.86(6)	2311.31(5)
Z		4	2
Z'		0.5	1
Wavelength	Å	1.54184	1.54184
Radiation type		Cu K_a	Cu K□
Θ_{min}	°	4.50	2.80
Θ_{max}	°	78.69	80.00
Measured Refl's.		84319	102658
Indep't Refl's		3217	9972
Refl's I≥2 σ(I)		3063	9768
R_{int}		0.0230	0.0185
Parameters		352	1000
Restraints		0	0
Largest Peak		0.1055	0.5660
Deepest Hole		-0.0708	-0.2385
GooF		1.1088	1.0861
wR_2 (all data)		0.0382	0.0433
wR_2		0.0377	0.0430
R_1 (all data)		0.0156	0.0195
R_1		0.0144	0.0191

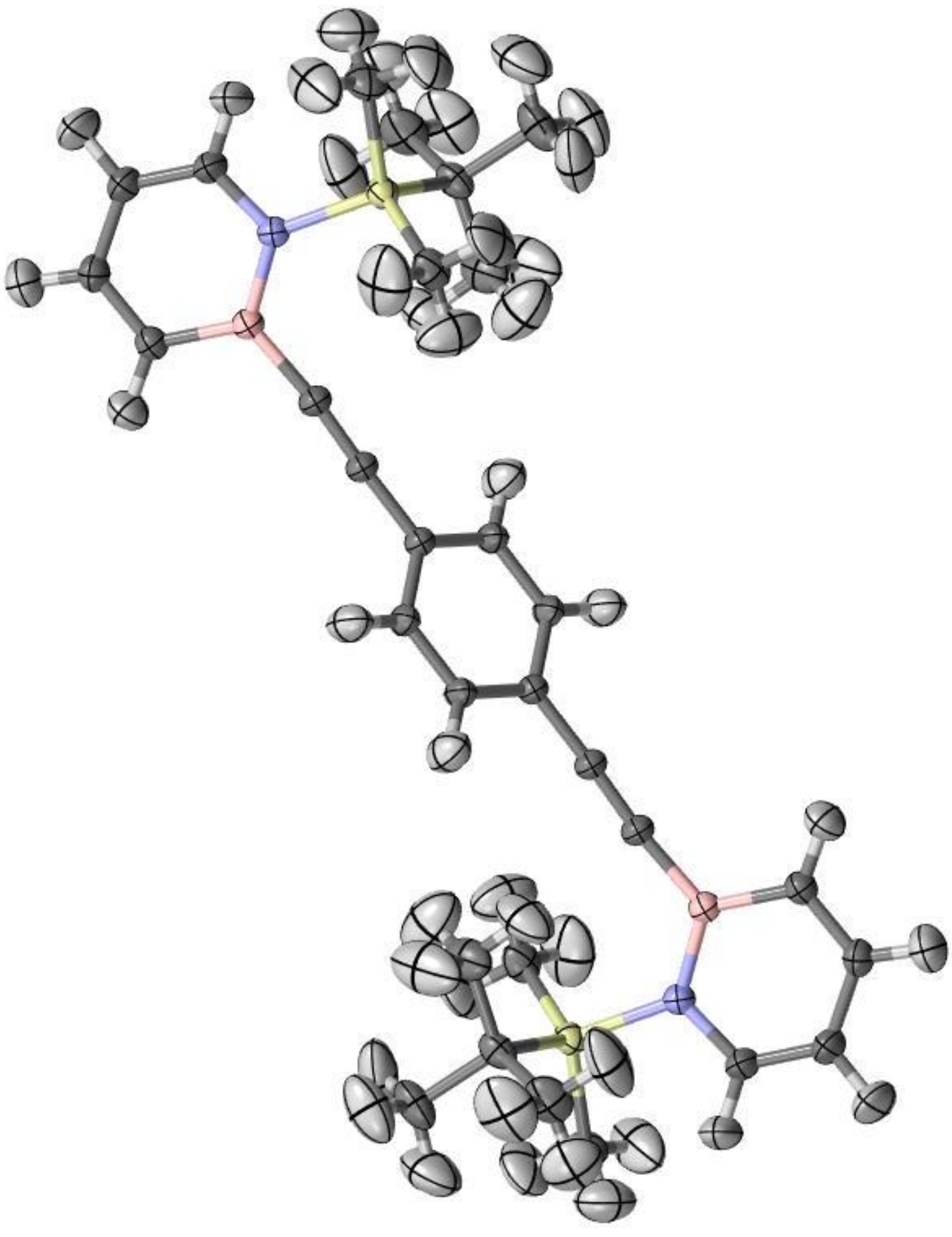

Figure S37. Crystal structure of **3**. Thermal ellipsoids are drawn at the 50% probability level.

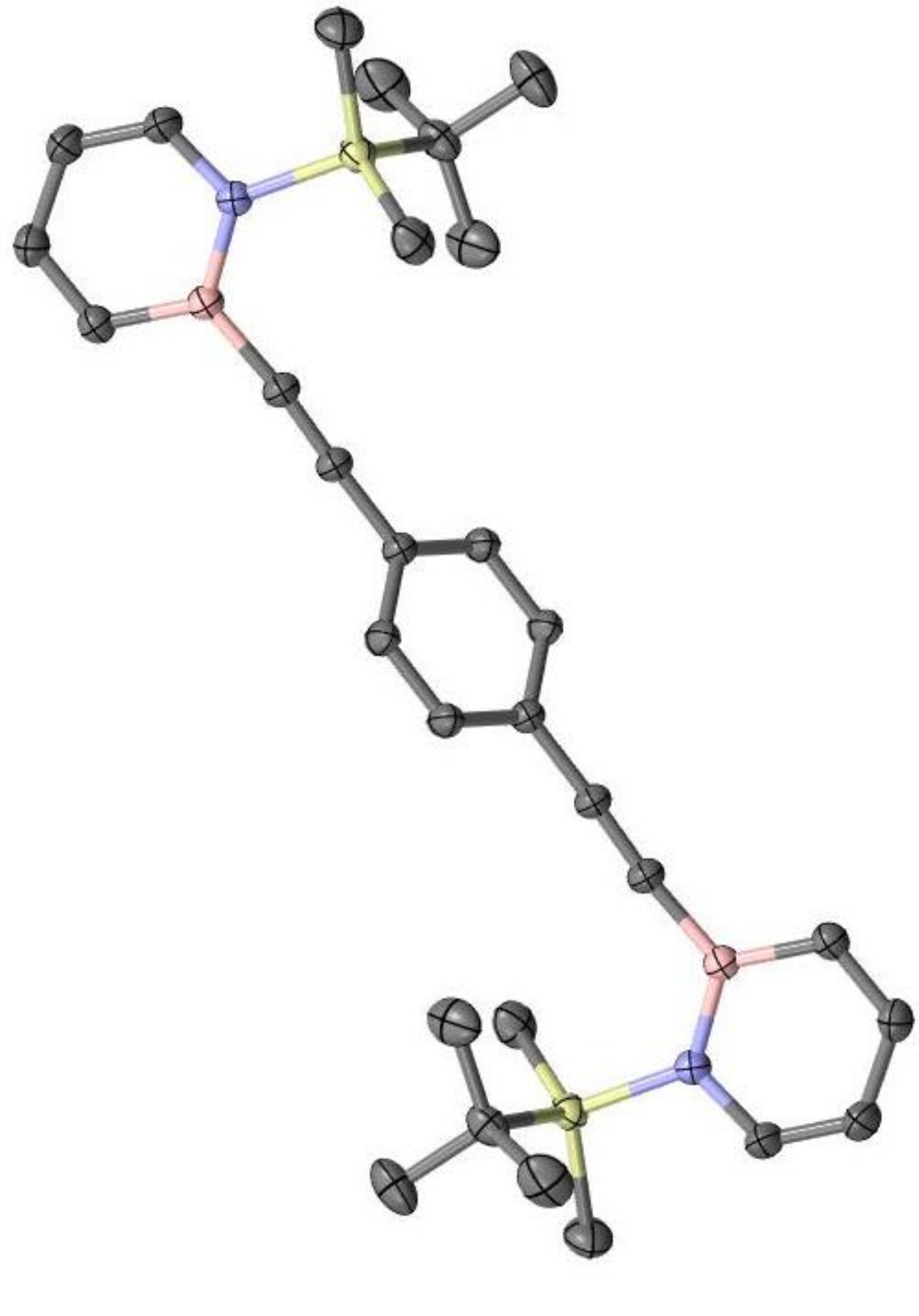

Figure S38. Crystal structure of **3**. Hydrogens are omitted for clarity and thermal ellipsoids are drawn at the 50% probability level.

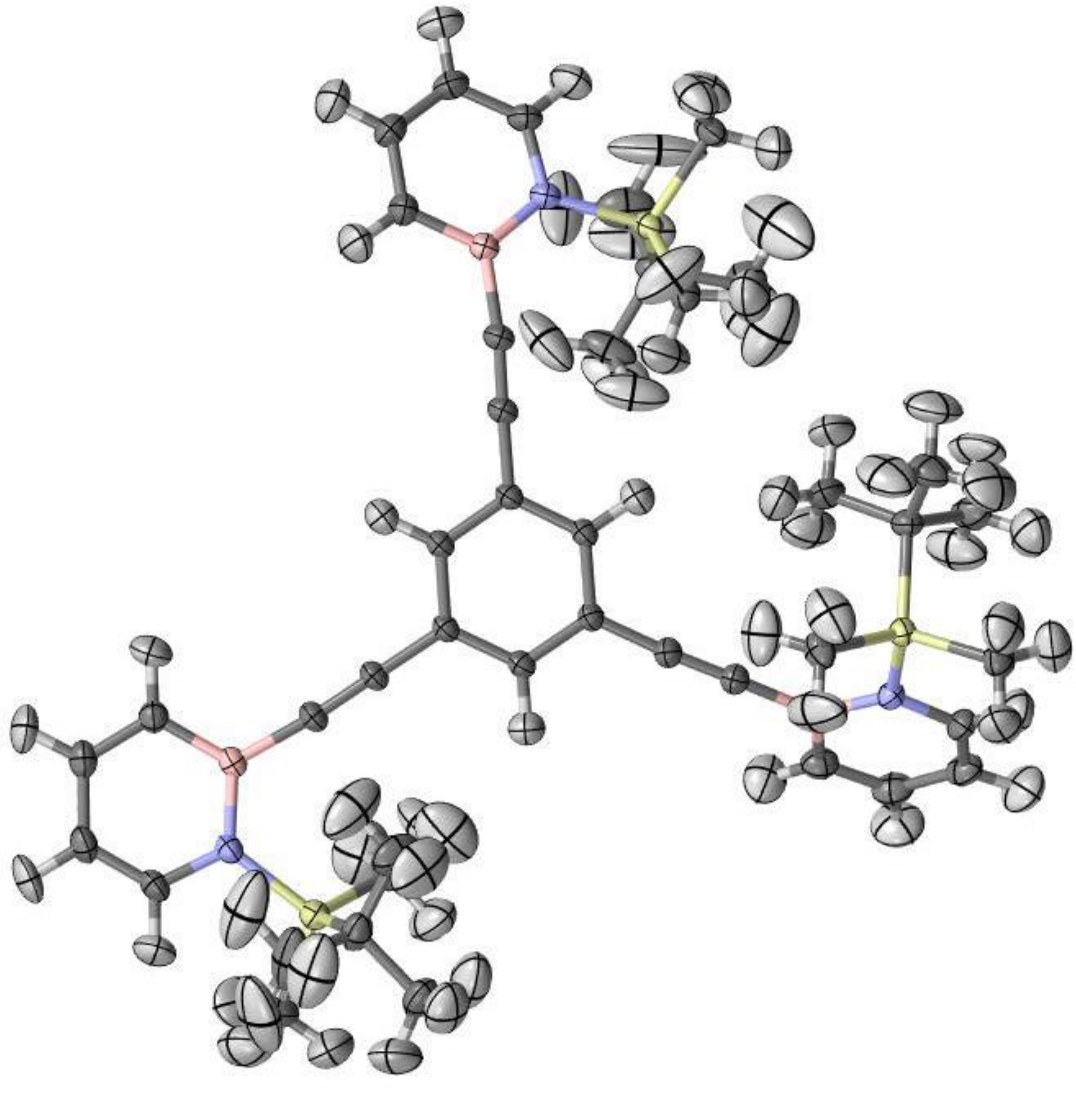

Figure S39. Crystal structure of **4**. Thermal ellipsoids are drawn at the 50% probability level.

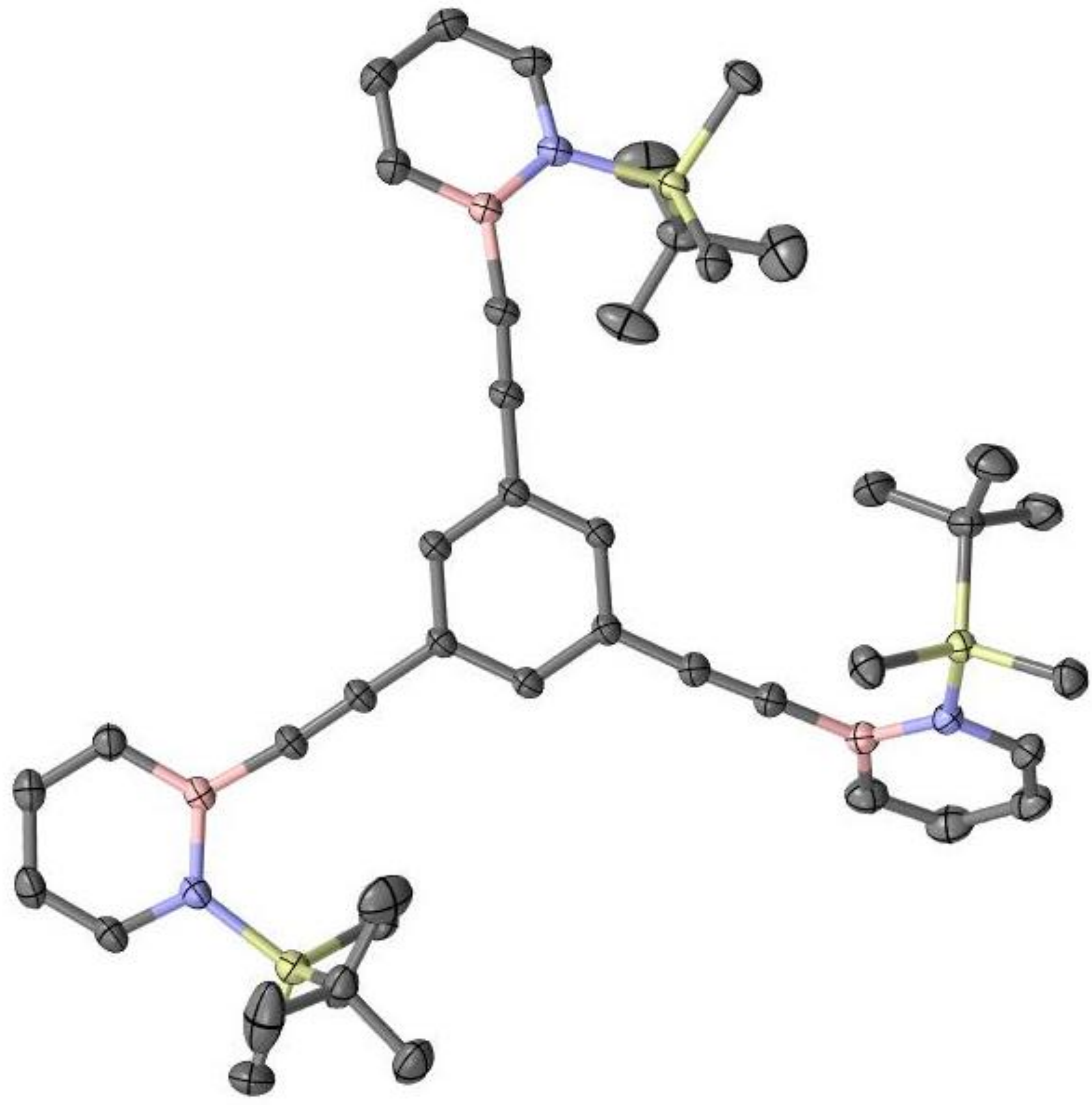

Figure S40. Crystal structure of **4**. Hydrogens are omitted for clarity and thermal ellipsoids are drawn at the 50% probability level.

11. Computations

Electronic structure computations: All geometries and harmonic vibrational frequencies were optimized using the Becke-Lee-Yang-Paar functional B3LYP[15, 16] and the 6-311+G(d,p) basis set[17] in Gaussian 16.[18] The following coordinates are given in Å. Verification of the stationary points involved analytical frequency computations, confirming minima with zero imaginary vibrational frequencies and one imaginary vibrational frequency for transition states.

For a verification of the transitions states, intrinsic reaction coordinates were calculated using the local quadratic approximation.REF

Free energies were computed using standard thermochemistry equations implemented in Gaussian. In this section, total energies are given in Hartree and kcal/mol.

In some images of the molecules given in the following, hydrogen atoms are omitted for clarity.

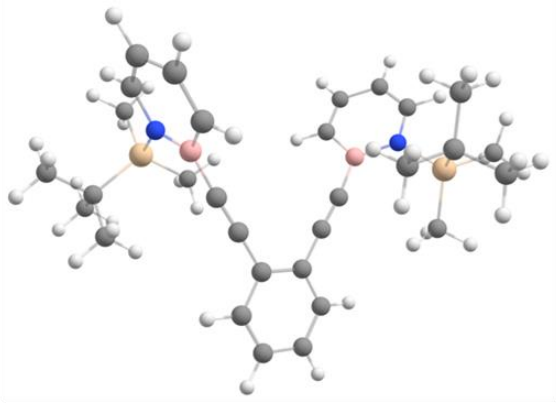

1

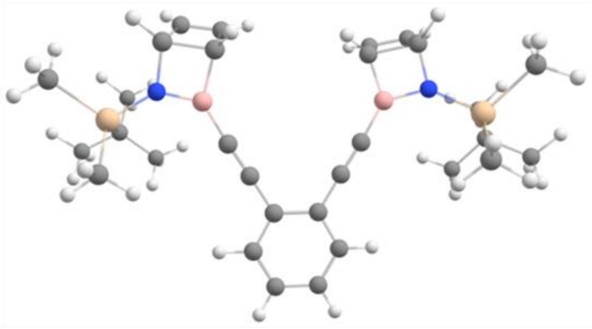

$\mathbf{1}_{\mathbf{Dewar}}$

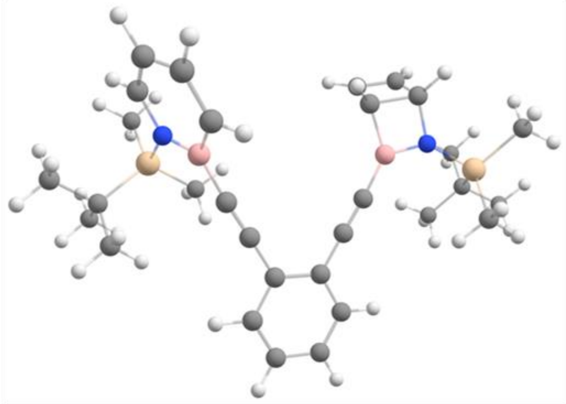

$\mathbf{1}_{\mathbf{IM}}$

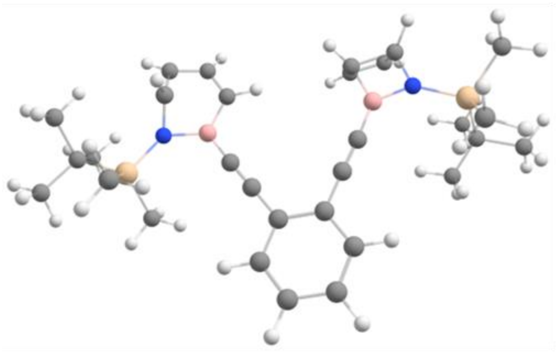

$\mathbf{1}_{\mathbf{im2}}$

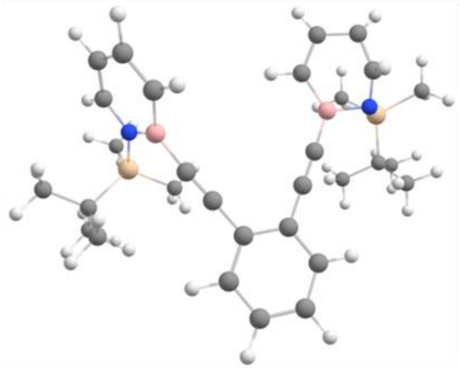

$\mathbf{1}_{\mathbf{im1}}$

Figure S41. Calculated geometries of the local minima **1**, $\mathbf{1}_{\mathbf{im1}}$, $\mathbf{1}_{\mathbf{IM}}$, $\mathbf{1}_{\mathbf{im2}}$ and $\mathbf{1}_{\boldsymbol{Dewar}}$ (B3LYP/6-311+G(d,p)).

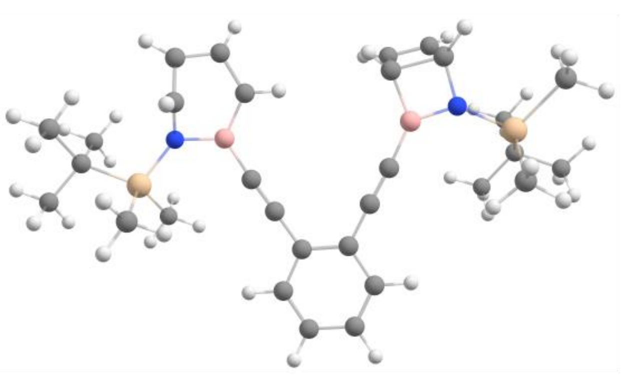

$\mathbf{1}_{TS1.1}$

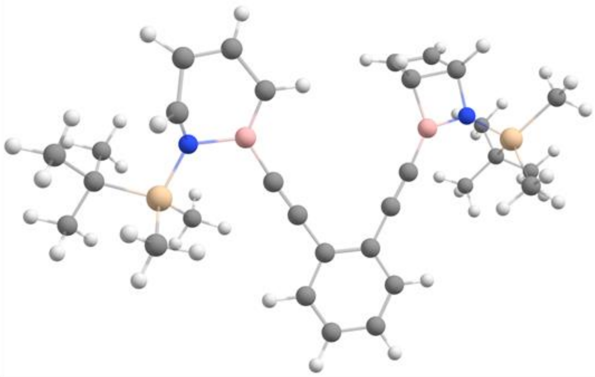

$\mathbf{1}_{TS1.2}$

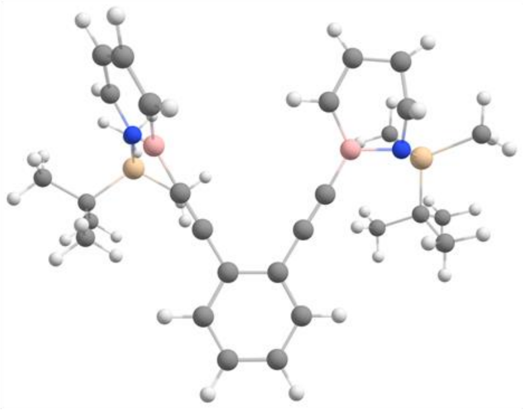

$\mathbf{1}_{TS2.1}$

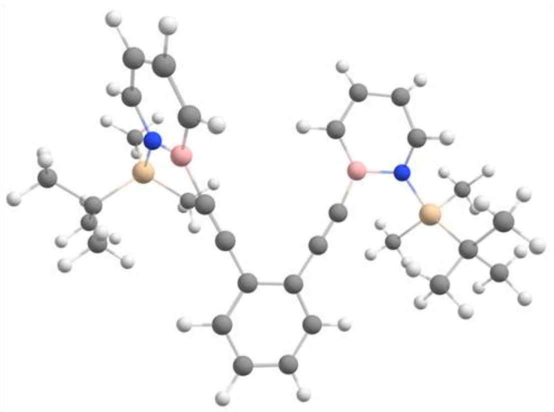

$\mathbf{1}_{TS2.2}$

Figure S42. Calculated geometries of the transition states $\mathbf{1}_{TS1.1}$, $\mathbf{1}_{TS1.2}$, $\mathbf{1}_{TS2.1}$ and $\mathbf{1}_{TS2.2}$ (B3LYP/6-311+G(d,p)).

B-N 1.458
C3-C6 2.809

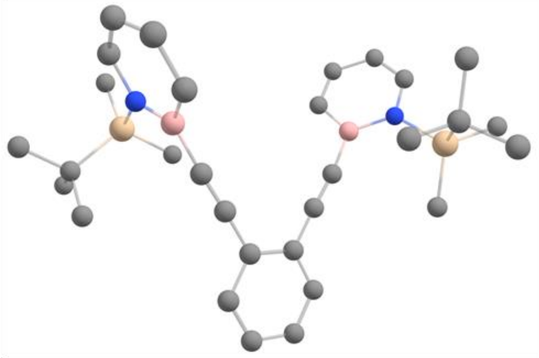

1

B-N 1.418
C3-C6 1.572

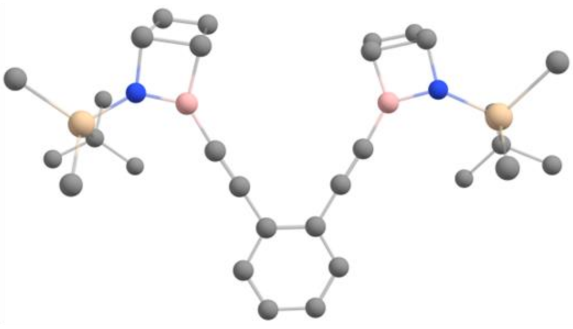

1_{Dewar}

B-N 1.458
C3-C6 2.809

B-N 1.419
C3-C6 1.572

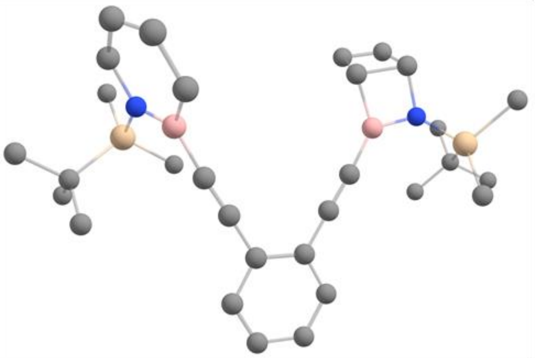

1_{IM}

B-N 1.459
C3-C6 2.810

B-N 1.583
C3-C6 2.285

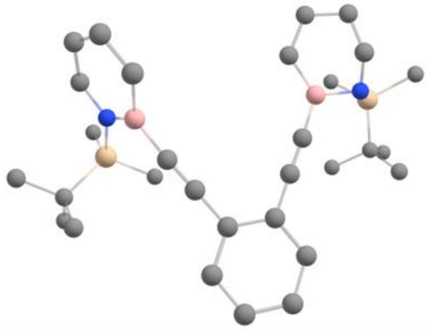

1_{im2}

B-N 1.510
C3-C6 2.134

B-N 1.420
C3-C6 1.572

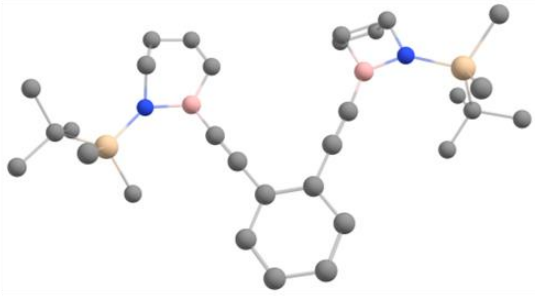

1_{im1}

Figure S43. Calculated geometries of the local minima **1**, **1_{im1}**, **1_{IM}**, **1_{im2}** and **1_{Dewar}** (B3LYP/6-311+G(d,p)). Hydrogens omitted for clarity. The B-N and C3-C6 distance is given in Å.

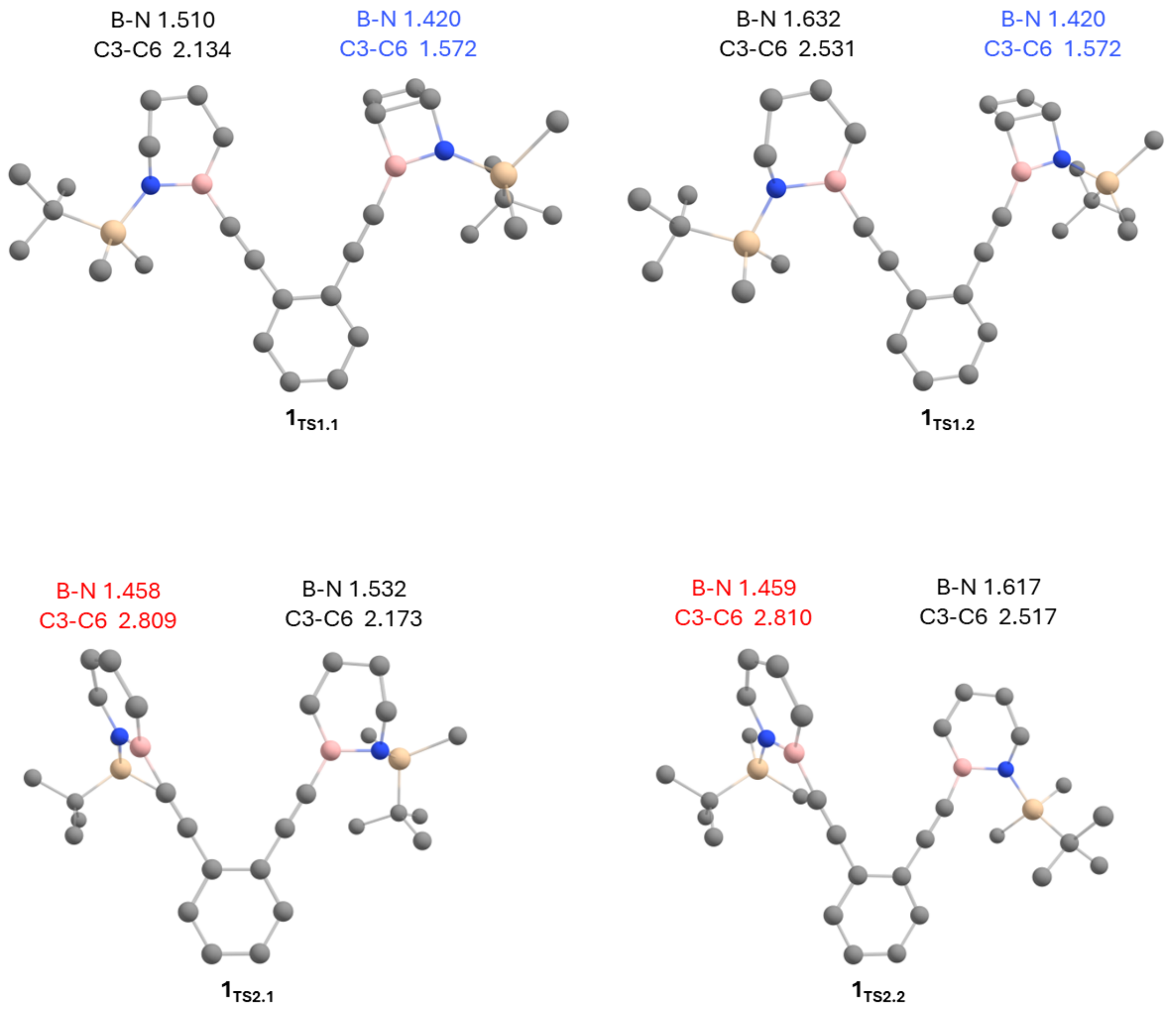

Figure S44. Calculated geometries of the transition states **1**$_{TS1.1}$, **1**$_{TS1.2}$, **1**$_{TS2.1}$ and **1**$_{TS2.2}$ (B3LYP/6-311+G(d,p)). Hydrogens omitted for clarity. The B-N and C3-C6 distance is given in Å.

Ortho-aza

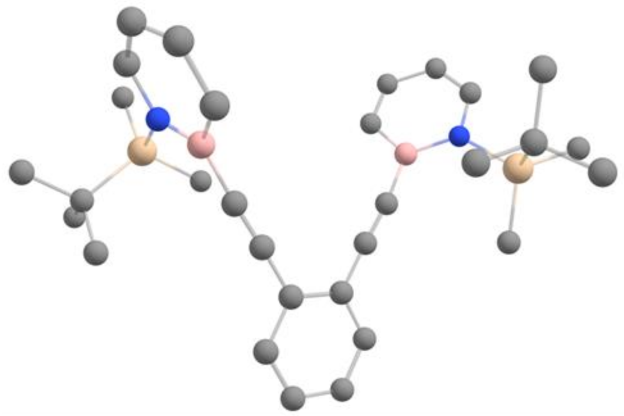

1

C	-0.66704300	5.23988900	-0.36967000
C	-1.25368900	4.01796400	-0.67402300
C	-0.63099400	2.80600100	-0.32678200
C	0.62221900	2.84377400	0.34411800
C	1.19698900	4.09263200	0.64121200
C	0.56213500	5.27754700	0.29142800
H	-1.16663200	6.16076300	-0.64756000
H	-2.20590500	3.98285700	-1.18924500
H	2.14983000	4.11585300	1.15599200
H	1.02409700	6.22785100	0.53285000
C	1.29876100	1.64899000	0.71876000
C	1.91801200	0.65302400	1.03914900
C	-1.25988500	1.57184800	-0.65534100
C	-1.83417600	0.53612200	-0.92914100
C	1.72745800	-1.61086100	2.37888800
C	4.36813800	-2.26383600	1.67909500
C	2.29016200	-2.78648800	2.80491400
H	0.70487600	-1.38592500	2.66574200
C	3.62412200	-3.11497000	2.44868800
H	5.38385700	-2.53767100	1.42466800
H	1.73227600	-3.48885600	3.41999200
H	4.07491300	-4.04194400	2.78102200
C	-1.96107300	-1.40016300	-2.71681700
C	-4.04447200	-2.61292000	-1.27440700
C	-2.54110500	-2.55703100	-3.17039200
H	-1.15594000	-0.95727000	-3.29498100
C	-3.59547100	-3.16640900	-2.44146500
H	-4.84365200	-3.10279700	-0.73333300
H	-2.20830100	-3.03144300	-4.09074000
H	-4.05706700	-4.08077000	-2.79336900
B	2.54411500	-0.65955200	1.52378800
B	-2.45798600	-0.77377700	-1.42698700
N	3.91021100	-1.05583600	1.19994300

N	-3.53661000	-1.45680400	-0.72286300
Si	-4.31849100	-0.88556200	0.83287900
Si	5.06756800	-0.11162700	0.13878300
C	-4.99620400	-2.41817700	1.70703300
H	-5.29753000	-2.13735500	2.72065800
H	-5.86616400	-2.86888800	1.22437800
H	-4.22318800	-3.18685900	1.79937600
C	-3.03998600	-0.12684400	1.98036300
H	-2.14251500	-0.74956900	2.02306600
H	-2.73198000	0.87589700	1.68858000
H	-3.46164700	-0.08082300	2.98969800
C	-5.74902100	0.32725400	0.41353600
C	-5.22660400	1.59528900	-0.29184900
H	-4.75050100	1.36564800	-1.24837800
H	-6.06267800	2.27700100	-0.49352900
H	-4.50000400	2.13694700	0.31939900
C	-6.43788900	0.74079600	1.73461700
H	-5.74992900	1.25320000	2.41392100
H	-7.26218500	1.43304300	1.52315200
H	-6.86214900	-0.11628200	2.26622200
C	-6.78808700	-0.35588800	-0.50070500
H	-7.60815400	0.33993800	-0.71748900
H	-6.35468800	-0.65711400	-1.45867200
H	-7.23064500	-1.24161700	-0.03512700
C	6.81902400	-0.59847400	0.65630400
H	7.52718200	0.08410100	0.17699100
H	6.94936800	-0.49471900	1.73764900
H	7.10903900	-1.61345600	0.37609400
C	4.90545600	1.73349300	0.45752400
H	4.87625400	1.93855000	1.53125900
H	5.78636800	2.23738000	0.04702400
H	4.01593800	2.17178500	0.00789600
C	4.75979400	-0.57386900	-1.69977000
C	5.81343100	0.15297200	-2.56731000
H	5.65736100	-0.09383000	-3.62462800
H	5.74256700	1.24110200	-2.47515000
H	6.83561400	-0.14254200	-2.31230300
C	4.90699600	-2.09668200	-1.90465200
H	4.74932900	-2.34661000	-2.96115100
H	5.90356000	-2.45785600	-1.63254100
H	4.17142600	-2.65992300	-1.32358600
C	3.35273300	-0.14452400	-2.16268100
H	3.19655600	0.93298500	-2.06568500
H	3.21741300	-0.40315700	-3.22046000
H	2.56230200	-0.64460800	-1.59829900

Ortho-TS2.2

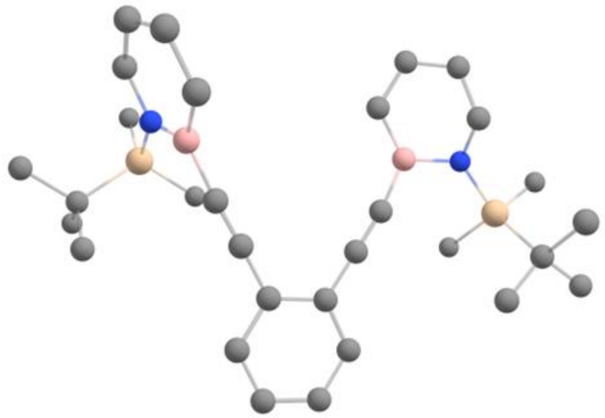

$\mathbf{1}_{TS2.2}$

C	-0.58381300	2.47801200	-0.72120100
C	-1.14238400	3.75358200	-0.91180800
C	-0.37192900	4.90258200	-0.78676000
C	0.79826800	2.37141300	-0.39372900
H	-0.82514500	5.87500700	-0.94147600
C	1.40376600	1.10272800	-0.19293700
C	-2.12734400	0.35188300	-0.94960000
C	-2.61151600	-1.78869900	-2.41416400
C	1.55527700	3.55057200	-0.26757700
H	1.58871700	5.69267000	-0.36153200
C	3.96502900	-2.62370500	-0.31949200
C	-4.43766100	-3.25637700	-1.79989900
H	-2.19315700	3.82553100	-1.16525800
H	2.60510900	3.46789400	-0.01631800
H	0.41983800	-2.47294300	-0.20434800
B	2.35196900	-1.42477400	0.21898800
N	3.91995600	-1.62926500	0.55561500
Si	-4.63661800	-0.45474300	1.12175300
C	-3.19603800	0.26793800	2.08649900
H	4.51877500	-2.53156100	-1.26137700
H	1.45416400	-4.77386900	0.47984000
H	4.05578400	-4.70937700	0.45361700
C	5.95999700	0.56632200	-0.51060600
C	-5.45364600	-1.73719900	2.24691000
H	-5.55309700	-2.77981700	-0.06580900
H	-1.79390100	-1.54733400	-3.08586500
H	-2.39145600	-0.46545900	2.18742500
C	-7.07514000	0.22678300	-0.19696400
H	-3.15314100	-3.55190100	-3.51084200
H	-5.03423200	-4.14048100	-1.98842500

H	-2.77013400	1.16016000	1.63052600
C	6.24683300	-0.34246100	-1.72496500
H	-3.54069200	0.52278000	3.09408300
H	-4.46926700	2.48976900	0.25510500
H	-7.81363800	0.98961300	-0.47343100
C	-5.27661900	1.97285300	-0.27039300
H	-6.72226000	-0.23640800	-1.12250500
H	-5.63773300	-1.28089300	3.22411400
H	-6.41109800	-2.11668700	1.88290800
H	-4.79074700	-2.59234300	2.40890300
C	-1.40183600	1.32233000	-0.85552200
C	1.90720600	0.00729800	-0.02499300
C	1.47240200	-2.59353300	0.02779100
C	0.98207100	4.79996700	-0.46245500
H	-7.60261800	-0.53615500	0.38389400
C	-4.73527100	-2.48604700	-0.71123800
C	-3.36266600	-2.90801500	-2.65972400
H	-4.86353000	1.57217000	-1.19915000
H	-6.03039400	2.72400300	-0.53905600
H	7.32390800	-0.94299400	2.18483800
B	-2.94113900	-0.91857600	-1.21509700
N	-4.04851700	-1.34163100	-0.36483600
Si	5.20253800	-0.42217600	0.96344000
C	4.46605700	0.69520300	2.27602600
H	-5.72573500	2.02038900	2.47261200
H	-7.23519300	2.29493800	1.60269300
H	-7.01079400	0.80347700	2.51717800
C	-5.92275900	0.87503600	0.59900900
C	6.52856700	-1.53284100	1.72133100
H	6.99333900	-2.18850200	0.97818300
H	6.08732300	-2.16687200	2.49488000
H	4.12772400	0.09659500	3.12638500
C	-6.49989100	1.52804900	1.87600100
H	3.60953100	1.25822800	1.90277400
H	5.21402700	1.40425900	2.64365800
H	5.32466400	-0.70121200	-2.19017900
C	7.29913300	1.17182300	-0.02475000
H	8.03735100	0.40391800	0.22094100
H	7.17166100	1.81083600	0.85510100
H	7.72840800	1.79696200	-0.81722300
C	5.04044600	1.71806300	-0.96155900
H	5.51059200	2.26745600	-1.78694600
H	4.86583700	2.43344500	-0.15276900
H	4.06850000	1.36083700	-1.30696600
C	2.04370900	-3.91001700	0.18502400
H	6.79121100	0.22202600	-2.49226600
C	3.38468000	-3.94351400	0.07515700
H	6.86483800	-1.20844800	-1.46601100

Ortho im2

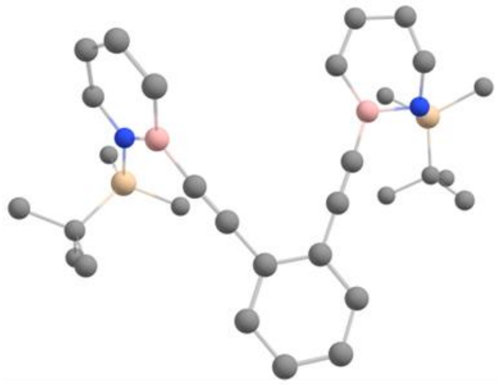

1$_{im2}$

C	-0.90479800	2.56284500	-1.24993800
C	-1.61363300	3.77142200	-1.36186900
C	-0.94895800	4.98849700	-1.44236800
C	0.51751900	2.59857500	-1.21875600
H	-1.51721500	5.90731900	-1.53137600
C	1.28083400	1.40561800	-1.10945100
C	-2.23436000	0.29086900	-1.08437900
C	-2.69626100	-1.99437000	-2.31822500
C	1.16738700	3.84332300	-1.29785500
H	0.96976900	5.97188100	-1.47166500
C	4.22783500	-1.75194900	-1.74615800
C	-4.23632100	-3.58370100	-1.33420600
H	-2.69599500	3.73725100	-1.39043100
H	2.25009400	3.86277600	-1.27233400
H	1.02222300	-2.33396000	-1.26224700
B	2.72850800	-0.90871300	-0.90376400
N	4.30307600	-0.82155600	-0.76247400
Si	-4.30136600	-0.59521400	1.40362800
C	-2.81494000	0.32737600	2.08762900
H	4.00558000	-1.41855500	-2.76194700
H	2.49900500	-4.45902900	-0.96685100
H	5.06554500	-3.80760200	-1.24699300
C	5.23947100	0.94726700	1.46411900
C	-4.80380600	-1.86061200	2.71702500
H	-5.12481700	-3.08517500	0.52073400
H	-2.02440200	-1.72403300	-3.12678700
H	-1.93828600	-0.32533400	2.12041200
C	-6.96360100	-0.24576700	0.43587400
H	-3.20194200	-3.88154200	-3.20651700
H	-4.75430100	-4.53413000	-1.37165900
H	-2.54501200	1.20961400	1.50964500
C	5.54565300	1.97923600	0.35913900
H	-3.03531700	0.63772500	3.11427000
H	-4.57364700	2.28351900	0.35729000
H	-7.81094500	0.42108600	0.23359500
C	-5.38906700	1.65468600	-0.00982500

H	-6.70355700	-0.73766100	-0.50547900
H	-4.88219100	-1.35212400	3.68255800
H	-5.76231400	-2.35098400	2.53237700
H	-4.04130500	-2.63811000	2.82222200
C	-1.61167900	1.33094400	-1.17161600
C	1.94492900	0.39143300	-1.01869600
C	2.09702000	-2.26321800	-1.09891200
C	0.44640500	5.02466400	-1.40962200
H	-7.31663000	-1.01244300	1.13244100
C	-4.44999000	-2.76358600	-0.26212500
C	-3.34835300	-3.19835500	-2.37297100
H	-5.07469800	1.23006100	-0.96613500
H	-6.24923000	2.30826100	-0.20332400
H	7.68905900	-1.35592600	1.05677100
B	-2.93510800	-1.06993900	-1.13804300
N	-3.84925300	-1.53340500	-0.09963700
Si	5.26534700	-0.83084300	0.74449000
C	4.62137200	-2.06831900	2.01586900
H	-5.43426800	1.85447900	2.76307200
H	-7.07468000	1.92197600	2.11914000
H	-6.56497900	0.52740700	3.07108900
C	-5.78193500	0.56524500	1.00852900
C	7.00699900	-1.31862800	0.20275900
H	7.41734700	-0.61249900	-0.52414300
H	7.01052400	-2.31093800	-0.25838300
H	4.74274500	-3.09439600	1.65977200
C	-6.23145800	1.24966300	2.31995800
H	3.55957300	-1.91401500	2.22320300
H	5.16492100	-1.97664700	2.96161500
H	4.79656300	1.95035600	-0.43482100
C	6.32085900	1.05400000	2.56383900
H	7.32908100	0.89540400	2.16990900
H	6.15973800	0.33833200	3.37656300
H	6.29978100	2.05639800	3.00900200
C	3.86626400	1.26894900	2.09236900
H	3.87637400	2.28464100	2.50820700
H	3.62197300	0.58637700	2.91191700
H	3.05668800	1.22424800	1.36031700
C	2.89496900	-3.47071100	-1.18621900
H	5.55412300	2.99125300	0.78377500
C	4.19659300	-3.19270600	-1.44557000
H	6.52584700	1.81138400	-0.09873400

Ortho-TS2.1

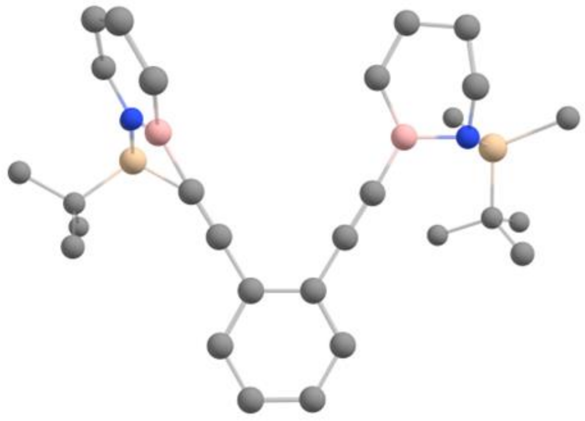

$\mathbf{1}_{TS2.1}$

C	-0.89645700	2.57551200	-1.21266700
C	-1.58092200	3.79668500	-1.34092800
C	-0.89306400	5.00293200	-1.38904000
C	0.52436700	2.58709900	-1.13173300
H	-1.44246600	5.93173900	-1.49079200
C	1.26432800	1.37991900	-1.00438800
C	-2.27178500	0.32668100	-1.10177300
C	-2.80871700	-1.91323400	-2.38721800
C	1.19788100	3.82095500	-1.17962600
H	1.04180700	5.95398300	-1.34397100
C	4.06017400	-1.96451000	-1.60350300
C	-4.36299700	-3.49719100	-1.41656200
H	-2.66205100	3.78223800	-1.40671000
H	2.27922100	3.82292100	-1.11693400
H	0.95729900	-2.28421400	-1.35743100
B	2.67256500	-0.96177800	-0.75927600
N	4.20103700	-0.97495600	-0.65692000
Si	-4.33086000	-0.57873300	1.39860900
C	-2.82830100	0.32944200	2.06612800
H	3.94858100	-1.68837600	-2.65166800
H	2.21115100	-4.50100100	-0.63839900
H	4.85183100	-4.01385600	-0.96149200
C	5.48116800	0.98285900	1.22196600
C	-4.80631200	-1.87104000	2.69333100
H	-5.20816600	-3.02795500	0.46636600
H	-2.14559900	-1.63749000	-3.20144000
H	-1.94640500	-0.31649500	2.04837600
C	-7.02147400	-0.20374700	0.51727600
H	-3.36845700	-3.76737700	-3.31414200
H	-4.90061100	-4.43595300	-1.46806500
H	-2.58494200	1.23553400	1.51355700
C	5.77928900	1.84573000	-0.02135600

H	-3.02106500	0.59999300	3.10937400
H	-4.60547100	2.29757700	0.35858200
H	-7.86718200	0.47315800	0.34353200
C	-5.44196200	1.68003800	0.02090600
H	-6.79829700	-0.69701200	-0.43294700
H	-4.86904400	-1.38078700	3.66940800
H	-5.76602300	-2.36118300	2.51543000
H	-4.03823600	-2.64654700	2.76864900
C	-1.62704900	1.35521300	-1.16629700
C	1.91165600	0.35604100	-0.89773400
C	1.99329800	-2.29261400	-1.01644500
C	0.50091100	5.01546600	-1.30725000
H	-7.35901300	-0.96718100	1.22473500
C	-4.54082200	-2.69956400	-0.31998400
C	-3.48647000	-3.10316800	-2.46103000
H	-5.16949500	1.25173300	-0.94686600
H	-6.29972300	2.34513200	-0.14104100
H	7.71808600	-1.52186600	0.89607800
B	-3.00694700	-1.01637200	-1.17893900
N	-3.91282900	-1.48681500	-0.13742100
Si	5.31599900	-0.86489800	0.72874800
C	4.71065100	-1.88729400	2.19489400
H	-5.39629800	1.88071000	2.79258500
H	-7.05799200	1.95850900	2.20513000
H	-6.52380500	0.56052500	3.13931300
C	-5.81294600	0.59410100	1.05114100
C	6.96179100	-1.54682200	0.10653500
H	7.34384500	-0.97280700	-0.74210500
H	6.86085100	-2.58844700	-0.21239900
H	4.71054000	-2.95219600	1.94704800
C	-6.21232300	1.28137100	2.37743700
H	3.69140800	-1.61361900	2.48033300
H	5.35320300	-1.74706400	3.06982100
H	4.97214800	1.78526700	-0.75483200
C	6.64770500	1.13026500	2.22552600
H	7.60766800	0.83889700	1.78923600
H	6.49399400	0.53453500	3.13093700
H	6.73717500	2.17772700	2.53947800
C	4.18753200	1.49146000	1.89347700
H	4.30378400	2.54611100	2.17480500
H	3.95623900	0.93605100	2.80732500
H	3.32371700	1.42320900	1.22790100
C	2.69144300	-3.57371800	-0.93809900
H	5.89451100	2.89753900	0.27050400
C	4.00087100	-3.38566800	-1.19319600
H	6.70701400	1.54313000	-0.51753700

Ortho-IM

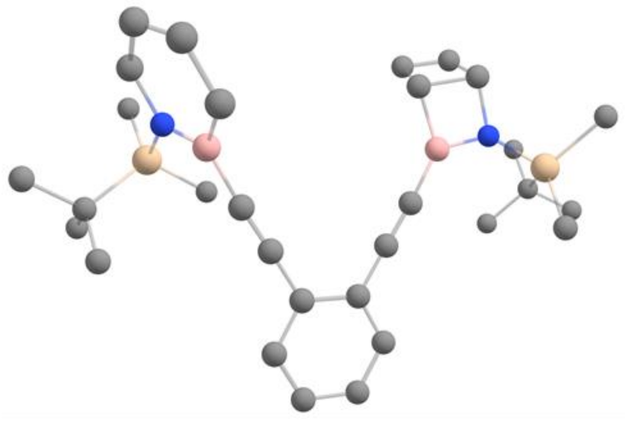

$\mathbf{1_{IM}}$

C	0.45836400	5.13832300	-0.50980400
C	1.15196500	3.93636400	-0.45849700
C	0.48326200	2.70607700	-0.58724000
C	-0.92777300	2.70191400	-0.77113500
C	-1.60862400	3.93076300	-0.82066300
C	-0.92598500	5.13411400	-0.69249700
H	0.99358100	6.07521500	-0.40901600
H	2.22579800	3.93025400	-0.31728400
H	-2.68195900	3.92520100	-0.96571800
H	-1.47289600	6.06893500	-0.73578500
C	-1.65096600	1.48453100	-0.90759800
C	-2.29342600	0.45709700	-1.00352000
C	1.21989100	1.49163900	-0.53060000
C	1.86638900	0.46239100	-0.48023000
C	-2.69943400	-1.63365000	-2.55770500
C	-4.60956900	-2.62408000	-0.75115400
C	-3.36275600	-2.80542000	-2.81786700
H	-1.96445500	-1.27848700	-3.27347100
C	-4.32927500	-3.30228300	-1.90508500
H	-5.34415800	-3.03008500	-0.06769800
H	-3.16381500	-3.37730300	-3.72126700
H	-4.85556900	-4.22772600	-2.10379900
C	2.10112700	-2.40734400	-0.43466000
C	3.65069500	-2.67347100	-0.43307400
C	2.11545600	-3.13970500	0.89788600
H	1.46267300	-2.78552900	-1.23405300
C	3.44133800	-3.34683800	0.91647900
H	4.13405500	-3.23022900	-1.23657800
H	1.31923200	-3.36423000	1.59940400
H	4.12748100	-3.76136200	1.64532900
B	-3.01169200	-0.86857200	-1.28510300
B	2.58281900	-0.87072900	-0.43281900
N	-4.00600100	-1.44044200	-0.38542000
N	3.96272100	-1.20116300	-0.41921000

Si	5.53932700	-0.38508800	-0.44848700
Si	-4.56754800	-0.69878700	1.19345300
C	6.77536300	-1.63299100	-1.13658100
H	7.77144200	-1.18942300	-1.22262500
H	6.86239000	-2.52356800	-0.50789800
H	6.47371200	-1.95867800	-2.13669500
C	5.39400600	1.08828700	-1.61370700
H	5.15990700	0.74535100	-2.62574600
H	4.59812600	1.77001900	-1.30439100
H	6.32875000	1.65480400	-1.65986200
C	6.05902500	0.19084400	1.31244200
C	5.04483700	1.21854100	1.85614600
H	4.03440800	0.80466800	1.91549200
H	5.33285100	1.53272300	2.86763700
H	5.00158200	2.11830100	1.23530900
C	7.45481400	0.84958900	1.25089200
H	7.47002400	1.72408900	0.59322700
H	7.75326500	1.19017400	2.25054800
H	8.22423500	0.15363300	0.90287300
C	6.11630200	-1.01081800	2.27773700
H	6.42320700	-0.67707000	3.27734200
H	5.14135800	-1.49500800	2.37871900
H	6.83818300	-1.76655900	1.95249600
C	-5.13613700	-2.12028700	2.30098900
H	-4.36595800	-2.89474700	2.36443100
H	-5.29261400	-1.73438100	3.31272400
H	-6.06856200	-2.59516000	1.98810000
C	-3.14152700	0.14238900	2.08073000
H	-3.43210800	0.30873800	3.12311400
H	-2.25455900	-0.49683300	2.08196200
H	-2.85936600	1.09916000	1.64424100
C	-6.02785000	0.49900200	0.83935400
C	-6.54668100	1.04783300	2.18859100
H	-7.38319200	1.73543200	2.01315400
H	-6.91236000	0.25278600	2.84529500
H	-5.77622800	1.60560800	2.72959200
C	-5.58233800	1.68438200	-0.04090600
H	-6.43096600	2.35924100	-0.21014000
H	-4.78629900	2.26801600	0.42911200
H	-5.22157000	1.35687900	-1.01917000
C	-7.17730700	-0.24406600	0.12608700
H	-7.56528600	-1.07587100	0.72179500
H	-8.01275200	0.44459900	-0.05100100
H	-6.86828900	-0.63834100	-0.84607500

Ortho-TS1.2

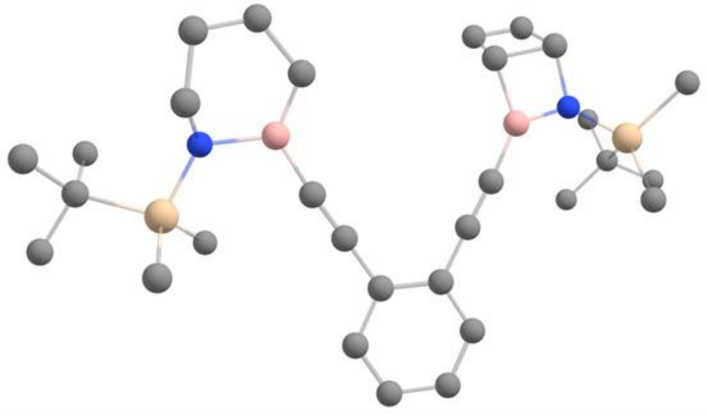

$\mathbf{1}_{TS1.2}$

C	-0.74949200	2.50485800	-0.38028600
C	0.67259100	2.56829400	-0.43610400
C	0.55899600	5.00040000	-0.44373100
C	-0.83521000	4.93943500	-0.38952700
H	1.06419800	5.95888800	-0.46839700
H	-1.42040100	5.85184800	-0.37198600
C	1.46243400	1.38806800	-0.46115000
C	2.15754300	0.39040100	-0.48107700
C	2.53336400	-2.46335000	-0.57861500
C	2.58889100	-3.25024600	0.72115800
H	1.91305100	-2.83850900	-1.39380000
C	4.09388500	-2.65270900	-0.59234900
C	3.92295100	-3.39287300	0.72733400
H	1.80751400	-3.53987900	1.41512300
H	4.60072200	-3.15064800	-1.41993400
H	4.63123000	-3.80363400	1.43684300
B	2.93644000	-0.90583100	-0.50809500
N	4.33225000	-1.16893300	-0.51664800
Si	5.86167800	-0.27185000	-0.50321000
C	7.16913000	-1.43033800	-1.21832300
H	6.89316900	-1.74227900	-2.23018600
H	8.14097200	-0.93277000	-1.28370300
H	7.30050600	-2.33395200	-0.61643000
C	6.33841300	0.27382000	1.27934800
C	5.65055800	1.22851300	-1.62357600
H	4.80534700	1.84588300	-1.31004100
H	6.54685300	1.85576200	-1.63150200
H	5.45823400	0.90681700	-2.65126000
C	7.70929800	0.98485400	1.25600500
H	7.98049100	1.31165100	2.26792400
H	8.50955500	0.32689200	0.90353900
H	7.70208000	1.87576300	0.62040700

C	5.27942900	1.24740500	1.83712800
H	4.28342900	0.79757000	1.86756800
H	5.54058500	1.54102400	2.86199000
H	5.21308800	2.16367800	1.24307600
C	6.42721800	-0.95234400	2.21081600
H	5.46972600	-1.47468400	2.28346300
H	7.18139900	-1.67131700	1.87506200
H	6.70815700	-0.63726800	3.22398800
C	-1.43215000	1.26138400	-0.34979600
C	-2.02637800	0.19939600	-0.32642500
C	-1.97628500	-2.43430300	-0.62313500
C	-2.77627900	-3.62864600	-0.77078300
H	-0.89968200	-2.47309300	-0.74771900
C	-4.40329200	-1.94398200	-1.14589200
C	-4.08352400	-3.39784700	-0.99246000
H	-2.37598300	-4.62693600	-0.61636600
H	-4.82631400	-1.57143700	-2.08908100
H	-4.90218400	-4.09837300	-0.85922200
B	-2.66708400	-1.17725000	-0.30181800
N	-4.28462600	-1.16105200	-0.08791700
Si	-5.29211400	0.29147400	0.28595200
C	-5.35589300	1.45171100	-1.19817400
H	-5.92941000	2.35278800	-0.96054400
H	-4.34559500	1.75793100	-1.47934900
H	-5.82146800	0.98997800	-2.07343100
C	-7.04031400	-0.41121900	0.68443800
C	-4.54628700	1.11040400	1.79827500
H	-3.57558800	1.55270200	1.57149000
H	-5.20428600	1.90156200	2.16999200
H	-4.40179600	0.38646900	2.60417100
C	-7.62423200	-1.18810100	-0.51207900
H	-8.63561600	-1.53921100	-0.27293400
H	-7.70084200	-0.57159500	-1.41281500
H	-7.03581800	-2.07799700	-0.75758300
C	-6.97847100	-1.34800200	1.90965200
H	-6.31270100	-2.19854400	1.74081200
H	-6.63057400	-0.82592900	2.80533300
H	-7.97750000	-1.74410000	2.13064300
C	-7.98031800	0.77418900	1.00474000
H	-8.09556800	1.45220500	0.15377000
H	-8.97921700	0.39964500	1.25935200
H	-7.62876400	1.36069800	1.85861100
C	1.29955500	3.82635000	-0.46552100
H	2.38128800	3.86278200	-0.50666400
C	-1.47739700	3.70916800	-0.35697700
H	-2.55872900	3.66203900	-0.31456400

Ortho-im1

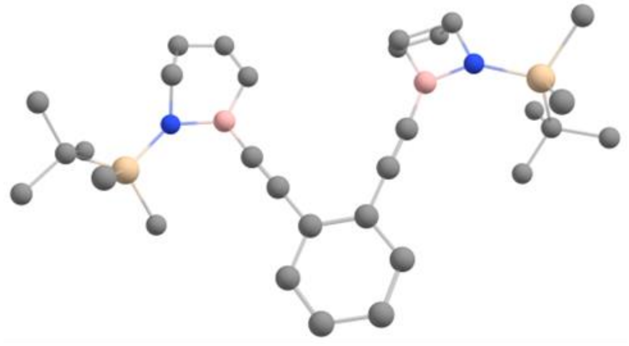

$\mathbf{1}_{im1}$

C	-0.65647600	2.64053500	-0.02655300
C	0.75887600	2.64734200	-0.17682800
C	0.74063200	5.08088600	-0.15384500
C	-0.64761400	5.07467000	-0.00567300
H	1.28070000	6.01928100	-0.20299700
H	-1.19315100	6.00895800	0.06121500
C	1.49835100	1.43722900	-0.26528500
C	2.15505700	0.41652000	-0.34018300
C	2.43898900	-2.44955000	-0.43904300
C	2.59926200	-3.22774100	0.85785800
H	1.73496300	-2.81626000	-1.18757200
C	3.98526800	-2.68261800	-0.60079100
C	3.92316600	-3.40719900	0.73685400
H	1.88273900	-3.49048500	1.62844100
H	4.39552400	-3.20145200	-1.46803900
H	4.68553800	-3.83157600	1.37894700
B	2.89030600	-0.90302700	-0.42588500
N	4.27035400	-1.20603500	-0.56436100
Si	5.82165400	-0.35551200	-0.70150900
C	7.02590300	-1.56731400	-1.50348700
H	6.65080700	-1.89343200	-2.47829000
H	8.00051800	-1.09931400	-1.66887700
H	7.18795400	-2.46073700	-0.89389000
C	6.46577500	0.20602900	1.02226100
C	5.55619500	1.12983400	-1.83000000
H	4.75496100	1.77432900	-1.46055200
H	6.46372600	1.73363100	-1.92316500
H	5.27264400	0.79557900	-2.83224900
C	7.85951500	0.85392200	0.86945700
H	8.22529600	1.19303100	1.84689300
H	8.59954600	0.15198600	0.47315700
H	7.83935500	1.72834700	0.21157100
C	5.49932500	1.23820500	1.63954800

H	4.49026200	0.83524800	1.76130000
H	5.85572000	1.54079800	2.63253400
H	5.42540200	2.14394600	1.03043200
C	6.57801100	-1.00256800	1.97391800
H	5.60784400	-1.47839300	2.13902800
H	7.26871500	-1.76275600	1.59510000
H	6.95690600	-0.67907300	2.95185600
C	-1.38644400	1.42276000	0.04069000
C	-2.02282900	0.38924800	0.09662400
C	-1.98864600	-2.24937100	0.04009000
C	-2.64453000	-3.54982700	0.08071700
H	-0.93128500	-2.17731800	-0.21565800
C	-3.91602700	-1.96803200	-0.83163100
C	-3.89894000	-3.38150000	-0.37002800
H	-2.19835100	-4.44738300	0.49752700
H	-3.80041300	-1.70332500	-1.88449000
H	-4.78330600	-3.99203800	-0.22900100
B	-2.75823100	-0.94852700	0.21136500
N	-4.26438900	-1.03015700	0.13531600
Si	-5.46650500	0.27511300	0.09851000
C	-5.28739700	1.31457200	-1.46898400
H	-6.00061300	2.14448600	-1.47827300
H	-4.28077200	1.73724200	-1.52678300
H	-5.45424400	0.72942500	-2.37816700
C	-7.20400200	-0.54650100	0.15906500
C	-5.14819800	1.32290400	1.62567900
H	-4.14415100	1.75236500	1.59249700
H	-5.86482000	2.14664500	1.69496400
H	-5.22548500	0.72944200	2.54016300
C	-7.41464700	-1.46594400	-1.06179700
H	-8.41294900	-1.92013000	-1.02501900
H	-7.34214500	-0.92031700	-2.00747200
H	-6.69192800	-2.28709100	-1.09043800
C	-7.35625800	-1.37969500	1.44870200
H	-6.59400000	-2.15977000	1.52107500
H	-7.28141800	-0.75845500	2.34603100
H	-8.33961600	-1.86651900	1.47067000
C	-8.28901400	0.55328100	0.14127200
H	-8.25252800	1.15465000	-0.77209500
H	-9.28631500	0.09863000	0.19109200
H	-8.20071800	1.23206300	0.99507400
C	1.43230700	3.87994400	-0.23807500
H	2.50931000	3.87573700	-0.35222300
C	-1.33444900	3.86932400	0.05651300
H	-2.41141600	3.86064700	0.17132800

Ortho_TS1.1

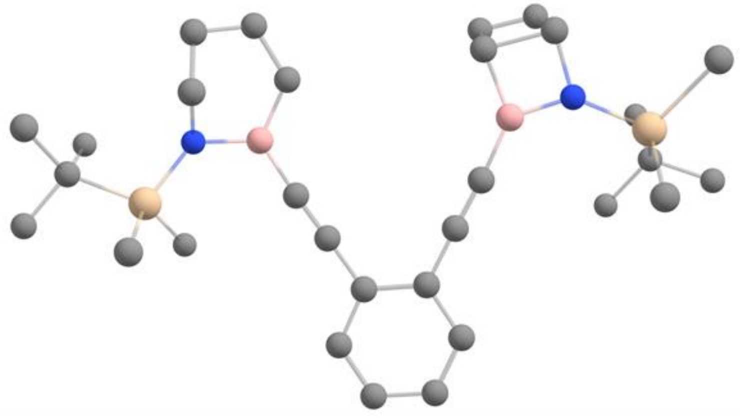

$\mathbf{1}_{TS1.1}$

C	-0.65647600	2.64053500	-0.02655300
C	0.75887600	2.64734200	-0.17682800
C	0.74063200	5.08088600	-0.15384500
C	-0.64761400	5.07467000	-0.00567300
H	1.28070000	6.01928100	-0.20299700
H	-1.19315100	6.00895800	0.06121500
C	1.49835100	1.43722900	-0.26528500
C	2.15505700	0.41652000	-0.34018300
C	2.43898900	-2.44955000	-0.43904300
C	2.59926200	-3.22774100	0.85785800
H	1.73496300	-2.81626000	-1.18757200
C	3.98526800	-2.68261800	-0.60079100
C	3.92316600	-3.40719900	0.73685400
H	1.88273900	-3.49048500	1.62844100
H	4.39552400	-3.20145200	-1.46803900
H	4.68553800	-3.83157600	1.37894700
B	2.89030600	-0.90302700	-0.42588500
N	4.27035400	-1.20603500	-0.56436100
Si	5.82165400	-0.35551200	-0.70150900
C	7.02590300	-1.56731400	-1.50348700
H	6.65080700	-1.89343200	-2.47829000
H	8.00051800	-1.09931400	-1.66887700
H	7.18795400	-2.46073700	-0.89389000
C	6.46577500	0.20602900	1.02226100
C	5.55619500	1.12983400	-1.83000000
H	4.75496100	1.77432900	-1.46055200
H	6.46372600	1.73363100	-1.92316500
H	5.27264400	0.79557900	-2.83224900
C	7.85951500	0.85392200	0.86945700
H	8.22529600	1.19303100	1.84689300
H	8.59954600	0.15198600	0.47315700
H	7.83935500	1.72834700	0.21157100
C	5.49932500	1.23820500	1.63954800
H	4.49026200	0.83524800	1.76130000

H	5.85572000	1.54079800	2.63253400
H	5.42540200	2.14394600	1.03043200
C	6.57801100	-1.00256800	1.97391800
H	5.60784400	-1.47839300	2.13902800
H	7.26871500	-1.76275600	1.59510000
H	6.95690600	-0.67907300	2.95185600
C	-1.38644400	1.42276000	0.04069000
C	-2.02282900	0.38924800	0.09662400
C	-1.98864600	-2.24937100	0.04009000
C	-2.64453000	-3.54982700	0.08071700
H	-0.93128500	-2.17731800	-0.21565800
C	-3.91602700	-1.96803200	-0.83163100
C	-3.89894000	-3.38150000	-0.37002800
H	-2.19835100	-4.44738300	0.49752700
H	-3.80041300	-1.70332500	-1.88449000
H	-4.78330600	-3.99203800	-0.22900100
B	-2.75823100	-0.94852700	0.21136500
N	-4.26438900	-1.03015700	0.13531600
Si	-5.46650500	0.27511300	0.09851000
C	-5.28739700	1.31457200	-1.46898400
H	-6.00061300	2.14448600	-1.47827300
H	-4.28077200	1.73724200	-1.52678300
H	-5.45424400	0.72942500	-2.37816700
C	-7.20400200	-0.54650100	0.15906500
C	-5.14819800	1.32290400	1.62567900
H	-4.14415100	1.75236500	1.59249700
H	-5.86482000	2.14664500	1.69496400
H	-5.22548500	0.72944200	2.54016300
C	-7.41464700	-1.46594400	-1.06179700
H	-8.41294900	-1.92013000	-1.02501900
H	-7.34214500	-0.92031700	-2.00747200
H	-6.69192800	-2.28709100	-1.09043800
C	-7.35625800	-1.37969500	1.44870200
H	-6.59400000	-2.15977000	1.52107500
H	-7.28141800	-0.75845500	2.34603100
H	-8.33961600	-1.86651900	1.47067000
C	-8.28901400	0.55328100	0.14127200
H	-8.25252800	1.15465000	-0.77209500
H	-9.28631500	0.09863000	0.19109200
H	-8.20071800	1.23206300	0.99507400
C	1.43230700	3.87994400	-0.23807500
H	2.50931000	3.87573700	-0.35222300
C	-1.33444900	3.86932400	0.05651300
H	-2.41141600	3.86064700	0.17132800

Ortho-Dewar

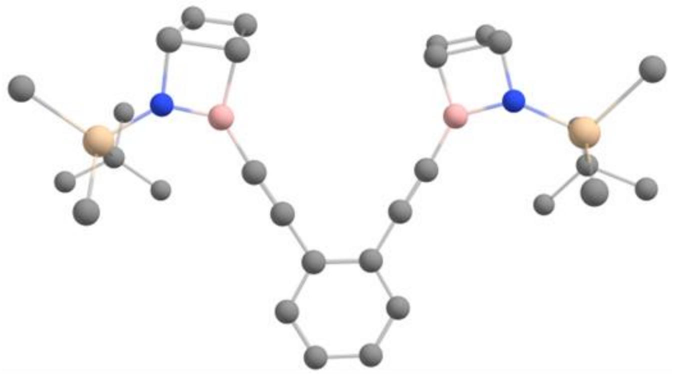

$\mathbf{1}_{\mathbf{Dewar}}$

C	0.71722000	5.11733600	0.12450300
C	1.39717900	3.90697900	0.07892400
C	0.70588200	2.69279700	-0.07593600
C	-0.71262500	2.71654400	-0.18853400
C	-1.37858300	3.95374300	-0.14201700
C	-0.67442100	5.14095600	0.01269600
H	1.27048100	6.04126800	0.24598700
H	2.47643900	3.88268300	0.16499000
H	-2.45797800	3.96594800	-0.22931500
H	-1.20851100	6.08333400	0.04643600
C	-1.46121100	1.51919000	-0.35073900
C	-2.12757300	0.51141400	-0.49088300
C	1.42950100	1.46995900	-0.11288600
C	2.07347800	0.43855500	-0.13900300
C	-2.43755200	-2.31573400	-0.95507600
C	-3.99368900	-2.53326000	-1.01063100
C	-2.48723100	-3.27177000	0.22675000
H	-1.80243800	-2.56631200	-1.80588600
C	-3.81818100	-3.44087600	0.19914600
H	-4.48323100	-2.92412200	-1.90325600
H	-1.70571600	-3.63866000	0.88316200
H	-4.52377300	-3.95794300	0.83824000
C	2.33144100	-2.42653400	0.02447500
C	3.85521100	-2.69787000	-0.25113400
C	2.60365500	-3.07540200	1.37287800
H	1.55733300	-2.85105000	-0.61612900
C	3.91053400	-3.28594700	1.15220200
H	4.18264100	-3.30413600	-1.09631800
H	1.95740500	-3.25157200	2.22599500
H	4.72571800	-3.65676000	1.76185400
B	-2.87747300	-0.79075400	-0.68449100
B	2.79302200	-0.89472100	-0.15664200
N	-4.26533200	-1.07816000	-0.73788500
N	4.15221200	-1.22840300	-0.38565400

Si	5.69119600	-0.42319100	-0.75884000
Si	-5.82100000	-0.22899500	-0.61624000
C	6.78973000	-1.72380000	-1.57104700
H	7.74879400	-1.29228000	-1.87151400
H	6.99934800	-2.56656700	-0.90636700
H	6.31166700	-2.12010800	-2.47199200
C	5.32418800	0.96449100	-1.97917200
H	4.91299000	0.55170600	-2.90500600
H	4.59252600	1.67097600	-1.57971400
H	6.23040800	1.52168500	-2.23456400
C	6.51652500	0.27263200	0.83365500
C	5.61591000	1.35082800	1.47139200
H	4.63245000	0.95596200	1.74097800
H	6.08030800	1.73439500	2.38894400
H	5.46270900	2.20296000	0.80255400
C	7.87870500	0.90654900	0.47506700
H	7.77618300	1.72899600	-0.23953300
H	8.35027600	1.31707300	1.37694200
H	8.57384100	0.17623400	0.04986400
C	6.74542600	-0.85592300	1.85982300
H	7.22750500	-0.45435600	2.76040000
H	5.80424900	-1.31775600	2.16970100
H	7.39646400	-1.64345500	1.46738900
C	-5.63999000	1.41534200	-1.51838100
H	-5.42082700	1.24155700	-2.57594600
H	-6.55818300	2.00720500	-1.45944200
H	-4.82349700	2.01275700	-1.10565400
C	-7.08989000	-1.31192800	-1.49664500
H	-8.07329500	-0.83326200	-1.50465500
H	-6.79645700	-1.47627300	-2.53787300
H	-7.20204800	-2.29127200	-1.02306600
C	-6.31777000	0.05285800	1.22077600
C	-7.70530500	0.72861200	1.28406200
H	-7.98942600	0.90401600	2.32941400
H	-7.71543500	1.69930000	0.77885300
H	-8.48692600	0.10775200	0.83576800
C	-6.38266700	-1.29238300	1.97268100
H	-6.67759000	-1.12662100	3.01680900
H	-7.11665400	-1.97509500	1.53295500
H	-5.41322400	-1.79749800	1.98177800
C	-5.28561400	0.96375800	1.91746500
H	-5.23720200	1.95474000	1.45654300
H	-5.56001900	1.10619900	2.97057900
H	-4.27966700	0.53557200	1.89516700

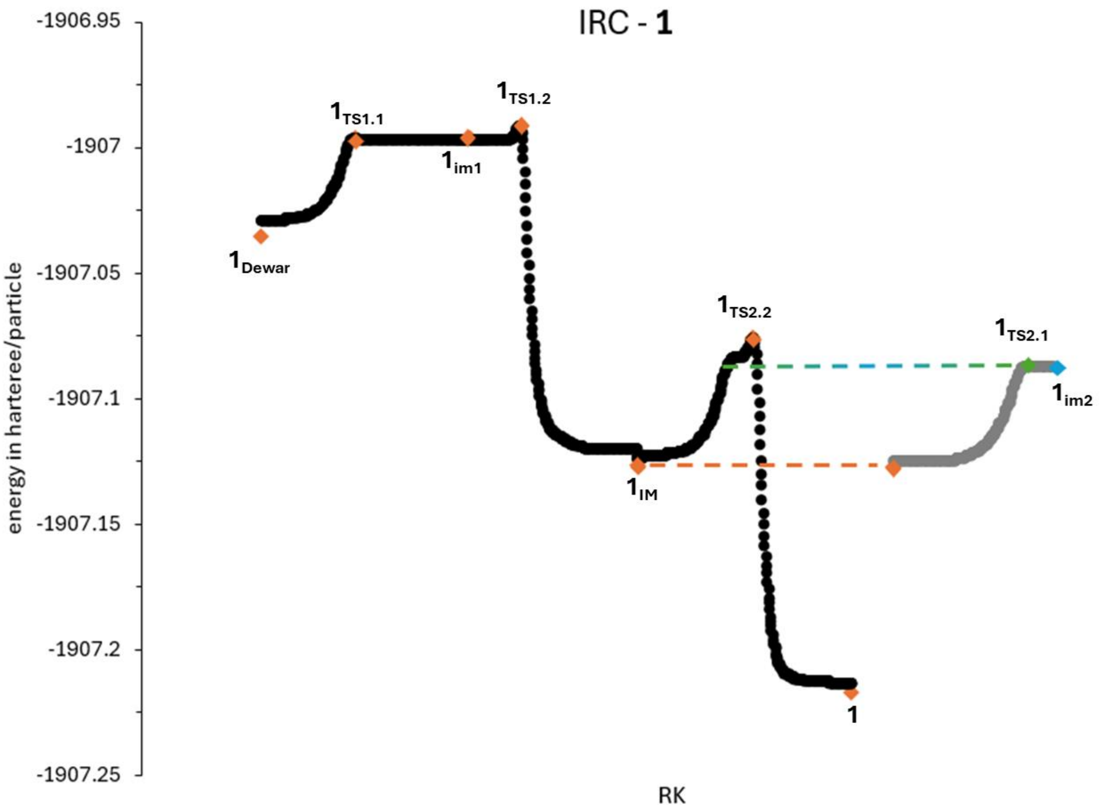

Figure S45. Intrinsic reaction coordinates calculated in both directions from the transition states $\mathbf{1_{TS1.1}}$, $\mathbf{1_{TS1.2}}$, $\mathbf{1_{TS2.1}}$ and $\mathbf{1_{TS2.2}}$. The corresponding calculated geometries of the transition states as well as the local minima are given in orange. The offset of the IRC's at the intermediates $\mathbf{1_{im1}}$ and $\mathbf{1_{im2}}$ are due to different rotamers along the C-C triple bond or the *tert*-butyldimethylsilyl group. Transition state 1TS2.2 relaxes directly to 1IM or 1 respectively. This IRC from TS2.2 to IM shows a shoulder. Based on the geometry at this shoulder also a TS2.1 and im1 structure can be calculated.

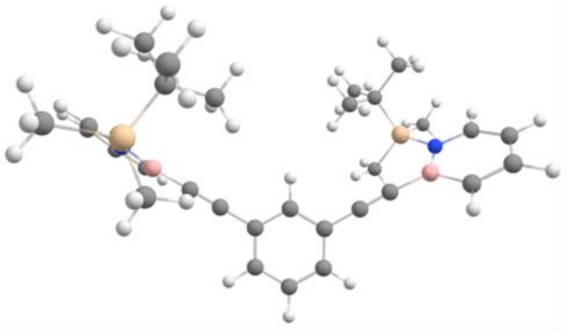

2

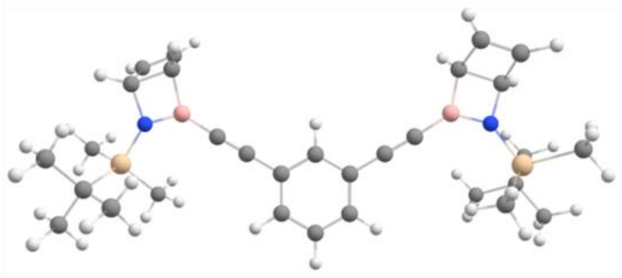

2_{Dewar}

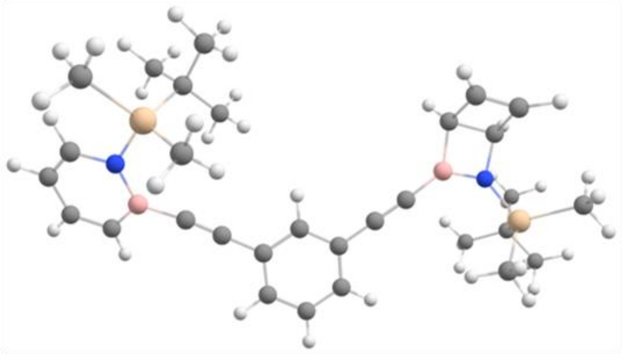

2_{IM}

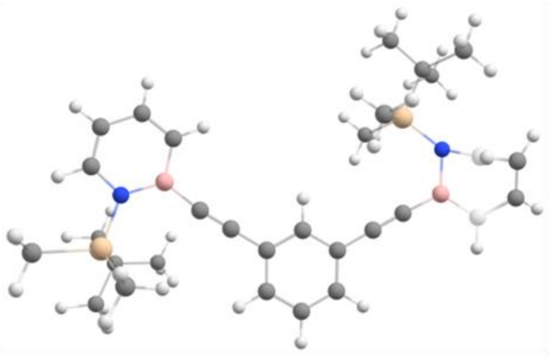

2_{im2}

Figure S46. Calculated geometries of the local minima **2**, **2_{IM}**, **2_{im2}** and **2_{Dewar}** (B3LYP/6-311+G(d,p)).

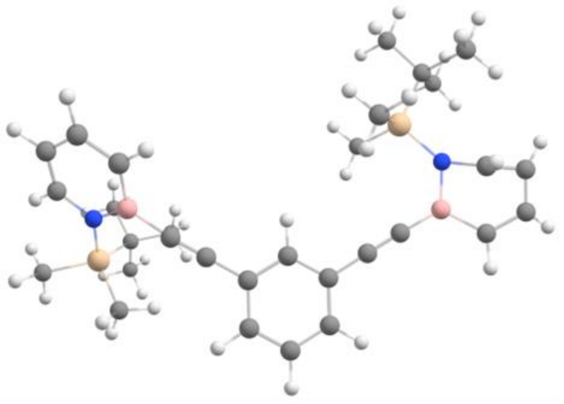

$\mathbf{2}_{TS2.1}$

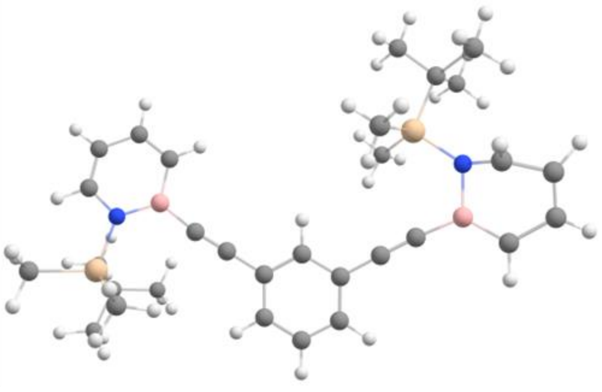

$\mathbf{2}_{TS2.2}$

Figure S47. Calculated geometries of the transition states $\mathbf{2}_{\mathbf{TS2.1}}$ and $\mathbf{2}_{\mathbf{TS2.2}}$ (B3LYP/6-311+G(d,p)).

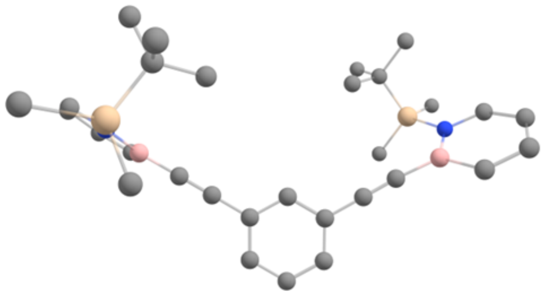

2

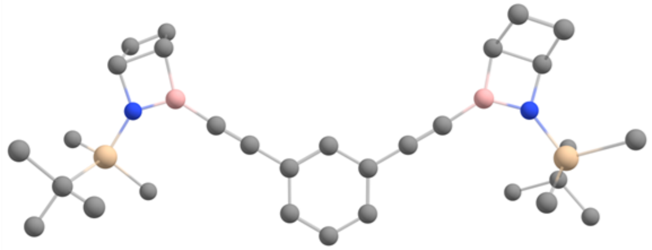

2$_{\mathbf{Dewar}}$

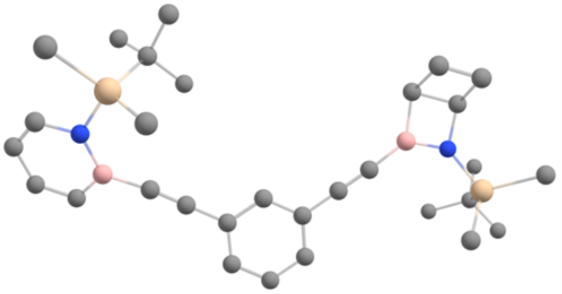

2$_{\mathbf{IM}}$

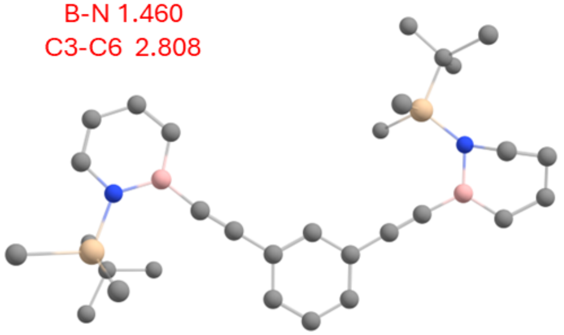

2$_{\mathbf{im2}}$

Figure S48. . Calculated geometries of the local minima **2**, **2**$_{\mathbf{IM}}$, **2**$_{\mathbf{im2}}$ and **2**$_{\boldsymbol{Dewar}}$ (B3LYP/6-311+G(d,p)). Hydrogens omitted for clarity. The B-N and C3-C6 distance is given in Å.

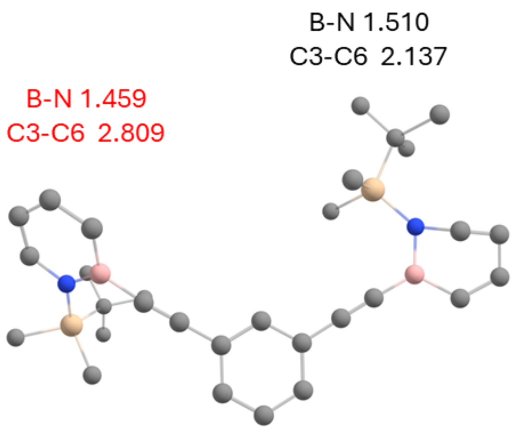

2$_{TS2.1}$

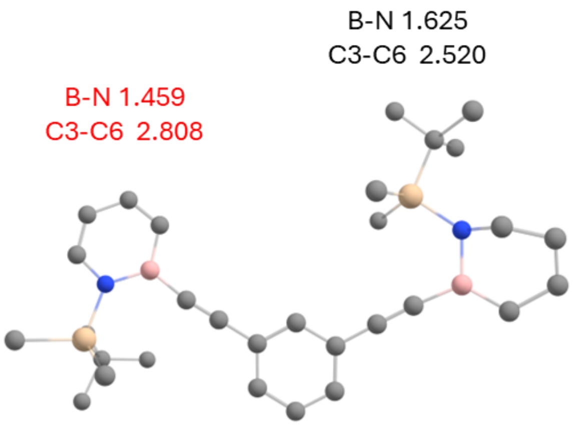

2$_{TS2.2}$

Figure S49. Calculated geometries of the transition states **2**$_{TS2.1}$ and **2**$_{TS2.2}$ (B3LYP/6-311+G(d,p)). Hydrogens omitted for clarity. The B-N and C3-C6 distance is given in Å.

Meta-Aza

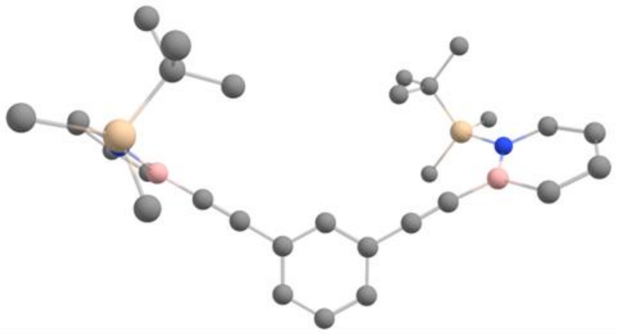

2

C	1.16674500	4.06692100	-0.54153300
C	1.16024500	2.66099700	-0.48488700
C	-0.02876900	1.99823700	-0.14395000
C	-1.19902700	2.71786600	0.14261000
C	-1.16745000	4.12359400	0.08746100
C	0.00882100	4.78278200	-0.25469700
H	2.08060700	4.58350800	-0.80788500
H	-0.04292900	0.91675200	-0.10029200
H	-2.06676100	4.68407700	0.31111700
H	0.02349400	5.86587800	-0.29798900
C	-2.40267400	2.03249100	0.48468700
C	2.34496800	1.91804400	-0.76844200
C	3.36086100	1.28541600	-0.98073300
C	-3.43304300	1.44475500	0.75008200
C	-5.22608500	1.07477200	2.64884700
C	-6.36077500	0.45175800	3.10210000
H	-4.71271300	1.76877400	3.30744400
C	-6.61450500	-0.73151000	1.00598900
C	-7.05859700	-0.46284300	2.27135600
H	-6.74914800	0.64325300	4.09971200
H	-7.17162000	-1.42555000	0.39004800
H	-7.95553400	-0.95798700	2.62239400
C	5.03254500	0.53479800	-2.87972100
C	6.14344300	-0.16889800	-3.26958600
H	4.47103600	1.08239400	-3.63059100
C	6.54214500	-0.90525600	-0.99874100
C	6.90249200	-0.89832400	-2.31805300
H	6.46589300	-0.18356200	-4.30812000
H	7.14373800	-1.46259000	-0.29234600
H	7.78069200	-1.45607400	-2.61899500
B	4.61864500	0.52440500	-1.42005100
B	-4.71881800	0.77435200	1.25075100
N	-5.49089800	-0.16450700	0.44521400
N	5.45026500	-0.23401600	-0.49316200
Si	5.14953200	-0.41229400	1.30664300
Si	-5.07961700	-0.69281900	-1.26158800

C	-6.70098500	-1.19864300	-2.09029700
H	-6.52003200	-1.32348500	-3.16208100
H	-7.12504800	-2.13581800	-1.72309700
H	-7.45775500	-0.41659200	-1.97790200
C	-3.87505200	-2.18776000	-1.19045900
C	-4.39382600	0.74857100	-2.25218300
H	-5.01724200	1.63715700	-2.11906600
H	-3.37237500	1.01511800	-1.98577700
H	-4.41781900	0.48408400	-3.31428000
C	-3.63596600	-2.68827900	-2.63387400
H	-3.18581700	-1.91794500	-3.26740100
H	-2.94699300	-3.54169500	-2.62149300
H	-4.56009100	-3.02367300	-3.11392600
C	-2.51884900	-1.79294800	-0.57161600
H	-2.02326000	-1.00187600	-1.14041900
H	-2.62240600	-1.44658300	0.45964400
H	-1.84905400	-2.66203800	-0.56347900
C	-4.48561700	-3.33465600	-0.35725300
H	-5.43491600	-3.68814500	-0.77100500
H	-3.79975600	-4.19077200	-0.34407300
H	-4.65774600	-3.03868500	0.68143300
C	6.82628600	-0.72772700	2.11896100
H	6.70950500	-0.64328500	3.20352700
H	7.25171200	-1.71283400	1.91550800
H	7.55615300	0.02705800	1.81141900
C	4.49148800	1.19078000	2.03255200
H	5.07484500	2.04414700	1.67581700
H	3.44570700	1.37982100	1.79574300
H	4.59855500	1.14847400	3.12128800
C	3.97211600	-1.90056600	1.60439900
C	2.58520100	-1.65811400	0.97513200
H	2.09816600	-0.76842000	1.38269500
H	2.64172300	-1.53879300	-0.10972500
H	1.93126600	-2.51502300	1.18024300
C	3.80131600	-2.08837200	3.12972400
H	3.13104800	-2.93396300	3.32695300
H	4.75083300	-2.30340900	3.62904300
H	3.36034900	-1.20748700	3.60602100
C	4.56991000	-3.19120700	1.00493400
H	5.54076400	-3.44102700	1.44344000
H	3.89932600	-4.03725500	1.20003000
H	4.69590700	-3.11855600	-0.07906200

Meta-TS1

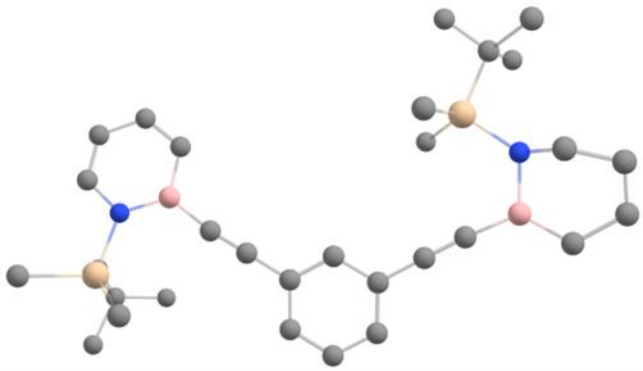

2$_{TS2.2}$

C	1.21720500	-2.53514900	-0.11524300
C	0.76052000	-3.86198000	-0.24093700
C	-0.60281800	-4.13211800	-0.28285700
C	-1.10305000	-1.76777500	-0.06880800
C	0.27418000	-1.49858000	-0.03235900
H	-0.94352600	-5.15643800	-0.38217100
H	0.61058300	-0.47436300	0.06462000
C	-2.04294900	-0.69958800	0.02443200
C	-2.84831200	0.20739500	0.11252200
C	-3.06301200	2.82443500	-0.06718800
C	-3.83441800	3.95777500	-0.03808100
H	-2.00010900	2.92150600	-0.26688000
C	-5.81878500	2.65856700	0.44434000
C	-5.22750300	3.87089300	0.21780400
H	-3.40075600	4.94046200	-0.20836600
H	-6.88168000	2.62276100	0.64513800
H	-5.84371000	4.76113500	0.24287000
B	-3.71051600	1.47248000	0.17320700
N	-5.14486800	1.45685800	0.44226800
Si	-6.15845300	-0.04003500	0.74148600
C	-5.20462400	-1.28036300	1.78115100
H	-5.89886800	-2.04713900	2.13961300
H	-4.39544000	-1.76945300	1.24203300
H	-4.77213500	-0.79030400	2.65806800
C	-6.73576600	-0.75958500	-0.94413700
C	-7.65155500	0.49217900	1.77020100
H	-8.38839000	1.08765200	1.22665200
H	-8.16337000	-0.40505600	2.13095600
H	-7.33682500	1.06160100	2.64974500
C	-7.68460000	-1.94909400	-0.66856800
H	-8.57630300	-1.64712200	-0.11128200
H	-8.02465000	-2.38015800	-1.61818300
H	-7.19066900	-2.74883000	-0.10814200
C	-7.49820200	0.31162700	-1.75271700

H	-8.38267700	0.67905800	-1.22357000
H	-6.86497600	1.17058400	-1.99180100
H	-7.84325800	-0.11495700	-2.70266000
C	-5.54405600	-1.25987000	-1.78504400
H	-4.98231000	-2.04634300	-1.27433800
H	-5.90864000	-1.67741500	-2.73202100
H	-4.84386000	-0.45668800	-2.02661900
H	1.48265200	-4.66647100	-0.30554300
C	2.61284800	-2.25526600	-0.07192000
C	3.80915400	-2.03555400	-0.03268400
C	6.28144800	-2.95991200	0.19789500
C	7.66713700	-2.61357200	0.42384400
H	5.97303500	-4.00011900	0.17458400
C	6.57302000	-0.61152900	1.06392600
C	7.85717900	-1.34393900	0.82809500
H	8.48801300	-3.28472300	0.18637200
H	6.32363200	-0.24507900	2.06846800
H	8.78053000	-0.77472500	0.78513300
B	5.32002600	-1.86102600	0.00575100
N	5.82335700	-0.31697100	0.01354700
Si	4.77817200	1.15164300	-0.17167800
C	3.67749400	1.36951400	1.34086600
H	3.02394500	2.23815300	1.21703900
H	3.04503200	0.48952200	1.47892600
H	4.25145600	1.51939700	2.25954300
C	6.01877800	2.61362100	-0.34084200
C	3.79048500	0.92906700	-1.74914500
H	4.44052800	0.65504900	-2.58395500
H	3.03828500	0.14711500	-1.63953800
H	3.27852100	1.85950900	-2.01174500
C	6.90065900	2.43171800	-1.59431900
H	6.30563100	2.40340600	-2.51137900
H	7.60090000	3.27146200	-1.68490300
H	7.48867900	1.51110000	-1.54970800
C	5.20770200	3.92287000	-0.48212300
H	4.55018000	3.90874000	-1.35620400
H	4.59309200	4.12512600	0.39997800
H	5.89140100	4.77165200	-0.60451300
C	6.92577100	2.73154600	0.89984700
H	7.59035800	3.59800400	0.79550900
H	6.35423800	2.87061300	1.82231700
H	7.57187800	1.85719700	1.02831400
C	-1.53382400	-3.10161900	-0.19700400
H	-2.59409500	-3.31870100	-0.23086100

Meta-im2

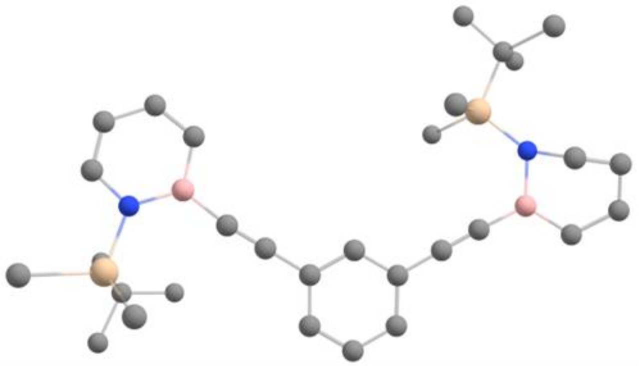

$\mathbf{2}_{im2}$

C	-1.18834800	-2.54562500	-0.01123000
C	-0.73618000	-3.87842700	0.02282000
C	0.62591500	-4.15639000	-0.00411700
C	1.13254400	-1.78553200	-0.10321600
C	-0.24364100	-1.51038300	-0.07312200
H	0.96419100	-5.18603100	0.02401400
H	-0.57768400	-0.48120300	-0.09883500
C	2.07319700	-0.71680400	-0.16818200
C	2.87428300	0.19586900	-0.22735000
C	3.05304700	2.81765000	-0.07469700
C	3.81458500	3.95670000	-0.07394700
H	1.97938100	2.90585000	0.05909600
C	5.83644100	2.67147100	-0.41741500
C	5.22177300	3.87980300	-0.24445900
H	3.36301900	4.93752400	0.05564900
H	6.90991100	2.64237600	-0.55284600
H	5.83014700	4.77580000	-0.24510100
B	3.72498900	1.46854800	-0.25684100
N	5.17351400	1.46288500	-0.43892500
Si	6.20847000	-0.02881900	-0.65232700
C	5.34079400	-1.27918300	-1.75413500
H	6.05763600	-2.05268000	-2.04800000
H	4.48717200	-1.75785000	-1.27808900
H	4.98155600	-0.79731700	-2.66792600
C	6.67028300	-0.73881400	1.07270200
C	7.77325200	0.50110400	-1.57285500
H	8.46979200	1.09655700	-0.97828100
H	8.31010300	-0.39582300	-1.89590400
H	7.52459200	1.07056700	-2.47326600
C	7.65469700	-1.91407300	0.87176100
H	8.58083100	-1.60061000	0.38079800
H	7.93064000	-2.33861500	1.84479000
H	7.21547500	-2.72290100	0.27954100
C	7.35503800	0.34649100	1.93072400

H	8.27050600	0.72636500	1.46675400
H	6.69310800	1.19622300	2.11902900
H	7.63661500	-0.07122500	2.90521800
C	5.42867100	-1.25570400	1.82705000
H	4.91788400	-2.05309800	1.28097200
H	5.73018300	-1.66439100	2.79975700
H	4.69858600	-0.46484000	2.01322600
H	-1.46146400	-4.68122200	0.07128800
C	-2.58368400	-2.25586800	0.01826100
C	-3.77577600	-2.02065300	0.04072300
C	-6.24951700	-2.94819100	0.04038000
C	-7.64959000	-2.67089000	-0.15119000
H	-5.88437900	-3.97402500	0.03483800
C	-6.47878200	-0.86009700	-0.94389200
C	-7.81467900	-1.40793600	-0.60702400
H	-8.44384300	-3.34065100	0.16519500
H	-6.09917800	-0.85438900	-1.96968200
H	-8.68373400	-0.77062200	-0.48284500
B	-5.28623100	-1.79083800	0.12067000
N	-5.81028100	-0.30751100	0.10883900
Si	-4.82532300	1.18206200	0.17206500
C	-3.74349700	1.35594600	-1.36512800
H	-3.11367200	2.24842200	-1.30069700
H	-3.08570000	0.48944300	-1.46742200
H	-4.33313800	1.43792800	-2.28291300
C	-6.08731400	2.63188400	0.26875600
C	-3.78598200	1.07832500	1.73186100
H	-4.41074000	0.90666100	2.61207200
H	-3.06922100	0.25728700	1.66664700
H	-3.22489100	2.00394100	1.89075500
C	-6.94551800	2.51006500	1.54503300
H	-6.33717100	2.56973400	2.45216500
H	-7.67615100	3.32781600	1.58826500
H	-7.49587400	1.56616500	1.57523900
C	-5.31503700	3.96980500	0.30792300
H	-4.64477200	4.03345600	1.17054200
H	-4.71788900	4.12874900	-0.59496000
H	-6.01964000	4.80742000	0.38325500
C	-7.01519200	2.63379700	-0.96322900
H	-7.71508200	3.47706500	-0.90671900
H	-6.45943700	2.73738500	-1.90011900
H	-7.61789700	1.72284000	-1.02695200
C	1.55847600	-3.12619800	-0.06719500
H	2.61818200	-3.34848500	-0.08707900

Meta-TS2.1

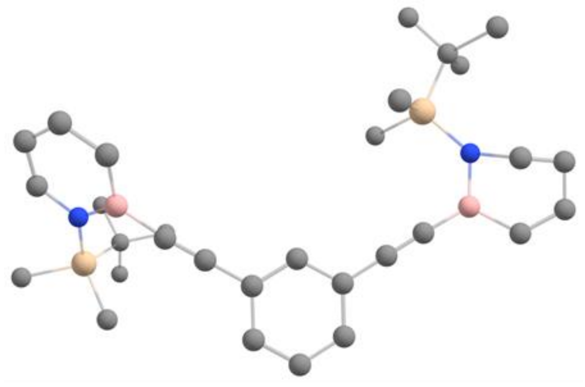

$\mathbf{2}_{TS2.1}$

C	1.12210900	2.69142400	-0.46083100
C	0.76849300	4.05231400	-0.52176400
C	-0.55372200	4.42158500	-0.74275500
C	-1.21316000	2.08883800	-0.84694300
C	0.12290600	1.72079300	-0.62703100
H	-0.81705400	5.47216500	-0.79065900
H	0.38417000	0.67125700	-0.58500900
C	-2.21610800	1.08898900	-1.01205000
C	-3.07901600	0.24297000	-1.14155600
C	-3.59956900	-2.06665200	-2.30172200
C	-4.45866100	-3.10918600	-2.53191600
H	-2.61888200	-2.08367300	-2.76685400
C	-6.15608200	-2.09284000	-1.13793100
C	-5.74977900	-3.12052500	-1.94197600
H	-4.17421800	-3.94461500	-3.16743100
H	-7.14692200	-2.12437200	-0.70338000
H	-6.43406400	-3.94063500	-2.12113200
B	-4.03995300	-0.91967100	-1.41085700
N	-5.38034800	-0.99300200	-0.83916000
Si	-6.10575400	0.22296500	0.31774100
C	-5.61599700	1.97461800	-0.15683000
H	-6.29434400	2.67604600	0.33952000
H	-4.59411200	2.22436300	0.12381400
H	-5.71262900	2.12532100	-1.23543800
C	-5.58899900	-0.22516300	2.11290400
C	-7.98466100	0.11916800	0.12972800
H	-8.43165400	-0.79317400	0.53066400
H	-8.43808900	0.96094100	0.66157100
H	-8.27717100	0.20503500	-0.92105700
C	-6.27671200	0.76492700	3.08109600
H	-7.36814600	0.71291500	3.02163900
H	-5.99653900	0.52960800	4.11513300
H	-5.97661800	1.80031000	2.89289200
C	-6.03911500	-1.66008100	2.46057700

H	-7.12416200	-1.78344200	2.38978800
H	-5.57020900	-2.40459300	1.81112300
H	-5.75361000	-1.90054200	3.49212200
C	-4.06145700	-0.12556600	2.30153700
H	-3.68270200	0.87825600	2.09294600
H	-3.80060900	-0.36668900	3.33973400
H	-3.52114800	-0.82212200	1.65606500
H	1.53739500	4.80437500	-0.39518700
C	2.47633000	2.30641700	-0.23615400
C	3.63369300	1.98813500	-0.04727200
C	6.16943000	2.71325100	0.13869500
C	7.58060700	2.42220100	0.35289200
H	5.86322900	3.70418700	-0.19827100
C	6.49042000	0.73795700	-0.61032000
C	7.78959100	1.14668700	-0.01464700
H	8.28170000	3.10635300	0.82077500
H	6.32708400	0.73304100	-1.68988300
H	8.58270000	0.45967900	0.25693900
B	5.09619400	1.63942900	0.24080800
N	5.56699200	0.20466400	0.28187200
Si	4.62068100	-1.29716800	0.20155100
C	3.76215100	-1.46270200	-1.47266400
H	3.14458400	-2.36507800	-1.51367600
H	3.10948000	-0.60339700	-1.64787100
H	4.47329500	-1.51450900	-2.30240800
C	5.84686900	-2.75430500	0.46955700
C	3.35854100	-1.19787900	1.58929600
H	3.84301300	-1.06757600	2.56030200
H	2.68559500	-0.35103000	1.43652400
H	2.75017700	-2.10602400	1.63332100
C	6.53490400	-2.62789500	1.84475000
H	5.81486200	-2.68152400	2.66655200
H	7.25179700	-3.44683700	1.98584400
H	7.07925900	-1.68494700	1.94189000
C	5.07000400	-4.08888500	0.41798300
H	4.29638400	-4.14453100	1.18987300
H	4.58972800	-4.24992700	-0.55182800
H	5.75502200	-4.92956500	0.58488400
C	6.92553200	-2.76945700	-0.63328900
H	7.61108800	-3.61247100	-0.47968700
H	6.49370500	-2.88159500	-1.63238200
H	7.53366700	-1.85970200	-0.62926500
C	-1.54211000	3.45584600	-0.90337300
H	-2.57034900	3.74722700	-1.07927100

Meta-IM

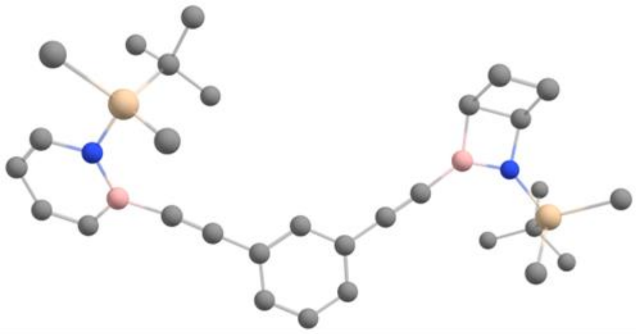

$\mathbf{2}_{IM}$

C	1.13693000	-3.16322600	0.51050700
C	0.80758300	-1.81495700	0.27866900
C	-0.54281100	-1.45063400	0.16342300
C	-1.56035100	-2.41021800	0.27429900
C	-1.20589400	-3.75390400	0.50214000
C	0.13131600	-4.11814800	0.61935000
H	2.17786000	-3.44779400	0.60072600
H	-0.79960700	-0.41497900	-0.01768300
H	-1.98703200	-4.49933000	0.58654900
H	0.39169400	-5.15529700	0.79634000
C	-2.93073700	-2.03493500	0.15883400
C	1.82915900	-0.82823800	0.16003200
C	2.70394800	0.01104700	0.06060200
C	-4.09969900	-1.71182100	0.07312800
C	-6.48021900	-2.64244900	-0.55713700
C	-7.82399500	-2.43893000	-0.74118700
H	-6.07112600	-3.63015500	-0.74707400
C	-7.61662000	-0.11830300	-0.08514700
C	-8.39503300	-1.16162500	-0.50528800
H	-8.47708400	-3.24443500	-1.06872000
H	-8.08012500	0.84286900	0.09557400
H	-9.45516600	-0.99283000	-0.64843800
C	3.66661800	2.66307100	-0.52738400
C	3.77215700	3.67926700	0.60005400
H	3.20844900	2.96852800	-1.46911100
C	5.22805800	2.55256100	-0.37681300
C	5.10100300	3.56746800	0.75022800
H	3.01137900	4.24793500	1.12392500
H	5.89694600	2.76398200	-1.21186500
H	5.81564700	3.97025200	1.45801800
B	3.73444800	1.10854700	-0.10757500
B	-5.60599800	-1.48834500	-0.10229300
N	-6.25961600	-0.20613900	0.13544200
N	5.14637900	1.10337200	0.01774300
Si	6.45605800	0.01705600	0.52247500
Si	-5.42536500	1.33472100	0.67399800

C	-6.71770900	2.38358300	1.56840300
H	-6.20584200	3.20538800	2.07792900
H	-7.47226300	2.82805000	0.91560600
H	-7.23395800	1.79806400	2.33488700
C	-4.76511900	2.26771000	-0.87025400
C	-4.08235100	0.95004100	1.92983600
H	-4.46911700	0.29168900	2.71291100
H	-3.20394300	0.47311200	1.49868000
H	-3.77153100	1.88469800	2.40766300
C	-4.19544500	3.63005800	-0.41119500
H	-3.36846400	3.51359400	0.29604600
H	-3.80683200	4.18032800	-1.27685000
H	-4.95641300	4.25978900	0.05913300
C	-3.64636000	1.47495800	-1.57569500
H	-2.78953600	1.30334100	-0.91887900
H	-3.99164500	0.50149200	-1.93205500
H	-3.28572900	2.03765600	-2.44588900
C	-5.90802900	2.51694800	-1.87736100
H	-6.71993500	3.10986800	-1.44523500
H	-5.52655600	3.07307700	-2.74258800
H	-6.33358800	1.58191300	-2.25216500
C	7.79147000	1.09585100	1.30435700
H	8.62899500	0.48749500	1.65765800
H	8.19133200	1.83583300	0.60534900
H	7.38821700	1.63446900	2.16688300
C	5.75982100	-1.17966300	1.79816200
H	5.43190800	-0.63385800	2.68756800
H	4.89841500	-1.72861600	1.41092800
H	6.51445700	-1.90729400	2.11130800
C	7.14609400	-0.92563200	-1.00570000
C	6.05283000	-1.83782400	-1.59972800
H	5.73079300	-2.60535000	-0.88981500
H	5.16812300	-1.27070500	-1.90308400
H	6.43487100	-2.35480000	-2.48929400
C	8.35143900	-1.79421500	-0.58464400
H	8.73789800	-2.34687200	-1.45036200
H	9.17526000	-1.19148700	-0.19052200
H	8.08138600	-2.53295400	0.17639900
C	7.60354600	0.07389400	-2.08863400
H	8.39350400	0.74014600	-1.72817300
H	8.00452400	-0.46630100	-2.95576500
H	6.77493100	0.69255400	-2.44523600

Meta-Dewar

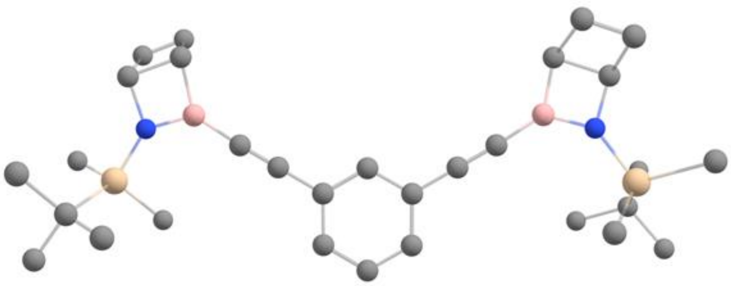

2$_{Dewar}$

C	1.20695100	-2.22735500	0.03803700
C	1.21925700	-0.81988900	0.03756000
C	0.00000300	-0.12665900	-0.00002900
C	-1.21924600	-0.81989800	-0.03760600
C	-1.20693000	-2.22736500	-0.03806500
C	0.00001300	-2.91758200	-0.00000900
H	2.14623300	-2.76552700	0.06760400
H	-0.00000100	0.95570700	-0.00003500
H	-2.14620700	-2.76554400	-0.06762400
H	0.00001800	-4.00135200	-0.00000100
C	-2.45169800	-0.10517600	-0.07435200
C	2.45170400	-0.10515800	0.07429900
C	3.50608200	0.49997500	0.10832800
C	-3.50608000	0.49994900	-0.10839300
C	-5.09956700	2.89866100	0.01040900
C	-5.40766900	3.64364000	-1.28000700
H	-4.76106100	3.46721000	0.87768600
C	-6.58223200	2.38702300	-0.10302500
C	-6.66428300	3.18707100	-1.39514700
H	-4.78853100	4.27898600	-1.90410300
H	-7.31089200	2.57324900	0.68670300
H	-7.43008700	3.27301800	-2.15674300
C	5.09958900	2.89867800	-0.01041300
C	5.40767200	3.64363900	1.28001500
H	4.76110100	3.46723600	-0.87769200
C	6.58224800	2.38702500	0.10304100
C	6.66428700	3.18707400	1.39516400
H	4.78852600	4.27898200	1.90410800
H	7.31091900	2.57324800	-0.68667700
H	7.43008200	3.27301700	2.15676900
B	4.77642700	1.32602700	0.12420400
B	-4.77642000	1.32600900	-0.12422700
N	-6.13994900	0.95480500	-0.23161700
N	6.13995000	0.95481100	0.23161900
Si	7.13062300	-0.48962100	0.51898600
Si	-7.13064400	-0.48961400	-0.51897100
C	-8.64787200	0.06896200	-1.49054300
H	-9.29964000	-0.77906900	-1.71953800

H	-9.24334700	0.80656000	-0.94521700
H	-8.34651400	0.51931400	-2.44082100
C	-7.64501000	-1.27135300	1.16159600
C	-6.11307000	-1.69747300	-1.54355600
H	-5.87949600	-1.26100400	-2.51893000
H	-5.16688600	-1.94530300	-1.05714600
H	-6.66131500	-2.62840600	-1.71607400
C	-8.59046800	-2.46732800	0.91562100
H	-8.12051800	-3.24608500	0.30688500
H	-8.87103000	-2.92792700	1.87134300
H	-9.51640000	-2.16442200	0.41745800
C	-6.39437800	-1.76745400	1.91678600
H	-5.86885300	-2.55299100	1.36580400
H	-5.68316600	-0.95808000	2.10524500
H	-6.68251000	-2.18696900	2.88916600
C	-8.37349700	-0.22926700	2.03591900
H	-9.28122800	0.15003000	1.55637700
H	-8.67425200	-0.68101500	2.98983400
H	-7.73096100	0.62481000	2.26862300
C	8.64783400	0.06892600	1.49059900
H	9.29958200	-0.77911800	1.71960300
H	9.24333500	0.80651800	0.94529400
H	8.34646000	0.51927500	2.44087300
C	6.11300700	-1.69747400	1.54353600
H	5.87942500	-1.26101400	2.51891200
H	5.16682600	-1.94527700	1.05710600
H	6.66122900	-2.62842200	1.71605100
C	7.64501900	-1.27135300	-1.16157500
C	6.39439600	-1.76743400	-1.91679400
H	5.86885200	-2.55296900	-1.36582900
H	5.68319700	-0.95805100	-2.10526200
H	6.68254300	-2.18694500	-2.88917200
C	8.59045900	-2.46734000	-0.91559000
H	8.87103100	-2.92793800	-1.87130900
H	9.51638600	-2.16444500	-0.41741200
H	8.12049200	-3.24609400	-0.30686400
C	8.37353600	-0.22926900	-2.03587500
H	9.28126000	0.15001500	-1.55631000
H	8.67430800	-0.68101300	-2.98978600
H	7.73101500	0.62481800	-2.26858800

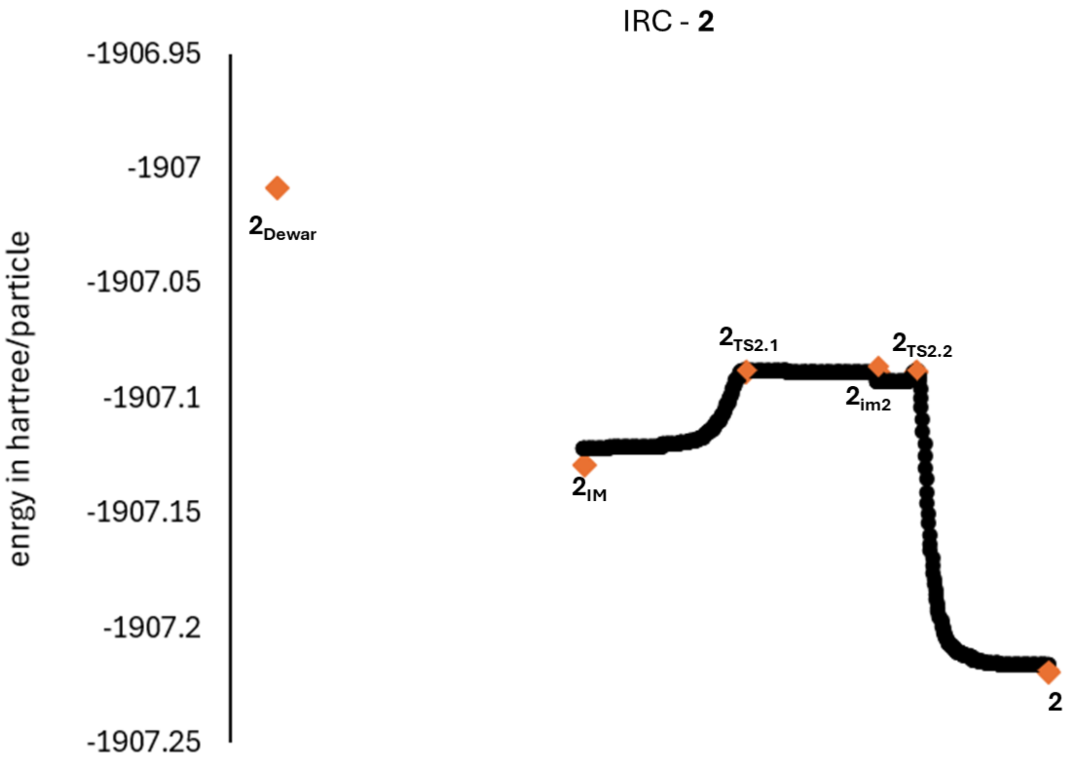

Figure S50. Intrinsic reaction coordinates calculated in both directions from the transition states $\mathbf{2}_{TS2.1}$ and $\mathbf{2}_{TS2.2}$. The corresponding calculated geometries of the transition states as well as the local minima are given in orange. The offset of the IRC's at the intermediate $\mathbf{1}_{im2}$ is due to different rotamers along the C-C triple bond or the *tert*-butyldimethylsilyl group.

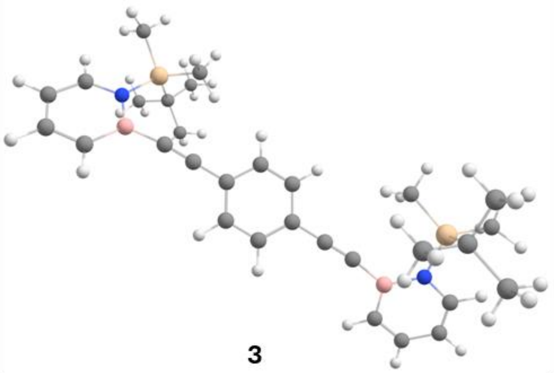

3

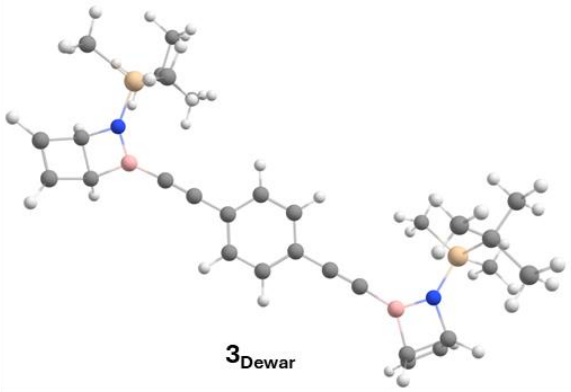

3_{Dewar}

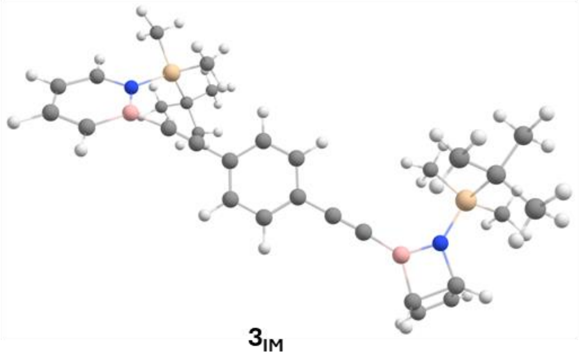

3_{IM}

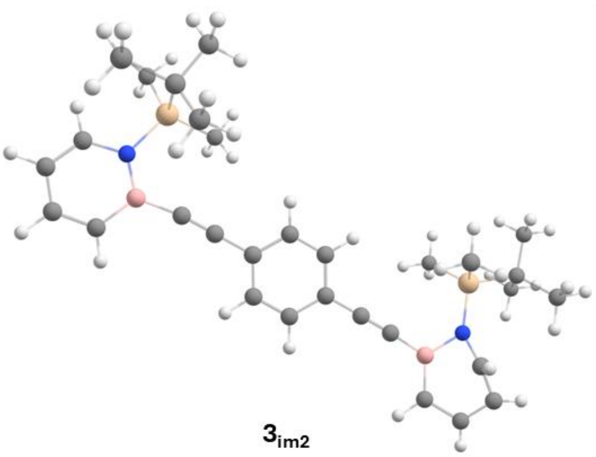

3_{im2}

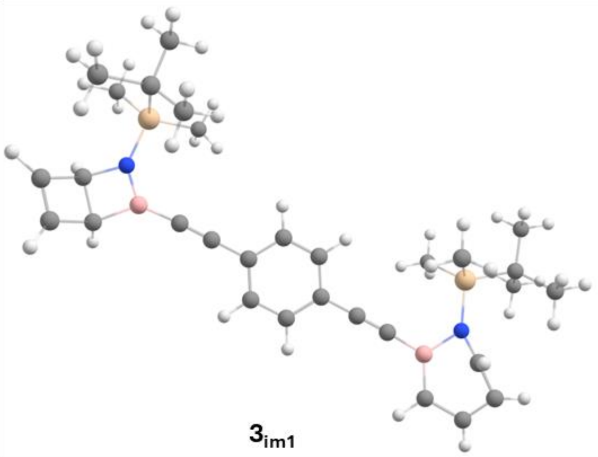

3_{im1}

Figure S51. Calculated geometries of the local minima **3**, **3_{im1}**, **3_{IM}**, **3_{im2}** and **3_{Dewar}** (B3LYP/6-311+G(d,p)).

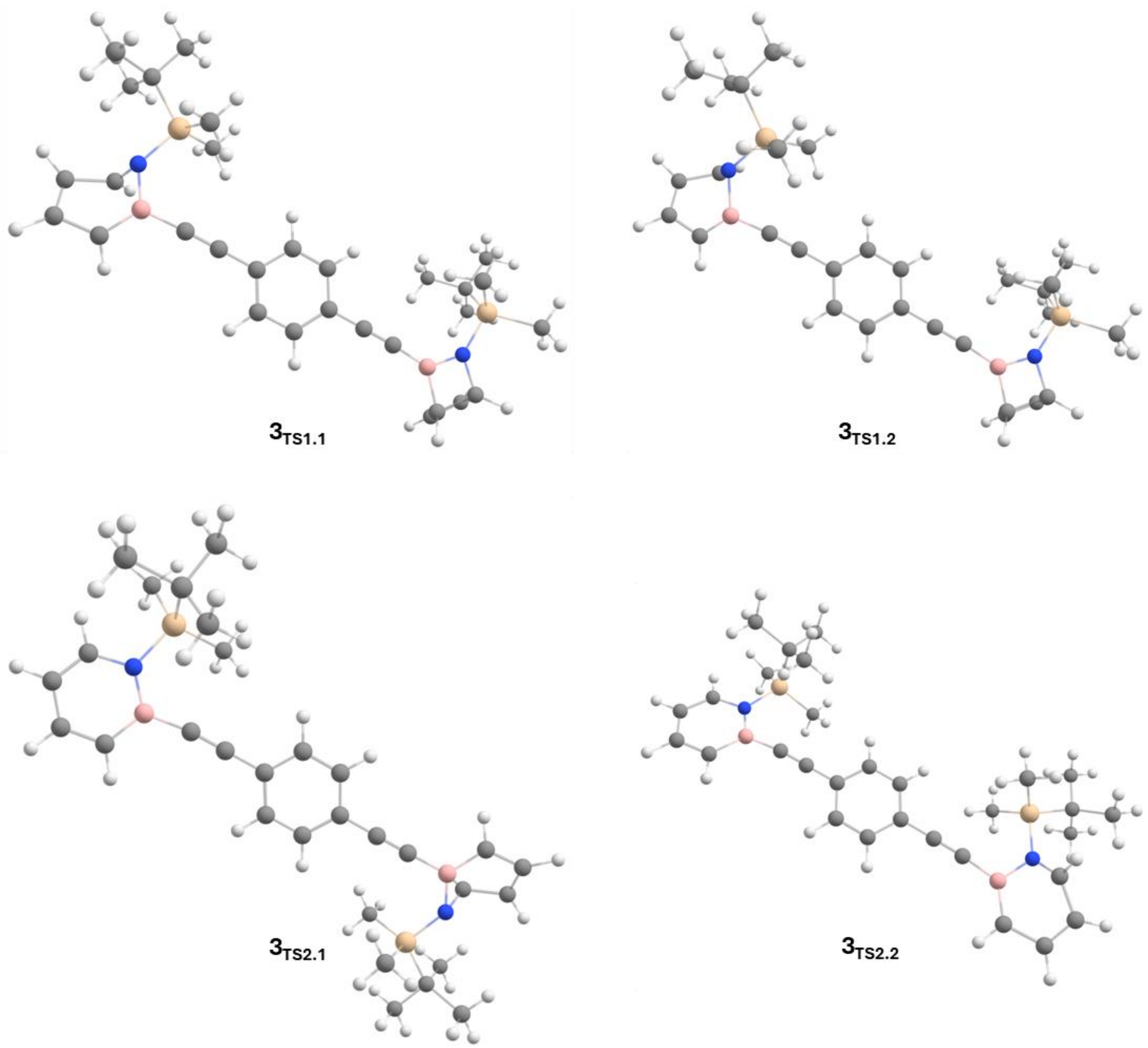

Figure S52. Calculated geometries of the transition states $\mathbf{3_{TS1.1}}$, $\mathbf{3_{TS1.2}}$, $\mathbf{3_{TS2.1}}$ and $\mathbf{3_{TS2.2}}$ (B3LYP/6-311+G(d,p)).

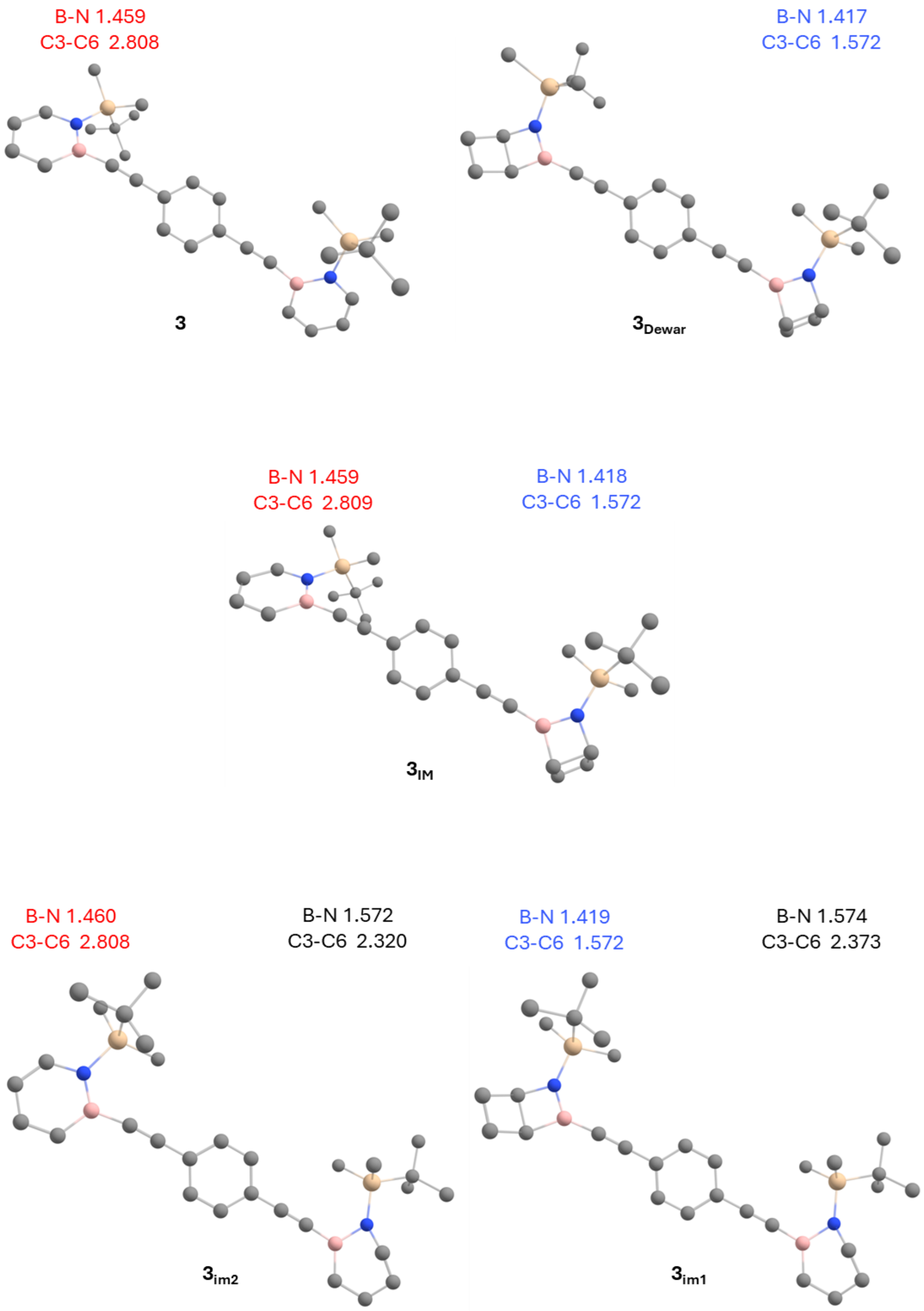

Figure S53. Calculated geometries of the local minima **3**, $\mathbf{3_{im1}}$, $\mathbf{3_{IM}}$, $\mathbf{3_{im2}}$ and $\mathbf{3}_{\boldsymbol{Dewar}}$ (B3LYP/6-311+G(d,p)). Hydrogens omitted for clarity. The B-N and C3-C6 distance is given in Å.

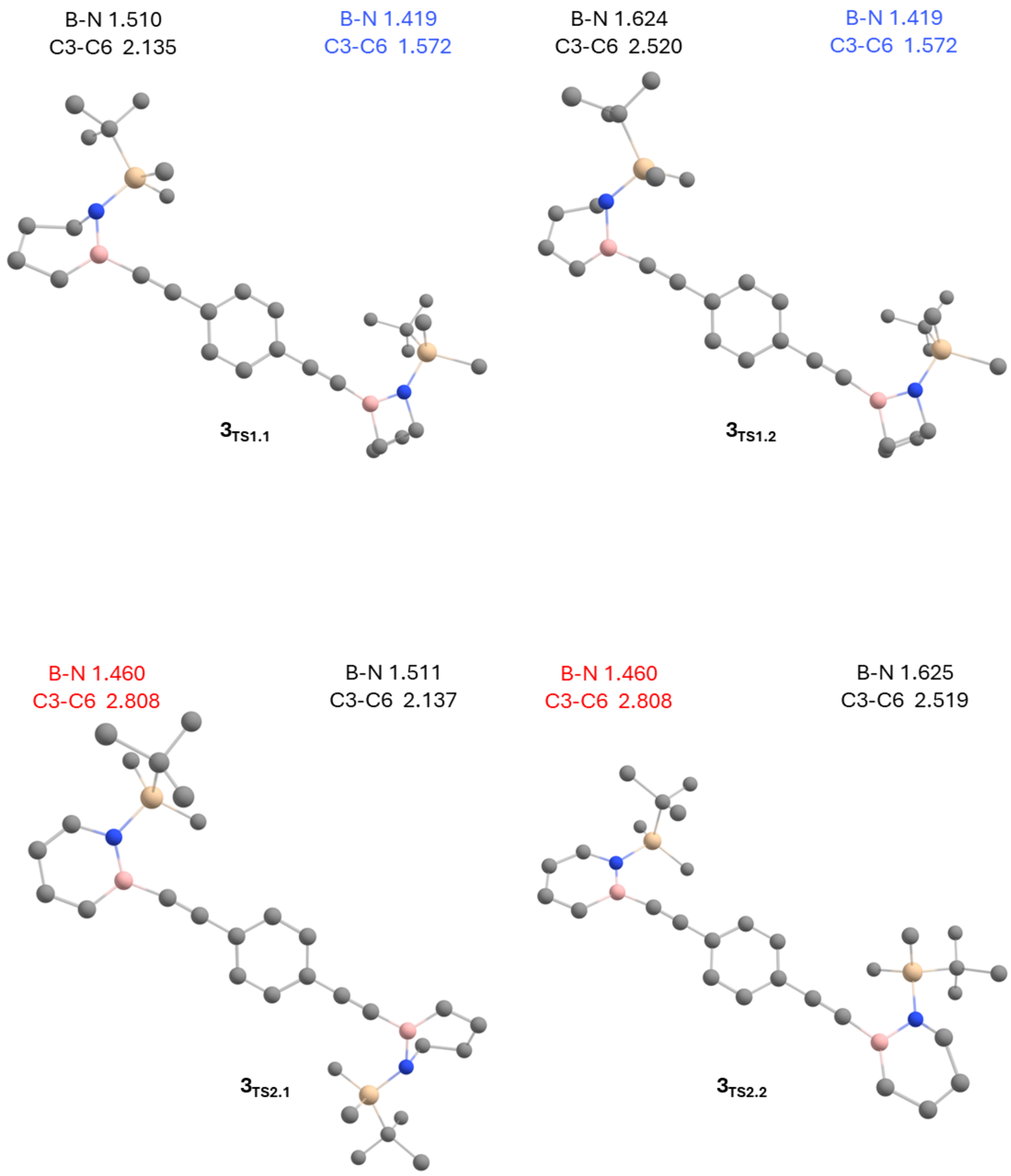

Figure S54. Calculated geometries of the transition states **3**$_{TS1.1}$, **3**$_{TS1.2}$, **3**$_{TS2.1}$ and **3**$_{TS2.2}$ (B3LYP/6-311+G(d,p)). Hydrogens omitted for clarity. The B-N and C3-C6 distance is given in Å.

Para-Aza

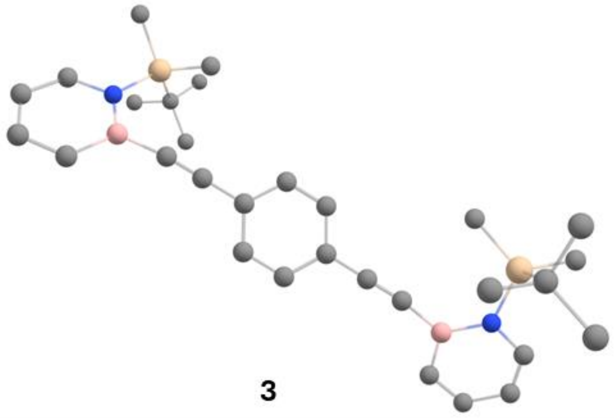

3

C	1.38794600	-0.99308600	-0.27521300
C	0.67916500	-2.20269400	-0.13682400
C	-0.67916500	-2.20269400	0.13682400
C	-1.38794600	-0.99308600	0.27521100
C	-0.68011000	0.21603700	0.13347200
C	0.68011000	0.21603700	-0.13347400
H	-1.20994700	-3.14085000	0.24409700
H	-1.20781200	1.15595800	0.24037700
H	1.20781100	1.15595900	-0.24038000
C	-2.78365000	-1.00032900	0.55274300
C	-3.97816600	-1.00559300	0.78375200
C	-5.87651900	-2.46395300	1.87847900
C	-7.19637200	-2.64524200	2.20361700
H	-5.16131900	-3.23414300	2.15101500
C	-7.78834200	-0.51801100	1.21200000
C	-8.16062400	-1.66070800	1.86493500
H	-7.53163000	-3.53914600	2.72437800
H	-8.54405000	0.21771200	0.96900400
H	-9.20460000	-1.79594900	2.11929900
B	-5.45224100	-1.19924800	1.15394500
N	-6.49418700	-0.22743500	0.83909400
Si	-6.25163600	1.33375100	-0.08774100
C	-7.62360800	2.51854800	0.44563400
H	-7.39047100	3.51679700	0.06330100
H	-8.61816800	2.25627900	0.07830200
H	-7.67311900	2.59208600	1.53610900
C	-6.37155700	0.97158000	-1.97054400
C	-4.62306900	2.14149400	0.38820700
H	-4.48962900	2.13647600	1.47338300
H	-3.75813200	1.65324200	-0.05744300
H	-4.64133000	3.18549300	0.05946200
C	-6.25730300	2.31211200	-2.73282400
H	-5.30223000	2.81215800	-2.54493400
H	-6.32229100	2.13178900	-3.81282000
H	-7.06045500	3.00746800	-2.47091000
C	-5.23900000	0.03533300	-2.43899700

H	-4.24870100	0.45839200	-2.25129900
H	-5.28133700	-0.93944500	-1.94697100
H	-5.32432300	-0.13594500	-3.51946900
C	-7.72798600	0.31712500	-2.30861600
H	-8.57527400	0.95338700	-2.03551400
H	-7.79447200	0.13533300	-3.38841100
H	-7.85273300	-0.64698800	-1.80765900
H	1.20994700	-3.14085000	-0.24409700
C	2.78365000	-1.00032900	-0.55274400
C	3.97816600	-1.00559200	-0.78375400
C	5.87651900	-2.46395100	-1.87848300
C	7.19637200	-2.64523800	-2.20362100
H	5.16131900	-3.23414000	-2.15101900
C	7.78834200	-0.51800800	-1.21200100
C	8.16062400	-1.66070400	-1.86493800
H	7.53163100	-3.53914100	-2.72438300
H	8.54404900	0.21771500	-0.96900500
H	9.20460000	-1.79594500	-2.11930200
B	5.45224100	-1.19924700	-1.15394700
N	6.49418700	-0.22743400	-0.83909400
Si	6.25163500	1.33375000	0.08774300
C	4.62306800	2.14149400	-0.38820300
H	4.48962700	2.13647700	-1.47337800
H	3.75813200	1.65324100	0.05744700
H	4.64132800	3.18549300	-0.05945600
C	6.37155700	0.97157700	1.97054500
C	7.62360600	2.51854900	-0.44563100
H	7.67311700	2.59208900	-1.53610600
H	7.39046900	3.51679700	-0.06329700
H	8.61816700	2.25628000	-0.07830000
C	6.25730300	2.31210700	2.73282800
H	5.30222900	2.81215300	2.54493900
H	6.32229100	2.13178300	3.81282300
H	7.06045400	3.00746500	2.47091500
C	5.23900200	0.03532800	2.43899800
H	4.24870200	0.45838700	2.25130000
H	5.28133900	-0.93944900	1.94697000
H	5.32432500	-0.13595100	3.51946900
C	7.72798700	0.31712300	2.30861600
H	8.57527400	0.95338600	2.03551500
H	7.79447400	0.13532900	3.38841000
H	7.85273500	-0.64698900	1.80765700

Para-TS2.2

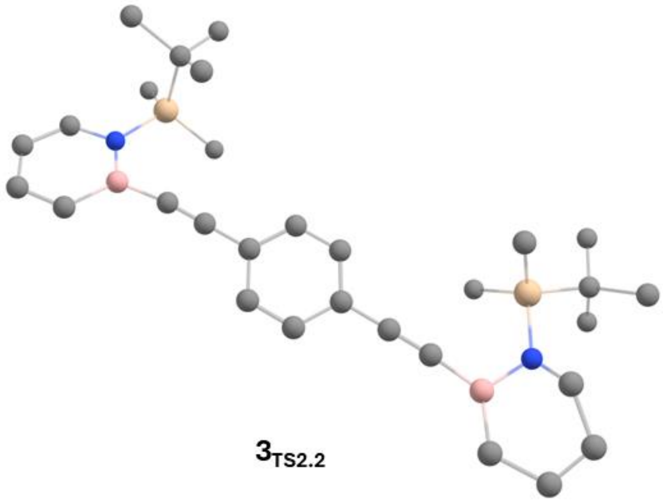

$3_{TS2.2}$

C	0.92499994	0.54999999	0.00000000
C	1.60166194	-0.68410801	0.06856000
C	2.98623094	-0.73759501	0.04613100
C	3.75424294	0.43953499	-0.04596400
C	3.07729594	1.67386699	-0.11189400
C	1.69349994	1.72857399	-0.08942300
H	3.48834794	-1.69579901	0.10169200
H	3.65370894	2.58836999	-0.18166600
H	1.18811694	2.68521999	-0.14142000
C	5.17542394	0.39226199	-0.07311200
C	6.39134094	0.35234699	-0.10461900
C	8.52304094	1.87716899	0.12643700
C	9.88688194	2.01495999	0.16012900
H	7.90580194	2.75964299	0.26530200
C	10.18924494	-0.35521201	-0.22765200
C	10.72668894	0.88446599	-0.01580600
H	10.35246694	2.98461999	0.32031400
H	10.85352394	-1.19813301	-0.36761700
H	11.80473994	0.98486499	0.00924300
B	7.91503294	0.50393799	-0.09732500
N	8.83682194	-0.61246801	-0.28416900
Si	8.35516294	-2.35885701	-0.55311600
C	6.84802994	-2.45860701	-1.67029800
H	6.72326394	-3.49455101	-2.00161200
H	5.92722694	-2.13414901	-1.18901800
H	6.99283394	-1.84166001	-2.56171300
C	8.08572794	-3.21218001	1.14776800
C	9.77314494	-3.19271101	-1.48322000
H	10.67040494	-3.36308801	-0.88409500
H	9.43032294	-4.16936501	-1.83765100
H	10.05858894	-2.61004301	-2.36403600
C	7.80353294	-4.71246101	0.90186400
H	8.63803994	-5.21483401	0.40372200
H	7.64199094	-5.22181201	1.85984500

H	6.90533594	-4.86835601	0.29633200
C	9.35054594	-3.07720701	2.02219300
H	10.22919794	-3.53107001	1.55390500
H	9.58544894	-2.03197801	2.24178400
H	9.19582394	-3.58640401	2.98149800
C	6.89130994	-2.60049701	1.90729500
H	5.95654394	-2.69565001	1.34856500
H	6.75695394	-3.11925401	2.86488200
H	7.04012294	-1.54014701	2.12456200
H	1.02844594	-1.60050801	0.14011600
C	-0.49415106	0.61323599	0.01910700
C	-1.70964906	0.68748599	0.03314500
C	-3.88823906	2.18782299	0.16713200
C	-5.32215606	2.19468099	0.35301100
H	-3.33565206	3.12109599	0.13073200
C	-4.76623706	0.00719699	1.07319800
C	-5.82655706	1.02100099	0.77691600
H	-5.94788006	3.03801499	0.07404400
H	-4.64066306	-0.37653201	2.09410900
H	-6.85906106	0.69153299	0.71580400
B	-3.21715006	0.88373299	0.03043500
N	-4.08150106	-0.49191101	0.05621200
Si	-3.42023006	-2.17421501	-0.05814400
C	-2.43886806	-2.60274401	1.49161800
H	-2.01563806	-3.60849201	1.41068200
H	-1.61369906	-1.89900401	1.62362100
H	-3.05178806	-2.57713501	2.39690500
C	-4.97244106	-3.29989401	-0.22617500
C	-2.37033006	-2.25016601	-1.60933600
H	-2.92031606	-1.87288401	-2.47513700
H	-1.46307206	-1.65434901	-1.50345600
H	-2.07740606	-3.28304901	-1.81988300
C	-5.75658506	-2.95312901	-1.50959600
H	-5.15194006	-3.09776101	-2.40922300
H	-6.63542506	-3.60379401	-1.59854400
H	-6.10788906	-1.91785501	-1.50691900
C	-4.49867306	-4.76998101	-0.30899500
H	-3.83969106	-4.94375001	-1.16470800
H	-3.96940806	-5.08539001	0.59503600
H	-5.36456206	-5.43257601	-0.42734800
C	-5.90888106	-3.15538601	0.98973200
H	-6.75945306	-3.84069701	0.88927500
H	-5.40856806	-3.39561001	1.93261200
H	-6.32909206	-2.14798601	1.07434900

Para-im2

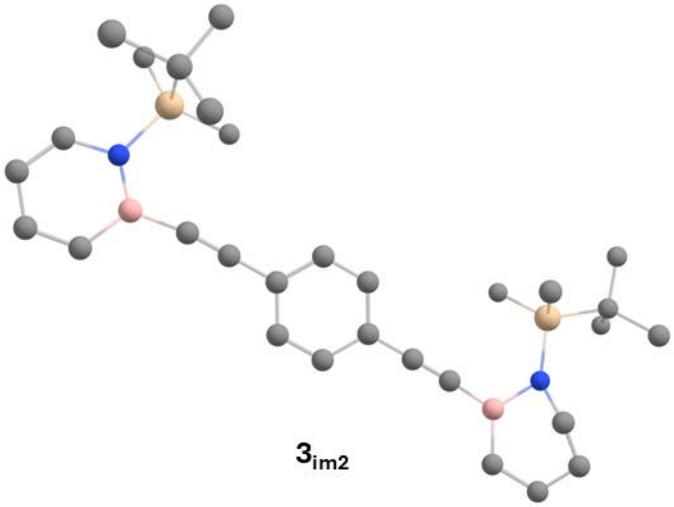

C	1.27230400	-1.57609800	-0.05278300
C	0.60057400	-0.33866900	-0.06564100
C	-0.78336400	-0.28172000	-0.10722900
C	-1.55492100	-1.45911700	-0.13874100
C	-0.88235500	-2.69700400	-0.12464500
C	0.50089600	-2.75443200	-0.08217300
H	-1.28262800	0.67955500	-0.11560700
H	-1.46318900	-3.61101600	-0.14819400
H	1.00441600	-3.71345700	-0.07226400
C	-2.97528600	-1.41011800	-0.18550900
C	-4.19045500	-1.37411300	-0.23147500
C	-6.31659800	-2.91909200	-0.10519100
C	-7.67901400	-3.06450700	-0.09923400
H	-5.69553400	-3.80331800	-0.00100100
C	-7.98996500	-0.67980300	-0.37285300
C	-8.52262900	-1.93054000	-0.23194600
H	-8.14174600	-4.04304000	0.00574800
H	-8.65652400	0.16630500	-0.47935800
H	-9.60034000	-2.03764000	-0.22849600
B	-5.71243400	-1.53409000	-0.25283900
N	-6.63756000	-0.41345500	-0.39610600
Si	-6.15739200	1.34273100	-0.55923900
C	-4.64962700	1.51248200	-1.66735000
H	-4.51226600	2.56808000	-1.92302800
H	-3.73325900	1.14176200	-1.21211700
H	-4.79971300	0.96332500	-2.60128100
C	-5.89571600	2.09752400	1.18877600
C	-7.57372200	2.23227000	-1.44215900
H	-8.47212500	2.36824000	-0.83579000
H	-7.23100900	3.22812500	-1.73861700
H	-7.85939400	1.70352100	-2.35630000
C	-5.63839400	3.61441300	1.03552800

H	-6.48196300	4.13356100	0.57091100
H	-5.48248900	4.06675400	2.02259100
H	-4.74400800	3.82290400	0.44012800
C	-7.15701200	1.88908700	2.05390300
H	-8.04375100	2.35617200	1.61430800
H	-7.37494300	0.82899000	2.20960100
H	-7.01035700	2.34191800	3.04226800
C	-4.69006500	1.46024100	1.90842500
H	-3.75770600	1.60681700	1.35685700
H	-4.56314900	1.92007000	2.89664800
H	-4.81809500	0.38583900	2.05800500
H	1.17839000	0.57739600	-0.04302000
C	2.69255400	-1.64042900	-0.01345900
C	3.90632800	-1.70697800	0.02146500
C	6.06477300	-3.21938100	0.19899500
C	7.48049700	-3.28862000	0.45232000
H	5.45345400	-4.11980400	0.23290900
C	6.77282800	-1.19901500	1.09374700
C	7.93987200	-2.08159500	0.85534200
H	8.09254000	-4.15297800	0.21202300
H	6.36560000	-1.03870100	2.09613300
H	8.94570900	-1.68974500	0.74759300
B	5.42725700	-1.86529500	0.01282600
N	6.30755900	-0.56106900	-0.01859000
Si	5.73419700	1.11933200	-0.20946600
C	4.66934600	1.65392000	1.25545100
H	4.28955400	2.67013500	1.11199700
H	3.81057800	0.98701600	1.36451800
H	5.22288200	1.64254100	2.19893000
C	7.32261200	2.20126200	-0.30930500
C	4.76411800	1.18176600	-1.81568600
H	5.35814200	0.80188200	-2.65074100
H	3.86015900	0.57327900	-1.74625100
H	4.46455600	2.20692900	-2.05288200
C	8.17374700	1.79107500	-1.52902100
H	7.63761600	1.94417600	-2.47013100
H	9.08772300	2.39697000	-1.57243200
H	8.46979100	0.73994900	-1.48216200
C	6.91394000	3.68414500	-0.45731900
H	6.31632400	3.85871700	-1.35724300
H	6.34051000	4.04256700	0.40281200
H	7.80893100	4.31376400	-0.53628100
C	8.17036500	2.04817800	0.97000300
H	9.06195800	2.68500200	0.90990600
H	7.62138600	2.34491000	1.86885000
H	8.52080400	1.02144300	1.11186300

Para-TS2.1

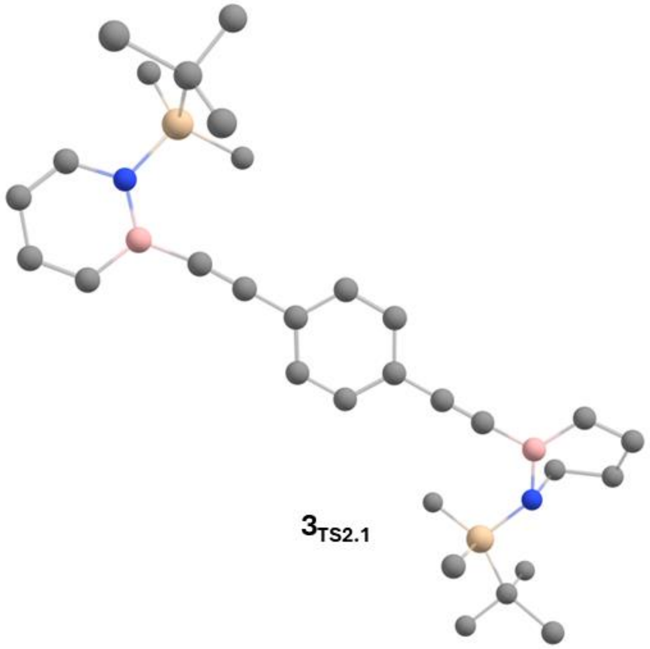

$3_{TS2.1}$

C	1.19103500	-0.47432400	-0.10386400
C	0.84897500	0.89167400	-0.10410900
C	-0.47686900	1.29187000	-0.07120000
C	-1.51708600	0.34278800	-0.03390800
C	-1.17421400	-1.02306900	-0.03471800
C	0.15173400	-1.42360100	-0.07022600
H	-0.72653000	2.34588200	-0.07215900
H	-1.96127200	-1.76687500	-0.01061900
H	0.40110000	-2.47773000	-0.07236700
C	-2.87515900	0.76263300	0.00537400
C	-4.03495000	1.12717800	0.04735100
C	-5.55955500	3.24344600	-0.30205700
C	-6.80360000	3.81689500	-0.33584000
H	-4.69346600	3.85887400	-0.52469400
C	-7.84802000	1.71766800	0.26464900
C	-7.95892100	3.04268800	-0.05177700
H	-6.93367400	4.86904700	-0.57815100
H	-8.74490000	1.15316100	0.48492300
H	-8.94536600	3.48907900	-0.07595300
B	-5.42412800	1.77030700	0.03855900
N	-6.65154800	1.03630300	0.33155000
Si	-6.75167200	-0.74226100	0.74338100
C	-5.32479000	-1.24299300	1.85831600
H	-5.52868500	-2.23606500	2.27157200
H	-4.36344200	-1.26894800	1.34878700
H	-5.23442000	-0.54811600	2.69807200
C	-6.83301600	-1.76860500	-0.87948300
C	-8.33284200	-0.99548200	1.74960500

H	-9.25614600	-0.91535300	1.17137900
H	-8.31268800	-1.99785300	2.18764000
H	-8.38912600	-0.28225600	2.57724700
C	-7.06177400	-3.25243900	-0.51032000
H	-8.00147300	-3.40555500	0.02868100
H	-7.10770700	-3.86008600	-1.42235200
H	-6.25035100	-3.65400800	0.10462400
C	-8.00535200	-1.28496800	-1.75937200
H	-8.97069500	-1.37783600	-1.25227700
H	-7.88302100	-0.24259100	-2.06608800
H	-8.06098500	-1.89048500	-2.67241900
C	-5.52569200	-1.65426700	-1.68915600
H	-4.66144800	-2.01808500	-1.12721000
H	-5.60132600	-2.25756000	-2.60259600
H	-5.31384200	-0.62555500	-1.98946200
H	1.63733000	1.63415200	-0.13091700
C	2.55154300	-0.88867900	-0.13653800
C	3.71235200	-1.24963500	-0.16061200
C	5.45738700	-3.23464700	-0.09254800
C	6.81731200	-3.75577900	-0.11520200
H	4.62304000	-3.90029800	0.13105300
C	6.68201900	-1.74587200	0.83077100
C	7.62556500	-2.79580400	0.36561300
H	7.09805900	-4.71265100	-0.54424200
H	6.39744300	-1.65001900	1.88051500
H	8.69210600	-2.64936200	0.23987400
B	5.15576000	-1.75079500	-0.24414900
N	6.32451600	-0.80047100	-0.12389000
Si	6.33148100	0.97436300	-0.04613600
C	5.46185000	1.58287300	1.51654600
H	5.42817800	2.67608000	1.55122200
H	4.43255100	1.21541100	1.54175500
H	5.95892500	1.24169500	2.42947300
C	8.17035200	1.53676700	-0.04829700
C	5.42936700	1.57062500	-1.58254300
H	5.90777400	1.20301600	-2.49385600
H	4.39628000	1.21550100	-1.58287900
H	5.40952800	2.66351700	-1.62788600
C	8.87603400	1.05284600	-1.33217300
H	8.42493000	1.48248900	-2.23137200
H	9.93101500	1.35504200	-1.32076800
H	8.83978200	-0.03520000	-1.43065500
C	8.23021700	3.07971100	0.00623700
H	7.72871700	3.54129900	-0.84984500
H	7.77545000	3.47853500	0.91798900
H	9.27429600	3.41643600	-0.01004300
C	8.91507800	0.97137500	1.17888300
H	9.95935100	1.30856100	1.17549400
H	8.47069600	1.30446400	2.12165600
H	8.93385900	-0.12278900	1.18333200

Para-IM

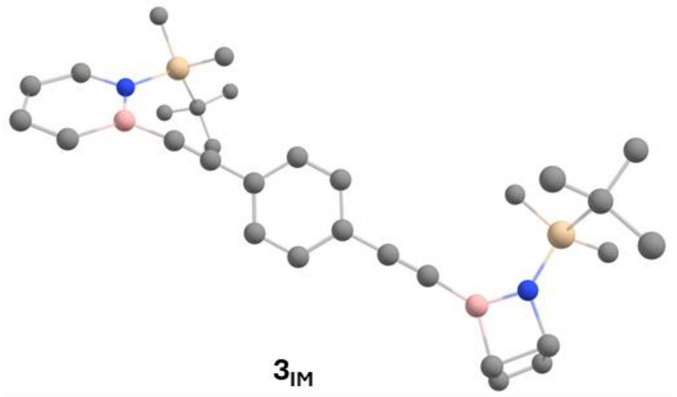

3_{IM}

C	1.59899100	-1.15750700	-0.45157100
C	0.95372000	-2.36741000	-0.12841200
C	-0.41762900	-2.40961700	0.06495600
C	-1.19868000	-1.24459800	-0.06226000
C	-0.55488900	-0.03588000	-0.38932800
C	0.81729400	0.00726300	-0.57858400
H	-0.90147100	-3.34585900	0.31525600
H	-1.14448000	0.86684200	-0.49276200
H	1.29787400	0.94401600	-0.83285300
C	-2.60650300	-1.29044900	0.13414400
C	-3.81101400	-1.32826100	0.30457200
C	-6.28134300	-2.69338900	0.88306800
C	-6.74998100	-2.78555600	2.32761700
H	-6.30607000	-3.59131200	0.26403100
C	-7.38300200	-1.57927100	0.75022000
C	-7.67203000	-1.81585400	2.22572700
H	-6.40424500	-3.39713000	3.15407100
H	-8.19542200	-1.66499500	0.02771100
H	-8.32149200	-1.31646200	2.93476000
B	-5.30369100	-1.46478600	0.51923800
N	-6.36033400	-0.52327100	0.43159700
Si	-6.60550800	1.21658100	0.18320000
C	-8.11480100	1.70935900	1.20216900
H	-8.31652000	2.78046300	1.11017500
H	-9.01791200	1.17481900	0.89434200
H	-7.94465900	1.49559600	2.26143900
C	-6.89574600	1.57391600	-1.68459300
C	-5.07643700	2.11566600	0.81420600
H	-4.95482900	1.94505900	1.88777400
H	-4.16762800	1.76677900	0.31861000
H	-5.15867800	3.19482000	0.65395500
C	-7.20713500	3.07248400	-1.88931900
H	-6.39079200	3.71410900	-1.54335300
H	-7.35581200	3.28369600	-2.95588300
H	-8.11923900	3.37839300	-1.36800800
C	-5.63370300	1.20834400	-2.49334800
H	-4.77046600	1.81303700	-2.20021600

H	-5.36132700	0.15602800	-2.37168300
H	-5.80719400	1.38310300	-3.56288800
C	-8.08273700	0.73998000	-2.21065900
H	-9.01381900	0.96783800	-1.68245300
H	-8.25169800	0.95284900	-3.27397200
H	-7.89551200	-0.33384100	-2.11960400
H	1.54321300	-3.27071200	-0.02997200
C	3.00806000	-1.11875800	-0.64617800
C	4.21355900	-1.07832700	-0.80365400
C	6.25054200	-2.49137000	-1.69083400
C	7.59574200	-2.61491700	-1.92840400
H	5.59577400	-3.31606300	-1.95556500
C	8.01101400	-0.40282600	-1.03782300
C	8.48299200	-1.55845900	-1.59712200
H	8.00959800	-3.51707500	-2.37271700
H	8.70984500	0.38805300	-0.79791100
H	9.54613600	-1.64805500	-1.78294600
B	5.71675200	-1.21254000	-1.07266200
N	6.68297800	-0.16608600	-0.75824200
Si	6.29998900	1.42849400	0.05920200
C	4.65840600	2.11000400	-0.55088600
H	4.58103700	2.02359600	-1.63802200
H	3.79744600	1.60789100	-0.11265400
H	4.60674100	3.17341200	-0.29641600
C	6.32973200	1.17974300	1.96363800
C	7.63171800	2.66019300	-0.46866000
H	7.73536900	2.67939800	-1.55764000
H	7.32424600	3.66198200	-0.15411100
H	8.61793200	2.47500000	-0.03761300
C	6.07316200	2.54818100	2.63645400
H	5.09719900	2.96283400	2.36632000
H	6.08687800	2.43399100	3.72727100
H	6.83788100	3.28678000	2.37779800
C	5.23983600	0.18869100	2.42044800
H	4.23563400	0.52391100	2.14832900
H	5.38432900	-0.80656200	1.99285400
H	5.26799000	0.08605500	3.51253200
C	7.70573600	0.64816500	2.41810500
H	8.52035600	1.33141300	2.15963500
H	7.71685000	0.52914400	3.50857300
H	7.93113600	-0.32901500	1.98168000

Para-TS1.2

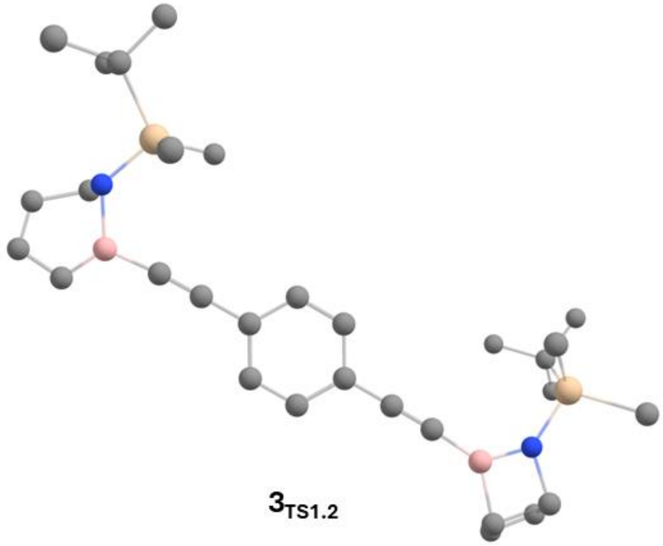

$3_{TS1.2}$

C	0.52499997	-0.17500000	0.00000000
C	1.28537997	1.00964700	-0.08145900
C	2.66556497	0.96050500	-0.18468500
C	3.34380797	-0.27414900	-0.20638200
C	2.58540397	-1.45788800	-0.12311100
C	1.20433097	-1.40973600	-0.02403400
H	3.23708297	1.87851100	-0.24806700
H	3.09385597	-2.41423400	-0.13821300
H	0.63620697	-2.32981600	0.03812900
C	4.76085497	-0.32025400	-0.31023500
C	5.97453197	-0.35606900	-0.39825400
C	8.55444297	0.86909000	-0.76667400
C	9.34089497	1.34751000	0.44411300
H	8.46878597	1.53014600	-1.63042200
C	9.56358897	-0.33658600	-0.76463400
C	10.18844997	0.30729800	0.46556800
H	9.21184297	2.21284600	1.08527800
H	10.19249697	-0.55418900	-1.62858500
H	10.97227297	-0.00992600	1.14282700
B	7.48186297	-0.30938600	-0.52804800
N	8.46276397	-1.33461400	-0.52781600
Si	8.55925897	-3.10240800	-0.38764200
C	7.10150397	-3.82560400	-1.33737400
H	7.17195897	-3.55657000	-2.39536800
H	7.08254797	-4.91734100	-1.26993900
H	6.14814797	-3.44524900	-0.96282100
C	8.51660997	-3.66008200	1.45333300
C	10.18011897	-3.61469500	-1.20533600
H	11.05332697	-3.18979400	-0.70236800
H	10.29238897	-4.70266900	-1.20132500
H	10.20314897	-3.28629000	-2.24882800
C	8.66780697	-5.19527300	1.53205500

H	8.63198997	-5.52232000	2.57897900
H	7.86473597	-5.71693700	1.00243300
H	9.62131897	-5.53730300	1.11825100
C	9.66869497	-3.00357100	2.24121700
H	9.58824097	-1.91334000	2.24100500
H	9.64705797	-3.33528900	3.28724500
H	10.64973797	-3.27135000	1.83632500
C	7.17615697	-3.25550500	2.10133900
H	7.01525897	-2.17439700	2.06863200
H	6.32340597	-3.73513400	1.61187000
H	7.16130397	-3.55944600	3.15590300
H	0.77648497	1.96556100	-0.06363200
C	-0.89019803	-0.11592100	0.10516900
C	-2.10219403	-0.04222100	0.19846700
C	-4.24893203	1.44529000	0.62584800
C	-5.66678003	1.43926300	0.91062000
H	-3.68836903	2.37411400	0.65188100
C	-5.08848603	-0.81621400	1.35423000
C	-6.15408003	0.22960600	1.24244800
H	-6.30008003	2.31024700	0.76664100
H	-4.89923203	-1.30960800	2.31658400
H	-7.19256203	-0.08582300	1.22216000
B	-3.60533503	0.15893900	0.30921900
N	-4.48230603	-1.20604300	0.24394400
Si	-3.85346303	-2.86840600	-0.10513300
C	-2.77574003	-3.47786900	1.31458800
H	-2.35497803	-4.46017900	1.07948400
H	-1.94618103	-2.78751100	1.48410200
H	-3.33063703	-3.57514800	2.25178200
C	-5.42878703	-3.95767100	-0.29285300
C	-2.91076503	-2.76917200	-1.72229000
H	-3.50834403	-2.28073200	-2.49608600
H	-1.98457003	-2.20465600	-1.60811500
H	-2.65657803	-3.77269500	-2.07610300
C	-6.28269903	-3.94101600	0.99041700
H	-7.14790603	-4.60464900	0.87146900
H	-5.72615503	-4.28798400	1.86609500
H	-6.68169203	-2.94617100	1.21286500
C	-6.28853203	-3.46716200	-1.47712200
H	-6.62471103	-2.43603300	-1.33903100
H	-5.74457303	-3.51673000	-2.42450900
H	-7.18019403	-4.09814500	-1.57907600
C	-4.98149203	-5.41307000	-0.56529000
H	-4.39896303	-5.82806000	0.26253500
H	-5.86224303	-6.05317200	-0.69694300
H	-4.38202803	-5.49735400	-1.47646300

Para-im1

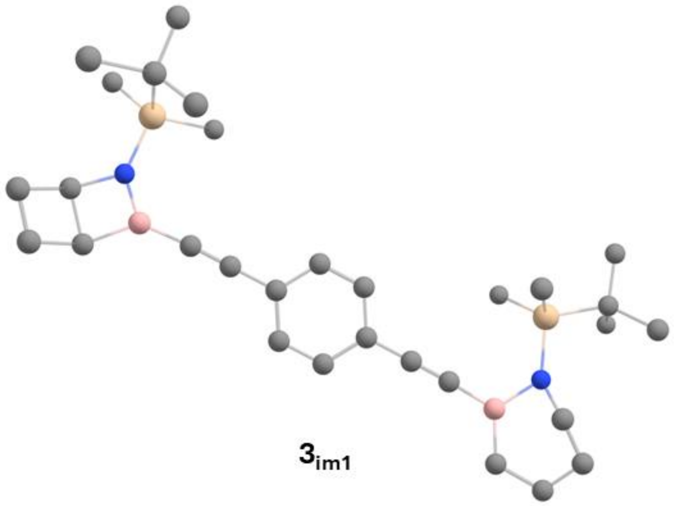

3_{im1}

C	1.44758600	-1.58846500	-0.14656300
C	0.69410700	-2.77604700	-0.22716000
C	-0.68908400	-2.73563900	-0.28857700
C	-1.37562100	-1.50616700	-0.27135600
C	-0.62335800	-0.31902400	-0.18998000
C	0.76019100	-0.35935800	-0.12901400
H	-1.25770900	-3.65552500	-0.35041500
H	-1.13969700	0.63303700	-0.17504700
H	1.32565100	0.56247000	-0.06731000
C	-2.79523400	-1.46803100	-0.33428600
C	-4.01054400	-1.43870400	-0.38615400
C	-6.61157200	-2.67441800	-0.47935400
C	-7.34216600	-2.97462800	0.82024700
H	-6.57096500	-3.44742400	-1.24816500
C	-7.61248500	-1.46751900	-0.59423700
C	-8.18135900	-1.93111100	0.73978400
H	-7.18787500	-3.74638400	1.56650400
H	-8.28132700	-1.36164700	-1.44923100
H	-8.93025600	-1.51619800	1.40355000
B	-5.52131000	-1.48876000	-0.45019100
N	-6.49558400	-0.46082900	-0.54687600
Si	-6.56985400	1.30957600	-0.64313100
C	-5.17452400	1.87526400	-1.77576500
H	-5.32419200	1.47884900	-2.78427700
H	-5.13088900	2.96596500	-1.84875900
H	-4.20328700	1.51867200	-1.42446600
C	-6.40192800	2.11126900	1.09762600
C	-8.23935600	1.73299400	-1.41444100
H	-9.07995000	1.38953400	-0.80493600
H	-8.34720200	2.81335200	-1.54667500
H	-8.33466200	1.27214400	-2.40227500
C	-6.55462700	3.64370400	0.97992800
H	-6.44479800	4.10922200	1.96756300

H	-5.79443600	4.08587400	0.32855000
H	-7.53715300	3.93225400	0.59412100
C	-7.49269900	1.57139100	2.04524600
H	-7.40903100	0.49021000	2.18328300
H	-7.39824400	2.03793400	3.03418200
H	-8.50129400	1.78982900	1.67998600
C	-5.01742600	1.79158900	1.69884500
H	-4.85051700	0.71550800	1.79675400
H	-4.20401600	2.20138600	1.09280200
H	-4.93151700	2.23136100	2.70076900
H	1.21121500	-3.72766200	-0.24118700
C	2.86766100	-1.63509600	-0.08606900
C	4.08140800	-1.68567300	-0.03218200
C	6.25826500	-3.17136800	0.13684500
C	7.66950700	-3.22631200	0.41764200
H	5.66104800	-4.08176200	0.13009700
C	6.91665100	-1.16560900	1.10565300
C	8.10113000	-2.02538400	0.86627000
H	8.30023900	-4.07335000	0.16461200
H	6.48941600	-1.03649100	2.10422900
H	9.10349300	-1.61675600	0.79480100
B	5.60495100	-1.82162200	-0.01875600
N	6.46564100	-0.50347700	0.00215500
Si	5.87114300	1.17378100	-0.15378800
C	4.77380000	1.65281900	1.30621200
H	4.38145600	2.66693200	1.18320300
H	3.92312600	0.97123400	1.38298700
H	5.31129500	1.62431800	2.25853700
C	7.44526200	2.27955900	-0.19632000
C	4.92805700	1.26550800	-1.77455100
H	5.54235300	0.91833200	-2.60917300
H	4.03254000	0.64184200	-1.73829400
H	4.61652500	2.29218500	-1.98860600
C	8.27297000	2.10362100	1.09316200
H	9.15575000	2.75476200	1.06617100
H	7.70424600	2.36754100	1.98997400
H	8.63641800	1.07859700	1.21302700
C	8.32323000	1.91496600	-1.41152800
H	8.63372500	0.86722400	-1.38806000
H	7.80134300	2.08636700	-2.35746900
H	9.22884400	2.53465100	-1.42219300
C	7.01748700	3.76004900	-0.31132700
H	6.42475400	4.08696200	0.54824900
H	7.90445900	4.40400700	-0.35808400
H	6.43248800	3.95062700	-1.21626500

Para-TS1.1

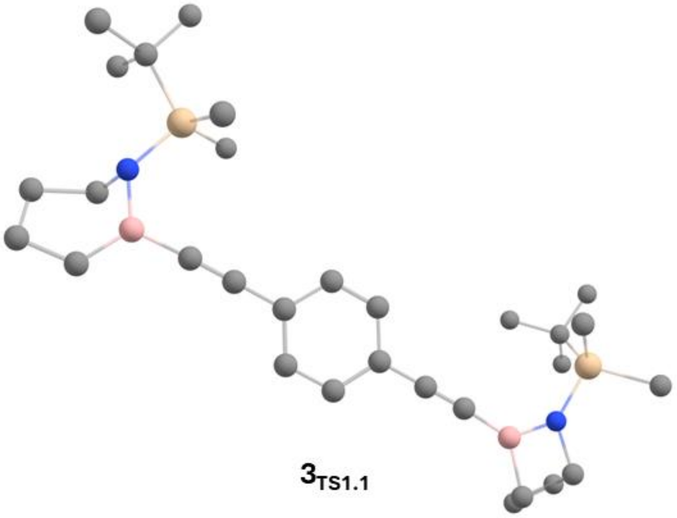

C	1.39500100	-1.55511300	-0.18260900
C	0.64393300	-2.74490100	-0.12034200
C	-0.74035600	-2.71193600	-0.15895600
C	-1.42884400	-1.48783000	-0.26194600
C	-0.67849100	-0.29833800	-0.32417400
C	0.70610200	-0.33137700	-0.28492500
H	-1.30807500	-3.63314100	-0.11060900
H	-1.19684700	0.64940800	-0.40321000
H	1.27132100	0.59134900	-0.33370100
C	-2.84962600	-1.45789300	-0.30277000
C	-4.06563200	-1.43693900	-0.33650200
C	-6.65820800	-2.69345200	-0.34249600
C	-7.37392500	-2.94033300	0.97648100
H	-6.61937800	-3.49987400	-1.07626900
C	-7.66941500	-1.50017000	-0.50126000
C	-8.22193600	-1.90768500	0.85768700
H	-7.20673100	-3.67690400	1.75481600
H	-8.34715200	-1.43757200	-1.35349400
H	-8.96804500	-1.46922700	1.50930000
B	-5.57709700	-1.49980300	-0.37868400
N	-6.55970500	-0.48425400	-0.51059400
Si	-6.64815300	1.27974300	-0.68535000
C	-5.25389500	1.80556300	-1.83818900
H	-5.39690100	1.36331400	-2.82842700
H	-5.21950400	2.89226700	-1.95952200
H	-4.28083800	1.47356800	-1.46844300
C	-6.49182200	2.15917600	1.01850500
C	-8.31836600	1.65428400	-1.48014400
H	-9.15862500	1.33660200	-0.85635900
H	-8.43227200	2.72600600	-1.66639800
H	-8.40817900	1.14422500	-2.44402200
C	-6.65633000	3.68349400	0.83190100

H	-6.55473900	4.19352400	1.79817700
H	-5.89698800	4.10266900	0.16453000
H	-7.63951600	3.94599800	0.42959200
C	-7.58095500	1.65363900	1.98666700
H	-7.48772300	0.58085900	2.17482100
H	-7.49406800	2.16617900	2.95325900
H	-8.59024900	1.84561500	1.60873000
C	-5.10660300	1.87716000	1.63668200
H	-4.93212900	0.80767300	1.78255200
H	-4.29445500	2.26584500	1.01528700
H	-5.02671500	2.36127400	2.61844300
H	1.16335100	-3.69215000	-0.04238100
C	2.81652700	-1.59379200	-0.14388200
C	4.03095900	-1.63737400	-0.10879100
C	6.22239500	-3.09225600	0.14855000
C	7.67052300	-3.24531900	0.19636000
H	5.57762900	-3.93409200	0.40307800
C	6.98275900	-1.28394000	0.99051000
C	8.18405400	-2.08107200	0.62750500
H	8.20452300	-4.12245000	-0.15548500
H	6.63899600	-1.19624700	2.02307500
H	9.18263600	-1.67296700	0.52290600
B	5.55769200	-1.75004200	-0.11931900
N	6.43708700	-0.52537200	-0.03961500
Si	5.99196800	1.19350600	-0.09468200
C	4.93643700	1.66252000	1.40012200
H	4.62750500	2.71125300	1.35358700
H	4.03239000	1.04848500	1.43066600
H	5.46706200	1.51948500	2.34608300
C	7.62686800	2.20526700	-0.10148200
C	5.02792100	1.43524000	-1.68912600
H	5.61680900	1.13787400	-2.56046000
H	4.11693600	0.83234900	-1.68231600
H	4.73751200	2.48207400	-1.81824500
C	8.44433100	1.93000500	1.17769300
H	9.36821600	2.52204500	1.17019900
H	7.89529200	2.19901400	2.08513200
H	8.73999500	0.87975400	1.26158700
C	8.47934400	1.83506400	-1.33309600
H	8.72327500	0.76974300	-1.35367800
H	7.96815200	2.07658200	-2.26958900
H	9.42237000	2.39627100	-1.32277300
C	7.29219500	3.71268300	-0.15781800
H	6.71829900	4.04132400	0.71386000
H	8.21701800	4.30257500	-0.17884400
H	6.72208100	3.97429700	-1.05446700

Para-Dewar

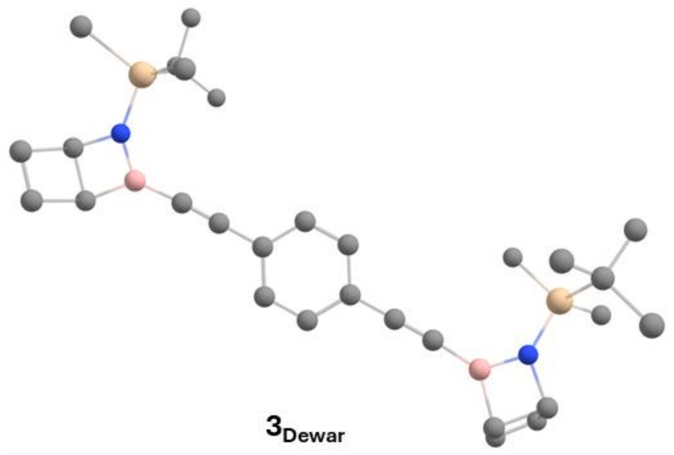

3_{Dewar}

C	-1.41082200	-1.42494800	0.03038800
C	-0.69260600	-2.63633100	0.01435800
C	0.69260600	-2.63633100	-0.01435900
C	1.41082200	-1.42494800	-0.03038900
C	0.69263700	-0.21382200	-0.01546300
C	-0.69263700	-0.21382200	0.01546300
H	1.23557200	-3.57339900	-0.02555500
H	1.23475400	0.72366700	-0.02736500
H	-1.23475400	0.72366700	0.02736500
C	2.83287800	-1.42711600	-0.06064100
C	4.04934000	-1.42811900	-0.08969400
C	6.62065000	-2.72607000	0.00533300
C	7.26259000	-3.18642100	-1.29516000
H	6.60476800	-3.41046200	0.85467700
C	7.65603600	-1.54509300	-0.07243600
C	8.12895600	-2.16512200	-1.37942900
H	7.04192500	-4.02800200	-1.94289500
H	8.37802300	-1.36710100	0.72531500
H	8.84036900	-1.84067300	-2.12940100
B	5.56195600	-1.51611400	-0.10150300
N	6.56344800	-0.51638700	-0.17848400
Si	6.71254400	1.23625500	-0.41822100
C	8.31592400	1.52428100	-1.36916300
H	8.46681900	2.58909400	-1.56858800
H	9.19292700	1.16177500	-0.82551900
H	8.28409300	1.00935900	-2.33377800
C	6.76065800	2.12419700	1.28740500
C	5.23946400	1.81093600	-1.43982100
H	5.25637500	1.33761500	-2.42577300
H	4.29045600	1.55207900	-0.96476700
H	5.26097200	2.89464200	-1.58858300
C	6.99284400	3.63726600	1.08380200
H	6.20311800	4.09828100	0.48247600
H	7.00309300	4.15029200	2.05381400
H	7.95054900	3.84490000	0.59706100

C	5.42293300	1.91673200	2.02758300
H	4.58176600	2.35486300	1.48236700
H	5.20350000	0.85682000	2.18529100
H	5.45889600	2.39684100	3.01385000
C	7.90302600	1.55485200	2.15458500
H	8.88283200	1.68641500	1.68503300
H	7.93474500	2.07038500	3.12292700
H	7.76499800	0.48898200	2.35783700
H	-1.23557200	-3.57339900	0.02555400
C	-2.83287800	-1.42711600	0.06064100
C	-4.04934000	-1.42811900	0.08969300
C	-6.62065000	-2.72607000	-0.00533200
C	-7.26259000	-3.18642100	1.29516000
H	-6.60476900	-3.41046200	-0.85467600
C	-7.65603600	-1.54509300	0.07243700
C	-8.12895600	-2.16512200	1.37943000
H	-7.04192400	-4.02800200	1.94289600
H	-8.37802300	-1.36710100	-0.72531400
H	-8.84036800	-1.84067300	2.12940200
B	-5.56195600	-1.51611400	0.10150300
N	-6.56344700	-0.51638700	0.17848400
Si	-6.71254400	1.23625500	0.41822100
C	-5.23946300	1.81093600	1.43982100
H	-5.25637500	1.33761600	2.42577300
H	-4.29045500	1.55207900	0.96476600
H	-5.26097200	2.89464200	1.58858300
C	-6.76065800	2.12419600	-1.28740500
C	-8.31592400	1.52428100	1.36916300
H	-8.28409200	1.00935900	2.33377800
H	-8.46681900	2.58909400	1.56858800
H	-9.19292700	1.16177500	0.82552000
C	-6.99284400	3.63726600	-1.08380200
H	-6.20311800	4.09828100	-0.48247700
H	-7.00309400	4.15029100	-2.05381500
H	-7.95054900	3.84490000	-0.59706100
C	-5.42293300	1.91673200	-2.02758400
H	-4.58176600	2.35486300	-1.48236700
H	-5.20350000	0.85682000	-2.18529100
H	-5.45889700	2.39684100	-3.01385000
C	-7.90302600	1.55485200	-2.15458500
H	-8.88283200	1.68641500	-1.68503300
H	-7.93474500	2.07038500	-3.12292700
H	-7.76499800	0.48898100	-2.35783700

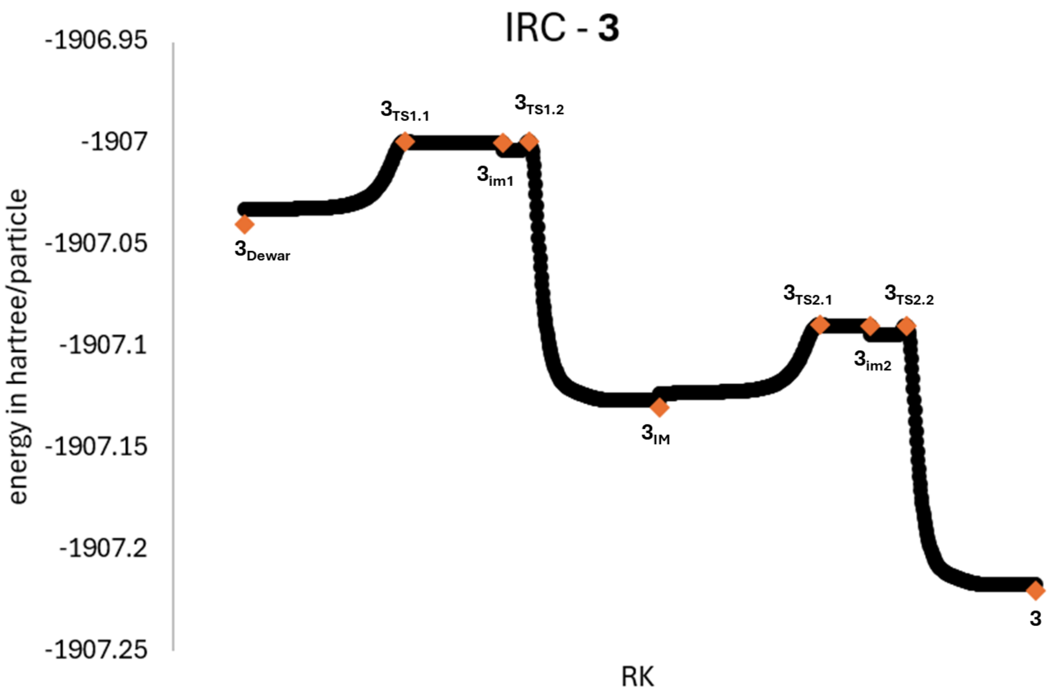

Figure S55. Intrinsic reaction coordinates calculated in both directions from the transition states $\mathbf{3}_{\mathbf{TS1.1}}$, $\mathbf{3}_{\mathbf{TS1.2}}$, $\mathbf{3}_{\mathbf{TS2.1}}$ and $\mathbf{3}_{\mathbf{TS2.2}}$. The corresponding calculated geometries of the transition states as well as the local minima are given in orange. The offset of the IRC's at the intermediates $\mathbf{3}_{\mathbf{im1}}$ and $\mathbf{3}_{\mathbf{im2}}$ are due to different rotamers along the C-C triple bond or the *tert*-butyldimethylsilyl group.

Rotamers

As previously computed, a rotation of the tert-butyldimethylsilyl group on nitrogen can cause a change up to 4 kcal/mol in the total energy.[6] The compounds under consideration also allow for rotation around the C-C triple bonds. Using scan calculations (M062X/6-311+G(d,p)), we were able to estimate the energy differences associated with this rotation as significantly lower (< 1 kcal/mol). An exception is the ortho isomer **1**. Due to the proximity of the two dihydroazaborinin units, strong repulsive interactions are expected for some conformers. This is reflected in the significantly higher energy of these rotamers.

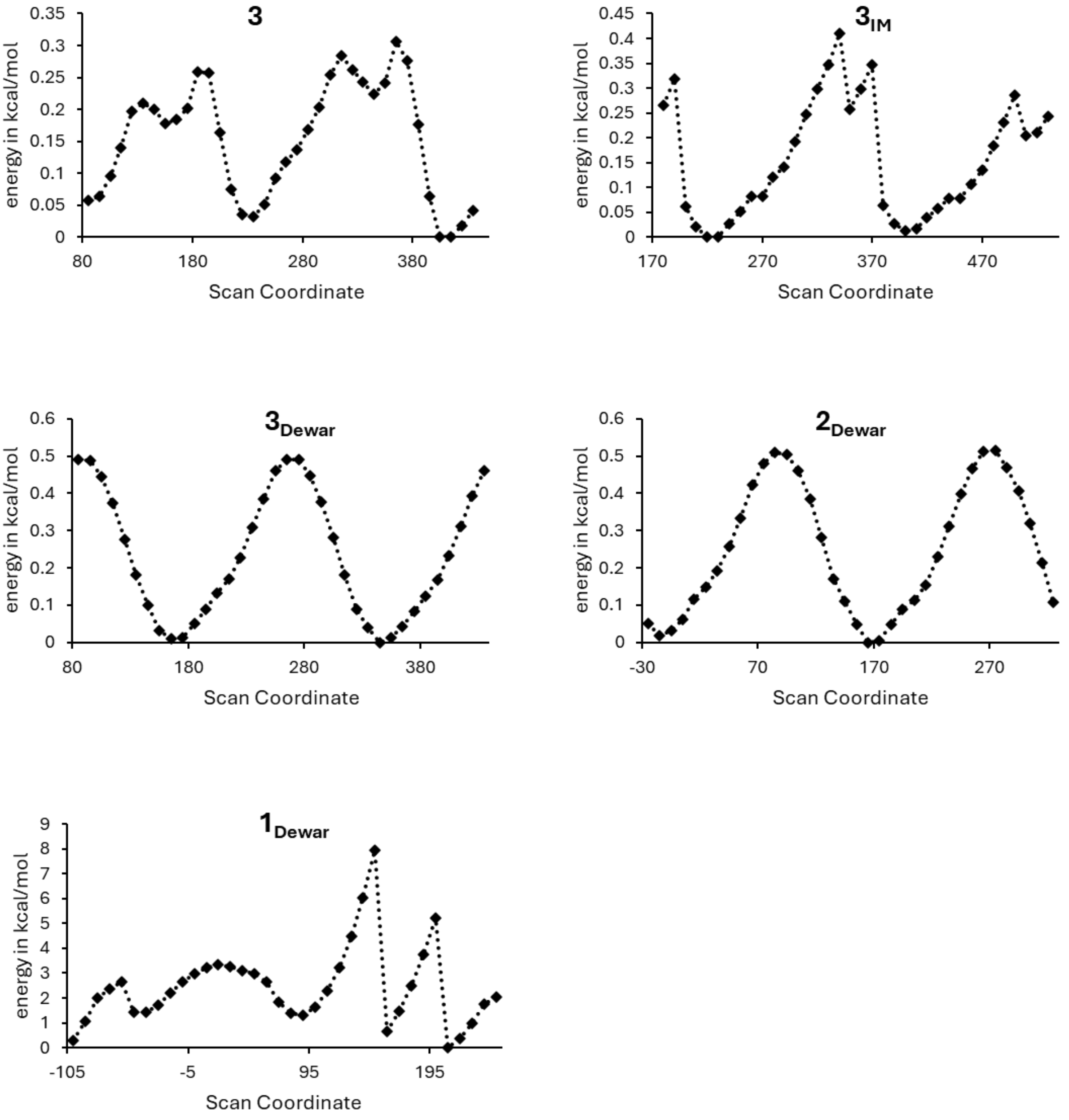

Figure S56. Scan of the rotation along a C-C triple bond of the molecules. The dihedral angle was scanned in 10 ° steps.

References

1. G. M. Sheldrick, *Acta Crystallogr., Sect. A*, 2008, **2008**, 112-122.
2. C. B. H. G. M. S. B. Dittrich, ShelXle: a Qt graphical user interface for SHELXL, *J. Appl. Crystallogr.*, 2011, **44**.
3. G. Sheldrick, Crystal structure refinement with SHELXL, *Acta Crystallographica Section C*, 2015, **71**, 3-8.
4. O. Dolomanov, L. Bourhis, R. Gildea, J. Howard and H. Puschmann, OLEX2: A complete structure solution, refinement and analysis program, *J. Appl. Cryst. J. Appl. Cryst*, 2009, **42**, 339-341.
5. L. J. Bourhis, O. V. Dolomanov, R. J. Gildea, J. A. Howard and H. Puschmann, The anatomy of a comprehensive constrained, restrained refinement program for the modern computing environment - Olex2 dissected, *Acta Crystallogr A Found Adv*, 2015, **71**, 59-75.
6. R. C. Richter, S. M. Biebl, R. Einholz, J. Walz, C. Maichle-Mössmer, M. Ströbele, H. F. Bettinger and I. Fleischer, Inside Cover: Facile Energy Release from Substituted Dewar Isomers of 1,2-Dihydro-1,2-Azaborinines Catalyzed by Coinage Metal Lewis Acids (Angew. Chem. Int. Ed. 30/2024), *Angew. Chem. Int. Ed.*, 2024, **63**, e202411078.
7. A. J. V. Marwitz, A. N. Lamm, L. N. Zakharov, M. Vasiliu, D. A. Dixon and S.-Y. Liu, BN-substituted diphenylacetylene: a basic model for conjugated π-systems containing the BN bond pair, *Chem. Sci.*, 2012, **3**, 825-829.
8. K. Edel, X. Yang, J. S. A. Ishibashi, A. N. Lamm, C. Maichle-Mössmer, Z. X. Giustra, S.-Y. Liu and H. F. Bettinger, The Dewar Isomer of 1,2-Dihydro-1,2-azaborinines: Isolation, Fragmentation, and Energy Storage, *Angew. Chem. Int. Ed.*, 2018, **57**, 5296-5300.
9. F. Kleemiss, O. V. Dolomanov, M. Bodensteiner, N. Peyerimhoff, L. Midgley, L. J. Bourhis, A. Genoni, L. A. Malaspina, D. Jayatilaka, J. L. Spencer, F. White, B. Grundkötter-Stock, S. Steinhauer, D. Lentz, H. Puschmann and S. Grabowsky, Accurate crystal structures and chemical properties from NoSpherA2, *Chem. Sci.*, 2021, **12**, 1675-1692.
10. Y. Zhao and D. G. Truhlar, The M06 suite of density functionals for main group thermochemistry, thermochemical kinetics, noncovalent interactions, excited states, and transition elements: two new functionals and systematic testing of four M06-class functionals and 12 other functionals, *Theor. Chem. Acc.*, 2008, **120**, 215-241.
11. R. Ditchfield, W. J. Hehre and J. A. Pople, Self-Consistent Molecular-Orbital Methods. IX. An Extended Gaussian-Type Basis for Molecular-Orbital Studies of Organic Molecules, *The Journal of Chemical Physics*, 1971, **54**, 724-728.
12. W. J. Hehre, R. Ditchfield and J. A. Pople, Self—Consistent Molecular Orbital Methods. XII. Further Extensions of Gaussian—Type Basis Sets for Use in Molecular Orbital Studies of Organic Molecules, *The Journal of Chemical Physics*, 1972, **56**, 2257-2261.
13. M. M. Francl, W. J. Pietro, W. J. Hehre, J. S. Binkley, M. S. Gordon, D. J. DeFrees and J. A. Pople, Self-consistent molecular orbital methods. XXIII. A polarization-type basis set for second-row elements, *The Journal of Chemical Physics*, 1982, **77**, 3654-3665.
14. F. Neese, The ORCA program system, *WIREs Computational Molecular Science*, 2012, **2**, 73-78.
15. A. D. Becke, Density-functional thermochemistry. III. The role of exact exchange, *The Journal of Chemical Physics*, 1993, **98**, 5648-5652.
16. C. Lee, W. Yang and R. G. Parr, Development of the Colle-Salvetti correlation-energy formula into a functional of the electron density, *Physical Review B*, 1988, **37**, 785-789.
17. A. D. McLean and G. S. Chandler, Contracted Gaussian basis sets for molecular calculations. I. Second row atoms, Z=11–18, *The Journal of Chemical Physics*, 1980, **72**, 5639-5648.
18. M. J. Frisch, G. W. Trucks, H. B. Schlegel, G. E. Scuseria, M. A. Robb, J. R. Cheeseman, G. Scalmani, V. Barone, G. A. Petersson, H. Nakatsuji, X. Li, M. Caricato, A. V. Marenich, J. Bloino, B. G. Janesko,

R. Gomperts, B. Mennucci, H. P. Hratchian, J. V. Ortiz, A. F. Izmaylov, J. L. Sonnenberg, Williams, F. Ding, F. Lipparini, F. Egidi, J. Goings, B. Peng, A. Petrone, T. Henderson, D. Ranasinghe, V. G. Zakrzewski, J. Gao, N. Rega, G. Zheng, W. Liang, M. Hada, M. Ehara, K. Toyota, R. Fukuda, J. Hasegawa, M. Ishida, T. Nakajima, Y. Honda, O. Kitao, H. Nakai, T. Vreven, K. Throssell, J. A. Montgomery Jr., J. E. Peralta, F. Ogliaro, M. J. Bearpark, J. J. Heyd, E. N. Brothers, K. N. Kudin, V. N. Staroverov, T. A. Keith, R. Kobayashi, J. Normand, K. Raghavachari, A. P. Rendell, J. C. Burant, S. S. Iyengar, J. Tomasi, M. Cossi, J. M. Millam, M. Klene, C. Adamo, R. Cammi, J. W. Ochterski, R. L. Martin, K. Morokuma, O. Farkas, J. B. Foresman and D. J. Fox, Gaussian 16 Rev. C.01.*Journal*, 2016.
